# Highly Stereoselective Ugi/Pictet–Spengler Sequence

**DOI:** 10.1021/acs.joc.2c00244

**Published:** 2022-05-12

**Authors:** Bidong Zhang, Katarzyna Kurpiewska, Alexander Dömling

**Affiliations:** † Department of Drug Design, University of Groningen, A. Deusinglaan 1, 9713 AV Groningen, The Netherlands; ‡ Department of Crystal Chemistry and Crystal Physics Faculty of Chemistry, Jagiellonian University, Gronostajowa 2, 30-387 Kraków, Poland

## Abstract

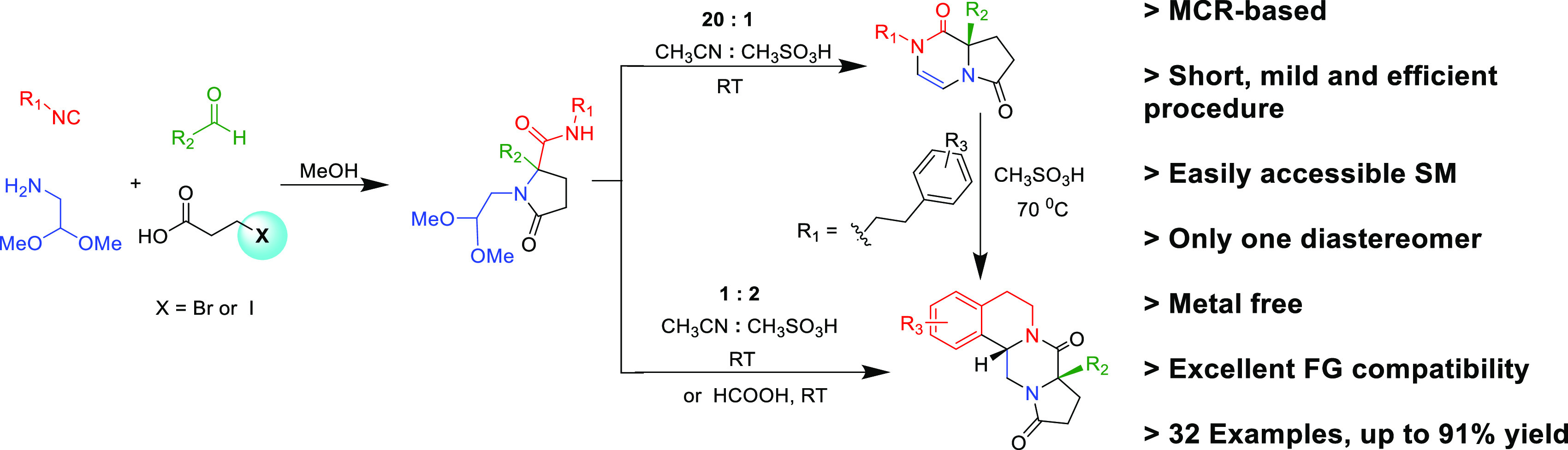

Discovering novel synthetic routes for rigid nitrogen-containing polyheterocycles using sustainable, atom-economical, and efficient (= short) synthetic pathways is of high interest in organic chemistry. Here, we describe an operationally simple and short synthesis of the privileged scaffold dihydropyrrolo[1,2-*a*]pyrazine-dione from readily accessible starting materials. The alkaloid-type polycyclic scaffold with potential bioactivity was achieved by a multicomponent reaction (MCR)-based protocol via a Ugi four-component reaction and Pictet–Spengler sequence under different conditions, yielding a diverse library of products.

## Introduction

**Figure 1:**
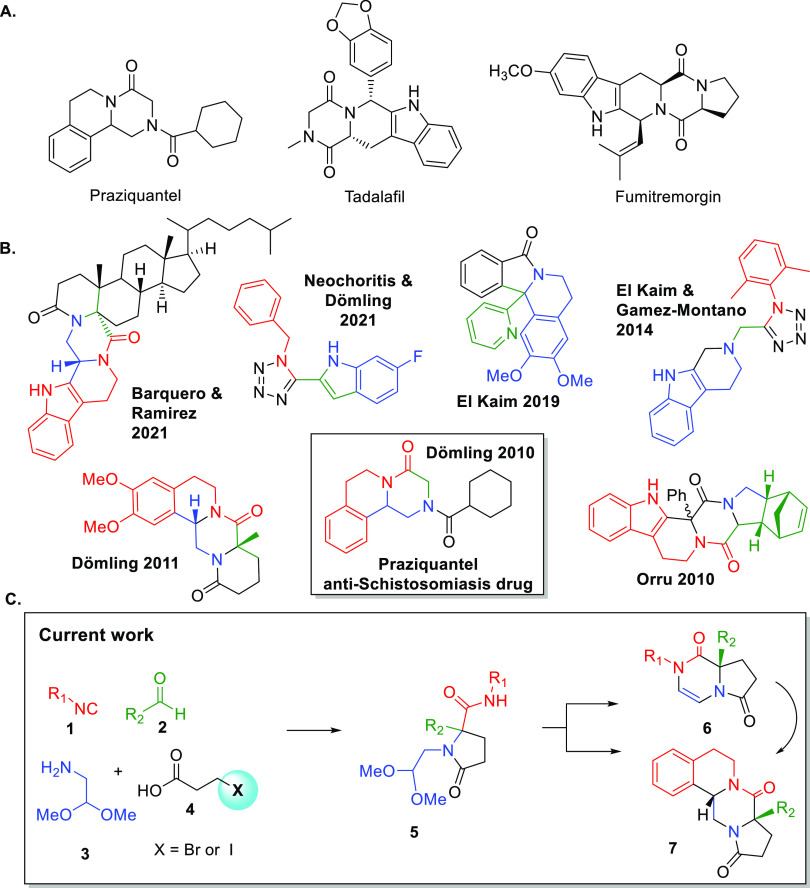
(A) Polyheterocyclic drugs. (B) Some previous and (C) current work on Ugi/Pictet–Spengler sequences for the synthesis of polycyclic scaffolds (the colors indicate the origin of atoms from different starting material classes: red, isocyanide; green, oxo component; blue, amine; and black, acid component).

Heterocyclic compounds, particularly nitrogen(N)-containing heterocycles, have important roles in organic chemistry due to the application in almost all branches of organic chemistry including pharmaceutical research, functional materials, catalysis, and coordination chemistry.^1^ Among the N-containing heterocycles, polyheterocycles as constituents of diverse natural alkaloids and pharmaceutical agents have drawn much attention from organic and bioorganic chemists during the past decades.^2^ Some of them are an integral part of many biologically active molecules, and many currently marketed drugs hold polyheterocycles as their core structure, such as praziquantel (anthelmintic agent),^3^ tadalafil (male erectile dysfunction),^4^ or fumitremorgin C (BCRP specific inhibitor)^5^ (Figure 1A). Polyheterocycles due to their conformational stiffness often have very good receptor binding and membrane diffusion properties. Therefore, the quest for the novel synthetic routes for nitrogen (N)-containing polyheterocycles using sustainable, atom-economical, and efficient pathways is in high demand.^6^ Multicomponent reactions (MCR) are one of the most powerful tools to synthesize novel scaffold compounds for drug discovery in general and polyheterocycles specifically.^7^ The wide functional group compatibility of isocyanide-based MCRs allows for the introduction of orthogonal groups, which can be further cyclized.^8^ One such strategy is the Pictet–Spengler reaction of electron-rich β-arylethylamine undergoing condensation with an aldehyde or ketone followed by ring closure. The Pictet–Spengler reaction not only has been explored as a convenient method for the asymmetric synthesis of isoquinoline alkaloids^9^ but also was widely used for the synthesis of alkaloid-like polycyclic compounds by combining with MCR chemistry in recent years (Figure 1B).^10^

The union of the Ugi reaction with a Pictet–Spengler-type ring closure is very popular and has recently led to a sustainable and efficient access to 2-tetrazolo-substituted indoles,^11^ steroidal pyrazinoisoquinolines,^12^ isoindolinones,^13^ 2-tetrazolylmethyl-2,3,4,9-tetrahydro-1*H*-β-carbolines,^14^ praziquantel and derivatives,^15^ polycycles,^16^ or chiral-pure synthetic alkaloids,^17^ among others, and is reviewed (Figure 1B).^18^

## Results and Discussion

Inspired by Orru’s^17^ and our own work,^19^ we report here the highly stereoselective synthesis of 8,8*a*-dihydropyrrolo[1,2-*a*]pyrazine-1,6(2*H*,7*H*)-dione species **6** by a Ugi/Pictet–Spengler sequence starting from isocyanides **1**, aldehydes **2**, 2,2-dimethoxyethan-1-amine **3**, and 3-bromopropanoic acids **4** with excellent stereoselectivity in the final target **7**. Moreover, we achieved the post-transformation of **6** to novel fused tricyclic systems **7** by treatment with methanesulfonic acid (Figure 1). To start, the Ugi-4CR of (2-isocyanoethyl)benzene **1a**, picolinaldehyde **2a**, 2,2-dimethoxyethan-1-amine **3a**, and 3-bromopropanoic acid **4a** in methanol at room temperature resulted in the expected Ugi-adduct **5a** in an excellent yield of 98% after 15 h. With compound **5a** in hand, we screened several cyclization conditions (Table 1). Noteworthy is that Dömling^19^ and Nadzan^20^ have reported the formation of 2-oxopiperazines by Ugi-N-acyliminium ion cyclization with good yield using HCOOH and TFA as acid components, respectively. To our surprise, TFA (entry 1), CH_3_COOH (entry 2), and 37% HCl(aq) solution (entry 5) in dioxane failed to give any 2-oxopiperazines product, while HCOOH afforded **6a** in 26% yield (entry 3). The combination of CH_3_COOH and conc. H_2_SO_4_ was found to induce an improvement of this cyclization, which afforded **6a** in 38% yield (entry 4). Disappointingly, methanesulfonic acid, which has been previously described as an appropriate acid for Ugi/Pictet–Spengler reactions, led to the formation of 65% **6a** and 31% **7a** when methanesulfonic acid in acetonitrile (v/v = 1:3) was used (entry 13). However, too much methanesulfonic acid turned out to be harmful for **6a** formation, which only afforded **6a** in 15% yield in two steps (entry 14). The reaction yield increased to 91% over two steps when the methanesulfonic acid ratio decreased and the time was increased to 12 h (entry 11). Interestingly, when the intermediate compound **5a** was treated with a high concentration of methanesulfonic acid for 12 h, polycyclic compound **7a** was formed with an excellent yield of 90% (entry 15). Unfortunately, the intermediate compound **5a** with solventless methanesulfonic acid only formed polycyclic compound **7a** at a good yield of 81%. To our delight, this reaction shows excellent stereoselectivity; only a single spatial configuration was formed in the products **6a** and **7a**.

**Table 1:**
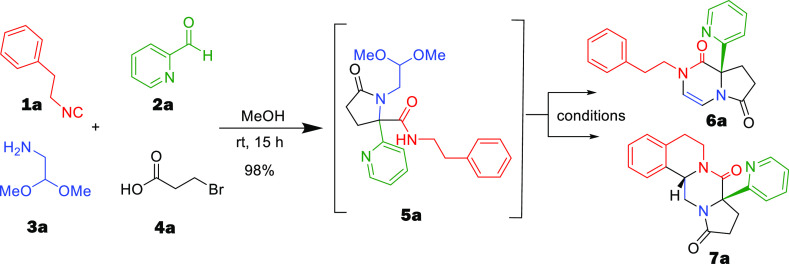

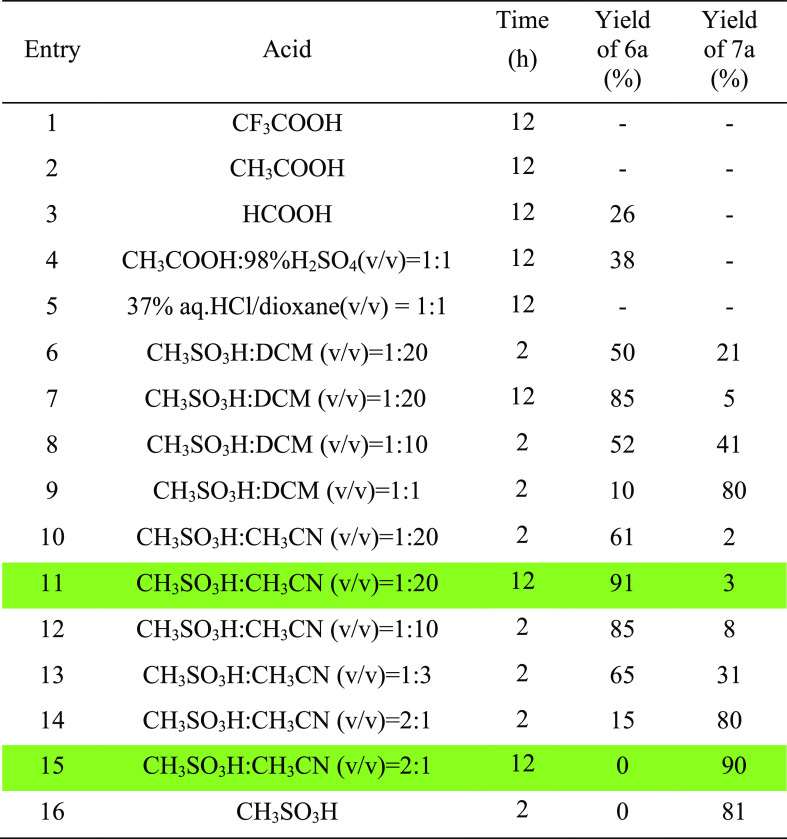
Optimization of Reaction Conditions^a^

a Reaction conditions: **5a** (0.2 mmol), 25 °C. The green color indicates the best conditions screened for **6a** and **7a**.

**Scheme 1:**
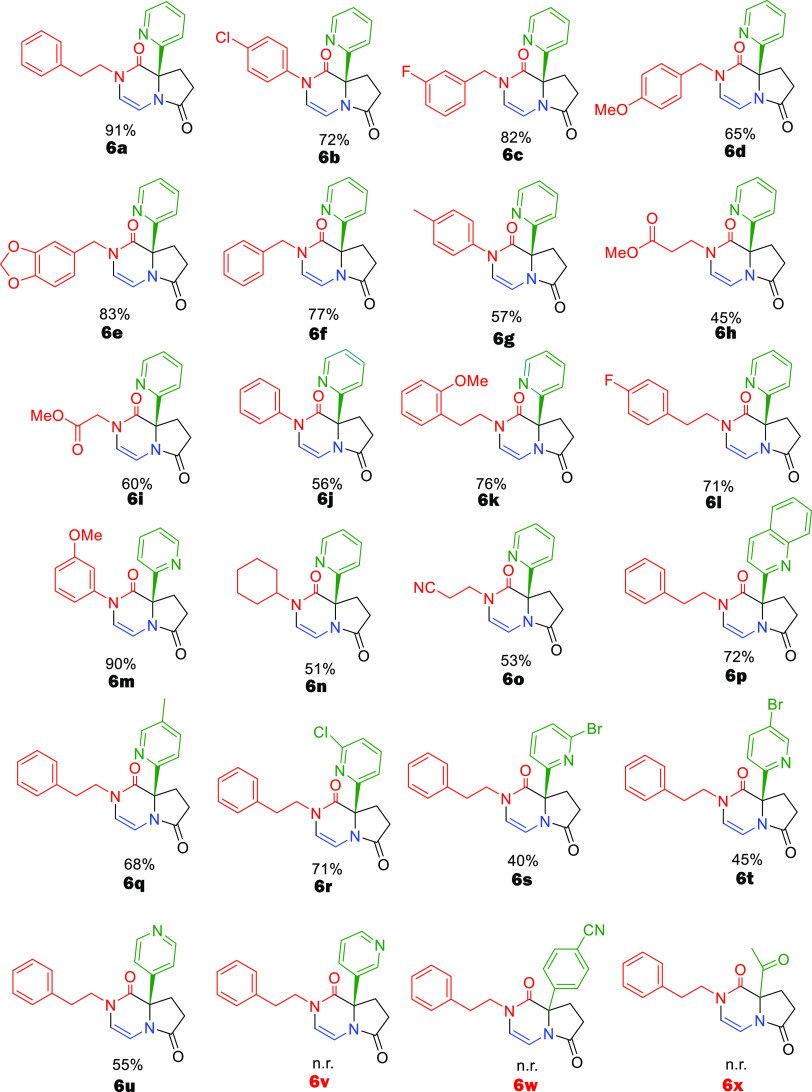
Synthesis of Heterocycles by Different Isocyanides and Aldehydes

With these optimized conditions in hand, we went on to examine the substrate scope of the reaction. We first reacted aldehyde **2**, carboxylic acid **3**, and bifunctional amine **4** with different isocyanides **1** in methanol at room temperature for 15 h (Scheme 1). The Ugi 4-CR proceeded well, and the desired product **5** was isolated in good yield (Supporting Infromation). To our delight, the subsequent Pictet–Spengler reaction of **5** went smoothly, and the desired product **6** was isolated in good yield from 45 to 91%; both aromatic and aliphatic isocyanides work well in this reaction (Scheme 1).

After successfully demonstrating that different isocyanides work well, we then focused on screening different aldehydes. Pyridine aldehyde was utilized in several cases and results in good yield, like picolinaldehyde (**6a**) and isonicotinaldehyde (**6u**) with 91 and 55% yields, respectively, while nicotinaldehyde did not work. Pyridine aldehyde with electron-donating and -withdrawing groups such as 5-Me,5-Br, 6-Cl, and 6-Br also reacted smoothly giving 68, 45, 71, and 40% yields, respectively. When quinoline-2-carbaldehyde was applied, it gave the corresponding products in a good yield of 72%. To our great surprise, utilizing aldehydes rather than pyridine-derived ones, neither compound **5** nor **6v** was formed, and we could not observe the compounds **6w** and **6x** (Scheme 1).

**Scheme 2:**
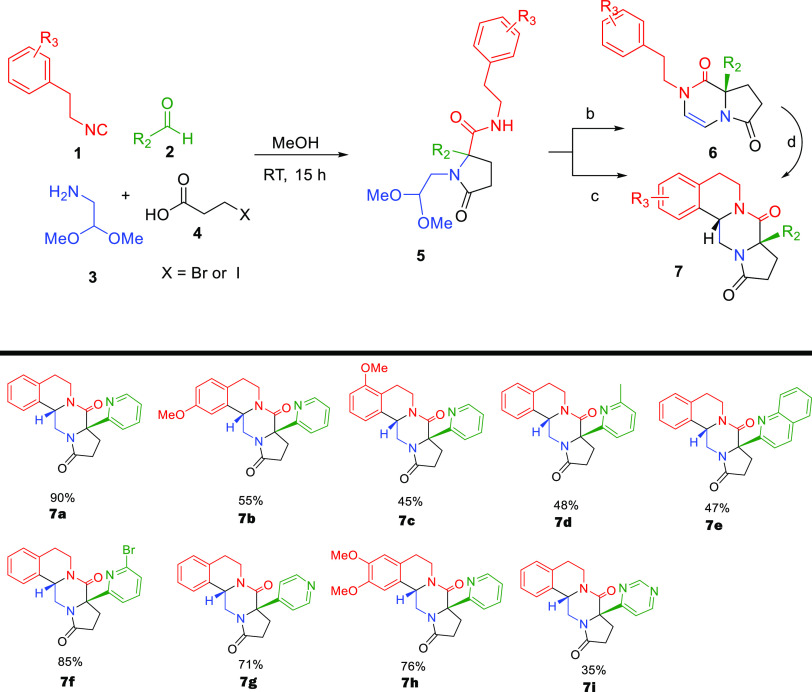
Structures of Polyheterocycles^,^^,^^,^ The isocyanide, aldehyde, amine, and carboxylic acid components are depicted in red, green, blue, and black, respectively. The Ugi reaction conditions are as follows: **1** (1.0 mmol), **2** (1.0 mmol), **3** (1.0 mmol), **4** (1.0 mmol), MeOH (1.0 mL), 15 h, and 25 °C. Reaction conditions: **5** (0.5 mmol), CH_3_SO_3_H (0.1 mL), CH_3_CN (2.0 mL), 25 °C, and 12 h. Reaction conditions: **5** (0.5 mmol), CH_3_SO_3_H (1.0 mL), CH_3_CN (0.5 mL), 25 °C, 12 h. or HCOOH (2.0 mL), 25 °C, and 4 h. Reaction conditions: CH_3_SO_3_H (2.0 mL), 70 °C, and 1 h.

A potential mechanistic explanation is given below. Next, we investigated the Pictet–Spengler cyclization of suitable **6** derived from (substituted) phenylethyl isocyanides to yield tetracyclic **7**. High-concentration methanesulfonic acid was found to be good for monomethoxy phenylethyl or phenylethyl Ugi products, yielding 35–90% of **7** (Scheme 2). However, more electron-rich dimethoxyphenyl Ugi product **5** was too reactive; using methanesulfonic acid and formic acid was found superior for the cyclization to at of polyheterocycle **7h**.

**Figure 2:**
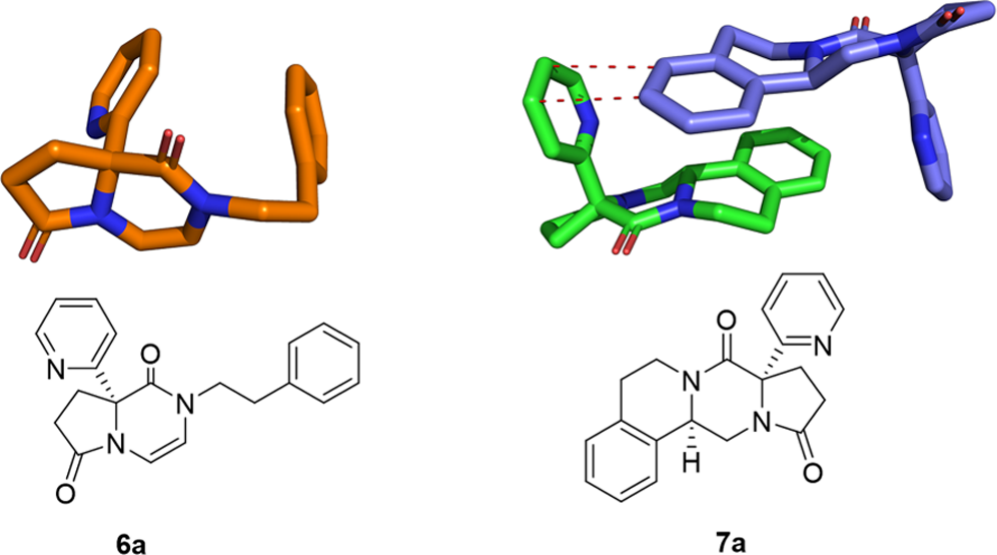
X-ray structures of the selected products CCDC 2089898 (**6a**) and CCDC 2089897 (**7a**) shown as sticks rendered using PyMol. Short intermolecular contacts (3.7 Å) in **7a** are shown as red dotted lines.

**Scheme 3:**
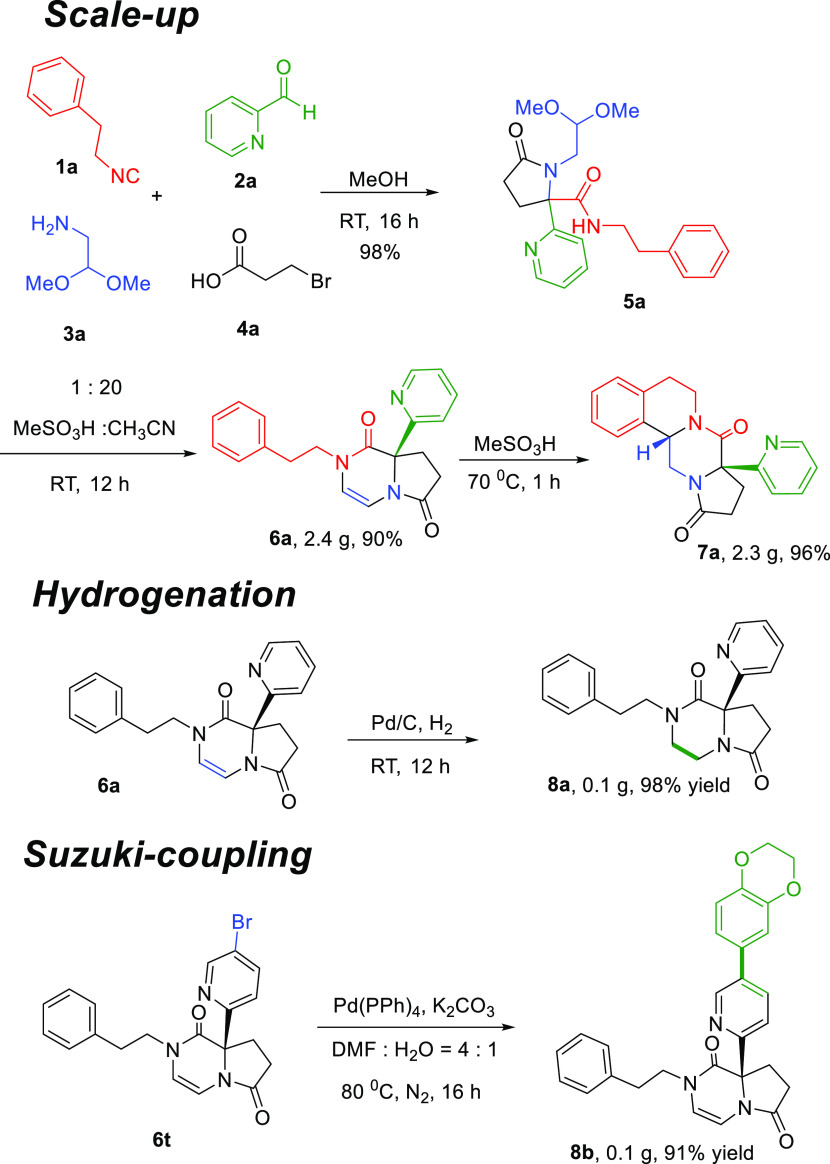
Further Synthetic Applications

The structures of **6a** and **7a** were confirmed by X-ray crystallography (Figure 2). Noteworthy is the pyridyl residue exit orthogonal to the bicyclic and tricyclic flat ring systems. The pyridyl ring of **7a** forms a short T-shaped stacking interaction with the phenyl group of a neighboring molecule, thus forming a dimer in the crystal. Next, the scalability of this method was investigated (Scheme 3). A four-component reaction of (2-isocyanoethyl)benzene, picolinaldehyde, 2,2-dimethoxyethan-1-amine, and 3-bromopropanoic acid was conducted on a 8 mmol scale, which upon further treatment with methanesulfonic acid conditions produced **6a** and polyheterocyclic product **7a** in 90% (2.4 g) and 89% (2.3 g) yields, respectively.

To further underscore the usefulness of the herein-described 8*a*-(pyridin-2-yl)-8,8*a*-dihydropyrrolo [1,2-a]pyrazine-1,6(2*H*,7*H*)-diones, we performed several late-stage functionalizations. In compound **6a**, we reduced the double bond with Pd/C to give the product **8a** in a quantitative yield (Scheme 3). In another application, the bromo group of **6t** was coupled with 1,4-benzodioxane-6-boronic acid to give the derivative **8b** (Scheme 3). C–C coupling reactions such as the Suzuki reaction are highly preferred “privileged” reactions in drug discovery.^21^

**Scheme 4:**
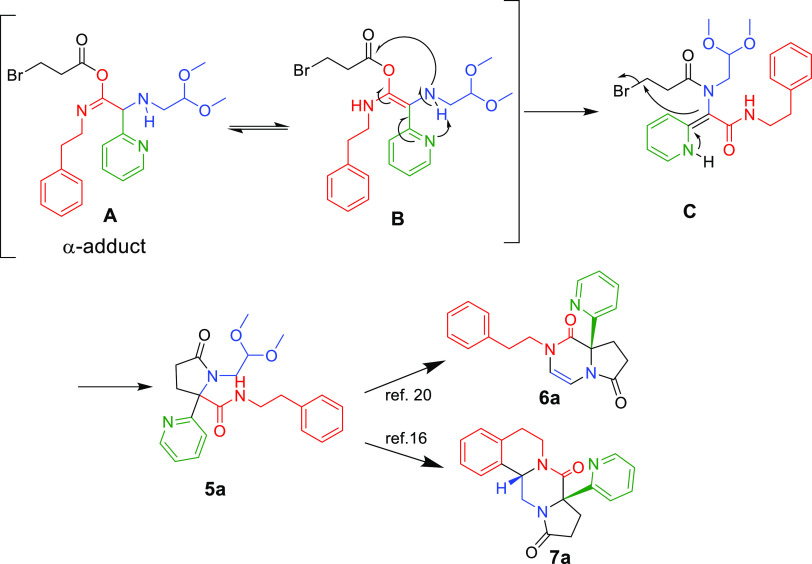
Proposed Reaction Mechanism

The findings of the high substrate specificity of the overall sequence, working with pyridine and pyrimidine derivatives, is unusual and deserves some mechanistic discussion. A hypothesized mechanism of this tandem reaction is shown in Scheme 4.

The Ugi α-adduct intermediate **A** forms under standard conditions, and a pyridine side chain-induced resonance structure **B** is conceivable. Supported by the vicinal pyridine, the Ugi product (**C**) formed through the Mumm rearrangement. The Ugi product **(C**) with a double bond cyclizes through an intramolecular substitution to yield the stable compound **5a** (derivative **5x** crystal, see the Supporting Infromation). The Ugi-cyclized product **5a** when treated under strongly acidic conditions further cyclizes as previously described by Nadzan^20^ and Dömling^16^ toward the target product **6a** and **7a**, respectively. The exclusive formation of the syn-diastereomers **7a** can be explained by the less-hindered reaction of the phenylethyl moiety anti to the pyridine. The mechanism involving a nucleophilic substitution differentiates the synthesis from previous ones and explains why only 2- and 4-pyridyl but not 3-pyridyl carbaldehyde or benzaldehyde used in the Ugi reaction can undergo further cyclization. Using MeOD and CD_3_CN as the solvent to perform certain control experiments, all the NMR data show that nothing changes.

## Conclusions

In summary, we have developed a straightforward method to assemble rigid nitrogen (N)-containing polyheterocycles. The Ugi postcyclization strategy is probably the most powerful tool in MCR chemistry to create structural diversity and large compound numbers while keeping the number of synthetic steps low. It has already attracted much attention from medicinal chemists in the field of drug discovery. Our new Ugi/Pictet–Spengler strategy is an expedited and convergent access to skeletally diverse compounds. Significantly, N-heterocycles and the novel fused tricyclic compound can now be constructed in just two steps with this method. We have also shown scalability of our method to gram-scale and several “late-stage” modifications. The process uses readily available starting materials and is simple to operate, provides a diverse compound library, and will therefore become a synthetical useful addition to the method arsenal in organic synthesis and medicinal chemistry.

## Experimental Section

### General Remarks

All chemicals (aldehydes **2**, 2,2-dimethoxyethan-1-amine **3**, 3-bromopropanoic acid **4**, and amines) were purchased from commercial suppliers and used without any purification unless otherwise noted. All isocyanides **1** were prepared according to the procedures reported from our lab, and all data are given in the Supporting Infromation.^11,16,22^ Nuclear magnetic resonance spectra were recorded on a Brucker 500 MHz. Chemical shifts for ^1^H NMR were reported as δ values, and coupling constants were reported in hertz (Hz). The following abbreviations were used for spin multiplicity: s = singlet, bs = broad singlet, d = doublet, t = triplet, q = quartet, quin = quintet, dd = double of doublets, ddd = double of doublet of doublets, and m = multiplet. Chemical shifts for ^13^C NMR were reported in ppm relative to the solvent peak. Thin-layer chromatography was performed on silica gel plates (0.20 mm thickness, particle size 25 μm). Flash chromatography was performed using RediSep R_f_ normal-phase silica flash columns (Silica Gel 60 Å, 230–400 mesh). High-resolution mass spectra were recorded using a LTQOrbitrap-XL (Thermo) at a resolution of 60000@*m*/*z*400. Melting points were obtained on a melting point apparatus and were uncorrected. Yields given refer to chromatographically purified and spectroscopically pure compounds unless otherwise stated.

#### General Experimental Procedure and Characterization Procedure A

General procedure for heterocycle **6**: A solution of isocyanide **1** (1 mmol, 1.0 equiv), aldehyde **2** (1 mmol, 1.0 equiv), amine **3** (1 mmol, 1.0 equiv), and acid **4** (1 mmol, 1.0 equiv) in methanol (1 mL) was stirred at room temperature for 15 h. The solvents were removed under vacuum. Then, the crude Ugi-adduct **5** was dissolved in 4 mL of acetonitrile, and 0.2 mL of methanesulfonic acid was added. The resulting mixture was stirred at room temperature for 12 h. The reaction was diluted with dichloromethane and quenched with saturated sodium bicarbonate solution at 0–5 °C. The resulting solution was extracted with dichloromethane (10 mL × 3). The solvents were removed under vacuum, and the crude product was purified by flash column chromatography to give pure product **6**.

#### Procedure B

General procedure for polyheterocycle **7**: A solution of isocyanide **1** (1 mmol, 1.0 equiv), aldehyde **2** (1 mmol, 1.0 equiv), amine **3** (1 mmol, 1.0 equiv), and acid **4** (1 mmol, 1.0 equiv) in methanol (1 mL) was stirred at room temperature for 15 h. The solvents were removed under vacuum. Then, the crude Ugi-adduct **5** was dissolved in 1 mL of acetonitrile, and 2 mL of methanesulfonic acid was added. The resulting mixture was stirred at 25 °C for 12 h. The reaction was diluted with dichloromethane and quenched with saturated sodium bicarbonate solution at 0–5 °C. The resulting solution was extracted with dichloromethane (10 mL × 3). The solvents were removed under vacuum, and the crude product was purified by flash column chromatography to give pure product **7**.

#### Procedure C

General procedure for polyheterocycle **7h**: A solution of isocyanide **1** (1 mmol, 1.0 equiv), aldehyde **2** (1 mmol, 1.0 equiv), amine **3** (1 mmol, 1.0 equiv), and acid **4** (1 mmol, 1.0 equiv) in methanol (1 mL) was stirred at room temperature for 15 h. The solvents were removed under vacuum. Then, the crude Ugi-adduct **5** was dissolved in 3.0 mL of HCOOH. The resulting mixture was stirred at room temperature for 4 h. The solvents were removed under vacuum; then, the crude product was diluted with dichloromethane and quenched with saturated sodium bicarbonate solution at 0–5 °C. The resulting solution was extracted with dichloromethane (10 mL × 3). The solvents were removed under vacuum, and the crude product was purified by flash column chromatography to give pure product **7h**.

#### Procedure D

General procedure for heterocycle **6** transformation to polyheterocycle **7**: A solution of heterocycle **6** (1 mmol, 1.0 equiv) in 3.0 mL of methanesulfonic acid was stirred at 70 °C for 1 h. The reaction was diluted with dichloromethane and quenched with saturated sodium bicarbonate solution at 0–5 °C. The resulting solution was extracted with dichloromethane (10 mL × 3). The solvents were removed under vacuum, and the crude product was purified by flash column chromatography to give pure product **7**.

#### Gram-Scale synthesis of **6a** and **7a**

A solution of isocyanide **1a** (8 mmol, 1.0 equiv), aldehyde **2a** (8.0 mmol, 1.0 equiv), amine **3a** (8.0 mmol, 1.0 equiv), and acid **4a** (8.0 mmol, 1.0 equiv) in methanol (8 mL) was stirred at room temperature for 15 h. The solvents were removed under vacuum. Then, the crude Ugi-adduct **5a** was dissolved in 20 mL of acetonitrile, and 1.0 mL of methanesulfonic acid was added. The resulting mixture was stirred at room temperature for 12 h. The reaction was diluted with dichloromethane and quenched with saturated sodium bicarbonate solution at 0–5 °C. The resulting solution was extracted with dichloromethane (80 mL × 3). The solvents were removed under vacuum, and the crude product was purified by flash column chromatography to give pure product **6a** (2.4 g, 90%). Then, a solution of heterocycle **6a** (2.4 g, 7.2 mmol, 1.0 equiv) in 10 mL of methanesulfonic acid was stirred at 70 °C for 1 h. The reaction mixture was diluted with dichloromethane and quenched with saturated sodium bicarbonate solution at 0–5 °C. The resulting solution was extracted with dichloromethane (100 mL × 3). The solvents were removed under vacuum, and the crude product was purified by flash column chromatography to give pure product **7a** (2.3 g, 96%).

#### Procedure E

Synthesis of product **8a**: Compound **6a** (100 mg, 0.3 mmol, 1.0 equiv) was dissolved in 5.0 mL of THF, Pd/C (6.4 mg, 0.06 mmol, 0.2 equiv) was added under a N_2_ atmosphere; the reaction mixture was stirred under a H_2_ atmosphere for 12 h. The reaction mixture was filtered, and the filtrate was removed under vacuum and purified by flash column chromatography to give pure product **8a** (0.1 g, 98%).

#### Procedure F

Synthesis of product **8b**: A solution of compound **6t** (99 mg, 0.24 mmol, 1.0 equiv), 1,4-benzodioxane-6-boronic acid (52.0 mg, 0.29 mmol, 1.2 equiv), Pd(PPh_3_)_4_ (14 mg, 0.012 mmol, 0.05 equiv), and K_2_CO_3_ (100 mg, 0.72 mmol, 3.0 equiv) in DMF/H_2_O (8/2 mL) was stirred at 80 °C under a N_2_ atmosphere for 16 h. After cooling, 10 mL of water was added to dilute the reaction mixture. The reaction mixture was extracted with EtOAc. The combined organic layers were washed with water and brine and dried over anhydrous Na_2_SO_4_. After removal of the EtOAc, the residue was purified by column chromatography (silica gel, methanol/dichloromethane = 5:95) to afford the product **8b** (0.1 g, 91%).

##### (*R*)-2-Phenethyl-8*a*-(pyridin-2-yl)-8,8*a*-dihydropyrrolo[1,2-*a*]pyrazine-1,6(2*H*,7*H*)-dione (**6a**)

Synthesis according to procedure **A** in the 1 mmol scale and purification by column chromatography (silica gel, petroleum ether/ethyl acetate = 1:3) afforded **6a** (303 mg, 91%) as a yellow solid; ^1^H NMR (500 MHz, chloroform-*d*) δ 8.62 (ddd, *J* = 4.7, 1.9, 1.1 Hz, 1H), 7.67 (td, *J* = 7.8, 1.8 Hz, 1H), 7.26 (ddd, *J* = 7.6, 4.8, 1.3 Hz, 1H), 7.23–7.17 (m, 4H), 7.08–7.04 (m, 2H), 6.49 (d, *J* = 5.7 Hz, 1H), 5.49 (d, *J* = 5.7 Hz, 1H), 3.90 (ddd, *J* = 14.0, 7.9, 6.3 Hz, 1H), 3.74 (dt, *J* = 13.4, 7.4 Hz, 1H), 2.93–2.82 (m, 2H), 2.76 (ddd, *J* = 12.5, 11.0, 9.5 Hz, 1H), 2.63 (ddd, *J* = 17.3, 11.0, 8.7 Hz, 1H), 2.55–2.43 (m, 2H). ^13^C{^1^H} NMR (126 MHz, chloroform-*d*) δ 173.5, 165.2, 157.6, 149.8, 137.9, 137.0, 128.9, 128.5, 126.6, 123.2, 119.7, 116.8, 106.7, 68.8, 48.3, 34.6, 30.7, 29.7. HRMS (ESI) *m*/*z* calculated for C_20_H_20_N_3_O_2_ [M + H]^+^: 334.1530; found [M + H]^+^: 334.1531.

##### (*R*)-2-(4-Chlorophenyl)-8*a*-(pyridin-2-yl)-8,8*a*-dihydropyrrolo[1,2-*a*]pyrazine-1,6(2*H*,7*H*)-dione (**6b**)

Synthesis according to procedure **A** in the 1 mmol scale and purification by column chromatography (silica gel, petroleum ether/ethyl acetate = 1:3) afforded **6b** (245 mg, 72%) as a brown solid. ^1^H NMR (500 MHz, chloroform-*d*) δ 8.67 (d, *J* = 4.7 Hz, 1H), 7.80–7.72 (m, 1H), 7.45–7.36 (m, 3H), 7.32 (dd, *J* = 7.5, 4.8 Hz, 1H), 7.28–7.17 (m, 2H), 6.67 (d, *J* = 5.5 Hz, 1H), 5.87 (d, *J* = 5.7 Hz, 1H), 2.89 (dt, *J* = 13.2, 10.4 Hz, 1H), 2.71 (ddd, *J* = 17.3, 11.0, 8.9 Hz, 1H), 2.58 (dd, *J* = 17.4, 9.9 Hz, 1H), 2.49 (dd, *J* = 13.3, 8.9 Hz, 1H). ^13^C{^1^H} NMR (126 MHz, chloroform-*d*) δ 173.3, 165.0, 157.7, 150.0, 138.1, 137.3, 133.4, 129.4, 127.2, 123.5, 119.4, 117.2, 107.5, 69.2, 30.1, 29.6. HRMS (ESI) *m*/*z* calculated for C_18_H_15_ClN_3_O_2_ [M + H]^+^: 340.0821; found [M + H]^+^: 340.0827.

##### (*R*)-2-(3-Fluorobenzyl)-8*a*-(pyridin-2-yl)-8,8*a*-dihydropyrrolo[1,2-*a*]pyrazine-1,6(2*H*,7*H*)-dione (**6c**)

Synthesis according to procedure **A** in the 1 mmol scale and purification by column chromatography (silica gel, petroleum ether/ethyl acetate = 1:3) afforded **6c** (276 mg, 82%) as a brown solid. ^1^H NMR (500 MHz, chloroform-*d*) δ 8.63 (d, *J* = 5.2 Hz, 1H), 7.70 (t, *J* = 7.8 Hz, 1H), 7.26 (dd, *J* = 13.2, 8.3 Hz, 3H), 6.96 (d, *J* = 8.7 Hz, 2H), 6.88 (d, *J* = 9.8 Hz, 1H), 6.57 (d, *J* = 5.7 Hz, 1H), 5.64 (d, *J* = 5.7 Hz, 1H), 4.92 (d, *J* = 15.1 Hz, 1H), 4.63 (d, *J* = 15.1 Hz, 1H), 2.92–2.80 (m, 1H), 2.72–2.59 (m, 1H), 2.59–2.48 (m, 2H). ^13^C{^1^H} NMR (126 MHz, chloroform-*d*) δ 173.4, 165.5, 164.0, 162.0, 157.6, 149.8, 138.6, 138.5, 137.0, 130.3, 130.2, 123.4, 123.3, 123.3, 119.6, 115.9, 114.9, 114.8, 114.6, 107.4, 68.9, 49.0, 30.4, 29.6. HRMS (ESI) *m*/*z* calculated for C_19_H_17_FN_3_O_2_ [M + H]^+^: 338.1235; found [M + H]^+^: 338.1238.

##### (*R*)-2-(4-Methoxybenzyl)-8*a*-(pyridin-2-yl)-8,8*a*-dihydropyrrolo[1,2-*a*]pyrazine-1,6(2*H*,7*H*)-dione (**6d**)

Synthesis according to procedure **A** in the 1 mmol scale and purification by column chromatography (silica gel, petroleum ether/ethyl acetate = 1:3) afforded **6d** (227 mg, 65%) as a brown solid. ^1^H NMR (500 MHz, chloroform-*d*) δ 8.61 (d, *J* = 5.5 Hz, 1H), 7.66 (td, *J* = 7.8, 1.9 Hz, 1H), 7.25 (dd, *J* = 7.6, 4.2 Hz, 2H), 7.13 (d, *J* = 8.7 Hz, 2H), 6.83 (d, *J* = 8.7 Hz, 2H), 6.53 (d, *J* = 5.7 Hz, 1H), 5.65 (d, *J* = 5.7 Hz, 1H), 4.82 (d, *J* = 14.7 Hz, 1H), 4.61 (d, *J* = 14.7 Hz, 1H), 3.80 (s, 3H), 2.88–2.77 (m, 1H), 2.75–2.65 (m, 1H), 2.56–2.47 (m, 2H). ^13^C{^1^H} NMR (126 MHz, chloroform-*d*) δ 173.5, 165.4, 159.3, 157.7, 149.8, 136.9, 129.4, 128.1, 123.3, 119.6, 116.0, 114.1, 107.1, 68.8, 55.3, 49.1, 30.5, 29.7. HRMS (ESI) *m*/*z* calculated for C_20_H_20_N_3_O_3_ [M + H]^+^: 350.1452; found [M + H]^+^: 350.1457.

##### (*R*)-2-(Benzo[*d*][1,3]dioxol-5-ylmethyl)-8*a*-(pyridin-2-yl)-8,8*a*-dihydropyrrolo[1,2-*a*]pyrazine-1,6(2*H*,7*H*)-dione (**6e**)

Synthesis according to procedure **A** in the 1 mmol scale and purification by column chromatography (silica gel, petroleum ether/ethyl acetate = 1:3) afforded **6e** (301 mg, 83%) as a yellow solid. ^1^H NMR (500 MHz, chloroform-*d*) δ 8.60 (d, *J* = 4.7 Hz, 1H), 7.67 (td, *J* = 7.8, 1.8 Hz, 1H), 7.28–7.23 (m, 2H), 6.71 (d, *J* = 7.9 Hz, 1H), 6.69–6.62 (m, 2H), 6.52 (d, *J* = 5.7 Hz, 1H), 5.94–5.90 (m, 2H), 5.64 (d, *J* = 5.7 Hz, 1H), 4.77 (d, *J* = 14.7 Hz, 1H), 4.56 (d, *J* = 14.7 Hz, 1H), 2.82 (ddd, *J* = 12.8, 11.0, 9.5 Hz, 1H), 2.65 (ddd, *J* = 17.5, 11.0, 9.0 Hz, 1H), 2.56–2.45 (m, 2H). ^13^C{^1^H} NMR (126 MHz, chloroform-*d*) δ 173.4, 165.4, 157.7, 149.7, 148.0, 147.4, 137.0, 129.8, 123.4, 121.5, 119.6, 115.9, 108.4, 108.3, 107.1, 101.2, 68.8, 49.3, 30.5, 29.7. HRMS (ESI) *m*/*z* calculated for C_20_H_18_N_3_O_4_ [M + H]^+^: 364.1248; found [M + H]^+^: 364.1249.

##### (*R*)-2-Benzyl-8*a*-(pyridin-2-yl)-8,8*a*-dihydropyrrolo[1,2-*a*]pyrazine-1,6(2*H*,7*H*)-dione (**6f**)

Synthesis according to procedure **A** in the 1 mmol scale and purification by column chromatography (silica gel, petroleum ether/ethyl acetate = 1:3) afforded **6f** (246 mg, 77%) as a yellow solid. ^1^H NMR (500 MHz, chloroform-*d*) δ 8.62 (dt, *J* = 5.2, 1.6 Hz, 1H), 7.67 (td, *J* = 7.7, 1.9 Hz, 1H), 7.38–7.23 (m, 5H), 7.17 (dd, *J* = 7.5, 2.1 Hz, 2H), 6.54 (d, *J* = 5.7 Hz, 1H), 5.66 (d, *J* = 5.7 Hz, 1H), 4.89 (d, *J* = 14.8 Hz, 1H), 4.69 (d, *J* = 14.8 Hz, 1H), 2.90–2.77 (m, 1H), 2.67 (ddd, *J* = 17.7, 11.0, 8.9 Hz, 1H), 2.58–2.48 (m, 2H). ^13^C{^1^H} NMR (126 MHz, chloroform-*d*) δ 173.5, 165.4, 157.63, 149.8, 137.0, 136.0, 128.8, 127.9, 127.9, 123.4, 119.6, 116.1, 107.2, 68.9, 49.5, 30.6, 29.7. HRMS (ESI) *m*/*z* calculated for C_19_H_18_N_3_O_2_ [M + H]^+^: 320.1357; found [M + H]^+^: 320.1359.

##### (*R*)-8*a*-(Pyridin-2-yl)-2-(p-tolyl)-8,8*a*-dihydropyrrolo[1,2-*a*]pyrazine-1,6(2*H*,7*H*)-dione (**6g**)

Synthesis according to procedure **A** in the 1 mmol scale and purification by column chromatography (silica gel, petroleum ether/ethyl acetate = 1:3) afforded **6g** (182 mg, 57%) as a yellow solid. ^1^H NMR (500 MHz, chloroform-*d*) δ 8.66 (dt, *J* = 4.7, 1.4 Hz, 1H), 7.75 (td, *J* = 7.7, 1.8 Hz, 1H), 7.42 (dt, *J* = 7.9, 1.2 Hz, 1H), 7.32–7.29 (m, 1H), 7.23 (d, *J* = 8.4 Hz, 2H), 7.17 (d, *J* = 8.5 Hz, 2H), 6.63 (d, *J* = 5.5 Hz, 1H), 5.88 (d, *J* = 5.7 Hz, 1H), 2.88 (ddd, *J* = 13.1, 11.0, 9.7 Hz, 1H), 2.72 (ddd, *J* = 17.3, 11.0, 8.9 Hz, 1H), 2.61–2.43 (m, 2H), 2.38 (s, 3H). ^13^C{^1^H} NMR (126 MHz, chloroform-*d*) δ 173.4, 165.1, 157.9, 149.9, 137.7, 137.1, 137.1, 129.9, 125.7, 123.4, 119.5, 117.9, 107.0, 69.2, 30.3, 29.7, 21.1. HRMS (ESI) *m*/*z* calculated for C_19_H_18_N_3_O_2_ [M + H]^+^: 320.1355; found [M + H]^+^: 320.1358.

##### Methyl (*R*)-3-(1,6-Dioxo-8*a*-(pyridin-2-yl)-6,7,8,8*a*-tetrahydropyrrolo[1,2-*a*]pyrazin-2(1H)-yl)propanoate (**6h**)

Synthesis according to procedure **A** in the 1 mmol scale and purification by column chromatography (silica gel, petroleum ether/ethyl acetate = 1:3) afforded **6h** (142 mg, 45%) as yellow oil. ^1^H NMR (500 MHz, chloroform-*d*) δ 8.84–8.24 (m, 1H), 7.68 (td, *J* = 7.8, 1.8 Hz, 1H), 7.28–7.20 (m, 2H), 6.50 (s, 1H), 5.77 (d, *J* = 5.7 Hz, 1H), 3.88–3.83 (m, 2H), 3.62 (s, 3H), 2.85–2.69 (m, 2H), 2.71–2.57 (m, 2H), 2.59–2.42 (m, 2H). ^13^C{^1^H} NMR (126 MHz, chloroform-*d*) δ 173.4, 171.7, 165.3, 157.7, 149.7, 137.0, 123.3, 119.5, 117.3, 106.7, 68.7, 51.8, 43.3, 32.8, 30.2, 29.6. HRMS (ESI) *m*/*z* calculated for C_16_H_18_N_3_O_4_ [M + H]^+^: 316.1252; found [M + H]^+^: 316.1256.

##### Methyl (*R*)-2-(1,6-Dioxo-8*a*-(pyridin-2-yl)-6,7,8,8*a*-tetrahydropyrrolo[1,2-*a*]pyrazin-2(1H)-yl)acetate (**6i**)

Synthesis according to procedure **A** in the 1 mmol scale and purification by column chromatography (silica gel, petroleum ether/ethyl acetate = 1:3) afforded **6i** (181 mg, 60%) as yellow oil. ^1^H NMR (500 MHz, chloroform-*d*) δ 8.60 (ddd, *J* = 4.9, 1.9, 1.0 Hz, 1H), 7.70 (td, *J* = 7.7, 1.8 Hz, 1H), 7.38 (dt, *J* = 8.0, 1.1 Hz, 1H), 7.25 (ddd, *J* = 7.6, 4.8, 1.2 Hz, 1H), 6.57 (d, *J* = 5.5 Hz, 1H), 5.64 (d, *J* = 5.7 Hz, 1H), 4.40 (d, *J* = 17.3 Hz, 1H), 4.24 (d, *J* = 17.2 Hz, 1H), 3.75 (s, 3H), 2.81–2.67 (m, 2H), 2.57–2.47 (m, 2H). ^13^C{^1^H} NMR (126 MHz, chloroform-*d*) δ 173.5, 168.2, 165.9, 157.2, 149.8, 136.9, 123.4, 120.0, 116.4, 107.5, 68.7, 52.5, 47.5, 30.6, 29.8. HRMS (ESI) *m*/*z* calculated for C_15_H_16_N_3_O_4_ [M + H]^+^: 302.1126; found [M + H]^+^: 302.1129.

##### (*R*)-2-Phenyl-8*a*-(pyridin-2-yl)-8,8*a*-dihydropyrrolo[1,2-*a*]pyrazine-1,6(2*H*,7*H*)-dione (**6j**)

Synthesis according to procedure **A** in the 1 mmol scale and purification by column chromatography (silica gel, petroleum ether/ethyl acetate = 1:3) afforded **6j** (171 mg, 56%) as a yellow solid. ^1^H NMR (500 MHz, chloroform-*d*) δ 8.67 (dt, *J* = 4.7, 1.3 Hz, 1H), 7.76 (d, *J* = 1.9 Hz, 1H), 7.44 (td, *J* = 7.6, 2.2 Hz, 3H), 7.38–7.27 (m, 4H), 6.65 (d, *J* = 5.7 Hz, 1H), 5.91 (d, *J* = 5.7 Hz, 1H), 2.95–2.84 (m, 1H), 2.73 (ddd, *J* = 17.3, 11.0, 8.9 Hz, 1H), 2.58 (ddd, *J* = 17.3, 9.8, 1.7 Hz, 1H), 2.50 (dd, *J* = 13.2, 7.3 Hz, 1H). ^13^C{^1^H} NMR (126 MHz, chloroform-*d*) δ 173.4, 165.0, 157.8, 149.9, 139.6, 137.2, 129.3, 127.7, 125.9, 123.4, 119.5, 117.7, 107.2, 69.2, 30.2, 29.7. HRMS (ESI) *m*/*z* calculated for C_18_H_16_N_3_O_2_ [M + H]^+^: 306.1265; found [M + H]^+^: 306.1267.

##### (*R*)-2-(2-Methoxyphenethyl)-8*a*-(pyridin-2-yl)-8,8*a*-dihydropyrrolo[1,2-*a*]pyrazine-1,6(2*H*,7*H*)-dione (**6k**)

Synthesis according to procedure **A** in the 1 mmol scale and purification by column chromatography (silica gel, petroleum ether/ethyl acetate = 1:3) afforded **6k** (276 mg, 76%) as a yellow solid. ^1^H NMR (500 MHz, chloroform-*d*) δ 8.62–8.60 (m, 1H), 7.66 (td, *J* = 7.7, 1.8 Hz, 1H), 7.27–7.17 (m, 3H), 6.91 (dd, *J* = 7.3, 1.8 Hz, 1H), 6.83 (d, *J* = 7.1 Hz, 1H), 6.77 (td, *J* = 7.4, 1.1 Hz, 1H), 6.46 (d, *J* = 5.7 Hz, 1H), 5.52 (d, *J* = 5.7 Hz, 1H), 3.85 (t, *J* = 6.4 Hz, 1H), 3.82 (s, 3H), 3.82–3.78 (m, 1H), 3.01–2.94 (m, 1H), 2.84–2.77 (m, 1H), 2.76–2.70 (m, 1H), 2.68–2.58 (m, 1H), 2.53–2.44 (m, 2H). ^13^C{^1^H} NMR (126 MHz, chloroform-*d*) δ 173.5, 165.3, 157.7, 157.6, 149.7, 136.9, 130.7, 128.0, 126.2, 123.1, 120.4, 119.7, 117.0, 110.2, 106.5, 68.8, 55.3, 46.6, 30.8, 29.7, 29.6. HRMS (ESI) *m*/*z* calculated for C_21_H_22_N_3_O_3_ [M + H]^+^: 364.1624; found [M + H]^+^: 364.1627.

##### (*R*)-2-(4-Fluorophenethyl)-8*a*-(pyridin-2-yl)-8,8*a*-dihydropyrrolo[1,2-*a*]pyrazine-1,6(2*H*,7*H*)-dione (**6l**)

Synthesis according to procedure **A** in the 1 mmol scale and purification by column chromatography (silica gel, petroleum ether/ethyl acetate = 1:3) afforded **6l** (250 mg, 71%) as a yellow solid. ^1^H NMR (500 MHz, chloroform-*d*) δ 8.64–8.59 (m, 1H), 7.66 (td, *J* = 7.8, 1.8 Hz, 1H), 7.26 (dd, *J* = 7.6, 4.7 Hz, 1H), 7.18 (d, *J* = 7.9 Hz, 1H), 7.04–6.93 (m, 2H), 6.92–6.86 (m, 2H), 6.51 (d, *J* = 5.5 Hz, 1H), 5.49 (d, *J* = 5.7 Hz, 1H), 3.90 (ddd, *J* = 13.9, 7.7, 6.4 Hz, 1H), 3.75–3.64 (m, 1H), 2.90–2.79 (m, 2H), 2.78–2.70 (m, 1H), 2.60 (ddd, *J* = 17.7, 11.0, 8.9 Hz, 1H), 2.53–2.45 (m, 2H). ^13^C{^1^H} NMR (126 MHz, chloroform-*d*) δ 173.4, 165.2, 162.7, 160.7, 155.6, 149.8, 136.9, 133.5, 130.3, 123.2, 119.6, 116.4, 115.4, 115.2, 106.9, 68.8, 48.1, 33.7, 30.8, 29.6. HRMS (ESI) *m*/*z* calculated for C_20_H_19_FN_3_O_2_ [M + H]^+^: 352.1471; found [M + H]^+^: 352.1473.

##### (*R*)-2-(3-Methoxyphenyl)-8*a*-(pyridin-2-yl)-8,8*a*-dihydropyrrolo[1,2-*a*]pyrazine-1,6(2*H*,7*H*)-dione (**6m**)

Synthesis according to procedure **A** in the 1 mmol scale and purification by column chromatography (silica gel, petroleum ether/ethyl acetate = 1:3) afforded **6m** (354 mg, 90%) as a yellow solid. ^1^H NMR (500 MHz, chloroform-*d*) δ 8.66 (d, *J* = 6.6 Hz, 1H), 7.75 (td, *J* = 7.7, 1.8 Hz, 1H), 7.41 (d, *J* = 7.9 Hz, 1H), 7.35–7.31 (m, 1H), 7.31–7.29 (m, 1H), 6.89 (t, *J* = 2.2 Hz, 1H), 6.87 (t, *J* = 2.0 Hz, 1H), 6.85 (t, *J* = 2.2 Hz, 1H), 6.64 (d, *J* = 5.7 Hz, 1H), 5.90 (d, *J* = 5.5 Hz, 1H), 3.83 (s, 3H), 2.89 (ddd, *J* = 13.2, 11.0, 9.7 Hz, 1H), 2.72 (ddd, *J* = 17.2, 10.9, 8.8 Hz, 1H), 2.58 (ddd, *J* = 17.2, 9.8, 1.6 Hz, 1H), 2.49 (ddd, *J* = 13.2, 9.0, 1.7 Hz, 1H). ^13^C{^1^H} NMR (126 MHz, chloroform-*d*) δ 173.4, 165.0, 160.2, 157.8, 149.9, 140.7, 137.2, 130.0, 123.5, 119.5, 118.1, 117.7, 113.4, 111.9, 107.1, 69.2, 55.5, 30.2, 29.7. HRMS (ESI) *m*/*z* calculated for C_19_H_18_N_3_O_3_ [M + H]^+^: 336.1325; found [M + H]^+^: 336.1328.

##### (*R*)-2-Cyclohexyl-8*a*-(pyridin-2-yl)-8,8*a*-dihydropyrrolo[1,2-*a*]pyrazine-1,6(2*H*,7*H*)-dione (**6n**)

Synthesis according to procedure **A** in the 1 mmol scale and purification by column chromatography (silica gel, petroleum ether/ethyl acetate = 1:3) afforded **6n** (159 mg, 51%) as yellow oil. ^1^H NMR (500 MHz, chloroform-*d*) δ 8.61–8.57 (m, 1H), 7.67 (td, *J* = 7.7, 1.8 Hz, 1H), 7.28 (d, *J* = 6.8 Hz, 1H), 7.24 (ddd, *J* = 7.5, 4.8, 1.1 Hz, 1H), 6.54 (d, *J* = 5.8 Hz, 1H), 5.77 (d, *J* = 5.9 Hz, 1H), 4.52 (tt, *J* = 11.8, 3.8 Hz, 1H), 2.84–2.75 (m, 1H), 2.74–2.65 (m, 1H), 2.62–2.43 (m, 2H), 1.83 (d, *J* = 9.1 Hz, 2H), 1.69 (d, *J* = 6.4 Hz, 2H), 1.51–1.25 (m, 5H), 1.16–1.07 (m, 1H). ^13^C{^1^H} NMR (126 MHz, chloroform-*d*) δ 173.6, 165.1, 158.0, 149.7, 136.9, 123.20, 119.5, 112.6, 106.9, 68.8, 52.9, 31.6, 30.6, 30.6, 29.8, 25.7, 25.6, 25.3. HRMS (ESI) *m*/*z* calculated for C_18_H_22_N_3_O_2_ [M + H]^+^: 312.1634; found [M + H]^+^: 312.1638.

##### (*R*)-3-(1,6-Dioxo-8*a*-(pyridin-2-yl)-6,7,8,8*a*-tetrahydropyrrolo[1,2-*a*]pyrazin-2(1H)-yl)propanenitrile (**6o**)

Synthesis according to procedure **A** in the 1 mmol scale and purification by column chromatography (silica gel, petroleum ether/ethyl acetate = 1:3) afforded **6o** (150 mg, 53%) as brown oil. ^1^H NMR (500 MHz, chloroform-*d*) δ 8.59 (dd, *J* = 5.4, 1.9 Hz, 1H), 7.71 (td, *J* = 7.8, 1.8 Hz, 1H), 7.28–7.23 (m, 2H), 6.58 (d, *J* = 5.7 Hz, 1H), 5.76 (d, *J* = 5.5 Hz, 1H), 3.92–3.77 (m, 2H), 2.85–2.74 (m, 2H), 2.69–2.60 (m, 2H), 2.57–2.44 (m, 2H). ^13^C{^1^H} NMR (126 MHz, chloroform-*d*) δ 173.2, 165.5, 157.4, 149.8, 137.2, 123.5, 119.4, 117.1, 116.3, 107.6, 68.7, 43.3, 30.0, 29.5, 16.9. HRMS (ESI) *m*/*z* calculated for C_15_H_15_N_4_O_2_ [M + H]^+^: 283.1145; found [M + H]^+^: 283.1149.

##### (*R*)-2-Phenethyl-8*a*-(quinolin-2-yl)-8,8*a*-dihydropyrrolo[1,2-*a*]pyrazine-1,6(2*H*,7*H*)-dione (**6p**)

Synthesis according to procedure **A** in the 1 mmol scale and purification by column chromatography (silica gel, petroleum ether/ethyl acetate = 1:3) afforded **6p** (276 mg, 72%) as a brown solid. ^1^H NMR (500 MHz, chloroform-*d*) δ 8.16 (d, *J* = 8.7 Hz, 1H), 8.09 (d, *J* = 8.5 Hz, 1H), 7.85 (dd, *J* = 11.2, 8.2 Hz, 1H), 7.73 (ddd, *J* = 8.5, 6.9, 1.6 Hz, 1H), 7.65–7.50 (m, 1H), 7.44 (d, *J* = 8.7 Hz, 1H), 7.21–7.11 (m, 3H), 7.06 (dd, *J* = 7.8, 1.8 Hz, 2H), 6.58 (d, *J* = 5.5 Hz, 1H), 5.48 (d, *J* = 5.5 Hz, 1H), 3.91 (dt, *J* = 13.7, 7.1 Hz, 1H), 3.75 (dt, *J* = 13.4, 7.5 Hz, 1H), 2.89 (t, *J* = 7.5 Hz, 2H), 2.86–2.77 (m, 1H), 2.71–2.61 (m, 1H), 2.57–2.50 (m, 2H). ^13^C{^1^H} NMR (126 MHz, chloroform-*d*) δ 173.5, 165.3, 157.1, 147.3, 137.9, 137.5, 130.0, 129.9, 128.8, 128.5, 127.6, 127.4, 127.0, 126.6, 117.4, 116.4, 107.4, 69.4, 48.3, 34.6, 30.6, 29.7. HRMS (ESI) *m*/*z* calculated for C_24_H_22_N_3_O_2_ [M + H]^+^: 384.1632; found [M + H]^+^: 384.1636.

##### (*R*)-8*a*-(5-Methylpyridin-2-yl)-2-phenethyl-8,8*a*-dihydropyrrolo[1,2-*a*]pyrazine-1,6(2*H*,7*H*)-dione (**6q**)

Synthesis according to procedure **A** in the 1 mmol scale and purification by column chromatography (silica gel, petroleum ether/ethyl acetate = 1:3) afforded **6q** (257 mg, 68%) as a yellow solid. ^1^H NMR (500 MHz, chloroform-*d*) δ 8.44 (d, *J* = 2.8 Hz, 1H), 7.46 (dd, *J* = 8.0, 2.4 Hz, 1H), 7.28–7.16 (m, 3H), 7.12–7.03 (m, 3H), 6.48 (d, *J* = 5.7 Hz, 1H), 5.49 (d, *J* = 5.7 Hz, 1H), 3.91–3.82 (m, 1H), 3.81–3.69 (m, 1H), 2.93–2.81 (m, 2H), 2.79–2.69 (m, 1H), 2.67–2.56 (m, 1H), 2.54–2.44 (m, 2H), 2.35 (s, 3H). ^13^C{^1^H} NMR (126 MHz, chloroform-*d*) δ 173.3, 164.8, 158.6, 151.5, 139.6, 137.7, 128.9, 128.6, 126.7, 124.2, 118.3, 116.5, 107.0, 68.3, 48.0, 34.5, 30.7, 29.7. HRMS (ESI) *m*/*z* calculated for C_21_H_22_N_3_O_2_ [M + H]^+^: 348.1615; found [M + H]^+^: 348.1637.

##### (*R*)-8*a*-(6-Chloropyridin-2-yl)-2-phenethyl-8,8*a*-dihydropyrrolo[1,2-*a*]pyrazine-1,6(2*H*,7*H*)-dione (**6r**)

Synthesis according to procedure **A** in the 1 mmol scale and purification by column chromatography (silica gel, petroleum ether/ethyl acetate = 1:3) afforded **6r** (314 mg, 71%) as a yellow solid. ^1^H NMR (500 MHz, chloroform-*d*) δ 7.59 (t, *J* = 7.8 Hz, 1H), 7.28–7.25 (m, 2H), 7.25–7.20 (m, 2H), 7.15–7.09 (m, 2H), 7.07 (d, *J* = 7.6 Hz, 1H), 6.48 (d, *J* = 5.7 Hz, 1H), 5.56 (d, *J* = 5.7 Hz, 1H), 3.98–3.89 (m, 1H), 3.76 (dt, *J* = 13.9, 7.3 Hz, 1H), 2.90 (td, *J* = 7.3, 3.9 Hz, 2H), 2.78–2.66 (m, 2H), 2.56–2.41 (m, 2H). ^13^C{^1^H} NMR (126 MHz, chloroform-*d*) δ 173.5, 165.3, 154.6, 150.3, 137.9, 137.4, 132.9, 128.9, 128.5, 126.6, 119.1, 116.7, 106.7, 68.6, 48.3, 34.6, 30.7, 29.7, 18.1. HRMS (ESI) *m*/*z* calculated for C_20_H_19_ClN_3_O_2_ [M + H]^+^: 368.1162; found [M + H]^+^: 368.1165.

##### (*R*)-8*a*-(6-Bromopyridin-2-yl)-2-phenethyl-8,8*a*-dihydropyrrolo[1,2-*a*]pyrazine-1,6(2*H*,7*H*)-dione (**6s**)

Synthesis according to procedure **A** in the 1 mmol scale and purification by column chromatography (silica gel, petroleum ether/ethyl acetate = 1:3) afforded **6s** (165 mg, 40%) as a yellow solid. ^1^H NMR (500 MHz, chloroform-*d*) δ 7.50–7.42 (m, 2H), 7.27–7.21 (m, 3H), 7.14–7.08 (m, 3H), 6.48 (d, *J* = 5.7 Hz, 1H), 5.56 (d, *J* = 5.7 Hz, 1H), 3.94 (dt, *J* = 14.3, 7.3 Hz, 1H), 3.76 (dt, *J* = 13.6, 7.2 Hz, 1H), 2.91 (td, *J* = 7.3, 2.3 Hz, 2H), 2.77–2.67 (m, 2H), 2.54–2.44 (m, 2H). ^13^C{^1^H} NMR (126 MHz, chloroform-*d*) δ 173.3, 164.8, 159.0, 142.1, 139.2, 137.7, 128.9, 128.6, 128.0, 126.7, 118.7, 116.4, 107.1, 68.3, 46.4, 34.1, 30.7, 29.7. HRMS (ESI) *m*/*z* calculated for C_20_H_19_BrN_3_O_2_ [M + H]^+^: 412.0653; found [M + H]^+^: 412.0656.

##### (*R*)-8*a*-(5-Bromopyridin-2-yl)-2-phenethyl-8,8*a*-dihydropyrrolo[1,2-*a*]pyrazine-1,6(2*H*,7*H*)-dione (**6t**)

Synthesis according to procedure **A** in the 1 mmol scale and purification by column chromatography (silica gel, petroleum ether/ethyl acetate = 1:3) afforded **6t** (186 mg, 45%) as a yellow solid. ^1^H NMR (500 MHz, chloroform-*d*) δ 8.64 (d, *J* = 2.4 Hz, 1H), 7.76 (dd, *J* = 8.5, 2.4 Hz, 1H), 7.24 (dd, *J* = 5.0, 2.2 Hz, 3H), 7.10–7.06 (m, 3H), 6.49 (d, *J* = 5.5 Hz, 1H), 5.54 (d, *J* = 5.7 Hz, 1H), 3.96–3.89 (m, 1H), 3.74 (dt, *J* = 13.7, 7.2 Hz, 1H), 2.94–2.82 (m, 2H), 2.78–2.70 (m, 1H), 2.59 (ddd, *J* = 17.3, 11.0, 8.5 Hz, 1H), 2.53–2.42 (m, 2H). ^13^C{^1^H} NMR (126 MHz, chloroform-*d*) δ 173.3, 164.8, 156.1, 150.8, 139.6, 137.6, 128.8, 128.6, 126.7, 121.1, 120.5, 116.5, 106.8, 68.4, 48.0, 34.5, 30.7, 29.5. HRMS (ESI) *m*/*z* calculated for C_20_H_19_BrN_3_O_2_ [M + H]^+^: 412.0655; found [M + H]^+^: 412.0658.

##### (*R*)-2-Phenethyl-8*a*-(pyridin-4-yl)-8,8*a*-dihydropyrrolo[1,2-*a*]pyrazine-1,6(2*H*,7*H*)-dione (**6u**)

Synthesis according to procedure **A** in the 1 mmol scale and purification by column chromatography (silica gel, petroleum ether/ethyl acetate = 1:3) afforded **6u** (183 mg, 55%) as a yellow solid. ^1^H NMR (500 MHz, chloroform-*d*) δ 8.62 (d, *J* = 6.3 Hz, 2H), 7.19 (ddd, *J* = 13.1, 5.0, 2.1 Hz, 5H), 6.99–6.91 (m, 2H), 6.52 (d, *J* = 5.7 Hz, 1H), 5.48 (d, *J* = 5.7 Hz, 1H), 3.88 (dt, *J* = 13.6, 6.8 Hz, 1H), 3.69–3.61 (m, 1H), 2.89–2.73 (m, 3H), 2.53–2.34 (m, 3H). ^13^C{^1^H} NMR (126 MHz, chloroform-*d*) δ 172.9, 164.7, 150.4, 147.3, 137.4, 128.7, 128.6, 126.7, 120.0, 117.1, 105.8, 67.0, 48.3, 34.4, 32.5, 29.0. HRMS (ESI) *m*/*z* calculated for C_20_H_20_N_3_O_2_ [M + H]^+^: 334.1526; found [M + H]^+^: 334.1528.

##### (8*aR*,13*aS*)-8*a*-(Pyridin-2-yl)-5,6,9,10,13,13*a*-hexahydro-8*H*-pyrrolo[1′,2′:4,5]pyrazino[2,1-*a*]isoquinoline-8,11(8*aH*)-dione (**7a**)

Synthesis according to procedure **B** in the 1 mmol scale and purification by column chromatography (silica gel, petroleum ether/ethyl acetate = 1:3) afforded **7a** (300 mg, 90%) as a white solid. ^1^H NMR (500 MHz, chloroform-*d*) δ 8.65 (ddd, *J* = 4.7, 1.7, 1.1 Hz, 1H), 7.78 (td, *J* = 7.7, 1.9 Hz, 1H), 7.52 (d, *J* = 7.9 Hz, 1H), 7.32–7.29 (m, 1H), 7.24–7.16 (m, 3H), 7.04 (dd, *J* = 6.9, 2.2 Hz, 1H), 4.66 (ddd, *J* = 12.6, 4.7, 3.2 Hz, 1H), 4.42 (dd, *J* = 11.0, 5.5 Hz, 1H), 3.93 (dd, *J* = 12.6, 5.5 Hz, 1H), 3.60–3.54 (m, 1H), 3.09–3.02 (m, 2H), 2.93 (ddd, *J* = 16.1, 11.3, 4.7 Hz, 1H), 2.82 (dt, *J* = 15.8, 3.4 Hz, 1H), 2.61–2.53 (m, 1H), 2.42 (ddd, *J* = 17.0, 9.9, 3.5 Hz, 1H), 2.26 (ddd, *J* = 13.1, 9.3, 3.5 Hz, 1H). ^13^C{^1^H} NMR (126 MHz, chloroform-*d*) δ 175.1, 170.0, 159.3, 149.8, 137.5, 134.8, 132.5, 128.9, 127.6, 126.9, 125.6, 123.2, 119.6, 70.9, 52.1, 46.6, 39.2, 31.3, 29.5, 28.9. HRMS (ESI) *m*/*z* calculated for C_20_H_20_N_3_O_2_ [M + H]^+^: 334.1543; found [M + H]^+^: 334.1548.

##### (8*aR*,13*aS*)-2-Methoxy-8*a*-(pyridin-2-yl)-5,6,9,10,13,13*a*-hexahydro-8*H*-pyrrolo[1′,2′:4,5]pyrazino[2,1-*a*]isoquinoline-8,11(8*aH*)-dione (**7b**)

Synthesis according to procedure B in the 1 mmol scale and purification by column chromatography (silica gel, petroleum ether/ethyl acetate = 1:3) afforded 7b (200 mg, 55%) as a white solid. ^1^H NMR (500 MHz, chloroform-*d*) δ 8.65 (ddd, *J* = 4.9, 1.9, 0.9 Hz, 1H), 7.78 (td, *J* = 7.7, 1.8 Hz, 1H), 7.52 (dt, *J* = 7.9, 1.0 Hz, 1H), 7.30 (ddd, *J* = 7.4, 4.8, 1.1 Hz, 1H), 7.08 (dd, *J* = 8.4, 4.3 Hz, 1H), 6.78 (dd, *J* = 8.4, 2.7 Hz, 1H), 6.55 (d, *J* = 2.7 Hz, 1H), 4.64 (ddd, *J* = 12.8, 4.7, 3.3 Hz, 1H), 4.38 (dd, *J* = 11.0, 5.4 Hz, 1H), 3.92 (dd, *J* = 12.6, 5.5 Hz, 1H), 3.77 (s, 3H), 3.60–3.54 (m, 1H), 3.09–2.98 (m, 2H), 2.92–2.80 (m, 1H), 2.75 (dt, *J* = 15.4, 3.5 Hz, 1H), 2.62–2.53 (m, 1H), 2.42 (ddd, *J* = 17.0, 9.9, 3.4 Hz, 1H), 2.26 (ddd, *J* = 13.2, 9.3, 3.4 Hz, 1H). ^13^C{^1^H} NMR (126 MHz, chloroform-*d*) δ 175.1, 169.9, 159.3, 158.5, 149.8, 137.5, 133.4, 129.9, 126.8, 123.2, 119.6, 113.7, 110.7, 70.9, 55.4, 52.2, 46.6, 39.5, 31.4, 29.5, 28.0.. HRMS (ESI) *m*/*z* calculated for C_21_H_22_N_3_O_3_ [M + H]^+^: 364.1631; found [M + H]^+^: 364.1634.

##### (8*aR*,13*aS*)-4-Methoxy-8*a*-(pyridin-2-yl)-5,6,9,10,13,13*a*-hexahydro-8*H*-pyrrolo[1′,2′:4,5]pyrazino[2,1-*a*]isoquinoline-8,11(8*aH*)-dione (**7c**)

Synthesis according to procedure B in the 1 mmol scale and purification by column chromatography (silica gel, petroleum ether/ethyl acetate = 1:3) afforded 7c (164 mg, 45%) as a white solid. ^1^H NMR (500 MHz, chloroform-*d*) δ 8.67–8.62 (m, 1H), 7.78 (td, *J* = 7.8, 1.9 Hz, 1H), 7.52 (d, *J* = 8.0 Hz, 1H), 7.33–7.28 (m, 1H), 7.17 (t, *J* = 8.0 Hz, 1H), 6.76 (d, *J* = 8.0 Hz, 1H), 6.64 (d, *J* = 7.7 Hz, 1H), 4.71 (ddd, *J* = 12.9, 5.1, 2.8 Hz, 1H), 4.42 (dd, *J* = 10.8, 5.4 Hz, 1H), 3.90 (dd, *J* = 12.5, 5.0 Hz, 1H), 3.84 (s, 3H), 3.59–3.51 (m, 1H), 3.10–2.99 (m, 2H), 2.99–2.88 (m, 1H), 2.69–2.49 (m, 2H), 2.43 (dd, *J* = 9.9, 3.3 Hz, 1H), 2.26 (ddd, *J* = 12.8, 9.3, 3.4 Hz, 1H). ^13^C{^1^H} NMR (126 MHz, chloroform-*d*) δ 175.0, 169.9, 159.3, 156.7, 149.8, 137.5, 133.8, 127.4, 123.7, 123.2, 119.7, 117.5, 108.8, 70.8, 55.5, 52.0, 46.5, 38.8, 31.3, 29.5, 22.0. HRMS (ESI) *m*/*z* calculated for C_21_H_22_N_3_O_3_ [M + H]^+^: 364.1642; found [M + H]^+^: 364.1645.

##### (8*aR*,13*aS*)-8*a*-(6-Methylpyridin-2-yl) 5,6,9,10, 13,13*a*-hexahydro-8*H*-pyrrolo[1′,2′:4,5]pyrazino[2,1-*a*]isoquinoline-8,11(8*aH*)-dione (**7d**)

Synthesis according to procedure **B** in the 1 mmol scale and purification by column chromatography (silica gel, petroleum ether/ethyl acetate = 1:3) afforded **7d** (167 mg, 48%) as a white solid. ^1^H NMR (500 MHz, chloroform-*d*) δ 7.64 (t, *J* = 7.7 Hz, 1H), 7.29 (s, 1H), 7.24–7.20 (m, 2H), 7.20–7.17 (m, 1H), 7.14 (d, *J* = 7.6 Hz, 1H), 7.06–7.02 (m, 1H), 4.65 (ddd, *J* = 12.6, 4.7, 3.2 Hz, 1H), 4.48 (dd, *J* = 11.0, 5.4 Hz, 1H), 3.94 (dd, *J* = 12.5, 5.4 Hz, 1H), 3.60–3.52 (m, 1H), 3.09–2.99 (m, 2H), 2.98–2.90 (m, 1H), 2.82 (dt, *J* = 15.8, 3.4 Hz, 1H), 2.61 (d, *J* = 7.7 Hz, 1H), 2.58 (s, 3H), 2.40 (ddd, *J* = 16.9, 9.9, 3.3 Hz, 1H), 2.27 (ddd, *J* = 12.9, 9.3, 3.2 Hz, 1H). ^13^C{^1^H} NMR (126 MHz, chloroform-*d*) δ 175.0, 170.2, 158.8, 158.4, 137.5, 134.8, 132.7, 128.9, 127.5, 126.9, 125.6, 122.6, 116.5, 70.9, 52.1, 46.52, 39.2, 31.3, 29.6, 28.9, 24.8. HRMS (ESI) *m*/*z* calculated for C_21_H_22_N_3_O_2_ [M + H]^+^: 348.1635; found [M + H]^+^: 348.1638.

##### (8*aR*,13*aS*)-8*a*-(Quinolin-2-yl)-5,6,9,10,13,13*a*-hexahydro-8*H*-pyrrolo[1′,2′:4,5]pyrazino[2,1-*a*]isoquinoline-8,11(8*aH*)-dione (**7e**)

Synthesis according to procedure **B** in the 1 mmol scale and purification by column chromatography (silica gel, petroleum ether/ethyl acetate = 1:3) afforded **7e** (180 mg, 47%) as a white solid. ^1^H NMR (500 MHz, chloroform-*d*) δ 8.26 (d, *J* = 8.7 Hz, 1H), 8.15 (d, *J* = 7.6 Hz, 1H), 7.87 (d, *J* = 9.8 Hz, 1H), 7.83–7.73 (m, 1H), 7.66 (d, *J* = 8.5 Hz, 1H), 7.60 (t, *J* = 7.5 Hz, 1H), 7.33 (d, *J* = 7.6 Hz, 1H), 7.24–7.18 (m, 2H), 7.04–6.98 (m, 1H), 4.67 (dt, *J* = 12.5, 4.0 Hz, 1H), 4.51 (dd, *J* = 11.0, 5.4 Hz, 1H), 4.12 (dd, *J* = 12.5, 5.5 Hz, 1H), 3.65–3.57 (m, 2H), 3.15–3.03 (m, 1H), 3.00–2.91 (m, 1H), 2.89–2.83 (m, 2H), 2.66–2.52 (m, 1H), 2.50–2.32 (m, 1H). ^13^C{^1^H} NMR (126 MHz, chloroform-*d*) δ 175.0, 170.0, 161.1, 158.7, 147.4, 138.5, 138.0, 134.7, 132.5, 130.1, 130.0, 128.9, 128.8, 128.7, 127.5, 127.1, 126.9, 126.7, 125.6, 117.4, 71.6, 52.1, 46.9, 39.3, 35.5, 31.0, 28.9. HRMS (ESI) *m*/*z* calculated for C_24_H_22_N_3_O_2_ [M + H]^+^: 384.1613; found [M + H]^+^: 384.1616.

##### (8*aR*,13*aS*)-8*a*-(6-Bromopyridin-2-yl)-5,6,9,10,13,13*a*-hexahydro-8*H*-pyrrolo[1′,2′:4,5]pyrazino[2,1-*a*] isoquinoline-8,11(8*aH*)-dione (**7f**)

Synthesis according to procedure **B** in the 1 mmol scale and purification by column chromatography (silica gel, petroleum ether/ethyl acetate = 1:3) afforded **7f** (350 mg, 85%) as a brown solid. ^1^H NMR (500 MHz, chloroform-*d*) δ 7.62 (t, *J* = 7.8 Hz, 1H), 7.50 (dd, *J* = 7.7, 5.0 Hz, 2H), 7.25 (dd, *J* = 5.8, 3.4 Hz, 2H), 7.19 (dd, *J* = 5.4, 3.5 Hz, 1H), 7.13 (dd, *J* = 5.5, 3.6 Hz, 1H), 4.63 (ddd, *J* = 12.6, 4.8, 3.3 Hz, 1H), 4.53 (dd, *J* = 10.4, 5.5 Hz, 1H), 3.97 (dd, *J* = 12.7, 5.4 Hz, 1H), 3.65 (dd, *J* = 12.7, 10.0 Hz, 1H), 3.06 (ddd, *J* = 12.6, 11.2, 3.5 Hz, 1H), 3.01–2.91 (m, 2H), 2.89–2.79 (m, 1H), 2.67–2.54 (m, 1H), 2.40 (ddd, *J* = 16.9, 9.8, 3.0 Hz, 1H), 2.30 (ddd, *J* = 13.4, 9.2, 3.0 Hz, 1H). ^13^C{^1^H} NMR (126 MHz, chloroform-*d*) δ 174.7, 169.4, 160.4, 142.2, 139.7, 134.7, 132.5, 128.9, 127.9, 127.7, 127.1, 125.5, 118.8, 70.3, 52.5, 45.8, 39.6, 31.6, 29.4, 28.8. HRMS (ESI) *m*/*z* calculated for C_20_H_19_BrN_3_O_2_ [M + H]^+^: 412.0625; found [M + H]^+^: 412.0629.

##### (8*aR*,13*aS*)-8*a*-(Pyridin-4-yl)-5,6,9,10,13,13*a*-hexahydro-8*H*-pyrrolo[1′,2′:4,5]pyrazino[2,1-*a*]isoquinoline-8,11(8*aH*)-dione (**7g**)

Synthesis according to procedure **B** in the 1 mmol scale and purification by column chromatography (silica gel, petroleum ether/ethyl acetate = 1:3) afforded **7g** (237 mg, 71%) as a yellow solid. ^1^H NMR (500 MHz, chloroform-*d*) δ 8.72 (d, *J* = 6.3 Hz, 2H), 7.45–7.39 (m, 2H), 7.27–7.21 (m, 2H), 7.20 (dd, *J* = 6.5, 2.5 Hz, 1H), 7.07–7.01 (m, 1H), 4.70–4.62 (m, 1H), 4.45 (dd, *J* = 11.3, 5.2 Hz, 1H), 3.90 (dd, *J* = 12.8, 5.3 Hz, 1H), 3.59 (t, *J* = 12.1 Hz, 1H), 3.13–2.99 (m, 2H), 2.98–2.90 (m, 1H), 2.83 (dt, *J* = 15.8, 3.2 Hz, 1H), 2.58–2.47 (m, 1H), 2.43 (ddd, *J* = 17.2, 9.9, 3.6 Hz, 1H), 2.17 (ddd, *J* = 13.2, 9.1, 3.7 Hz, 1H). ^13^C{^1^H} NMR (126 MHz, chloroform-*d*) δ 175.2, 169.1, 151.0, 149.4, 134.7, 131.7, 129.1, 127.8, 127.1, 125.6, 119.4, 68.7, 51.9, 46.9, 39.3, 32.6, 29.0, 28.8. HRMS (ESI) *m*/*z* calculated for C_20_H_20_N_3_O_2_ [M + H]^+^: 334.1532; found [M + H]^+^: 334.1535.

##### (8*aR*,13*aS*)-2,3-Dimethoxy-8*a*-(pyridin-2-yl)-5,6,9,10,13,13*a*-hexahydro-8*H*-pyrrolo[1′,2′:4,5]pyrazino[2,1-*a*]isoquinoline-8,11(8*aH*)-dione (**7h**)

Synthesis according to procedure **C** in the 1 mmol scale and purification by column chromatography (silica gel, petroleum ether/ethyl acetate = 1:3) afforded **7h** (299 mg, 76%) as a yellow solid. ^1^H NMR (500 MHz, chloroform-*d*) δ 8.58–8.53 (m, 1H), 7.79 (td, *J* = 7.8, 1.9 Hz, 1H), 7.60 (d, *J* = 8.0 Hz, 1H), 7.32 (dd, *J* = 7.6, 4.7 Hz, 1H), 6.80–6.76 (m, 1H), 6.76–6.71 (m, 1H), 5.00 (s, 1H), 4.23 (dd, *J* = 14.0, 6.7 Hz, 1H), 3.97–3.89 (m, 1H), 3.87 (s, 3H), 3.86 (s, 3H), 3.63 (dt, *J* = 13.4, 7.2 Hz, 1H), 3.32 (dd, *J* = 13.4, 3.6 Hz, 1H), 2.88 (t, *J* = 7.5 Hz, 2H), 2.70 (ddd, *J* = 13.1, 8.4, 2.7 Hz, 1H), 2.66–2.45 (m, 2H), 2.38 (ddd, *J* = 16.6, 9.1, 2.8 Hz, 1H). ^13^C{^1^H} NMR (126 MHz, chloroform-*d*) δ 174.1, 170.3, 158.0, 149.1, 149.0, 147.7, 138.0, 131.2, 123.7, 121.4, 120.9, 112.0, 111.3, 78.7, 69.4, 55.94, 55.9, 46.5, 44.0, 33.9, 31.6, 29.9. HRMS (ESI) *m*/*z* calculated for C_22_H_24_N_3_O_4_ [M + H]^+^: 394.1733; found [M + H]^+^: 394.1738.

##### (8*aR*,13*aS*)-8*a*-(Pyrimidin-4-yl)-5,6,9,10,13,13*a*-hexahydro-8*H*-pyrrolo[1′,2′:4,5]pyrazino[2,1-*a*]isoquinoline-8,11(8*aH*)-dione (**7i**)

Synthesis according to procedure **C** in the 1 mmol scale and purification by column chromatography (silica gel, dichloromethane: methanol= 1:20) afforded **7i** (117 mg, 35%) as a light-yellow solid. ^1^H NMR (500 MHz, chloroform-*d*) δ 9.26 (s, 1H), 8.83 (s, 1H), 7.60 (s, 1H), 7.26–7.14 (m, 3H), 7.08 (d, *J* = 6.3 Hz, 1H), 4.62 (dt, *J* = 12.6, 4.1 Hz, 1H), 4.43 (dd, *J* = 10.4, 5.2 Hz, 1H), 3.90 (dd, *J* = 12.8, 5.2 Hz, 1H), 3.68–3.60 (m, 1H), 3.04 (td, *J* = 11.9, 3.6 Hz, 1H), 3.00–2.88 (m, 2H), 2.82 (d, *J* = 15.8 Hz, 1H), 2.53 (dt, *J* = 18.6, 9.4 Hz, 1H), 2.40 (ddd, *J* = 17.0, 9.9, 3.1 Hz, 1H), 2.25 (td, *J* = 9.5, 4.6 Hz, 1H). ^13^C{^1^H} NMR (126 MHz, chloroform-*d*) δ 174.7, 168.6, 167.9, 159.0, 158.1, 134.7, 132.1, 129.0, 127.8, 127.1, 125.4, 117.4, 70.0, 52.6, 45.7, 39.7, 31.4, 29.2, 28.7. HRMS (ESI) *m*/*z* calculated for C_19_H_19_N_4_O_2_ [M + H]^+^: 335.1432; found [M + H]^+^: 335.1437.

##### (*R*)-2-Phenethyl-8*a*-(pyridin-2-yl)tetrahydropyrrolo[1,2-*a*]pyrazine-1,6(2*H*,7*H*)-dione (**8a**)

Synthesis according to procedure **E** in the 0.3 mmol scale and purification by column chromatography (silica gel, petroleum ether/ethyl acetate = 1:3) afforded **8a** (100 mg, 98%) as a white solid. ^1^H NMR (500 MHz, chloroform-*d*) δ 8.59 (ddd, *J* = 4.9, 1.9, 1.0 Hz, 1H), 7.69 (td, *J* = 7.7, 1.8 Hz, 1H), 7.39 (d, *J* = 8.0 Hz, 1H), 7.27–7.20 (m, 4H), 7.17–7.13 (m, 2H), 3.95–3.82 (m, 2H), 3.53 (dt, *J* = 13.6, 7.2 Hz, 1H), 3.30–3.18 (m, 2H), 3.02–2.92 (m, 1H), 2.90 (t, *J* = 6.5 Hz, 2H), 2.76 (dt, *J* = 13.1, 9.3 Hz, 1H), 2.64–2.53 (m, 1H), 2.46–2.32 (m, 2H). ^13^C{^1^H} NMR (126 MHz, chloroform-*d*) δ 174.4, 169.6, 158.9, 149.5, 138.4, 137.1, 128.9, 128.6, 126.6, 123.0, 120.4, 70.1, 49.2, 45.9, 36.5, 33.7, 31.2, 30.0. HRMS (ESI) *m*/*z* calculated for C_20_H_22_N_3_O_2_ [M + H]^+^: 336.1624; found [M + H]^+^: 336.1627.

##### (8aR,13aR)-8*a*-(5-(2,3-Dihydrobenzo[*b*][1,4]dioxin-6-yl)pyridin-2-yl)-5,6,9,10,13,13*a*-hexahydro-8*H*-pyrrolo[1′,2′:4,5]pyrazino[2,1-*a*]isoquinoline-8,11(8*aH*)-dione (**8b**)

Synthesis according to procedure **F** in the 0.24 mmol scale and purification by column chromatography (silica gel, petroleum ether/ethyl acetate = 1:3) afforded **8b** (100 mg, 91%) as a white solid. ^1^H NMR (500 MHz, chloroform-*d*) δ 8.81 (dd, *J* = 2.4, 0.8 Hz, 1H), 7.89 (dd, *J* = 8.2, 2.4 Hz, 1H), 7.72–7.67 (m, 1H), 7.57 (td, *J* = 7.2, 1.5 Hz, 1H), 7.54 (d, *J* = 8.2 Hz, 1H), 7.51–7.46 (m, 1H), 7.21 (d, *J* = 13.7 Hz, 1H), 7.13 (d, *J* = 2.2 Hz, 1H), 7.08 (td, *J* = 8.7, 2.2 Hz, 1H), 6.99 (d, *J* = 8.4 Hz, 1H), 4.68 (dt, *J* = 12.6, 3.9 Hz, 1H), 4.51 (dd, *J* = 11.0, 5.4 Hz, 1H), 4.33 (s, 4H), 3.97 (dd, *J* = 12.6, 5.4 Hz, 1H), 3.63–3.55 (m, 1H), 3.07 (dt, *J* = 13.2, 9.5 Hz, 2H), 3.00–2.89 (m, 1H), 2.83 (dt, *J* = 15.8, 3.5 Hz, 1H), 2.70–2.54 (m, 1H), 2.44 (ddd, *J* = 16.9, 9.9, 3.4 Hz, 1H), 2.31 (ddd, *J* = 12.9, 9.3, 3.4 Hz, 1H). ^13^C{^1^H} NMR (126 MHz, chloroform-*d*) δ 175.1, 170.0, 157.4, 147.8, 144.1, 144.1, 135.4, 134.8, 132.2, 132.0, 130.4, 128.9, 128.6, 128.5, 127.6, 127.0, 125.6, 120.2, 119.5, 118.1, 115.9, 70.7, 64.4, 52.1, 46.6, 39.3, 31.4, 29.5, 28.9. HRMS (ESI) *m*/*z* calculated for C_28_H_26_N_3_O_4_ [M + H]^+^: 468.1815; found [M + H]^+^: 468.1818.

## Supporting Information Available

NMR spectra and crystal structure determinations (PDF)

## Accession Codes

CCDC 2089897–2089898 and 2141575 contain the supplementary crystallographic data for this paper. These data can be obtained free of charge via www.ccdc.cam.ac.uk/data_request/cif, or by emailing data_request@ccdc.cam.ac.uk, or by contacting The Cambridge Crystallographic Data Centre, 12 Union Road, Cambridge CB2 1EZ, UK; fax: +44 1223 336033.

CCDC 2089898 (**6a**) and CCDC 2089897 (**7a**) contain the supplementary crystallographic data for this paper. These data can be obtained free of charge via www.ccdc.cam.ac.uk/data_request/cif or by emailing data_request@ccdc.cam.ac.uk or by contacting the Cambridge Crystallographic Data Centre, 12 Union Road, Cambridge CB2 1EZ, UK; fax: +44 1223 336033.
