# Supplementary material for: Highly Stereoselective Ugi/Pictet–Spengler Sequence
Source: J Org Chem. 2022 May 12;87(11):7085–96. doi: 10.1021/acs.joc.2c00244 (PMC9490873; doi:10.1021/acs.joc.2c00244)
Supplement: Supplementary file 1 — jo2c00244_si_001.pdf [file jo2c00244_si_001.pdf]

Highly Stereoselective Ugi-Pictet-Spengler Sequence

Bidong Zhang[†], Katarzyna Kurpiewska[§], and Alexander Dömling[*†]

[†]University of Groningen, Department of Drug Design, A. Deusinglaan 1, 9713 AV Groningen, The Netherlands

[§]Department of Crystal Chemistry and Crystal Physics Faculty of Chemistry, Jagiellonian University, Gronostajowa 2, 30-387 Kraków, Poland

*e-mail: a.s.s.domling@rug.nl, www.drugdesign.nl

Table of contents

Characterization of Products (^{1}H NMR & ^{13}C NMR Chromatograms)………..S3-S89

X-ray structure determination……………………………………………………….S90-95

References……………………………………………………………………………S95

1. General procedure for isocyanides 1.

(a). General procedure for isocyanides 1a - 1n, 1p and 1q.

A solution of amine (20.0 mmol, 1.0 equiv.) was stirred at 0 ^{0}C, formic acid (34.6 mmol, 1.73 equiv.) was dropwise added, then the reaction mixture was heated to reflux for overnight. After completion, the solvents were removed under vacuum and the crude product was directly used for the next step without purification. The crude product was added to an flask containing triethylamine (100.0 mmol, 5.0 equiv.) in DCM, the $POCl_3$ (0.95 equiv.) was added dropwise over 20 minutes. The resulting mixture was stirred at 0 ^{0}C for 1 h and further stirred at r.t. for 2 h. A sodium bicarbonate solution was added and extracted with dichloromethane. The solvents were removed under vacuum and the crude product was purified by flash chromatography to give pure isocyanide **1.**

1a: (2-isocyanoethyl)benzene.

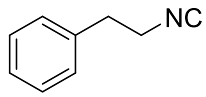

2.01 g, 77% yield, yellow oil. ^{1}H NMR (500 MHz, chloroform-d) δ 7.38 (t, *J* = 7.6 Hz, 2H), 7.32 (t, *J* = 7.3 Hz, 1H), 7.27 (d, *J* = 7.3 Hz, 2H), 3.64 (t, *J* = 7.1 Hz, 2H), 3.05 – 2.98 (t, 2H). ^{13}C{^{1}H} NMR (126 MHz, chloroform-d) δ 156.5 (q, J = 4.5 Hz), 136.7, 128.8, 128.7, 127.3, 43.0 (q, J = 5 Hz), 35.7.

1b: 1-chloro-4-isocyanobenzene.

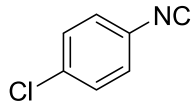

2.33 g, 85% yield, gray solid. ^{1}H NMR (500 MHz, chloroform-d) δ 7.41 (d, *J* = 8.8 Hz, 2H), 7.35 (d, *J* = 8.7 Hz, 2H). ^{13}C{^{1}H} NMR (126 MHz, chloroform-d) δ 165.6, 135.4, 129.8, 127.7.

1c: 1-fluoro-3-(isocyanomethyl)benzene.

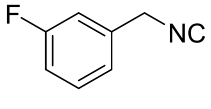

2.38 g, 88% yield, black oil. ^{1}H NMR (500 MHz, chloroform-d) δ 7.40 (td, *J* = 8.0, 5.7 Hz, 1H), 7.15 (d, *J* = 7.6 Hz, 1H), 7.12 – 7.05 (m, 2H), 4.67 (s, 2H). ^{13}C{^{1}H} NMR (126 MHz, chloroform-d) δ 164.0, 162.0, 158.5 (q, J = 4 Hz), 134.7 (d, J = 7 Hz), 130.7 (d, J = 7 Hz), 122.2 (d, J = 3 Hz), 115.5 (d, J = 17 Hz), 113.8 (d, J = 18 Hz), 45.0 (q, J = 2.6 Hz).

1d: 1-(isocyanomethyl)-4-methoxybenzene.

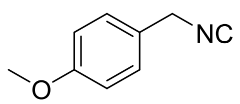

2.23 g, 76% yield, yellow oil. ^{1}H NMR (500 MHz, chloroform-d) δ 7.29 (d, *J* = 8.8 Hz, 2H), 6.94 (d, *J* = 8.8 Hz, 2H), 4.59 (s, 2H), 3.84 (s, 3H). ^{13}C{^{1}H} NMR (126 MHz, chloroform-d) δ 159.7, 157.1 (d, J = 2.5 Hz), 128.2, 124.6, 114.4, 55.4, 45.1 (t, J = 5.5 Hz).

1e: 5-(isocyanomethyl)benzo[d][1,3]dioxole.

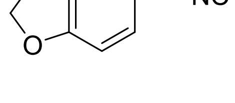

2.38 g, 74% yield, yellow solid. ^{1}H NMR (500 MHz, chloroform-d) δ 6.85 (s, 1H), 6.81 (d, J = 2.2 Hz, 2H), 6.00 (s, 2H), 4.55 (s, 2H). ^{13}C{^{1}H} NMR (126 MHz, chloroform-d) δ 157.4 (t, J = 4 Hz), 148.3, 147.8, 126.2, 120.4, 108.5, 107.4, 101.5, 45.4 (t, J = 5.5 Hz).

1f: (isocyanomethyl)benzene.

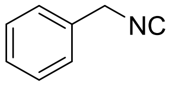

2.08 g, 89% yield, yellow solid. ^{1}H NMR (500 MHz, chloroform-d) δ 7.46 – 7.41 (m, 2H), 7.39 (t, J = 8.0 Hz, 3H), 4.66 (s, 2H). ^{13}C{^{1}H} NMR (126 MHz, chloroform-d) δ 157.7 (t, J = 4 Hz), 132.4, 129.0, 128.5, 126.7, 45.6 (t, J = 6 Hz).

1g: 1-isocyano-4-methylbenzene.

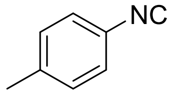

2.01 g, 86% yield, brown liquid. ^{1}H NMR (500 MHz, chloroform-d) δ 7.28 (d, J = 8.2 Hz, 2H), 7.20 (d, J = 8.4 Hz, 2H), 2.40 (s, 3H). ^{13}C{^{1}H} NMR (126 MHz, chloroform-d) δ 163.2 (d, J = 2.5 Hz), 139.7, 130.0, 126.2, 124.1 (t, J = 10.5 Hz), 21.4.

1h: methyl 3-isocyanopropanoate.

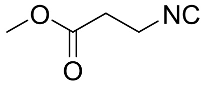

1.83 g, 81% yield, black liquid. ^{1}H NMR (500 MHz, chloroform-d) δ 3.73 (s, 3H), 3.68 (t, J = 6.8 Hz, 2H), 2.72 (t, J = 6.8 Hz, 2H). ^{13}C{^{1}H} NMR (126 MHz, chloroform-d) δ 169.9, 157.3 (t, J = 4 Hz), 52.23, 37.20 (t, J = 5.5 Hz), 33.91.

1i: methyl 2-isocyanoacetate.

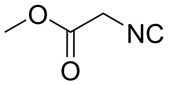

1.49 g, 75% yield, brown liquid. ^{1}H NMR (500 MHz, chloroform-d) δ 4.26 (s, 2H), 3.82 (s, 3H). ^{13}C{^{1}H} NMR (126 MHz, chloroform-d) δ 164.5, 161.1, 53.4, 43.4 (t, J = 7 Hz).

1j: isocyanobenzene.

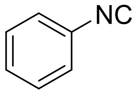

1.69 g, 82% yield, black liquid. ^{1}H NMR (500 MHz, chloroform-d) δ 7.44 – 7.39 (m, 3H), 7.38 (d, J = 5.5 Hz, 2H). ^{13}C{^{1}H} NMR (126 MHz, chloroform-d) δ 164.10 (t, J = 4.5 Hz), 129.5, 129.4, 126.6 (t, J = 10.5 Hz), 126.4.

1k: 1-(2-isocyanoethyl)-2-methoxybenzene.

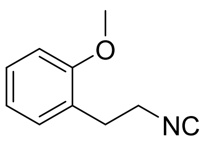

2.29 g, 71% yield, brown liquid. ^{1}H NMR (500 MHz, chloroform-d) δ 7.31 (td, *J* = 7.6, 1.8 Hz, 1H), 7.23 (dd, *J* = 7.4, 1.9 Hz, 1H), 6.97 (td, *J* = 7.4, 1.3 Hz, 1H), 6.92 (d, *J* = 8.2 Hz, 1H), 3.87 (s, 3H), 3.68 – 3.60 (m, 2H), 3.09 – 3.01 (m, 2H). ^{13}C{^{1}H} NMR (126 MHz, chloroform-d) δ 157.5, 155.8 (t, J = 5 Hz), 130.9, 128.7, 124.9, 120.7, 110.4, 55.3, 41.4 (t, J = 5.5 Hz), 31.2.

1l: 1-fluoro-4-(2-isocyanoethyl)benzene.

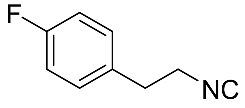

2.32 g, 78% yield, brown liquid. ^{1}H NMR (500 MHz, chloroform-d) δ 7.27 – 7.19 (m, 2H), 7.09 – 6.99 (m, 2H), 3.60 (td, *J* = 6.9, 2.1 Hz, 2H), 2.99 – 2.91 (m, 2H). ^{13}C{^{1}H} NMR (126 MHz, chloroform-d) δ 163.0, 161.1, 156.8 (t, J = 4.5 Hz), 132.5 (d, J = 1 Hz), 130.3 (d, J = 3.5 Hz), 115.6 (d, J = 3.5 Hz), 43.1 (t, J = 5 Hz), 34.7.

1m: 1-isocyano-3-methoxybenzene.

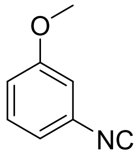

2.21 g, 83% yield, black liquid. ^{1}H NMR (500 MHz, chloroform-d) δ 7.29 (t, *J* = 8.2 Hz, 1H), 6.99 – 6.93 (m, 2H), 6.89 (s, 1H), 3.81 (s, 3H). ^{13}C{^{1}H} NMR (126 MHz, chloroform-d) δ 163.9, 160.1, 130.2, 127.4 (t, J = 10.5 Hz), 118.7, 115.7, 111.8, 55.6.

1n: isocyanocyclohexane.

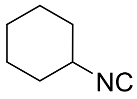

2.21 g, 83% yield, light yellow liquid. ^{1}H NMR (500 MHz, chloroform-d) δ 3.58 (s, 1H), 1.84 (d, *J* = 8.7 Hz, 2H), 1.75 – 1.68 (m, 2H), 1.64 (dt, *J* = 13.4, 6.7 Hz, 2H), 1.45 (q, *J* = 8.5, 6.8 Hz, 1H), 1.36 (q, *J* = 8.8, 7.9 Hz, 3H). ^{13}C{^{1}H} NMR (126 MHz, chloroform-d) δ 154.0 (t, J = 4 Hz), 51.6 (t, J = 4.5 Hz), 32.6, 24.9, 22.7.

1p: 1-(2-isocyanoethyl)-4-methoxybenzene.

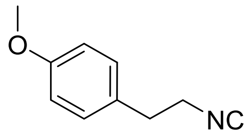

2.25 g, 70% yield, brown liquid. ^{1}H NMR (500 MHz, chloroform-d) δ 7.20 – 7.15 (m, 2H), 6.92 – 6.88 (m, 2H), 3.83 (s, 3H), 3.59 (tt, *J* = 6.9, 1.8 Hz, 2H), 3.00 – 2.92 (m, 2H). ^{13}C{^{1}H} NMR (126 MHz, chloroform-d) δ 158.8, 156.4, 129.7, 128.7, 114.2, 55.3, 43.3 (t, J = 5.5 Hz), 34.9.

1q: 4-(2-isocyanoethyl)-1,2-dimethoxybenzene.

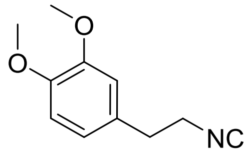

2.60 g, 68% yield, white solid. ^{1}H NMR (500 MHz, chloroform-d) δ 6.84 (d, *J* = 8.2 Hz, 1H), 6.80 – 6.75 (m, 2H), 3.89 (s, 3H), 3.88 (s, 3H), 3.59 (t, *J* = 7.0 Hz, 2H), 2.97 – 2.90 (m, 2H). ^{13}C{^{1}H} NMR (126 MHz, chloroform-d) δ 156.5 (t, J = 4.5 Hz), 149.1, 148.2, 129.3, 120.8, 111.9, 111.4, 55.9 (d, J = 1 Hz), 43.3 (t, J = 5 Hz), 35.3.

(b). Synthesis of 3-isocyanopropanenitrile 1o.

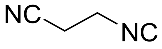

A solution of 3-aminopropionitrile (20.3 mmol, 1.5 mL) in ethyl formate (20.0 mL) was refluxed for five hours and subsequently stirred at room temperature overnight. The reaction was concentrated in vacuo and further dried under high vacuum. The N-(2-cyanoethyl)formamide was obtained as a light yellow oil (1.95 g, 19.9 mmol, 98%), this crude product was directly used to the next step. The N-(2-cyanoethyl)formamide (1.95 g, 19.9 mmol) was dissolved in CH_2Cl_2 (40.0 mL) and Et_3N (100.0 mol, 5.0 equiv., 13.9 mL) was added. The mixture was cooled to -5 ^{0}C at which $POCl_3$ (20.9 mmol, 1.05 equiv., 1.95 mL) was added drop wise over 60 minutes maintaining the temperature below 0 ^{0}C. After the addition the reaction was stirred at 0°C for an additional hour. An aqueous solution of Na_2CO_3 (0.6 M, 10.0 mL) was added carefully while the temperature increased to 20 °C. Additional water was added until all salts were dissolved (~30.0 mL). The mixture was transferred to a separatory funnel and the organic layer was separated. The water layer was extracted with dichloromethane (20.0 mL). The combined organic layers were washed with brine (10.0 mL), dried over $MgSO_4$, and concentrated in vacuo. The crude product was purified by filtration over silica (100% CH_2Cl_2) and after evaporation of the solvent obtained as a pale yellow oil (1.1 g, 69 %) which solidified upon standing. A pure sample (white solid) was obtained by trituration of a CH_2Cl_2 solution with petroleum ether at 0 °C. ^{1}H NMR (500 MHz, chloroform-d) δ 3.70 (t, *J* = 6.6 Hz, 2H), 2.79 (t, *J* = 6.5 Hz, 2H). ^{13}C{^{1}H} NMR (126 MHz, chloroform-d) δ ^{13}C NMR (126 MHz, $CDCl_3$) δ 159.8 (t, *J* = 3.5 Hz), 116.1, 37.7 (t, *J* = 6 Hz), 18.8.

2. General procedure for intermedia 5.

A solution of isocyanide **1** (1 mmol, 1.0 equiv.), aldehyde **2** (1 mmol, 1.0 equiv.), amine **3** (1 mmol, 1.0 equiv.) and acid **4** (1 mmol, 1.0 equiv.) in methanol (1 mL) was stirred at room temperature for 15 h. The solvents were removed under vacuum and the crude products were purified by flash column chromatography to give pure intermedia product **5.**

5a: 1-(2,2-dimethoxyethyl)-5-oxo-N-phenethyl-2-(pyridin-2-yl)pyrrolidine-2-carboxamide.

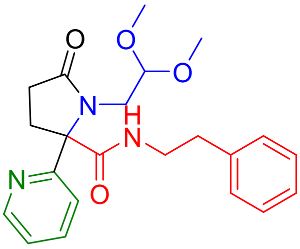

385 mg, 97% yield, yellow oil. mp 140 - 143 °C. ^{1}H NMR (500 MHz, chloroform-d) δ 8.66 (t, *J* = 5.8 Hz, 1H), 8.53 (dd, *J* = 4.8, 1.0 Hz, 1H), 7.61 (td, *J* = 7.7, 1.9 Hz, 1H), 7.35 – 7.30 (m, 2H), 7.28 – 7.24 (m, 3H), 7.24 – 7.21 (m, 1H), 7.19 (ddd, *J* = 7.6, 4.8, 1.2 Hz, 1H), 5.04 (dd, *J* = 7.6, 3.9 Hz, 1H), 3.75 – 3.61 (m, 2H), 3.37 (s, 6H), 3.15 (dd, *J* = 14.2, 3.8 Hz, 1H), 2.98 – 2.86 (m, 2H), 2.80 (td, *J* = 9.7, 9.1, 3.8 Hz, 1H), 2.67 – 2.53 (m, 2H), 2.53 – 2.43 (m, 2H). ^{13}C{^{1}H} NMR (126 MHz, chloroform-d) δ 179.1, 171.8, 158.4, 149.3, 139.0, 136.6, 129.0, 128.5, 126.5, 122.9, 122.8, 102.4, 75.1, 56.4, 55.4, 46.5, 40.8, 35.6, 33.8, 29.9. HRMS (ESI) m/z calculated for $C_{22}H_{28}N_3O_4$ $[M+H]^+$: 398.2031; found $[M+H]^+$: 398.2035.

5b: N-(4-chlorophenyl)-1-(2,2-dimethoxyethyl)-5-oxo-2-(pyridin-2-yl)pyrrolidine-2-carboxamide.

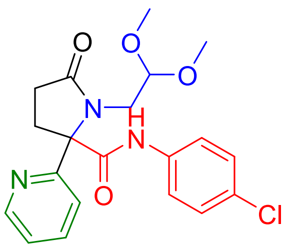

380 mg, 94% yield, yellow oil. mp 147 - 149 °C. ^{1}H NMR (500 MHz, chloroform-d) δ 10.91 (s, 1H), 8.66 – 8.61 (m, 1H), 7.78 – 7.72 (m, 1H), 7.72 – 7.64 (m, 2H), 7.53 (dd, *J* = 8.0, 1.1 Hz, 1H), 7.37 – 7.32 (m, 2H), 7.31 – 7.28 (m, 1H), 5.02 (dd, *J* = 6.5, 3.8 Hz, 1H), 3.47 (d, *J* = 3.8 Hz, 1H), 3.45 (s, 3H), 3.41 (s, 3H), 2.80 – 2.73 (m, 2H), 2.69 (t, *J* = 4.7 Hz, 3H). ^{13}C{^{1}H} NMR (126 MHz, chloroform-d) δ 178.9, 170.3, 158.6, 148.9, 137.2, 136.8, 129.5, 129.0, 123.1, 122.9, 121.6, 102.5, 74.7, 56.3, 55.4, 46.0, 34.9, 29.8. HRMS (ESI) m/z calculated for $C_{20}H_{23}ClN_3O_4$ [M+H]$^+$: 404.1320; found [M+H]$^+$: 404.1324.

5c: 1-(2,2-dimethoxyethyl)-N-(3-fluorobenzyl)-5-oxo-2-(pyridin-2-yl)pyrrolidine-2-carboxamide.

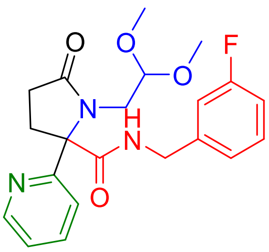

393 mg, 98% yield, yellow oil. mp 145 - 147 °C. ^{1}H NMR (500 MHz, chloroform-d) δ 9.11 (t, *J* = 5.6 Hz, 1H), 8.62 – 8.57 (m, 1H), 7.69 (td, *J* = 7.7, 1.9 Hz, 1H), 7.48 (dd, *J* = 8.0, 0.9 Hz, 1H), 7.34 (td, *J* = 8.0, 5.8 Hz, 1H), 7.24 (dd, *J* = 7.6, 4.7 Hz, 1H), 7.14 (d, *J* = 7.7 Hz, 1H), 7.09 (dd, *J* = 9.7, 2.3 Hz, 1H), 7.00 (td, *J* = 8.4, 2.7 Hz, 1H), 5.04 (dd, *J* = 7.6, 3.8 Hz, 1H), 4.65 (dd, *J* = 14.9, 6.1 Hz, 1H), 4.48 (dd, *J* = 14.8, 5.0 Hz, 1H), 3.33 (s, 3H), 3.25 (dd, *J* = 14.3, 3.7 Hz, 1H), 3.22 (s, 3H), 2.91 – 2.78 (m, 1H), 2.70 – 2.60 (m, 3H), 2.58 (d, *J* = 13.1 Hz, 1H). ^{13}C{^{1}H} NMR (126 MHz, chloroform-d) δ 179.0, 172.0, 164.0, 162.0, 158.4, 149.4, 140.9, 140.8, 136.8, 130.2, 130.2, 123.5, 123.5, 123.0, 122.7, 114.9, 114.7, 114.5, 114.3, 102.2, 75.2, 56.1, 55.2, 46.6, 43.3, 43.3, 34.1, 29.9. HRMS (ESI) m/z calculated for $C_{21}H_{25}FN_3O_4$ [M+H]$^+$: 401.1821; found [M+H]$^+$: 401.1826.

5d: 1-(2,2-dimethoxyethyl)-N-(4-methoxybenzyl)-5-oxo-2-(pyridin-2-yl)pyrrolidine-2-carboxamide.

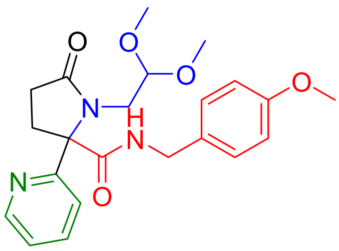

381 mg, 92% yield, yellow oil. mp 144 - 146 °C. ^{1}H NMR (500 MHz, chloroform-d) δ 8.93 (t, *J* = 5.4 Hz, 1H), 8.61 – 8.56 (m, 1H), 7.68 (td, *J* = 7.8, 1.9 Hz, 1H), 7.48 (d, *J* = 8.0 Hz, 1H), 7.29 (s, 1H), 7.28 (d, *J* = 2.2 Hz, 1H), 7.22 (dd, *J* = 7.6, 4.9 Hz, 1H), 6.95 – 6.85 (m, 2H), 5.03 (dd, *J* = 7.7, 3.6 Hz, 1H), 4.63 (dd, *J* = 14.3, 6.1 Hz, 1H), 4.38 (dd, *J* = 14.4, 4.7 Hz, 1H), 3.82 (s, 3H), 3.32 (s, 3H), 3.19 (dd, *J* = 14.0, 3.8 Hz, 1H), 3.14 (s, 3H), 2.94 – 2.83 (m, 1H), 2.70 – 2.49 (m, 4H). ^{13}C{^{1}H} NMR (126 MHz, chloroform-d) δ 179.1, 171.7, 159.0, 158.4, 149.4, 136.7, 130.3, 129.4, 122.9, 122.9, 114.0, 102.1, 75.3, 56.1, 55.3, 55.3, 46.7, 43.3, 34.0, 30.0. HRMS (ESI) m/z calculated for $C_{22}H_{28}N_3O_5$ [M+H]$^+$: 414.2043; found [M+H]$^+$: 414.2045.

5e: N-(benzo[d][1,3]dioxol-5-ylmethyl)-1-(2,2-dimethoxyethyl)-5-oxo-2-(pyridin-2-yl)pyrrolidine-2 carboxamide.

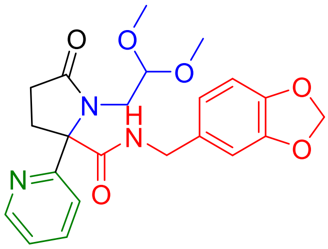

410 mg, 96% yield, yellow oil. mp 143 - 146 °C. ^{1}H NMR (500 MHz, chloroform-d) δ 8.96 (t, *J* = 5.8 Hz, 1H), 8.61 – 8.56 (m, 1H), 7.68 (tt, *J* = 7.7, 1.7 Hz, 1H), 7.48 (d, *J* = 8.0 Hz, 1H), 7.23 (ddt, *J* = 7.6, 4.9, 1.4 Hz, 1H), 6.86 (s, 1H), 6.86 – 6.75 (m, 2H), 5.97 (s, 2H), 5.04 (ddd, *J* = 7.7, 3.6, 1.4 Hz, 1H), 4.59 (dd, *J* = 13.3, 6.2 Hz, 1H), 4.35 (dd, *J* = 14.4, 4.8 Hz, 1H), 3.34 (s, 3H), 3.23 (s, 3H), 3.20 (d, *J* = 3.5 Hz, 1H), 2.92 – 2.80 (m, 1H), 2.72 – 2.49 (m, 4H). ^{13}C{^{1}H} NMR (126 MHz, chloroform-d) δ 179.1, 171.8, 158.4, 149.4, 147.9, 147.0, 136.7, 132.1, 123.0, 122.8, 121.3, 108.6, 108.3, 102.2, 101.1, 75.3, 56.2, 55.3, 46.6, 43.6, 34.0, 30.0. HRMS (ESI) m/z calculated for $C_{22}H_{26}N_3O_6$ $[M+H]^+$: 428.1732; found $[M+H]^+$: 428.1736.

5f: N-benzyl-1-(2,2-dimethoxyethyl)-5-oxo-2-(pyridin-2-yl)pyrrolidine-2-carboxamide.

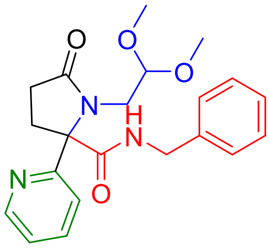

364 mg, 95% yield, yellow oil. mp 140 - 142 °C. ^{1}H NMR (500 MHz, chloroform-d) δ 9.02 (t, *J* = 5.5 Hz, 1H), 8.61 – 8.56 (m, 1H), 7.68 (td, *J* = 7.7, 2.0 Hz, 1H), 7.49 (d, *J* = 7.9 Hz, 1H), 7.40 – 7.34 (m, 4H), 7.31 (dd, *J* = 6.0, 2.7 Hz, 1H), 7.23 (dd, *J* = 7.5, 4.8 Hz, 1H), 5.03 (dd, *J* = 7.7, 3.6 Hz, 1H), 4.70 (dd, *J* = 14.6, 6.2 Hz, 1H), 4.45 (dd, *J* = 14.7, 4.7 Hz, 1H), 3.32 (s, 3H), 3.21 (dd, *J* = 14.2, 3.6 Hz, 1H), 3.12 (s, 3H), 2.96 – 2.81 (m, 1H), 2.71 – 2.51 (m, 4H). ^{13}C{^{1}H} NMR (126 MHz, chloroform-d) δ 179.1, 171.8, 158.4, 149.4, 138.1, 136.7, 128.7, 128.1, 127.5, 123.0, 122.9, 102.1, 75.3, 56.0, 55.3, 46.7, 43.9, 34.0, 30.0. HRMS (ESI) m/z calculated for $C_{21}H_{26}N_3O_4$ $[M+H]^+$: 384.1824; found $[M+H]^+$: 384.1827.

5g: 1-(2,2-dimethoxyethyl)-5-oxo-2-(pyridin-2-yl)-N-(p-tolyl)pyrrolidine-2-carboxamide.

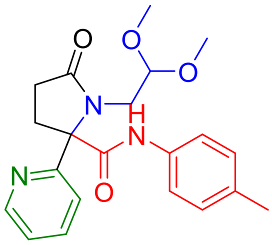

368 mg, 96% yield, yellow oil. mp 141 - 143 °C. ^{1}H NMR (500 MHz, chloroform-d) δ 10.64 (s, 1H), 8.63 (ddd, *J* = 4.7, 1.9, 0.9 Hz, 1H), 7.72 (td, *J* = 7.8, 1.9 Hz, 1H), 7.62 – 7.57 (m, 2H), 7.55 (d, *J* = 8.0 Hz, 1H), 7.28 – 7.25 (m,

1H), 7.19 (d, *J* = 8.4 Hz, 2H), 5.07 (dd, *J* = 7.3, 3.9 Hz, 1H), 3.47 (s, 3H), 3.41 (m, 4H), 2.86 – 2.73 (m, 2H), 2.72 – 2.65 (m, 3H), 2.36 (s, 3H). $^{13}C\{^1H\}$ NMR (126 MHz, chloroform-d) δ 179.1, 170.0, 158.7, 149.0, 137.0, 135.5, 134.2, 129.5, 123.0, 123.0, 120.4, 102.4, 75.0, 56.3, 55.3, 46.2, 34.7, 29.9, 20.9. HRMS (ESI) m/z calculated for $C_{21}H_{26}N_3O_4$ $[M+H]^+$: 384.1826; found $[M+H]^+$: 384.1828.

5h: methyl 3-(1-(2,2-dimethoxyethyl)-5-oxo-2-(pyridin-2-yl)pyrrolidine-2-carboxamido)propanoate.

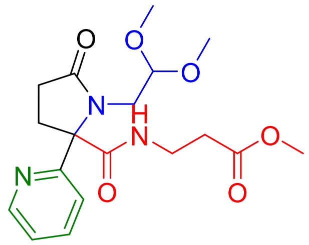

345 mg, 91% yield, yellow oil. mp 144 - 146 °C. ^1H NMR (500 MHz, chloroform-d) δ 8.83 (t, *J* = 5.8 Hz, 1H), 8.58 (d, *J* = 4.9 Hz, 1H), 7.69 (td, *J* = 7.8, 2.2 Hz, 1H), 7.48 (d, *J* = 8.0 Hz, 1H), 7.23 (dd, *J* = 7.6, 4.9 Hz, 1H), 5.03 (dd, *J* = 7.3, 4.0 Hz, 1H), 3.71 (s, 3H), 3.68 – 3.61 (m, 2H), 3.49 (s, 3H), 3.38 (s, 3H), 3.22 (dd, *J* = 14.2, 3.9 Hz, 1H), 2.80 (td, *J* = 10.2, 9.4, 3.6 Hz, 2H), 2.72 – 2.60 (m, 4H), 2.59 – 2.48 (m, 2H). $^{13}C\{^1H\}$ NMR (126 MHz, chloroform-d) δ 178.9, 172.2, 172.1, 158.4, 149.3, 136.7, 122.9, 122.9, 102.3, 75.0, 56.4, 55.3, 51.8, 46.4, 35.7, 33.9, 33.9, 29.9. HRMS (ESI) m/z calculated for $C_{18}H_{26}N_3O_6$ $[M+H]^+$: 380.1761; found $[M+H]^+$: 380.1765.

5i: methyl (1-(2,2-dimethoxyethyl)-5-oxo-2-(pyridin-2-yl)pyrrolidine-2-carbonyl)glycinate.

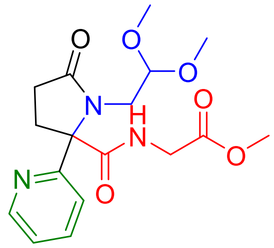

329 mg, 90% yield, yellow oil. mp 143 - 146 °C. ^1H NMR (500 MHz, chloroform-d) δ 9.18 (t, *J* = 5.8 Hz, 1H), 8.62 – 8.57 (m, 1H), 7.71 (td, *J* = 7.7, 2.0 Hz, 1H), 7.54 (d, *J* = 9.0 Hz, 1H), 7.24 (dd, *J* = 7.6, 4.7 Hz, 1H), 5.06 (dd, *J* = 7.3, 4.2 Hz, 1H), 4.13 (t, *J* = 5.8 Hz, 2H), 3.79 (s, 3H), 3.45 (s, 3H), 3.38 (s, 3H), 3.29 (dd, *J* = 14.3, 4.1 Hz, 1H), 2.89 – 2.78 (m, 1H), 2.73 (dd, *J* = 14.2, 7.4 Hz, 1H), 2.68 – 2.56 (m, 3H). $^{13}C\{^1H\}$ NMR (126 MHz, chloroform-d) δ 178.9, 172.8, 170.0, 158.2, 149.2, 136.8, 123.0, 123.0, 102.1, 75.0, 56.4, 54.9, 52.3, 46.1, 41.3, 33.9, 29.9. HRMS (ESI) m/z calculated for $C_{17}H_{24}N_3O_6$ $[M+H]^+$: 366.1614; found $[M+H]^+$: 366.1617.

5j: 1-(2,2-dimethoxyethyl)-5-oxo-N-phenyl-2-(pyridin-2-yl)pyrrolidine-2-carboxamide.

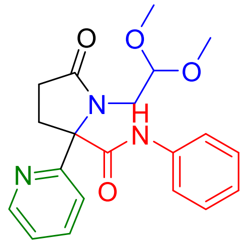

343 mg, 93% yield, yellow oil. mp 142 - 144 °C. ^1H NMR (500 MHz, chloroform-d) δ 10.77 (s, 1H), 8.67 – 8.61 (m, 1H), 7.75 (dd, *J* = 7.9, 1.9 Hz, 1H), 7.72 (d, *J* = 7.4 Hz, 2H), 7.55 (d, *J* = 8.0 Hz, 1H), 7.42 – 7.37 (m, 2H), 7.29 –

7.26 (m, 1H), 7.18 (t, *J* = 7.4 Hz, 1H), 5.07 (dd, *J* = 7.3, 3.9 Hz, 1H), 3.48 (s, 3H), 3.46 – 3.42 (m, 1H), 3.42 (s, 3H), 2.85 – 2.75 (m, 2H), 2.74 – 2.65 (m, 3H). $^{13}C\{^1H\}$ NMR (126 MHz, chloroform-d) δ 179.0, 170.2, 158.7, 149.0, 138.1, 137.0, 129.0, 124.6, 123.0, 123.0, 120.4, 102.4, 74.9, 56.3, 55.3, 46.1, 34.8, 29.9. HRMS (ESI) m/z calculated for $C_{20}H_{24}N_3O_4$ $[M+H]^+$: 370.1743; found $[M+H]^+$: 370.1744.

5k: 1-(2,2-dimethoxyethyl)-N-(2-methoxyphenethyl)-5-oxo-2-(pyridin-2-yl)pyrrolidine-2-carboxamide.

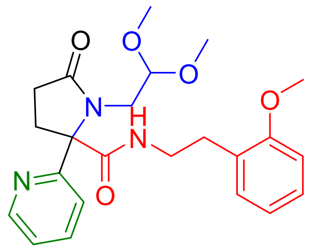

393 mg, 92% yield, yellow oil. mp 145 - 147 °C. ^1H NMR (500 MHz, chloroform-d) δ 8.54 (d, *J* = 5.7 Hz, 2H), 7.62 (td, *J* = 7.7, 2.0 Hz, 1H), 7.27 (d, *J* = 8.0 Hz, 1H), 7.24 (td, *J* = 7.6, 1.7 Hz, 1H), 7.21 – 7.19 (m, 1H), 7.18 (d, *J* = 6.3 Hz, 1H), 6.96 – 6.78 (m, 2H), 5.03 (dd, *J* = 7.6, 3.8 Hz, 1H), 3.85 (s, 3H), 3.67 (q, *J* = 6.3, 5.4 Hz, 2H), 3.39 (s, 3H), 3.37 (s, 3H), 3.15 (dd, *J* = 14.2, 3.8 Hz, 1H), 2.97 (dt, *J* = 13.4, 6.7 Hz, 1H), 2.92 – 2.76 (m, 2H), 2.66 – 2.54 (m, 2H), 2.53 – 2.43 (m, 2H). $^{13}C\{^1H\}$ NMR (126 MHz, chloroform-d) δ 179.1, 171.7, 158.4, 157.8, 149.3, 136.5, 130.7, 127.8, 127.3, 123.0, 122.8, 120.4, 110.4, 102.3, 75.2, 56.2, 55.4, 55.3, 46.5, 39.4, 33.7, 30.5, 29.9. HRMS (ESI) m/z calculated for $C_{23}H_{30}N_3O_5$ $[M+H]^+$: 428.2130; found $[M+H]^+$: 428.2135.

5l: 1-(2,2-dimethoxyethyl)-N-(4-fluorophenethyl)-5-oxo-2-(pyridin-2-yl)pyrrolidine-2-carboxamide.

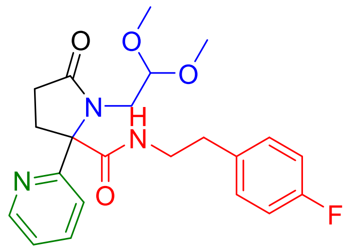

382 mg, 92% yield, yellow oil. mp 146 - 148 °C. ^1H NMR (500 MHz, chloroform-d) δ 8.67 (t, *J* = 5.8 Hz, 1H), 8.53 (ddd, *J* = 4.7, 1.9, 1.0 Hz, 1H), 7.62 (td, *J* = 7.8, 1.9 Hz, 1H), 7.25 – 7.21 (m, 2H), 7.21 – 7.18 (m, 1H), 7.16 (dt, *J* = 7.9, 1.1 Hz, 1H), 7.04 – 6.97 (m, 2H), 5.05 (dd, *J* = 7.6, 3.8 Hz, 1H), 3.74 – 3.66 (m, 1H), 3.61 (td, *J* = 12.8, 5.6 Hz, 1H), 3.41 (s, 3H), 3.39 (s, 3H), 3.14 (dd, *J* = 14.3, 3.9 Hz, 1H), 2.89 (q, *J* = 7.7, 7.3 Hz, 2H), 2.82 – 2.75 (m, 1H), 2.65 – 2.58 (m, 1H), 2.55 (dd, *J* = 14.2, 7.6 Hz, 1H), 2.51 – 2.43 (m, 2H). $^{13}C\{^1H\}$ NMR (126 MHz, chloroform-d) δ 179.1, 171.8, 162.7, 160.7, 158.4, 149.3, 136.6, 134.6, 134.6, 130.4, 130.4, 122.9, 122.8, 115.4, 115.2, 102.5, 75.1, 56.4, 55.6, 46.6, 40.8, 34.8, 33.8, 29.9. HRMS (ESI) m/z calculated for $C_{22}H_{27}FN_3O_4$ $[M+H]^+$: 416.1904; found $[M+H]^+$: 416.1907.

5m: 1-(2,2-dimethoxyethyl)-N-(3-methoxyphenyl)-5-oxo-2-(pyridin-2-yl)pyrrolidine-2-carboxamide.

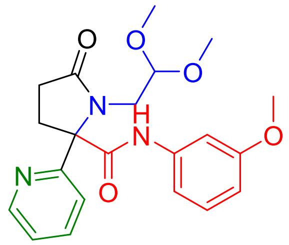

379 mg, 95% yield, yellow oil. mp 144 - 147 °C. ^{1}H NMR (500 MHz, chloroform-d) δ 10.78 (s, 1H), 8.63 (dq, *J* = 4.7, 1.7 Hz, 1H), 7.74 (tt, *J* = 7.7, 1.7 Hz, 1H), 7.54 (dd, *J* = 8.0, 1.6 Hz, 1H), 7.48 (q, *J* = 1.9 Hz, 1H), 7.29 (d, *J* = 3.2 Hz, 1H), 7.28 – 7.26 (m, 1H), 7.20 (dd, *J* = 8.0, 1.1 Hz, 1H), 6.73 (ddt, *J* = 8.2, 2.5, 1.2 Hz, 1H), 5.05 (ddd, *J* = 7.3, 3.9, 1.7 Hz, 1H), 3.85 (s, 3H), 3.48 (s, 3H), 3.43 (dd, *J* = 14.5, 2.4 Hz, 1H), 3.41 (s, 3H), 2.85 – 2.74 (m, 2H), 2.74 – 2.64 (m, 3H). ^{13}C{^{1}H} NMR (126 MHz, chloroform-d) δ 179.0, 170.2, 160.1, 158.7, 148.9, 139.4, 137.1, 129.7, 123.017, 123.0, 112.6, 110.5, 106.1, 102.4, 74.9, 56.3, 55.3, 55.2, 46.1, 34.8, 30.0. HRMS (ESI) m/z calculated for $C_{21}H_{26}N_3O_5$ [M+H]$^+$: 400.1845; found [M+H]$^+$: 400.1848.

5n: N-cyclohexyl-1-(2,2-dimethoxyethyl)-5-oxo-2-(pyridin-2-yl)pyrrolidine-2-carboxamide.

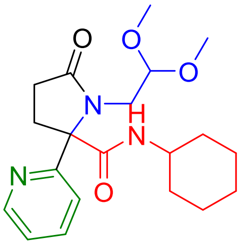

341 mg, 91% yield, yellow oil. mp 140 - 142 °C. ^{1}H NMR (500 MHz, chloroform-d) δ 8.60 – 8.55 (m, 1H), 8.33 (d, *J* = 8.0 Hz, 1H), 7.67 (td, *J* = 7.7, 2.0 Hz, 1H), 7.47 (d, *J* = 6.9 Hz, 1H), 7.21 (dd, *J* = 7.6, 4.7 Hz, 1H), 5.14 (dd, *J* = 7.6, 3.9 Hz, 1H), 3.89 (tdt, *J* = 11.5, 7.9, 3.9 Hz, 1H), 3.50 (s, 3H), 3.39 (s, 3H), 3.16 (dd, *J* = 14.2, 3.9 Hz, 1H), 2.95 – 2.81 (m, 1H), 2.72 – 2.48 (m, 4H), 2.07 – 1.93 (m, 2H), 1.84 – 1.72 (m, 2H), 1.69 (dt, *J* = 12.9, 3.8 Hz, 1H), 1.40 (tdd, *J* = 16.6, 8.2, 3.8 Hz, 2H), 1.33 – 1.12 (m, 3H). ^{13}C{^{1}H} NMR (126 MHz, chloroform-d) δ 179.2, 170.8, 158.6, 149.4, 136.5, 123.0, 122.8, 102.1, 75.1, 56.3, 55.1, 48.7, 46.5, 33.7, 33.1, 33.0, 30.0, 25.6, 25.2, 25.1. HRMS (ESI) m/z calculated for $C_{20}H_{30}N_3O_4$ [M+H]$^+$: 376.2251; found [M+H]$^+$: 376.2253.

5o: N-(2-cyanoethyl)-1-(2,2-dimethoxyethyl)-5-oxo-2-(pyridin-2-yl)pyrrolidine-2-carboxamide.

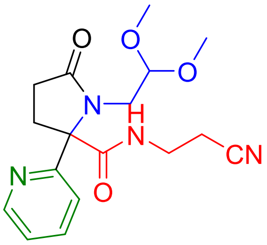

320 mg, 92% yield, yellow oil. mp 141 - 144 °C. ^{1}H NMR (500 MHz, chloroform-d) δ 9.31 (t, *J* = 5.8 Hz, 1H), 8.62 – 8.56 (m, 1H), 7.71 (td, *J* = 7.8, 1.9 Hz, 1H), 7.48 (d, *J* = 8.0 Hz, 1H), 7.25 (dd, *J* = 7.6, 4.7 Hz, 1H), 5.06 (dd, *J* = 7.5, 4.0 Hz, 1H), 3.71 – 3.56 (m, 2H), 3.51 (s, 3H), 3.39 (s, 3H), 3.29 (dd, *J* = 14.3, 4.0 Hz, 1H), 2.83 – 2.69 (m, 4H), 2.68 – 2.52 (m, 3H). ^{13}C{^{1}H} NMR (126 MHz, chloroform-d) δ 178.8, 172.9, 158.2, 149.3, 136.9, 123.1, 122.6, 118.0, 102.3, 74.8, 56.7, 55.2, 46.3, 35.8, 34.1, 29.8, 18.1. HRMS (ESI) m/z calculated for $C_{17}H_{23}N_4O_4$ [M+H]$^+$: 347.1612; found [M+H]$^+$: 347.1616.

5p: 1-(2,2-dimethoxyethyl)-5-oxo-N-phenethyl-2-(quinolin-2-yl)pyrrolidine-2-carboxamide.

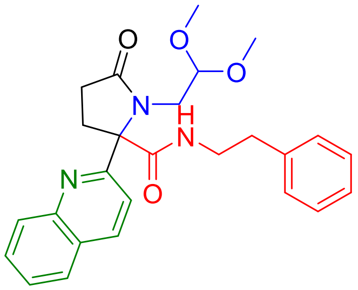

426 mg, 95% yield, yellow oil. mp 145 - 147 °C. ^1H NMR (500 MHz, chloroform-d) δ 9.10 (t, *J* = 5.7 Hz, 1H), 8.12 (d, *J* = 8.7 Hz, 1H), 7.87 (d, *J* = 8.5 Hz, 1H), 7.82 (d, *J* = 8.2 Hz, 1H), 7.74 – 7.67 (m, 1H), 7.56 (t, *J* = 7.6 Hz, 1H), 7.42 (d, *J* = 8.5 Hz, 1H), 7.33 (dt, *J* = 13.4, 7.0 Hz, 4H), 7.26 (d, *J* = 6.9 Hz, 1H), 4.98 (dd, *J* = 7.3, 3.9 Hz, 1H), 3.75 (q, *J* = 6.7 Hz, 2H), 3.37 (s, 3H), 3.33 (s, 3H), 3.31 (d, *J* = 3.9 Hz, 1H), 2.98 (t, *J* = 7.0 Hz, 2H), 2.87 – 2.82 (m, 1H), 2.79 (dd, *J* = 14.3, 7.3 Hz, 1H), 2.72 – 2.64 (m, 1H), 2.61 (dd, *J* = 9.5, 6.5 Hz, 1H), 2.58 – 2.49 (m, 1H). $^{13}C\{^1H\}$ NMR (126 MHz, chloroform-d) δ 179.0, 171.8, 159.2, 147.0, 139.1, 136.8, 129.7, 129.4, 128.9, 128.6, 127.4, 127.1, 127.0, 126.5, 120.4, 102.4, 75.0, 56.03, 55.2, 46.7, 40.8, 35.7, 34.3, 30.0. HRMS (ESI) m/z calculated for $C_{26}H_{30}N_3O_4$ $[M+H]^+$: 448.2261; found $[M+H]^+$: 448.2265.

5q: 1-(2,2-dimethoxyethyl)-2-(5-methylpyridin-2-yl)-5-oxo-N-phenethylpyrrolidine-2-carboxamide.

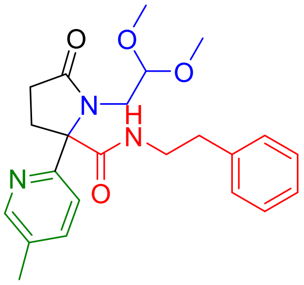

392 mg, 95% yield, yellow oil. mp 143 - 145 °C. ^1H NMR (500 MHz, chloroform-d) δ 8.65 (t, *J* = 5.8 Hz, 1H), 8.38 – 8.33 (m, 1H), 7.41 (dd, *J* = 8.7, 1.9 Hz, 1H), 7.33 (t, *J* = 7.7 Hz, 2H), 7.28 – 7.23 (m, 3H), 7.11 (d, *J* = 8.0 Hz, 1H), 5.02 (dd, *J* = 7.6, 3.5 Hz, 1H), 3.75 – 3.60 (m, 2H), 3.38 (s, 3H), 3.37 (s, 3H), 3.17 (dd, *J* = 14.2, 3.9 Hz, 1H), 2.92 (t, *J* = 7.2 Hz, 2H), 2.83 – 2.72 (m, 1H), 2.64 – 2.52 (m, 2H), 2.52 – 2.42 (m, 2H), 2.32 (s, 3H). $^{13}C\{^1H\}$ NMR (126 MHz, chloroform-d) δ 178.9, 172.0, 155.4, 149.7, 139.0, 137.0, 132.4, 129.0, 128.5, 126.4, 122.3, 102.3, 75.0, 56.3, 55.3, 46.4, 40.8, 35.6, 33.8, 29.9, 18.1. HRMS (ESI) m/z calculated for $C_{23}H_{30}N_3O_4$ $[M+H]^+$: 412.2215; found $[M+H]^+$: 412.2218.

5r: 2-(6-chloropyridin-2-yl)-1-(2,2-dimethoxyethyl)-5-oxo-N-phenethylpyrrolidine-2-carboxamide.

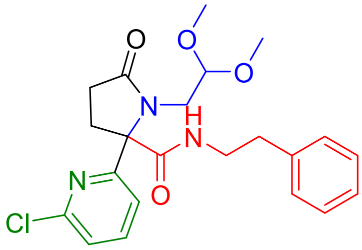

388 mg, 90% yield, yellow oil. mp 147 - 149 °C. ^1H NMR (500 MHz, chloroform-d) δ 8.44 (t, *J* = 5.8 Hz, 1H), 7.55 (t, *J* = 7.8 Hz, 1H), 7.35 – 7.30 (m, 2H), 7.28 – 7.24 (m, 3H), 7.23 (d, *J* = 7.9 Hz, 1H), 7.09 (d, *J* = 7.7 Hz, 1H), 5.11 (dd, *J* = 7.8, 3.7 Hz, 1H), 3.71 – 3.63 (m, 2H), 3.41 (s, 6H), 3.17 (dd, *J* = 14.0, 3.8 Hz, 1H), 2.98 – 2.88 (m, 2H), 2.90 – 2.80 (m, 1H), 2.70 – 2.58 (m, 1H), 2.50 (dd, *J* = 14.0, 7.7 Hz, 1H), 2.47 – 2.38 (m, 2H). $^{13}C\{^1H\}$ NMR (126 MHz, chloroform-d) δ 178.9, 171.3, 158.8, 151.1, 139.1, 138.8, 129.0, 128.6, 126.5, 123.8, 121.7, 102.5, 75.0, 56.7, 55.8, 46.7, 40.8, 35.6, 33.5, 29.7. HRMS (ESI) m/z calculated for $C_{22}H_{27}ClN_3O_4$ $[M+H]^+$: 432.1631; found $[M+H]^+$: 432.1635.

5s: 2-(6-bromopyridin-2-yl)-1-(2,2-dimethoxyethyl)-5-oxo-N-phenethylpyrrolidine-2-carboxamide.

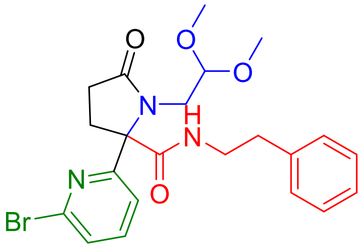

414 mg, 87% yield, yellow oil. mp 146 - 148 °C. ^{1}H NMR (500 MHz, chloroform-d) δ 8.44 (t, *J* = 5.8 Hz, 1H), 7.44 (t, *J* = 7.7 Hz, 1H), 7.38 (d, *J* = 7.9 Hz, 1H), 7.35 – 7.30 (m, 2H), 7.25 (dd, *J* = 12.5, 6.5 Hz, 3H), 7.12 (d, *J* = 7.6 Hz, 1H), 5.10 (dd, *J* = 7.7, 3.8 Hz, 1H), 3.67 (q, *J* = 7.7, 6.9 Hz, 2H), 3.41 (s, 6H), 3.18 (dd, *J* = 14.2, 3.8 Hz, 1H), 2.91 (q, *J* = 6.9 Hz, 2H), 2.88 – 2.80 (m, 1H), 2.62 (d, *J* = 9.8 Hz, 1H), 2.54 – 2.46 (m, 1H), 2.46 – 2.39 (m, 2H). ^{13}C{^{1}H} NMR (126 MHz, chloroform-d) δ 178.9, 171.2, 159.3, 141.6, 138.8, 138.8, 129.0, 128.6, 127.5, 126.5, 122.0, 102.4, 75.0, 56.7, 55.8, 46.7, 40.8, 35.5, 33.5, 29.7. HRMS (ESI) m/z calculated for $C_{22}H_{27}BrN_3O_4$ $[M+H]^+$: 476.1127; found $[M+H]^+$: 476.1128.

5t: 2-(5-bromopyridin-2-yl)-1-(2,2-dimethoxyethyl)-5-oxo-N-phenethylpyrrolidine-2-carboxamide.

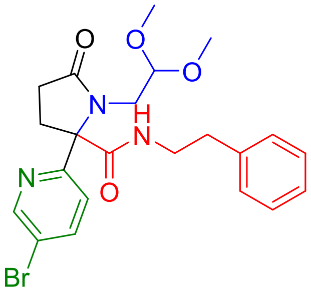

417 mg, 88% yield, yellow oil. mp 146 - 148 °C. ^{1}H NMR (500 MHz, chloroform-d) δ 8.58 (d, *J* = 2.5 Hz, 1H), 8.49 (t, *J* = 5.8 Hz, 1H), 7.71 (dd, *J* = 8.4, 2.4 Hz, 1H), 7.37 – 7.30 (m, 2H), 7.26 (d, *J* = 6.6 Hz, 3H), 7.10 (d, *J* = 8.5 Hz, 1H), 5.07 (dd, *J* = 7.8, 3.7 Hz, 1H), 3.67 (q, *J* = 6.1 Hz, 2H), 3.40 (s, 6H), 3.14 (dd, *J* = 14.1, 3.7 Hz, 1H), 2.91 (q, *J* = 7.6, 7.1 Hz, 2H), 2.83 – 2.72 (m, 1H), 2.65 – 2.53 (m, 1H), 2.53 – 2.39 (m, 3H). ^{13}C{^{1}H} NMR (126 MHz, chloroform-d) δ 179.1, 171.4, 156.9, 150.5, 139.0, 138.9, 129.0, 128.6, 126.5, 124.4, 120.0, 102.4, 74.9, 56.6, 55.7, 46.8, 40.7, 35.6, 33.7, 29.7. HRMS (ESI) m/z calculated for $C_{22}H_{27}BrN_3O_4$ $[M+H]^+$: 476.1124; found $[M+H]^+$: 476.1126.

5u: 1-(2,2-dimethoxyethyl)-5-oxo-N-phenethyl-2-(pyridin-4-yl)pyrrolidine-2-carboxamide.

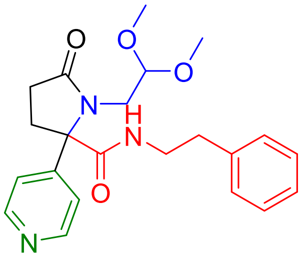

337 mg, 85% yield, yellow oil. mp 142 - 144 °C. ^{1}H NMR (500 MHz, chloroform-d) δ 9.37 (s, 1H), 8.60 (s, 1H), 8.33 (t, *J* = 5.7 Hz, 1H), 7.92 (s, 1H), 7.32 (t, *J* = 7.2 Hz, 2H), 7.26 (dd, *J* = 12.5, 7.0 Hz, 3H), 7.18 (d, *J* = 6.1 Hz, 1H), 5.07 (dd, *J* = 7.4, 3.8 Hz, 1H), 3.69 (dt, *J* = 11.2, 6.1 Hz, 2H), 3.39 (s, 3H), 3.37 (s, 3H), 3.20 (dd, *J* = 14.3, 3.8 Hz, 1H), 2.91 (p, *J* = 7.5 Hz, 2H), 2.87 – 2.73 (m, 1H), 2.50 – 2.38 (m, 4H). ^{13}C{^{1}H} NMR (126 MHz, chloroform-d) δ

177.4, 170.1, 150.4, 148.6, 145.6, 138.7, 129.0, 128.6, 126.6, 123.2, 101.9, 73.8, 56.6, 55.7, 55.7, 46.5, 40.9, 35.6, 35.5, 29.4. HRMS (ESI) m/z calculated for $C_{22}H_{28}N_3O_4$ $[M+H]^+$: 398.2011; found $[M+H]^+$: 398.2016.

5v: 1-(2,2-dimethoxyethyl)-N-(4-methoxyphenethyl)-5-oxo-2-(pyridin-2-yl)pyrrolidine-2-carboxamide.

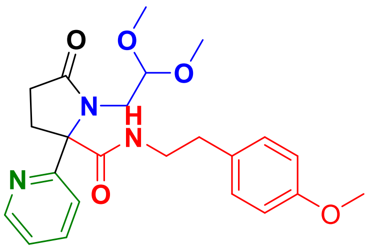

389 mg, 91% yield, yellow oil. mp 143 - 145 °C. ^{1}H NMR (500 MHz, chloroform-d) δ 8.63 (t, *J* = 5.5 Hz, 1H), 8.56 – 8.51 (m, 1H), 7.64 – 7.57 (m, 1H), 7.24 – 7.14 (m, 4H), 6.90 – 6.81 (m, 2H), 5.05 (dd, *J* = 7.6, 3.9 Hz, 1H), 3.81 (s, 3H), 3.71 – 3.57 (m, 2H), 3.41 (s, 3H), 3.38 (s, 3H), 3.15 (dd, *J* = 14.2, 3.8 Hz, 1H), 2.92 – 2.74 (m, 3H), 2.68 – 2.52 (m, 2H), 2.54 – 2.44 (m, 2H). ^{13}C{^{1}H} NMR (126 MHz, chloroform-d) δ 179.1, 171.7, 158.4, 158.3, 149.3, 136.6, 131.0, 129.9, 122.9, 122.8, 113.9, 102.4, 75.2, 56.4, 55.5, 55.3, 46.6, 41.0, 34.7, 33.8, 29.9. HRMS (ESI) m/z calculated for $C_{23}H_{30}N_3O_5$ $[M+H]^+$: 428.2143; found $[M+H]^+$: 428.2147.

5w: 1-(2,2-dimethoxyethyl)-2-(6-methylpyridin-2-yl)-5-oxo-N-phenethylpyrrolidine-2-carboxamide.

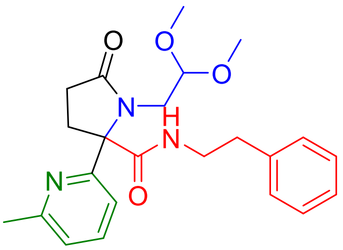

378 mg, 92% yield, yellow oil. mp 143 - 145 °C. ^{1}H NMR (500 MHz, chloroform-d) δ 8.73 (t, *J* = 5.7 Hz, 1H), 7.50 (t, *J* = 7.8 Hz, 1H), 7.32 (t, *J* = 7.8 Hz, 2H), 7.29 – 7.22 (m, 3H), 7.03 (t, *J* = 8.3 Hz, 2H), 5.03 (dd, *J* = 6.9, 3.3 Hz, 1H), 3.75 – 3.61 (m, 2H), 3.38 (s, 3H), 3.37 (s, 3H), 3.17 (dd, *J* = 14.2, 3.9 Hz, 1H), 2.92 (t, *J* = 7.1 Hz, 2H), 2.87 – 2.75 (m, 1H), 2.66 – 2.56 (m, 2H), 2.53 – 2.42 (m, 5H). ^{13}C{^{1}H} NMR (126 MHz, chloroform-d) δ 179.2, 171.9, 158.1, 157.6, 139.0, 136.8, 128.9, 128.5, 126.4, 122.3, 119.6, 102.4, 75.1, 56.3, 55.3, 46.5, 40.8, 35.6, 33.7, 30.0, 24.5. HRMS (ESI) m/z calculated for $C_{23}H_{30}N_3O_4$ $[M+H]^+$: 412.2231; found $[M+H]^+$: 412.2235.

5x: 1-(2,2-dimethoxyethyl)-N-(3,4-dimethoxyphenethyl)-5-oxo-2-(pyridin-2-yl)pyrrolidine-2-carboxamide.

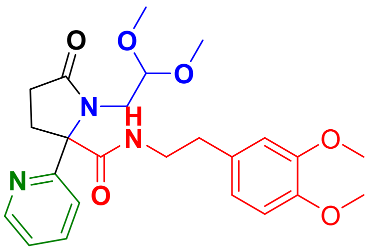

448 mg, 98% yield, yellow oil. mp 142 - 145 °C. ^{1}H NMR (500 MHz, chloroform-d) δ 8.64 (t, *J* = 5.7 Hz, 1H), 8.53 (d, *J* = 6.0 Hz, 1H), 7.60 (tt, *J* = 7.9, 1.7 Hz, 1H), 7.21 – 7.17 (m, 1H), 7.15 (d, *J* = 8.0 Hz, 1H), 6.80 (d, *J* = 3.5 Hz, 3H), 5.05 (dd, *J* = 6.9, 3.1 Hz, 1H), 3.88 (s, 3H), 3.86 (s, 3H), 3.75 – 3.67 (m, 1H), 3.66 – 3.58 (m, 1H), 3.43 (s, 3H), 3.39 (s, 3H), 3.14 (dd, *J* = 14.3, 3.9 Hz, 1H), 2.92 – 2.74 (m, 3H), 2.66 – 2.52 (m, 2H), 2.54 – 2.42 (m, 2H). ^{13}C{^{1}H} NMR (126 MHz, chloroform-d) δ 179.0, 171.7, 158.4, 149.3, 149.0, 147.7, 136.6, 131.5, 122.9, 121.0, 112.0,

111.2, 102.5, 75.2, 56.4, 55.94, 55.9, 55.6, 46.6, 40.8, 35.3, 33.7, 29.9. HRMS (ESI) m/z calculated for $C_{24}H_{32}N_3O_6$ $[M+H]^+$: 458.2213; found $[M+H]^+$: 458.2217.

5y: 1-(2,2-dimethoxyethyl)-5-oxo-N-phenethyl-2-(pyrimidin-4-yl)pyrrolidine-2-carboxamide.

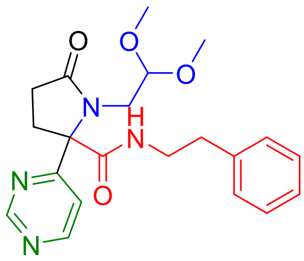

259 mg, 65% yield, yellow oil. mp 144 - 147 °C. ^{1}H NMR (500 MHz, chloroform-d) δ 9.15 (d, J = 1.4 Hz, 1H), 8.66 (d, J = 5.4 Hz, 1H), 8.54 (t, J = 5.7 Hz, 1H), 7.35 – 7.31 (m, 1H), 7.29 – 7.25 (m, 4H), 5.09 (dd, J = 7.8, 3.5 Hz, 1H), 3.74 – 3.64 (m, 2H), 3.42 (s, 3H), 3.41 (s, 3H), 3.21 (dd, J = 14.1, 3.5 Hz, 1H), 2.99 – 2.90 (m, 2H), 2.75 – 2.68 (m, 1H), 2.64 (dd, J = 16.3, 6.2 Hz, 1H), 2.55 – 2.47 (m, 2H), 2.46 – 2.39 (m, 1H). $^{13}C\{^{1}H\}$ NMR (126 MHz, chloroform-d) δ 179.2, 170.4, 166.9, 158.6, 157.5, 138.7, 129.0, 128.6, 126.6, 120.2, 102.4, 74.5, 56.8, 56.0, 47.1, 40.7, 35.5, 33.5, 29.5. HRMS (ESI) m/z calculated for $C_{21}H_{27}N_4O_4$ $[M+H]^+$: 399.2013; found $[M+H]^+$: 399.2018

^{1}H NMR spectrum of **1a** (500 MHz, $CDCl_3$)

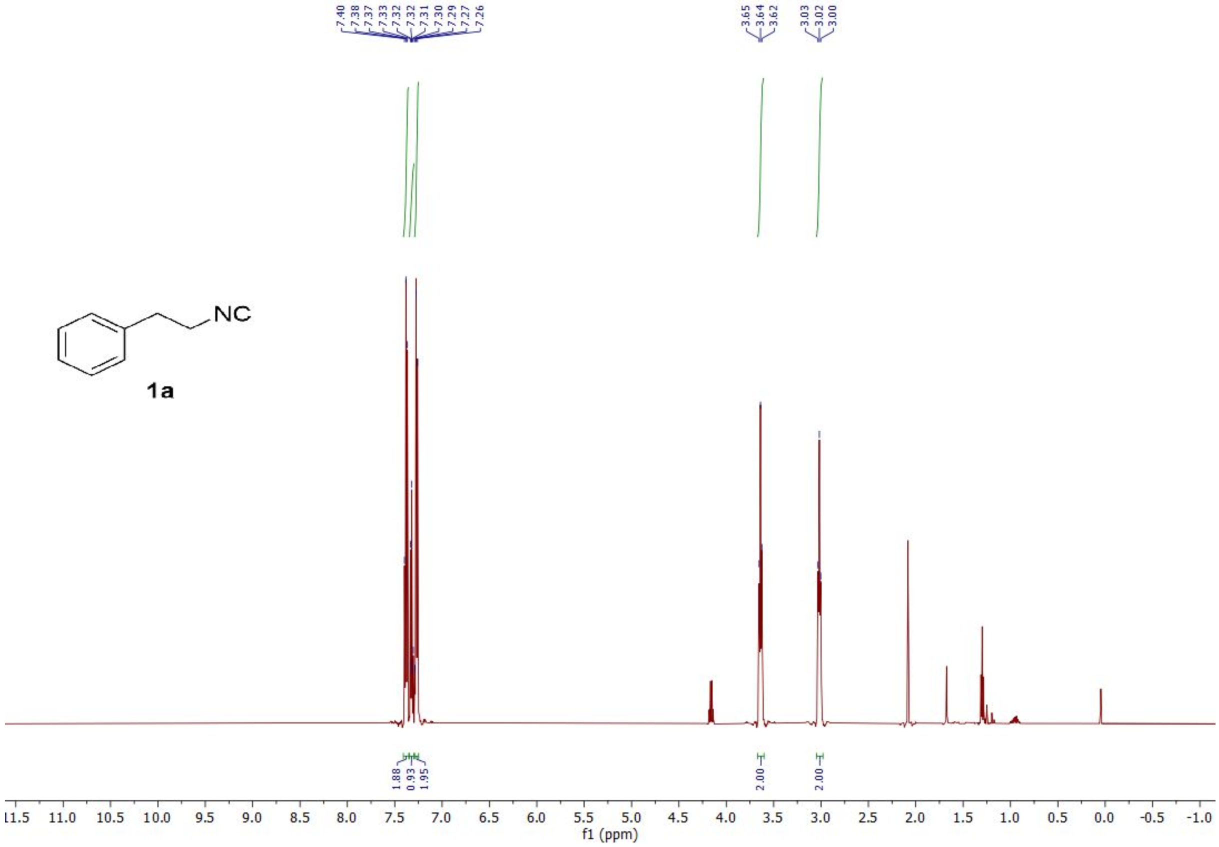

$^{13}C\{^{1}H\}$ NMR spectrum of **1a** (126 MHz, $CDCl_3$)

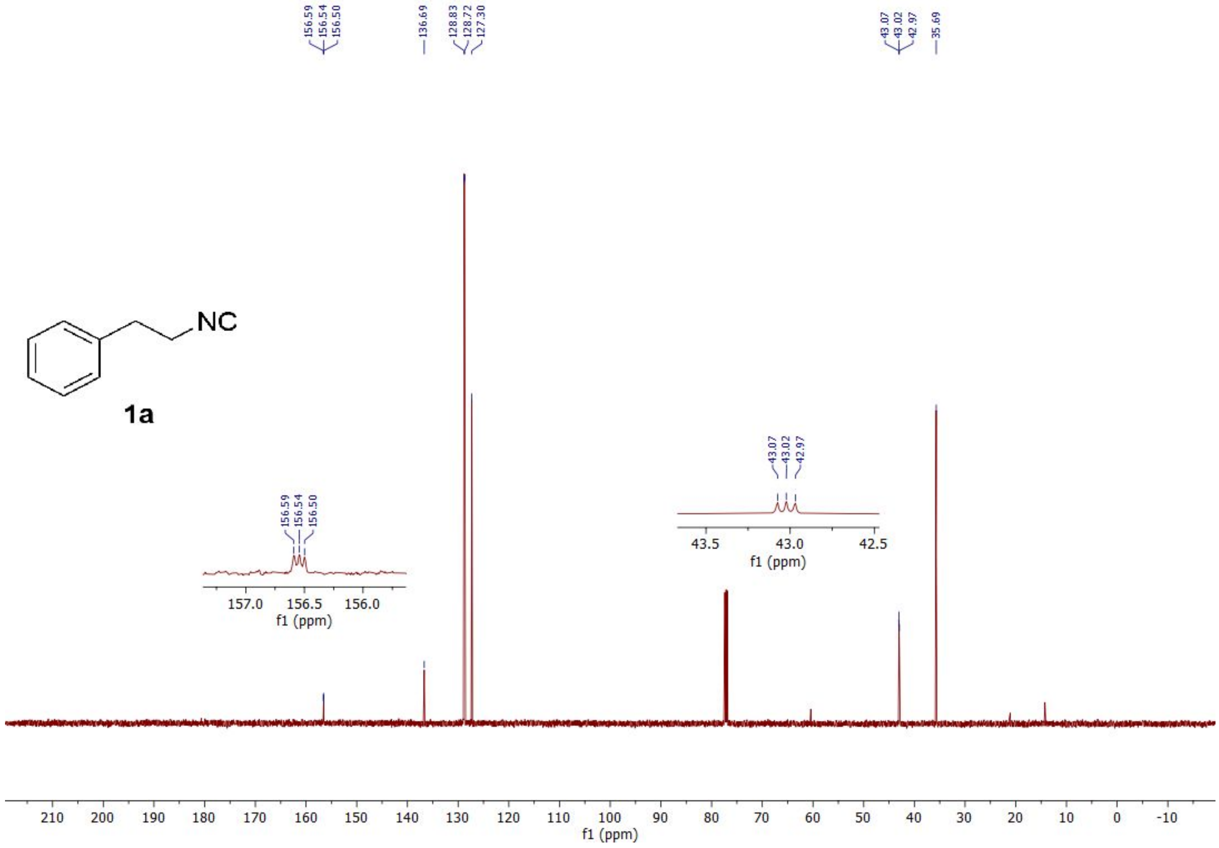

^{1}H NMR spectrum of **1b** (500 MHz, $CDCl_3$)

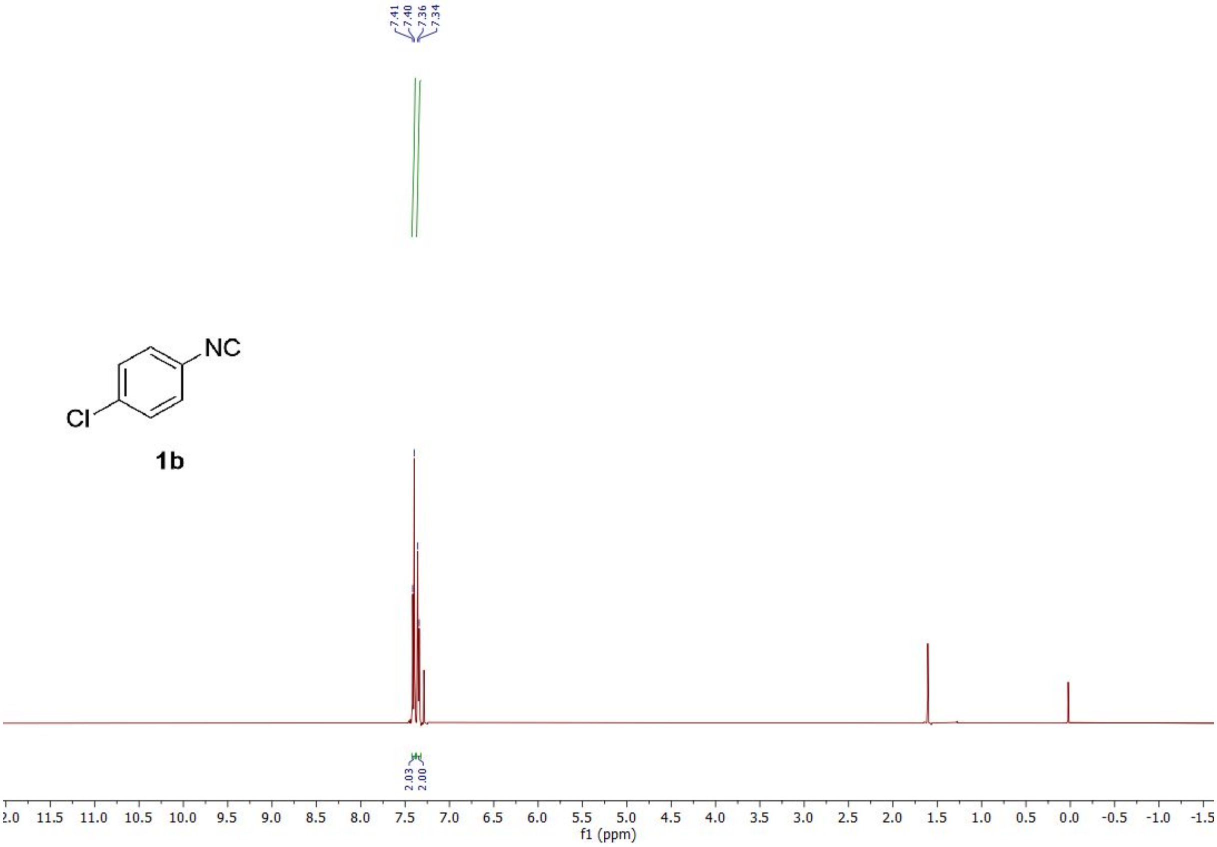

$^{13}C\{^{1}H\}$ NMR spectrum of **1b** (126 MHz, $CDCl_3$)

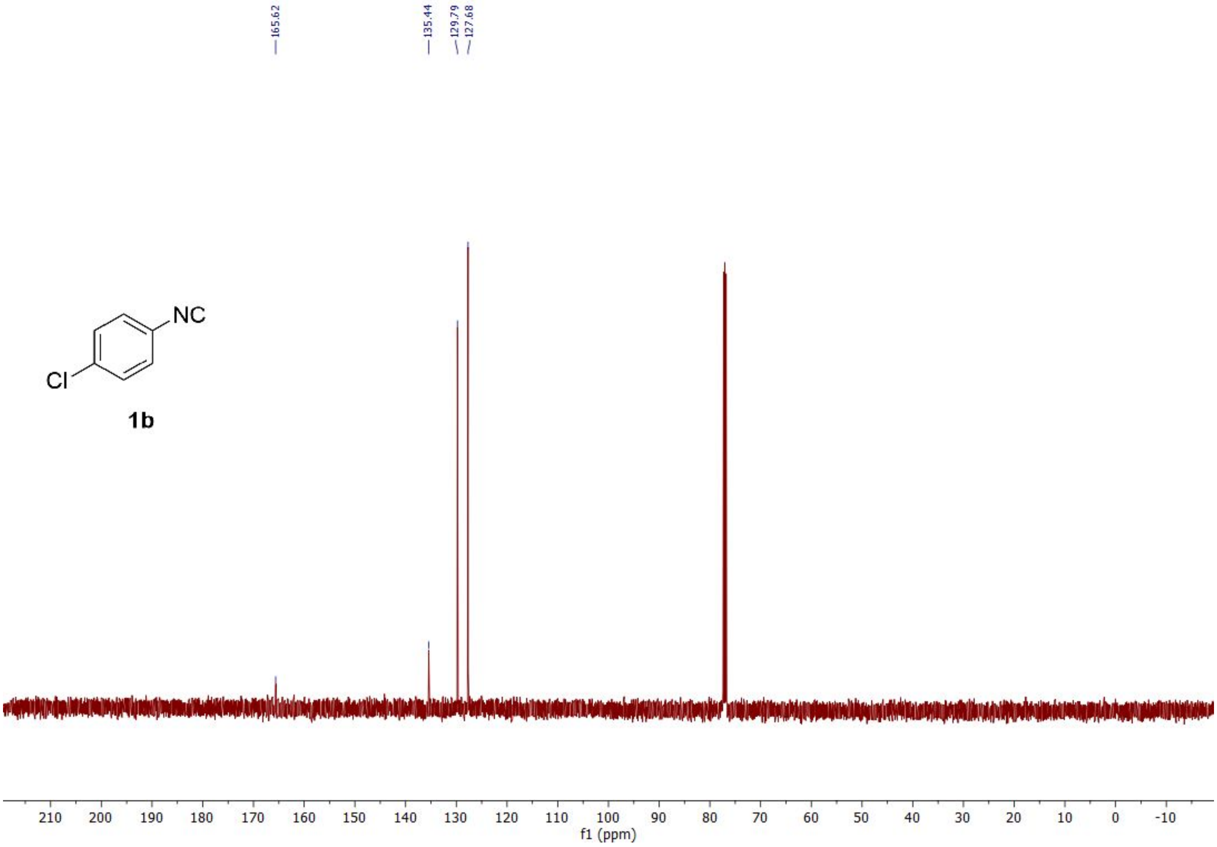

^{1}H NMR spectrum of **1c** (500 MHz, $CDCl_3$)

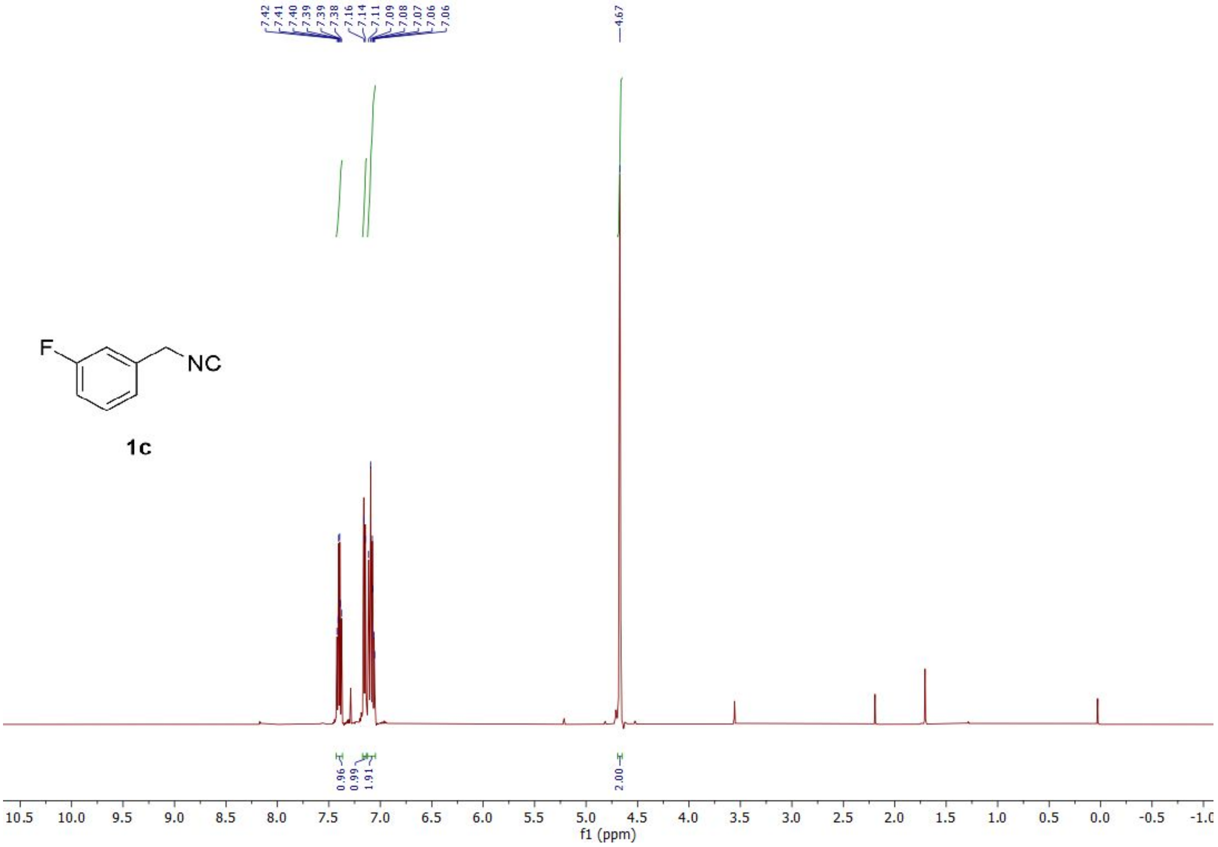

^{13}C{^{1}H} NMR spectrum of **1c** (126 MHz, $CDCl_3$)

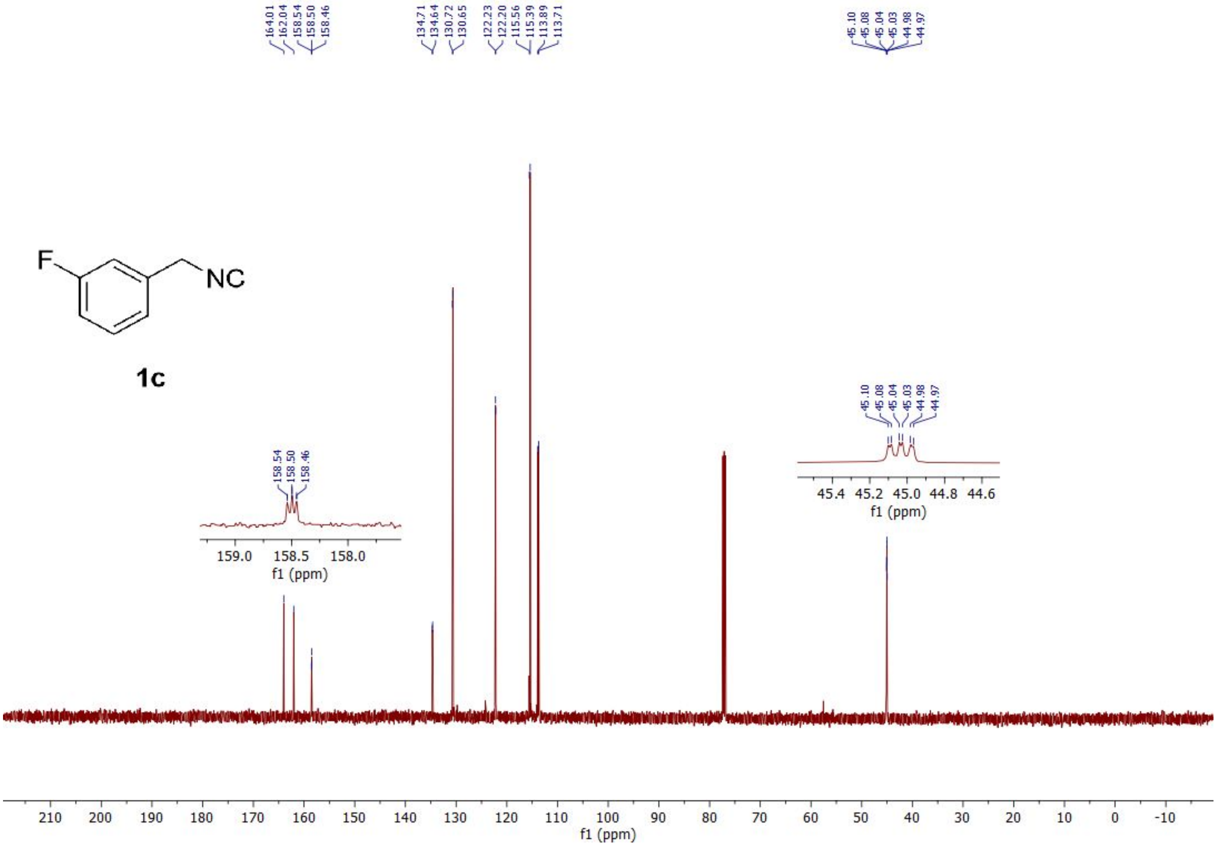

^{1}H NMR spectrum of **1d** (500 MHz, $CDCl_3$)

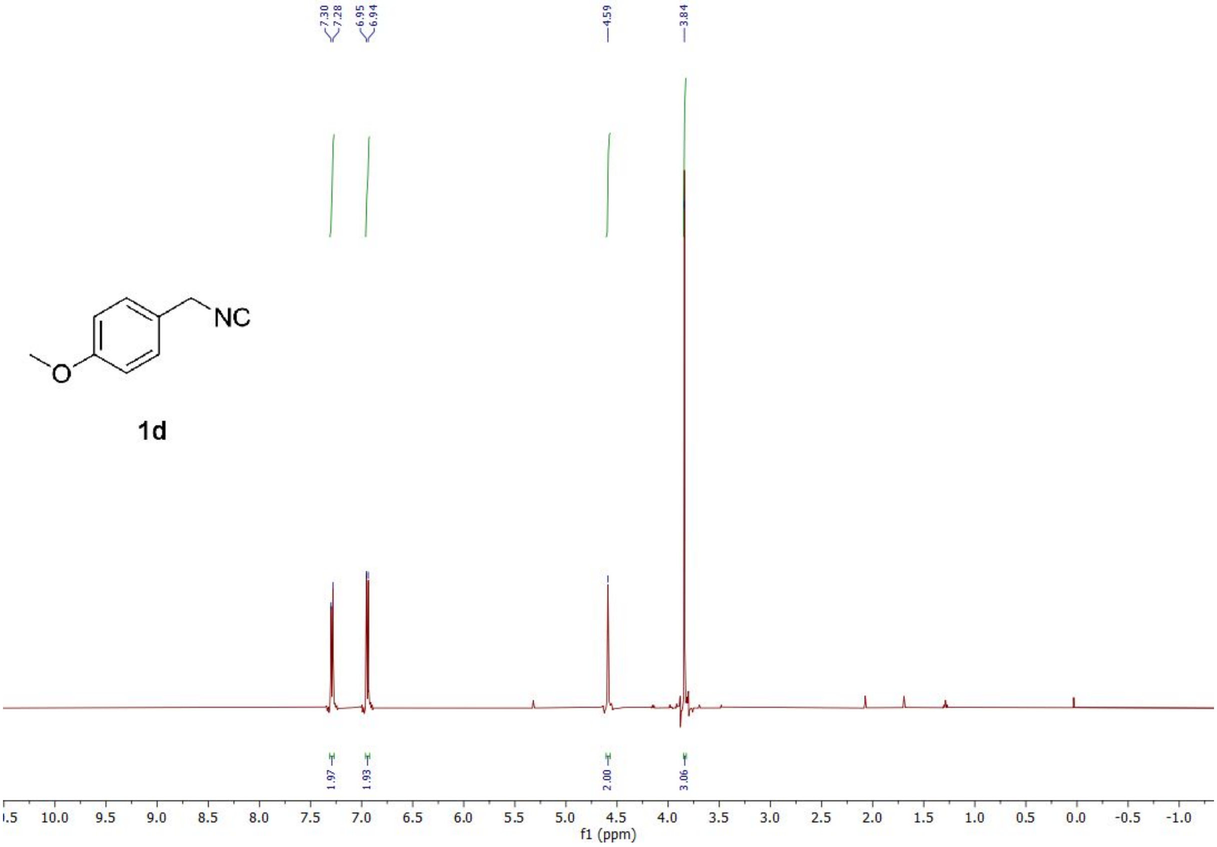

$^{13}C\{^{1}H\}$ NMR spectrum of **1d** (126 MHz, $CDCl_3$)

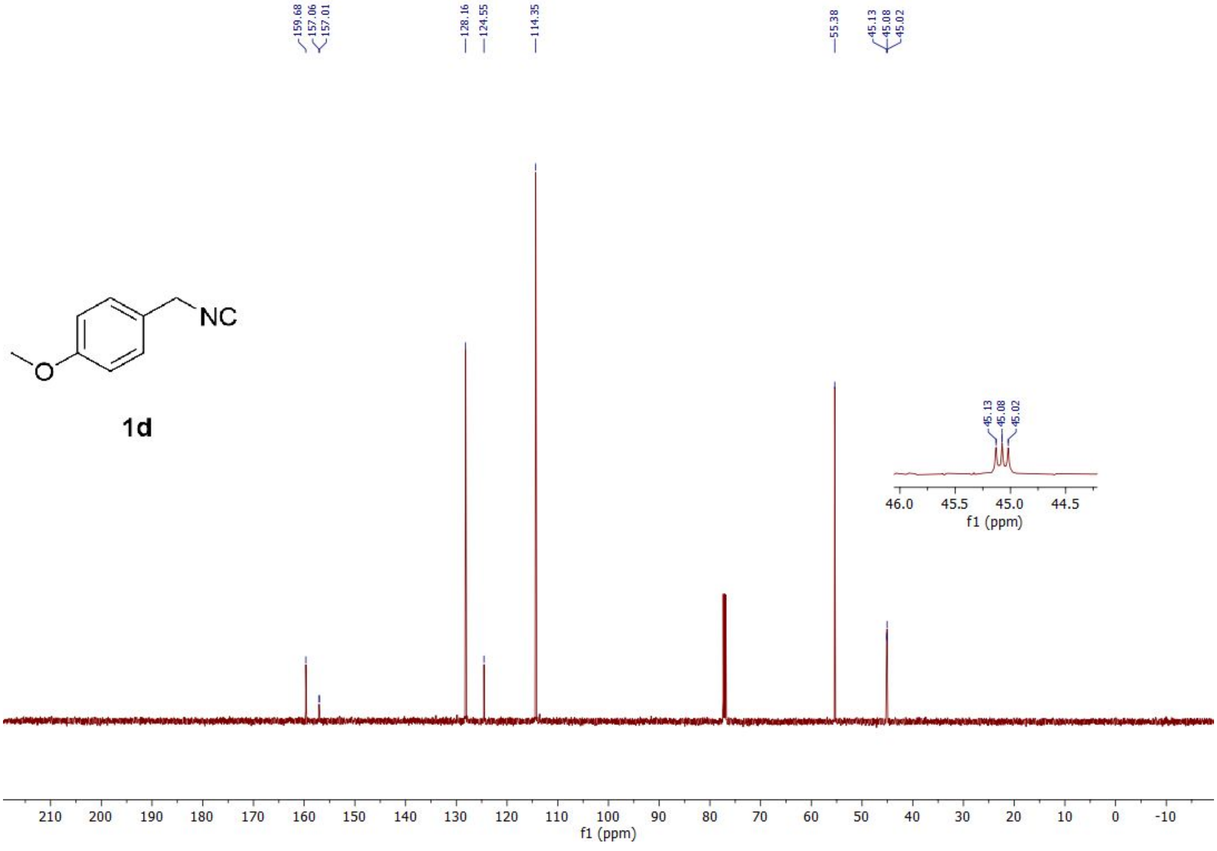

^{1}H NMR spectrum of **1e** (500 MHz, $CDCl_3$)

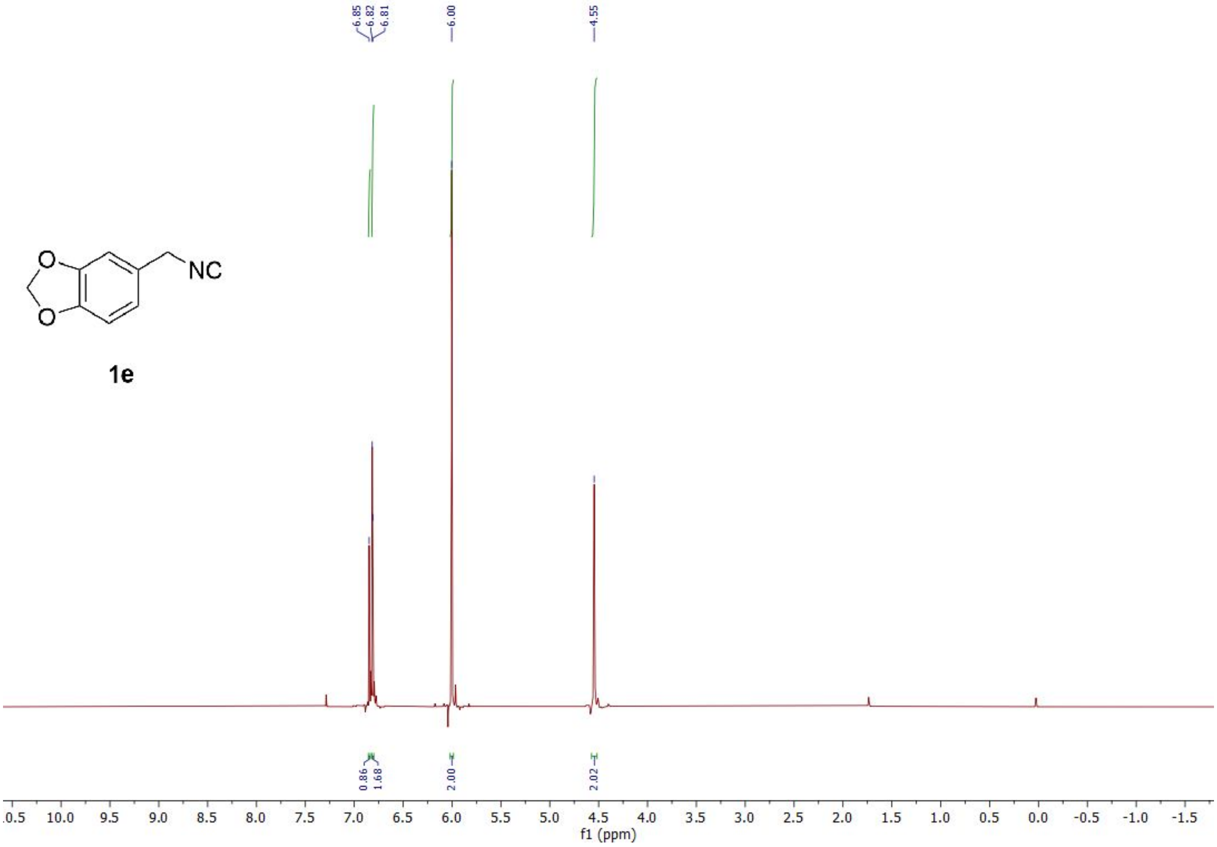

$^{13}C\{^{1}H\}$ NMR spectrum of **1e** (126 MHz, $CDCl_3$)

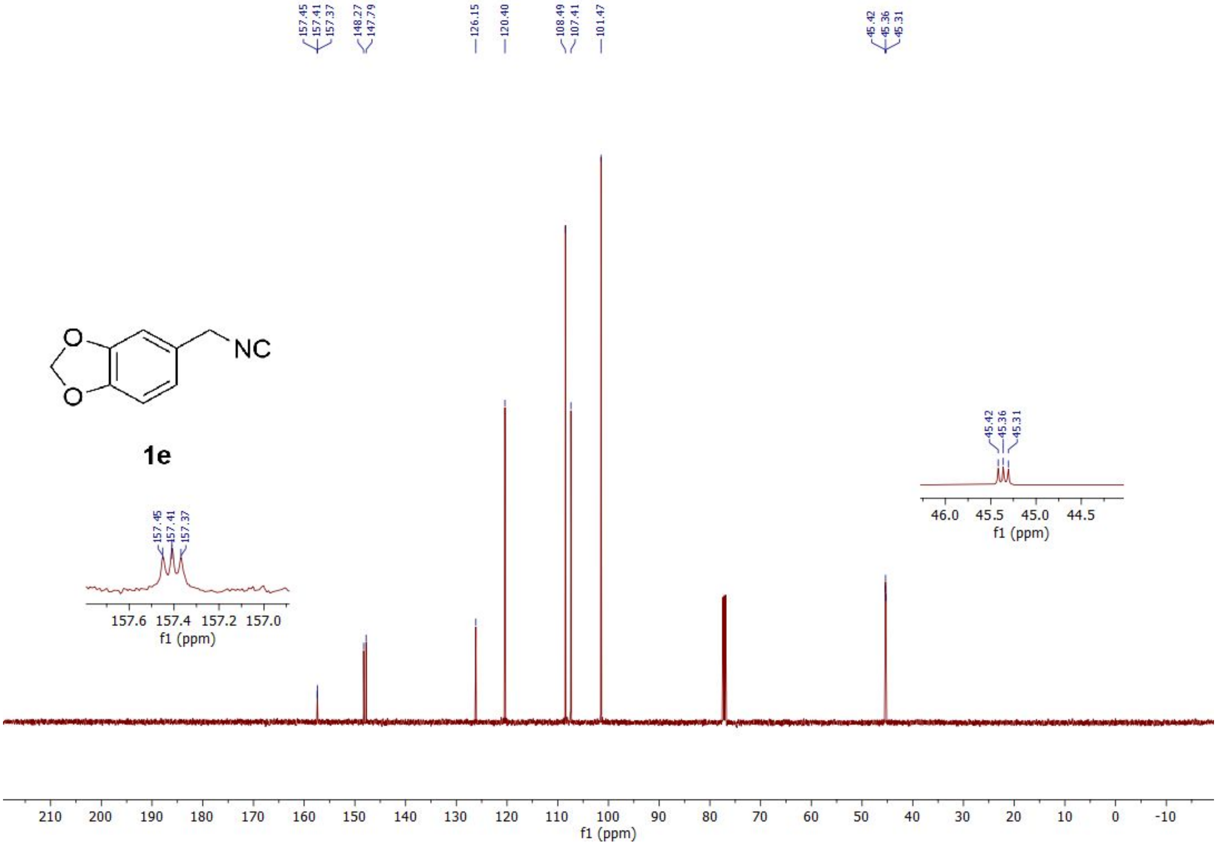

^{1}H NMR spectrum of **1f** (500 MHz, $CDCl_3$)

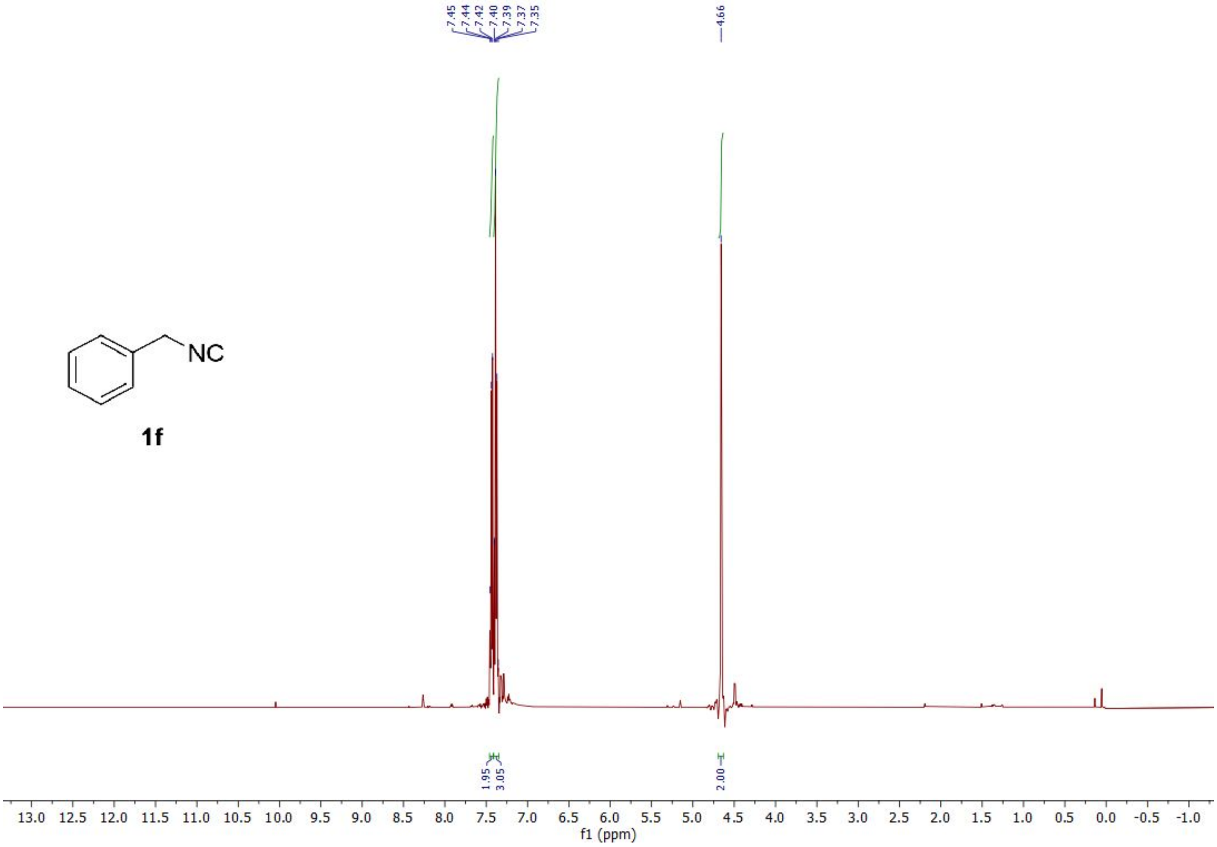

$^{13}C\{^{1}H\}$ NMR spectrum of **1f** (126 MHz, $CDCl_3$)

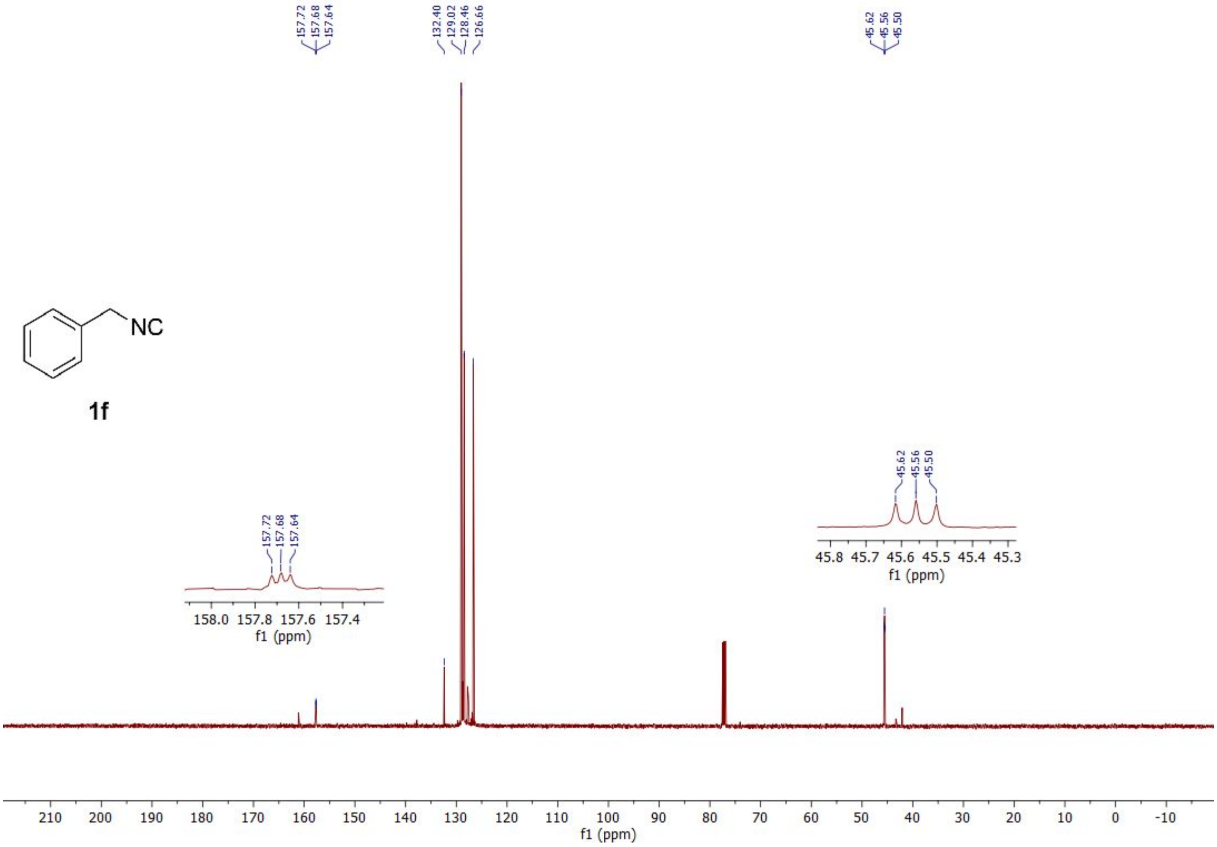

^{1}H NMR spectrum of **1g** (500 MHz, $CDCl_3$)

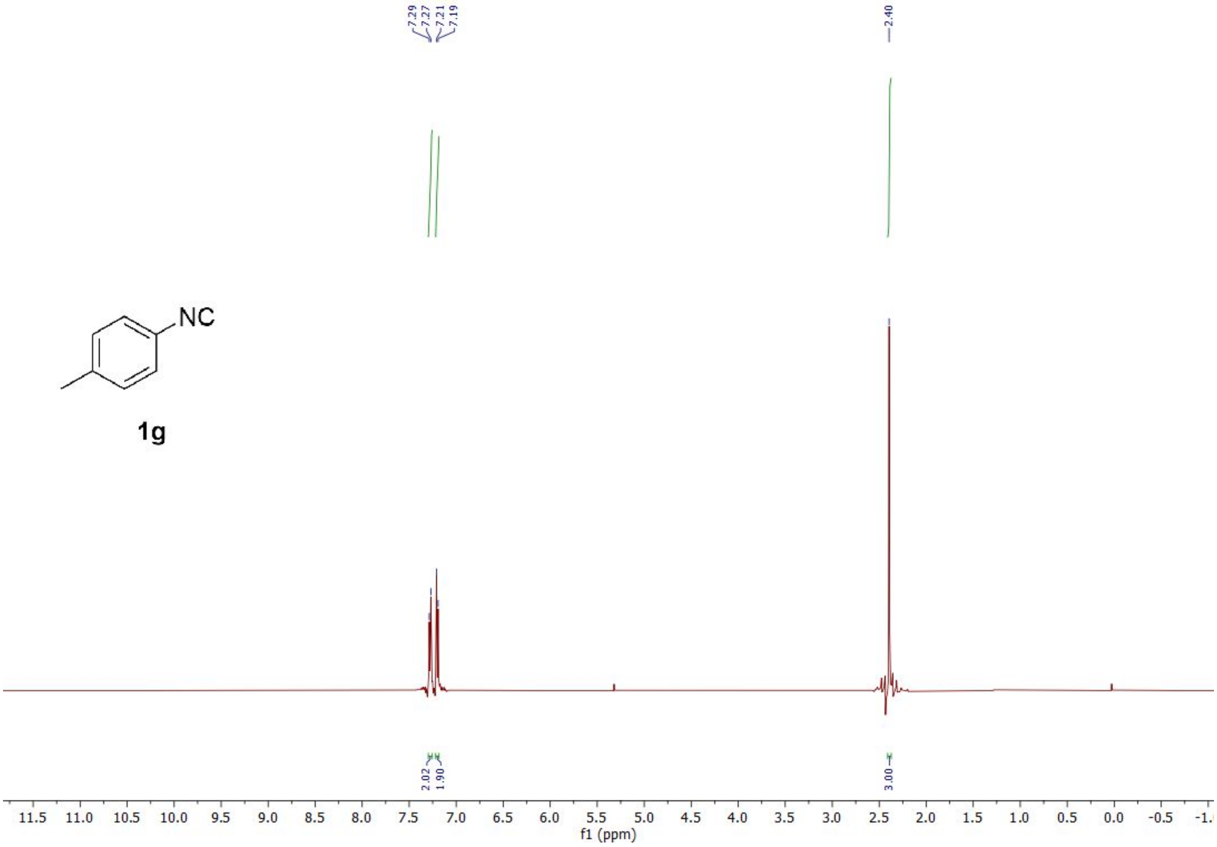

^{13}C{^{1}H} NMR spectrum of **1g** (126 MHz, $CDCl_3$)

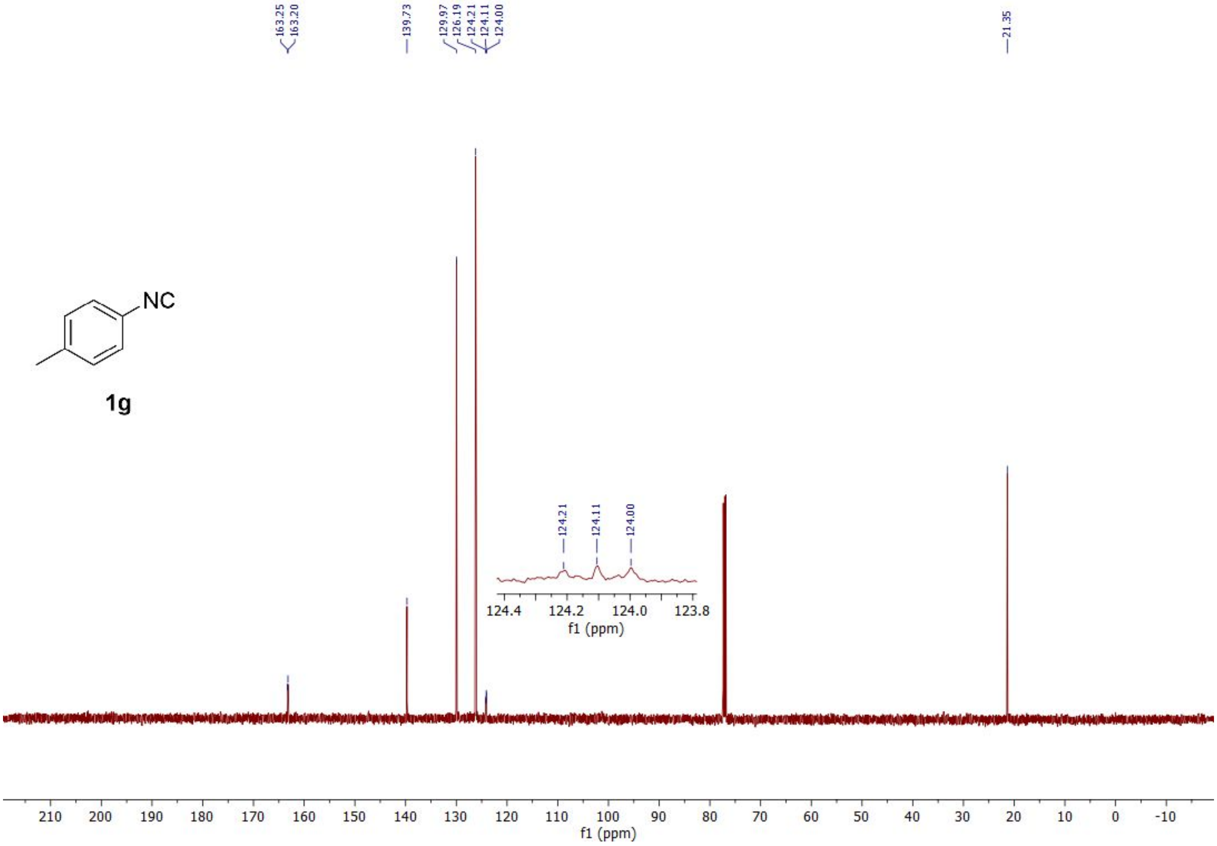

^{1}H NMR spectrum of **1h** (500 MHz, $CDCl_3$)

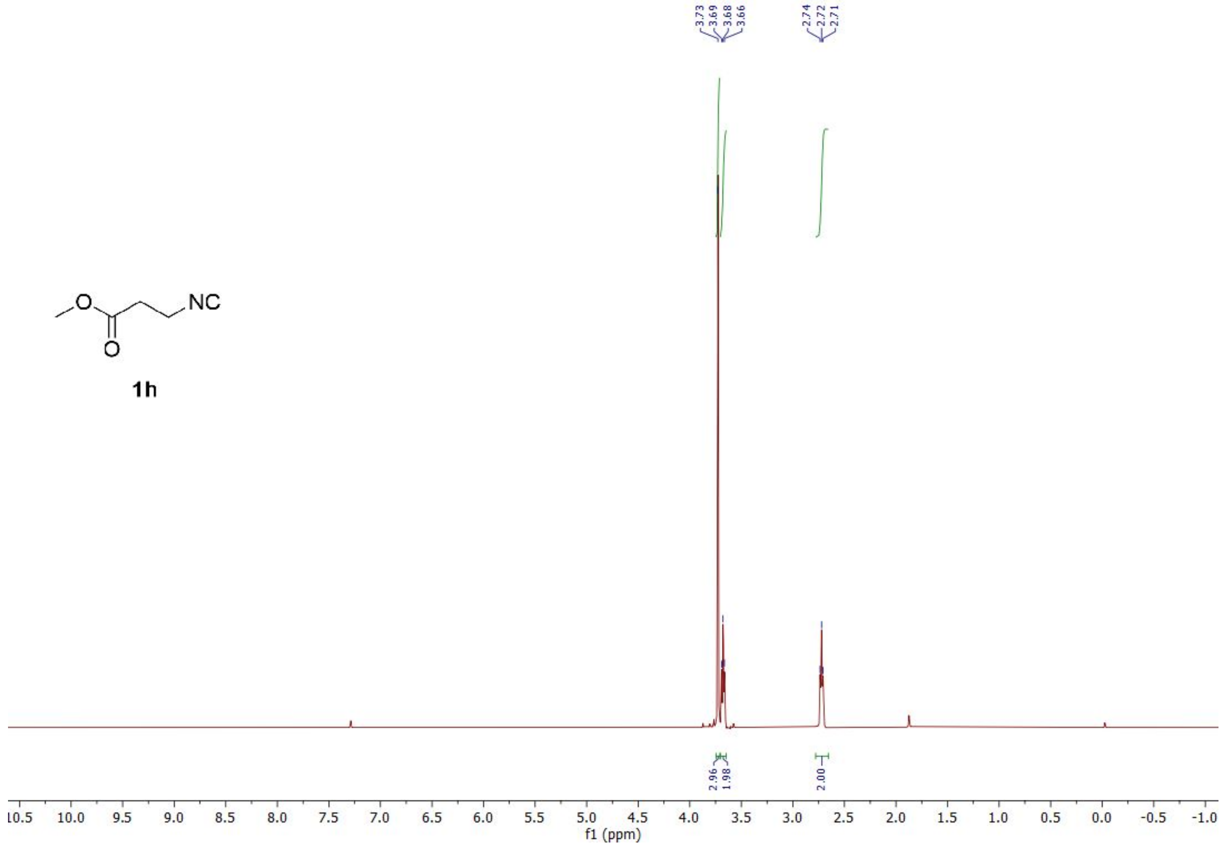

$^{13}C\{^{1}H\}$ NMR spectrum of **1h** (126 MHz, $CDCl_3$)

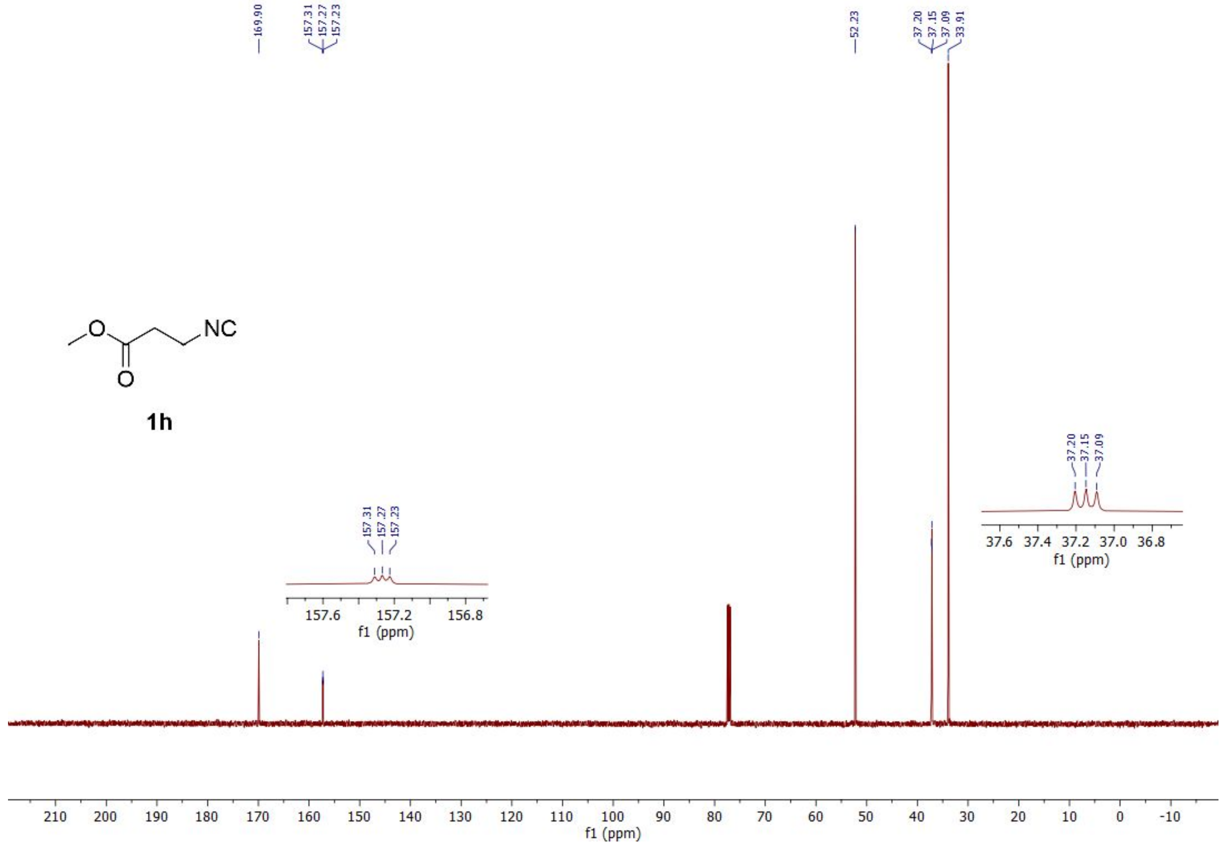

^{1}H NMR spectrum of **1i** (500 MHz, $CDCl_3$)

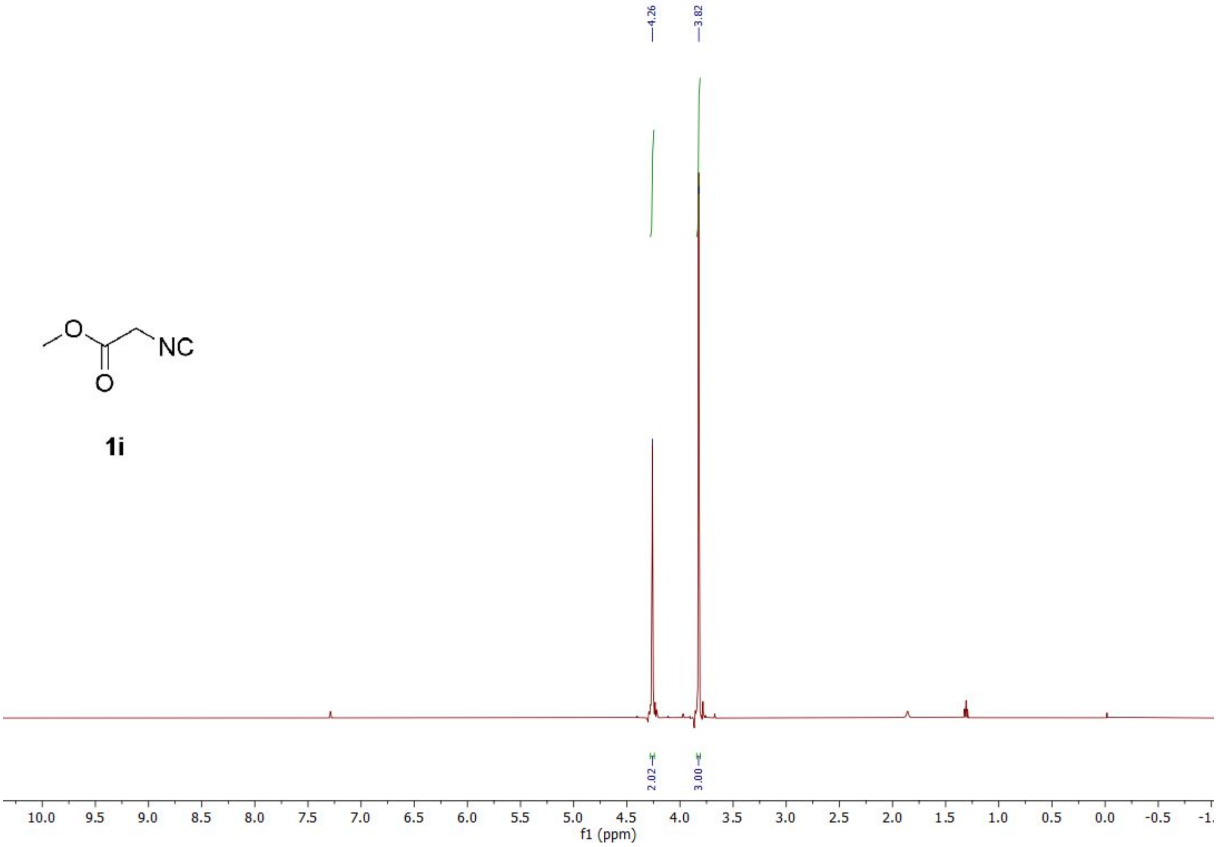

^{13}C{^{1}H} NMR spectrum of **1i** (126 MHz, $CDCl_3$)

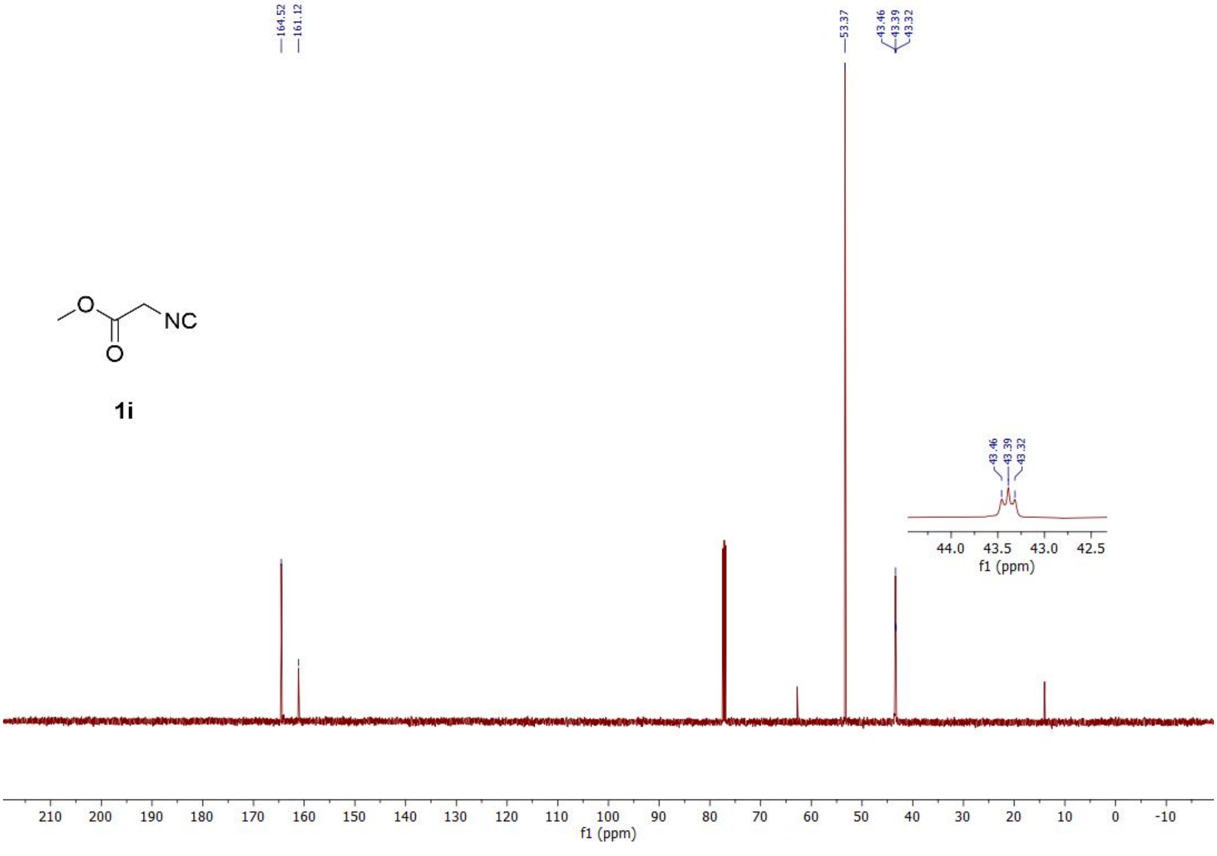

^{1}H NMR spectrum of **1j** (500 MHz, $CDCl_3$)

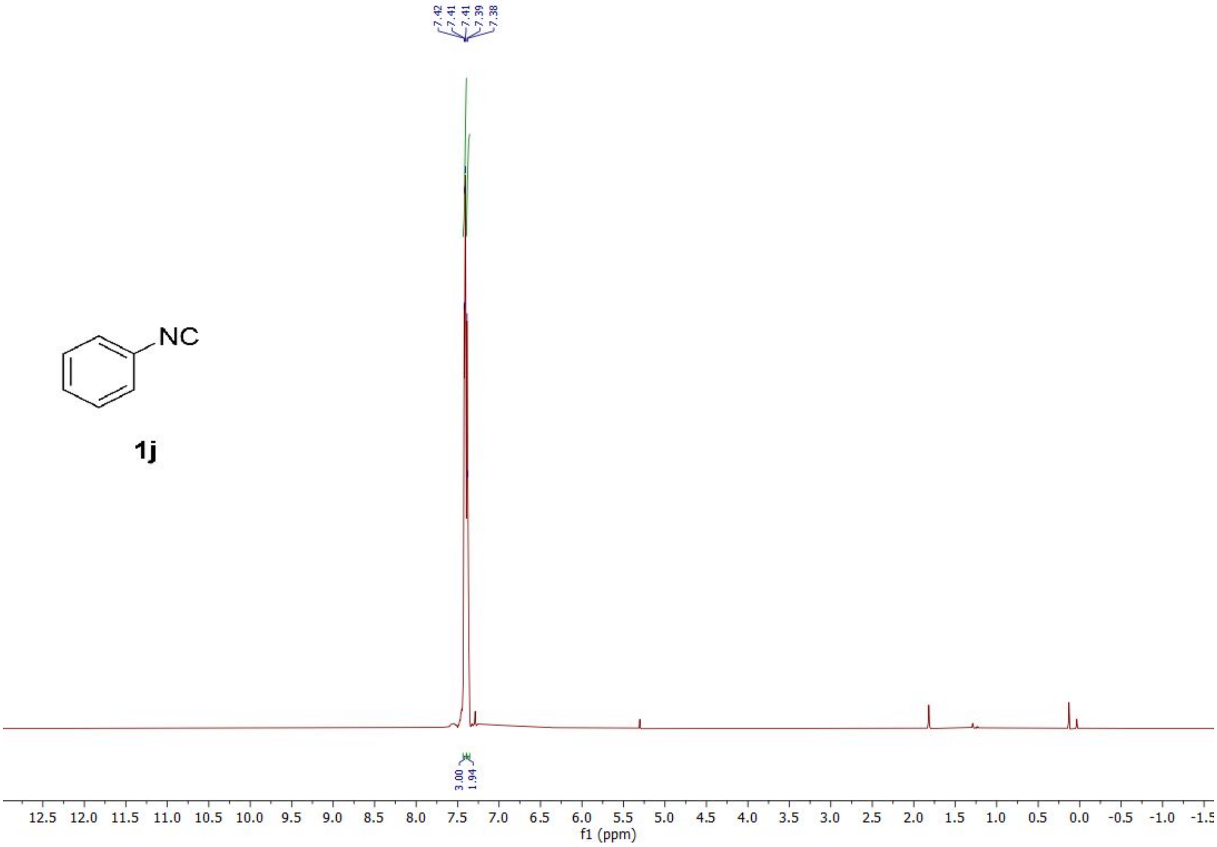

$^{13}C\{^{1}H\}$ NMR spectrum of **1j** (126 MHz, $CDCl_3$)

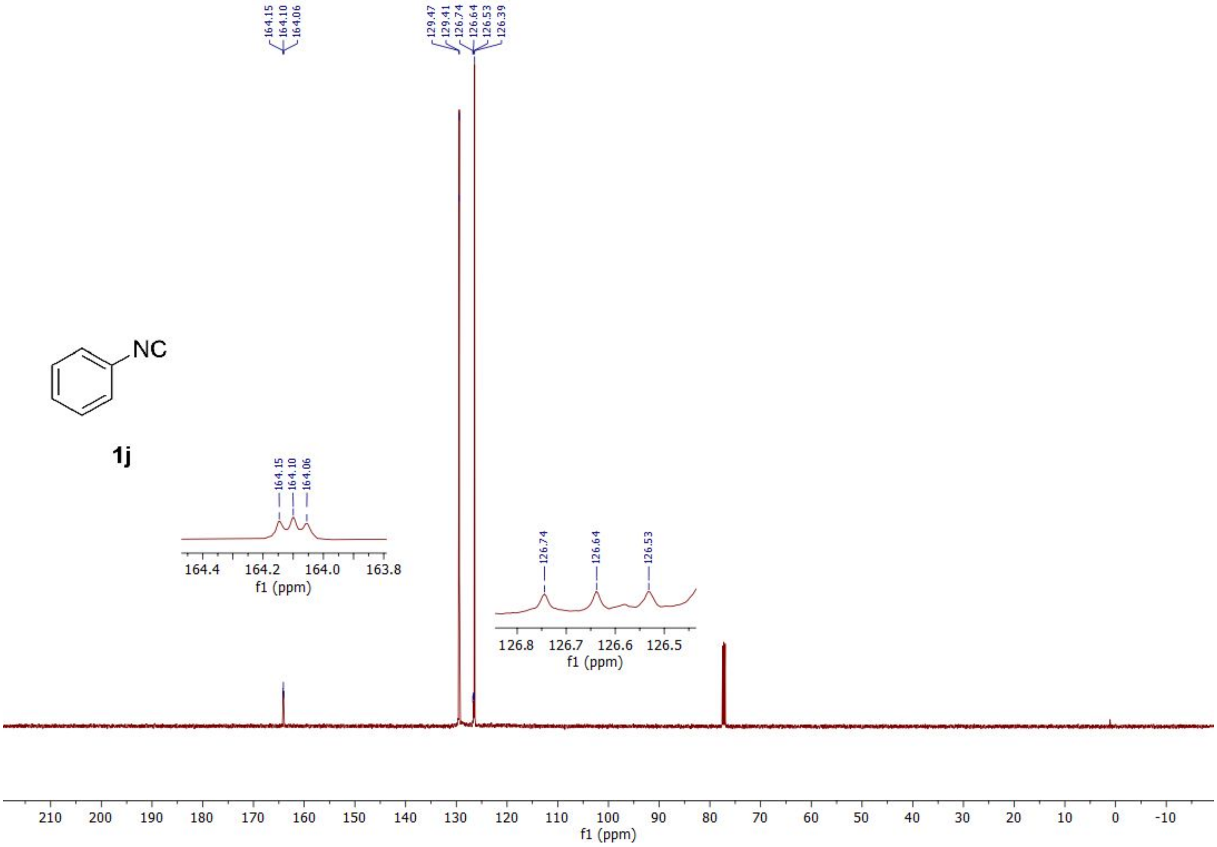

^{1}H NMR spectrum of **1k** (500 MHz, $CDCl_3$)

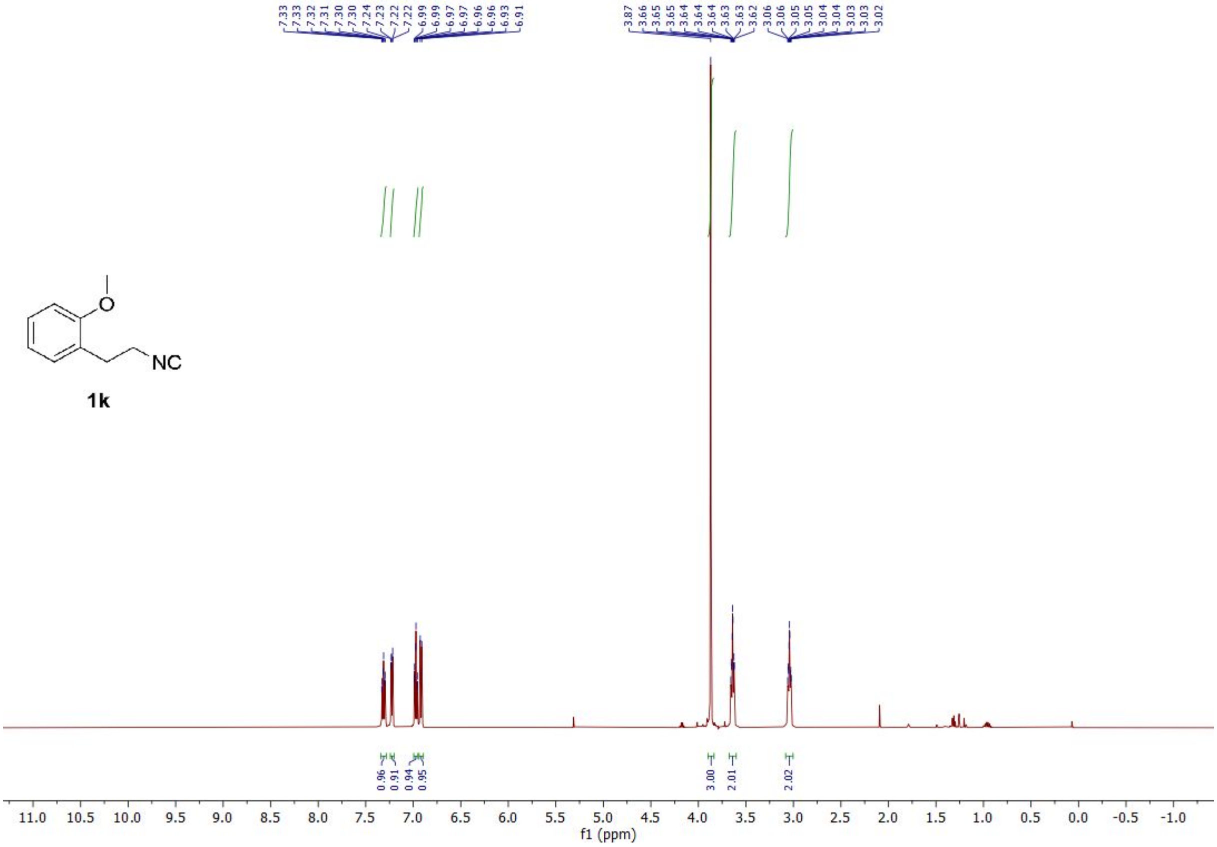

$^{13}C\{^{1}H\}$ NMR spectrum of **1k** (126 MHz, $CDCl_3$)

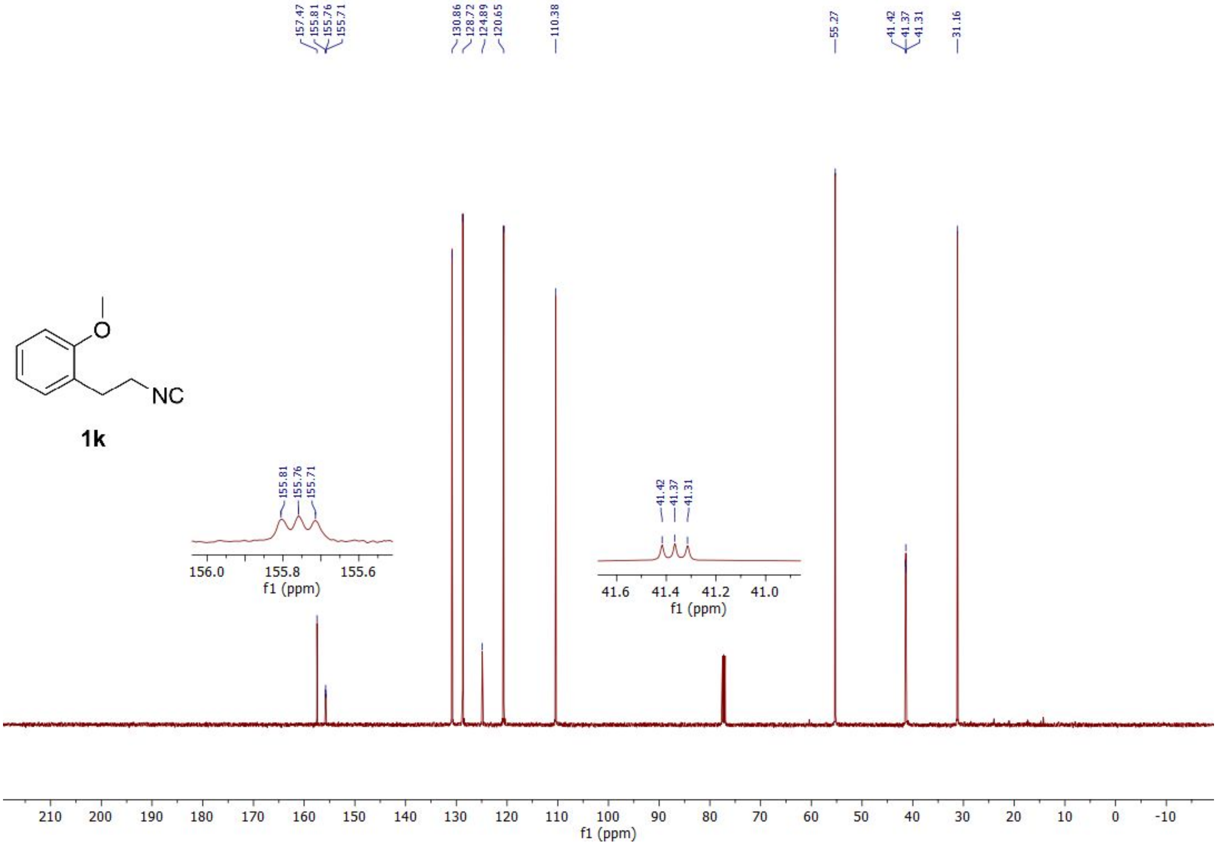

^{1}H NMR spectrum of **1l** (500 MHz, $CDCl_3$)

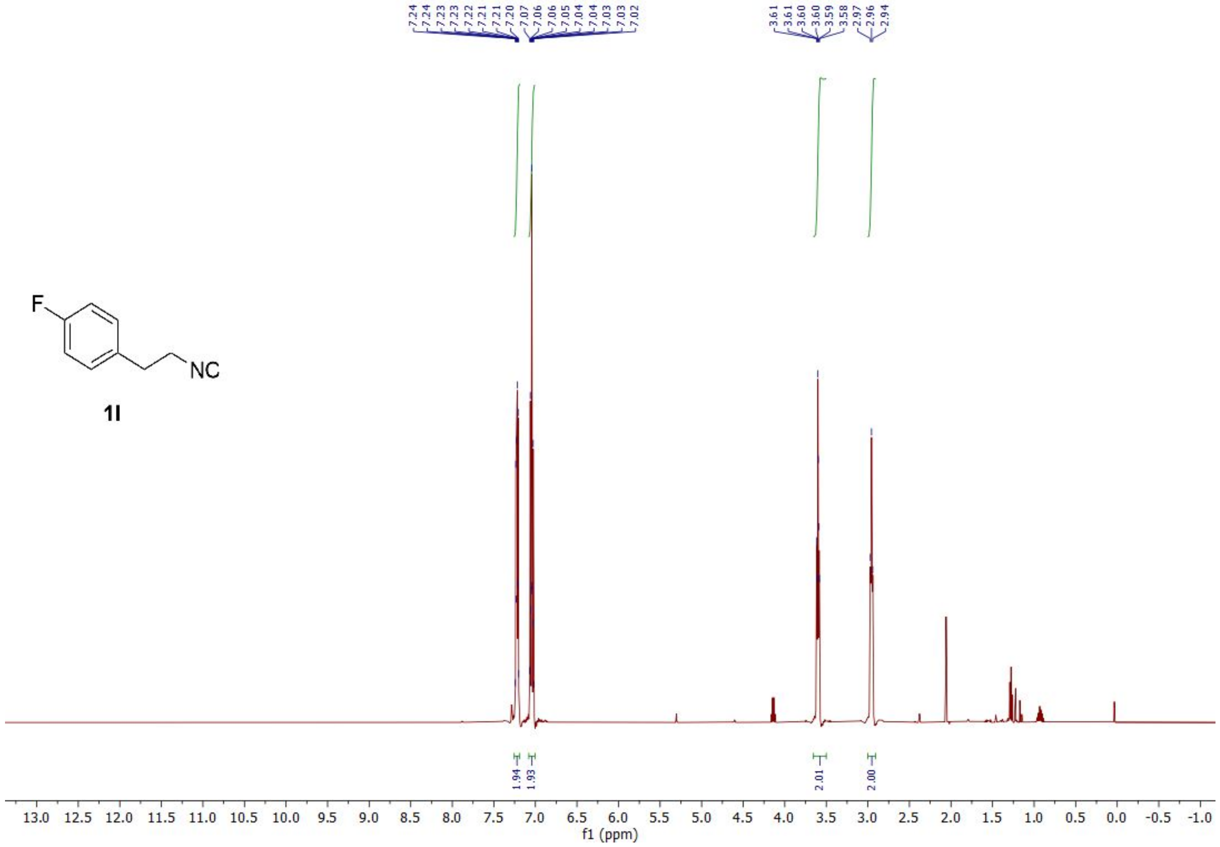

^{13}C{^{1}H} NMR spectrum of **1l** (126 MHz, $CDCl_3$)

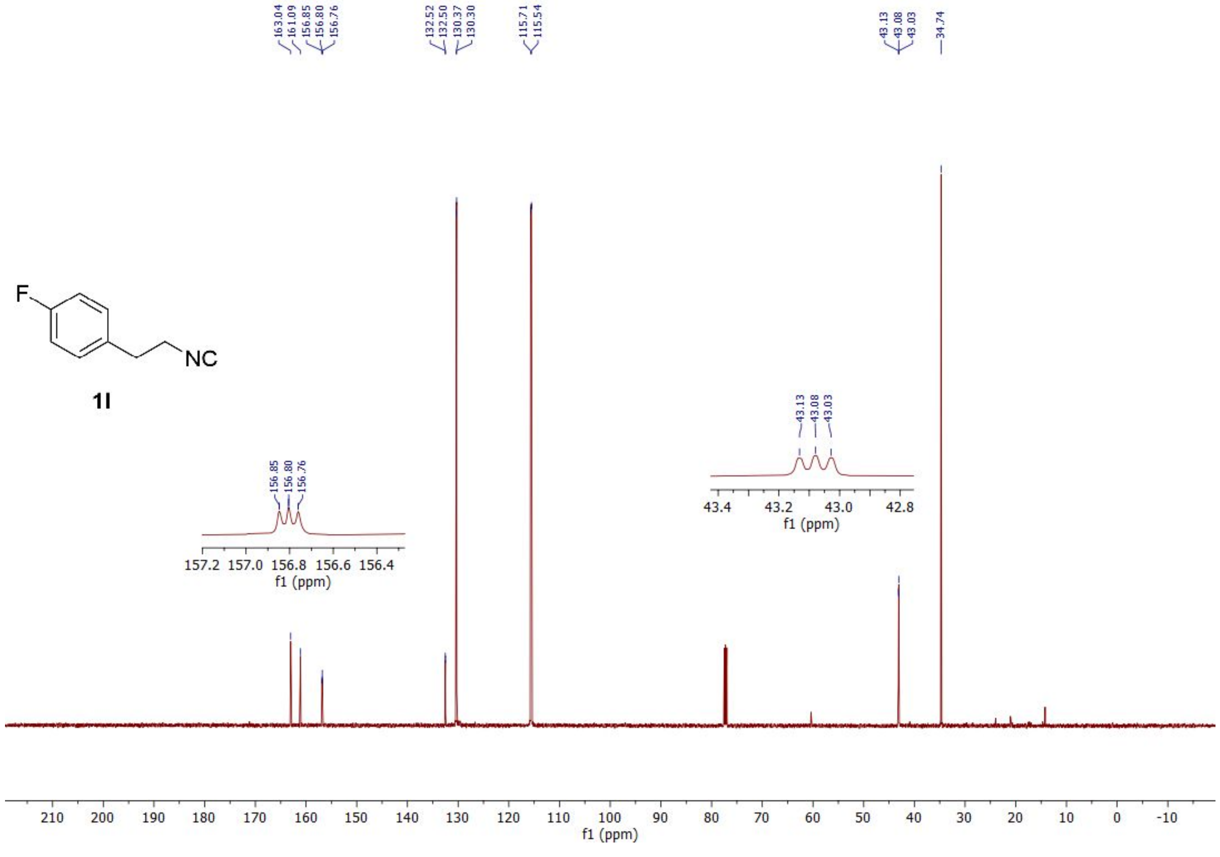

^{1}H NMR spectrum of **1m** (500 MHz, $CDCl_3$)

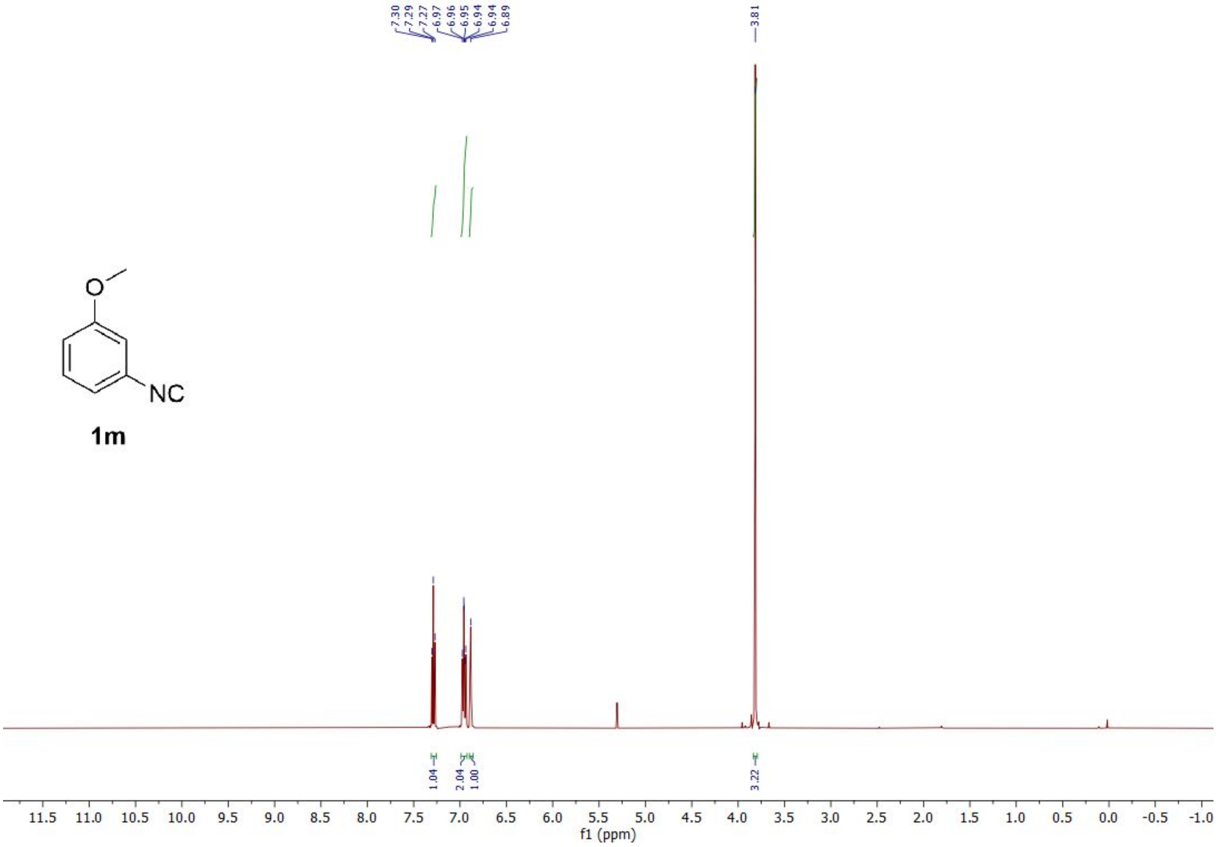

$^{13}C\{^{1}H\}$ NMR spectrum of **1m** (126 MHz, $CDCl_3$)

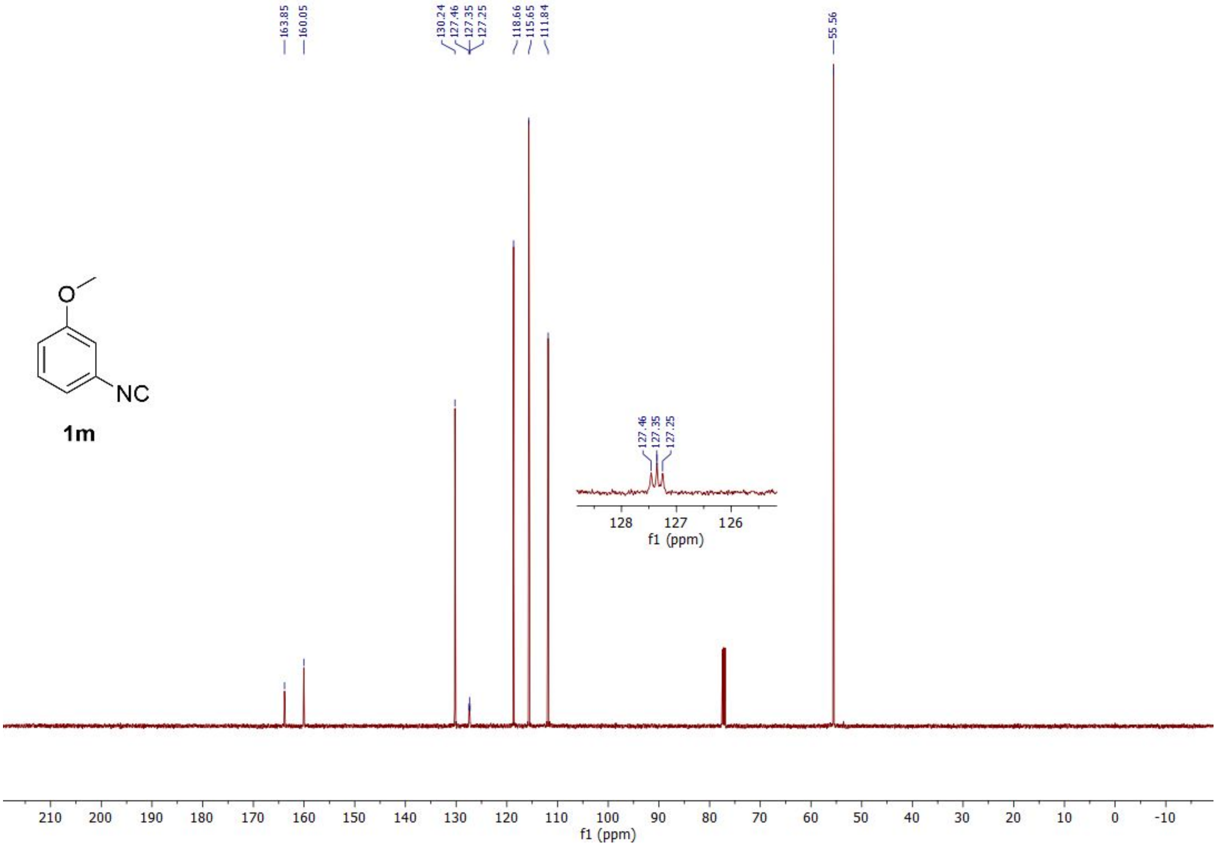

^{1}H NMR spectrum of **1n** (500 MHz, $CDCl_3$)

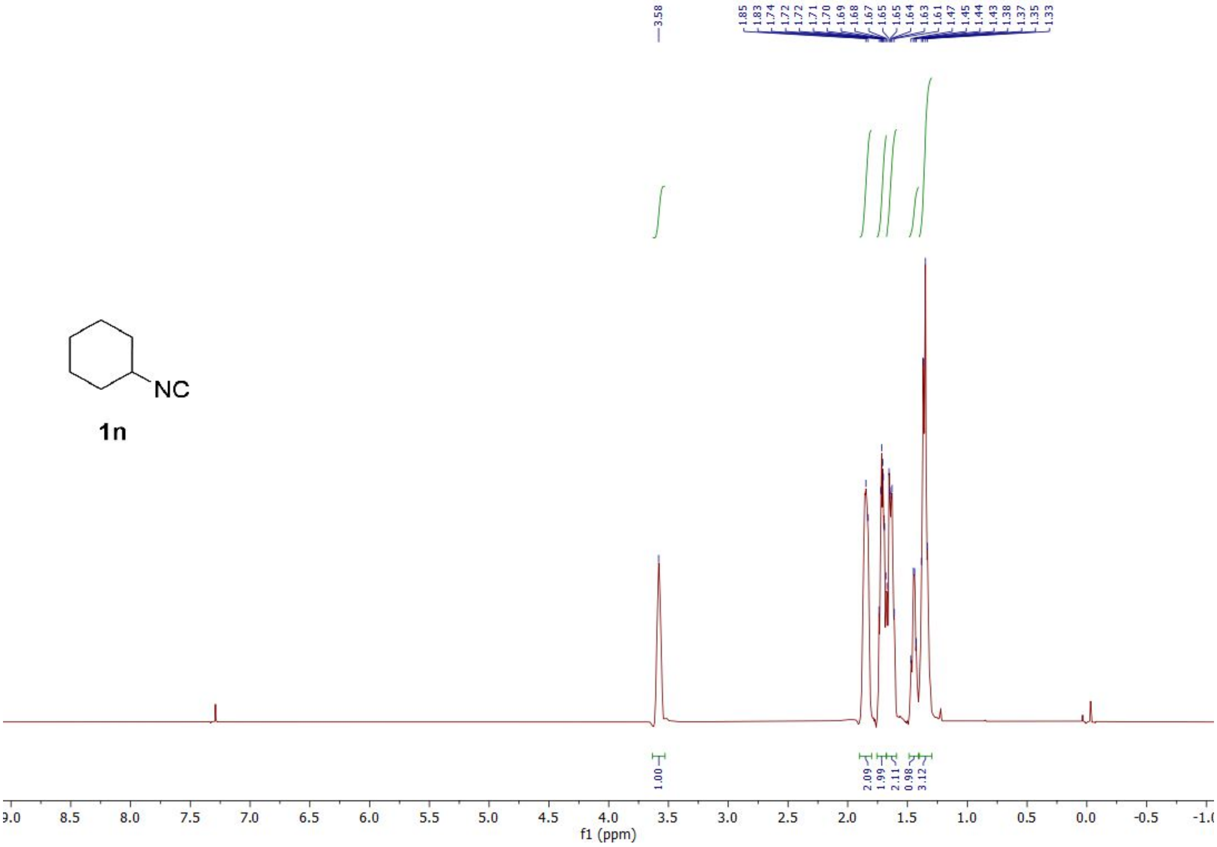

$^{13}C\{^{1}H\}$ NMR spectrum of **1n** (126 MHz, $CDCl_3$)

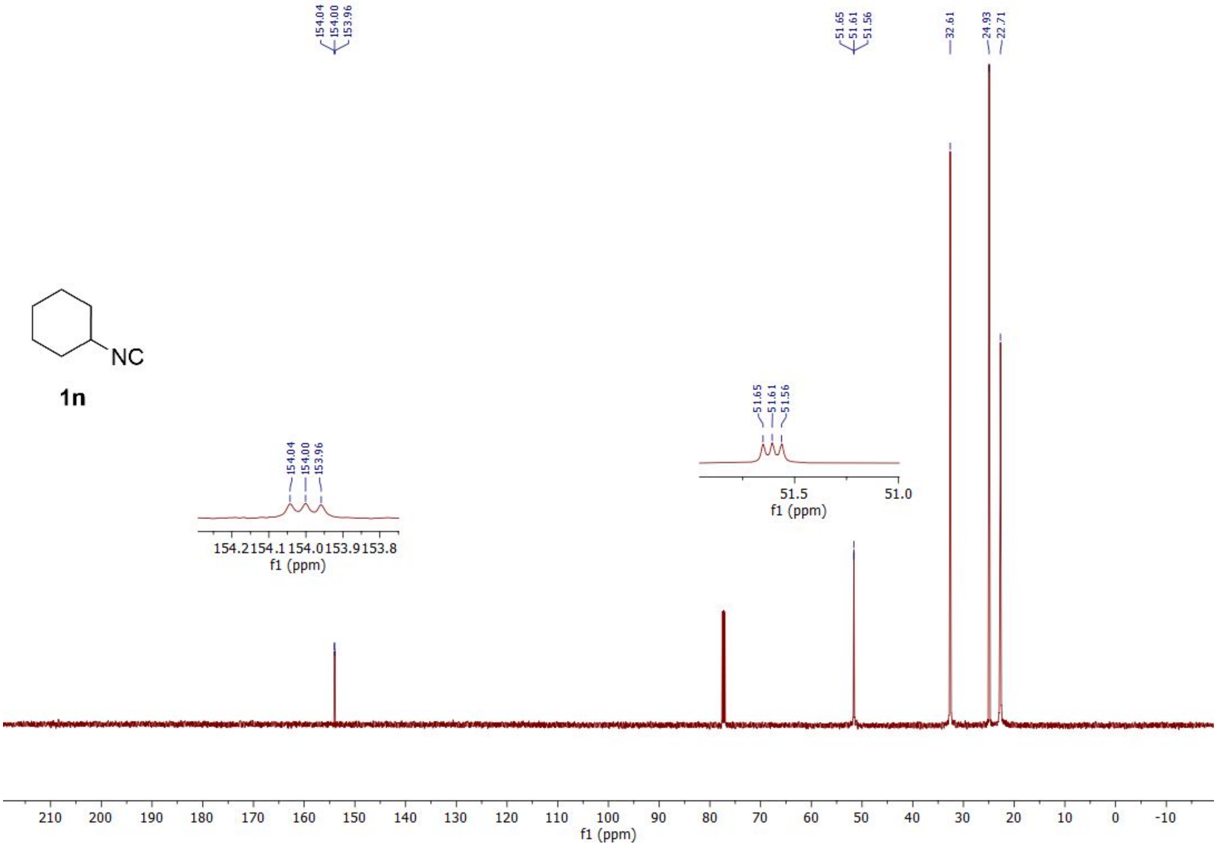

^{1}H NMR spectrum of **1o** (500 MHz, $CDCl_3$)

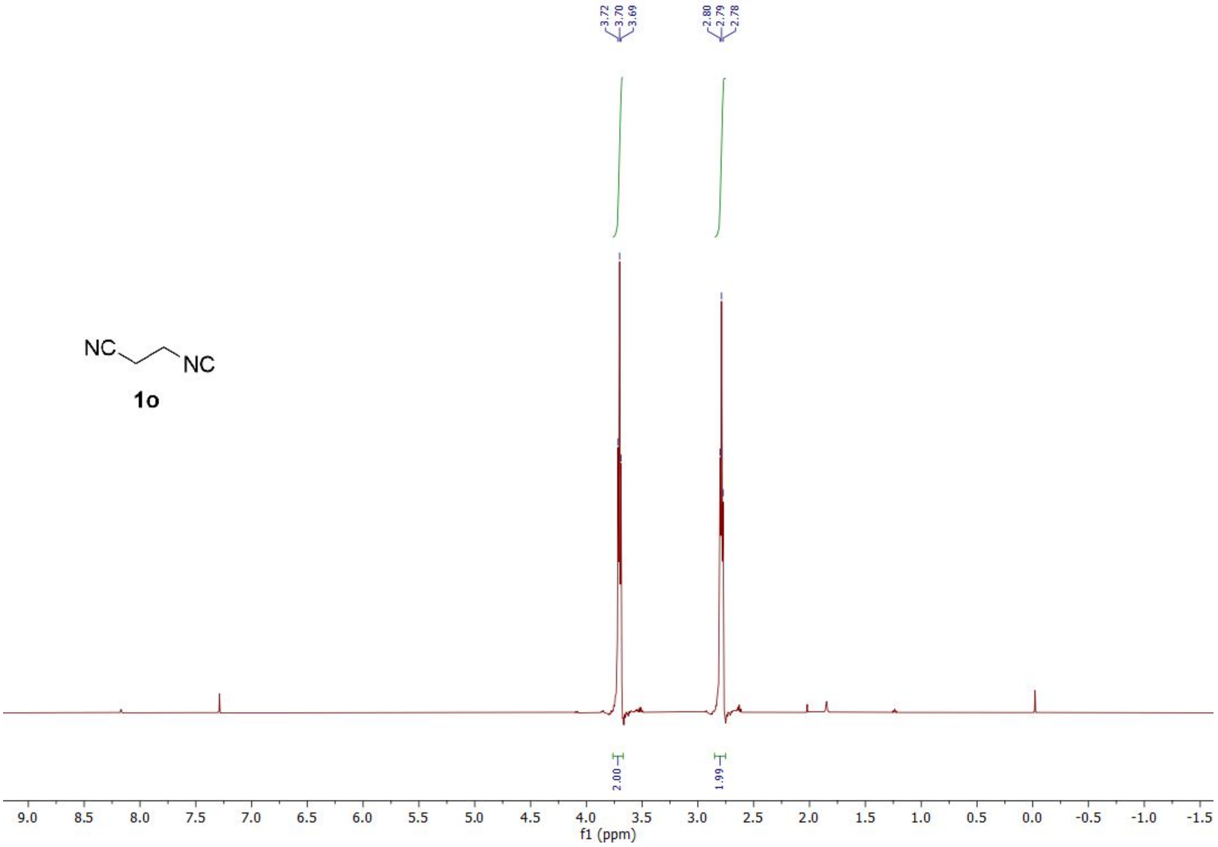

$^{13}C\{^1H\}$ NMR spectrum of **1o** (126 MHz, $CDCl_3$)

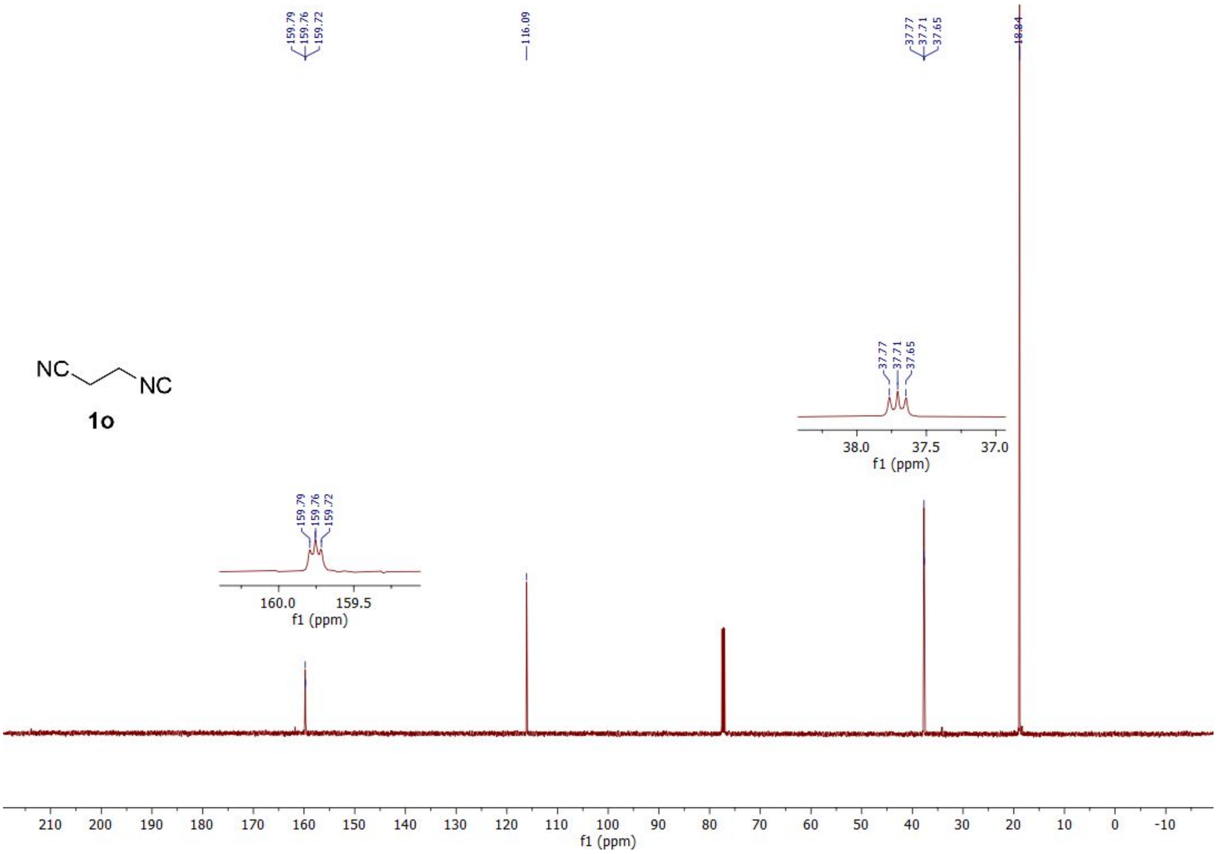

^{1}H NMR spectrum of **1p** (500 MHz, $CDCl_3$)

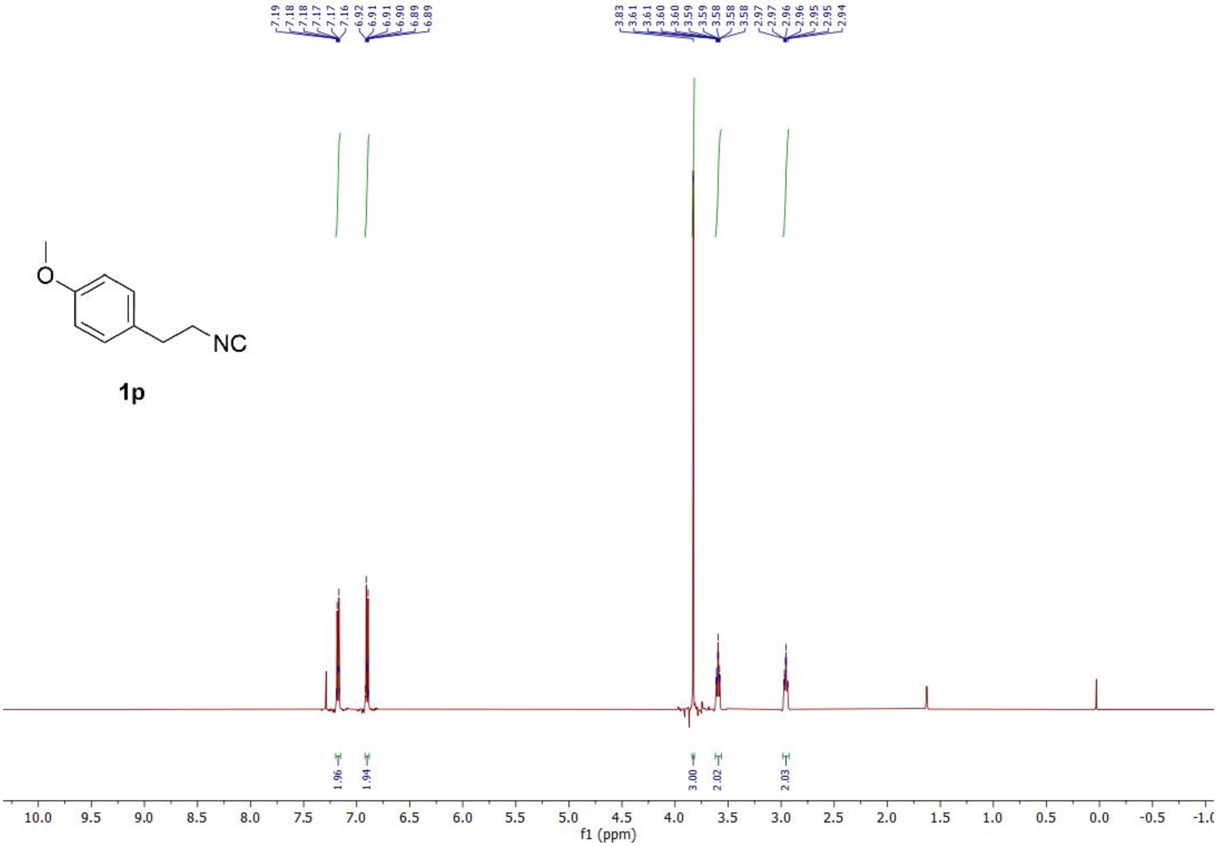

$^{13}C\{^{1}H\}$ NMR spectrum of **1p** (126 MHz, $CDCl_3$)

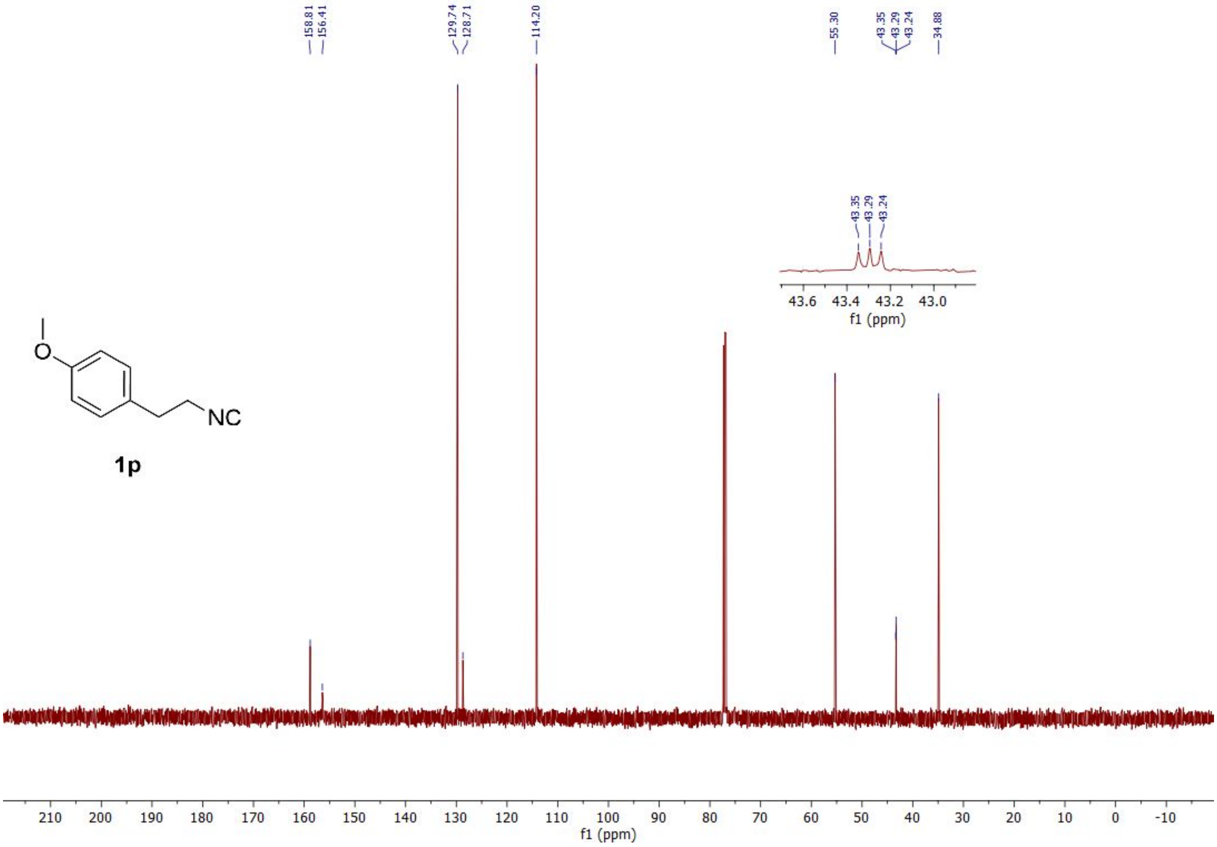

^{1}H NMR spectrum of **1q** (500 MHz, $CDCl_3$)

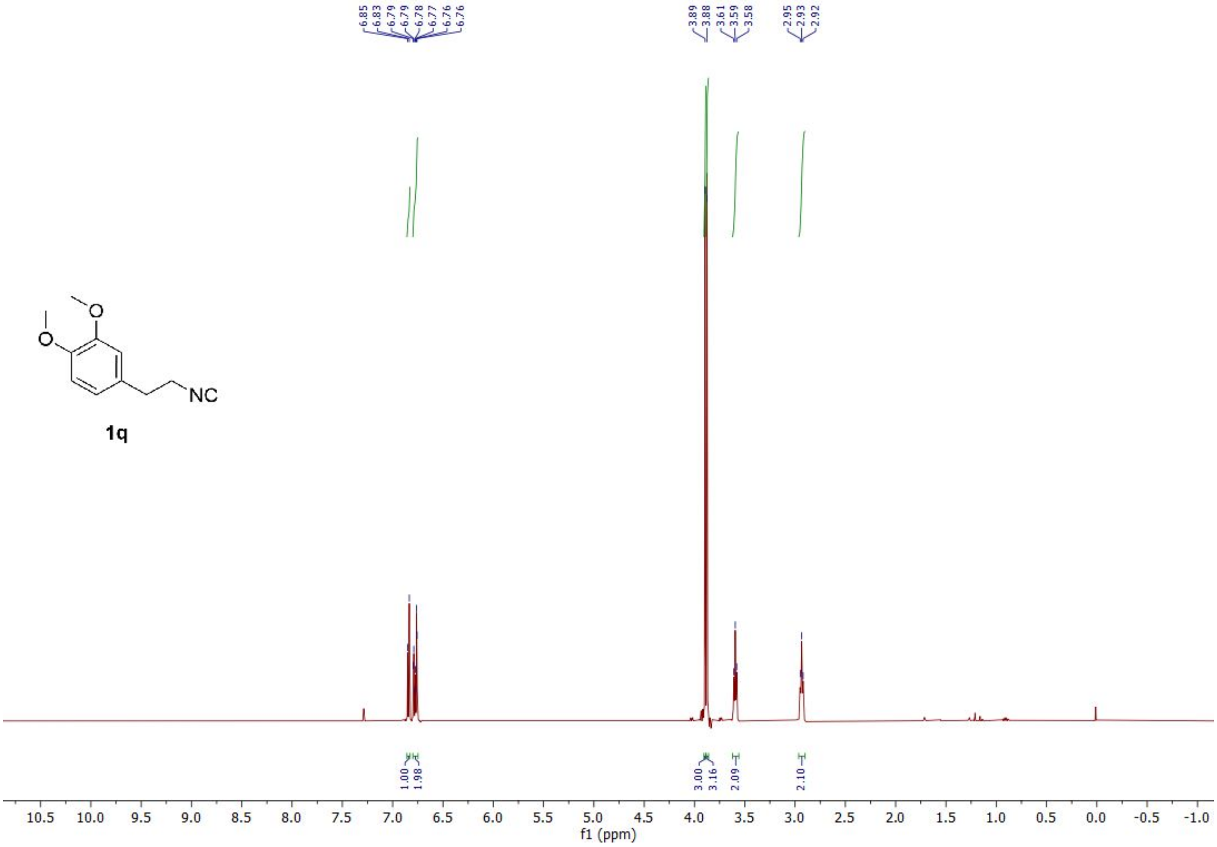

$^{13}C\{^{1}H\}$ NMR spectrum of **1q** (126 MHz, $CDCl_3$)

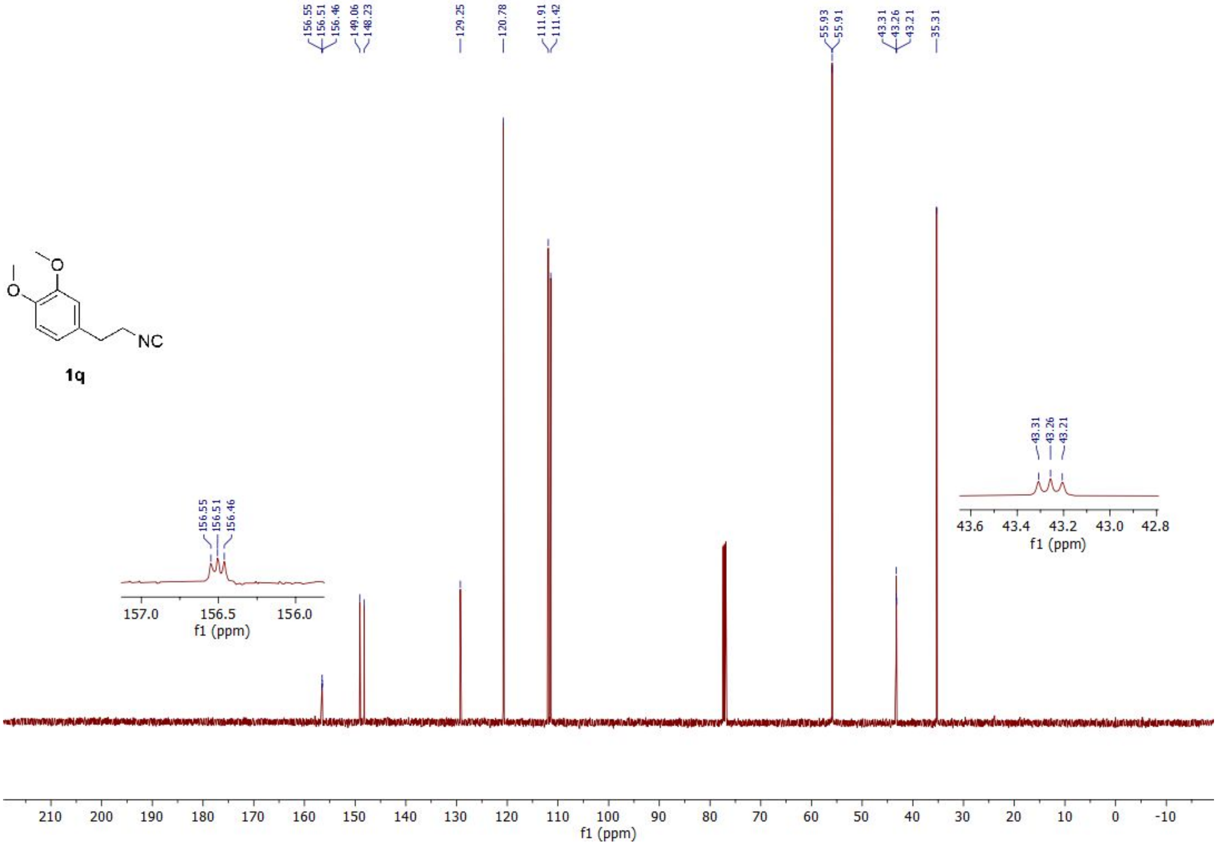

^{1}H NMR spectrum of **5a** (500 MHz, $CDCl_3$)

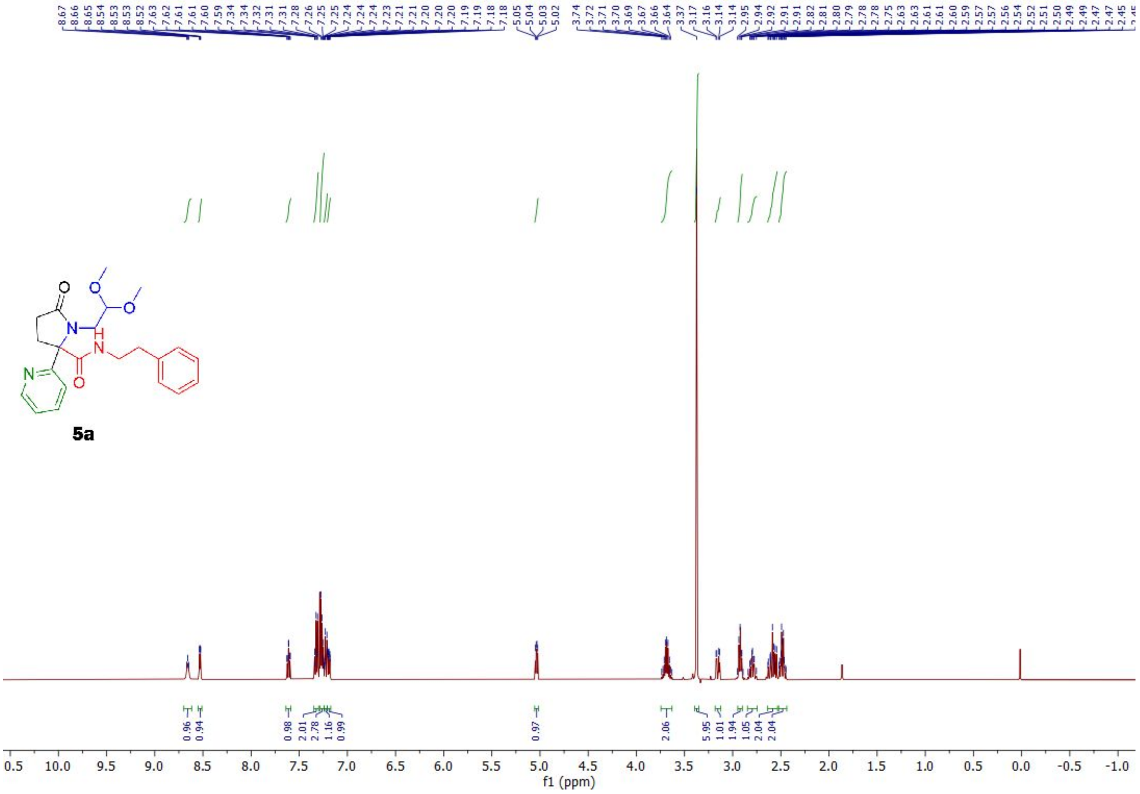

^{13}C{^{1}H} NMR spectrum of **5a** (126 MHz, $CDCl_3$)

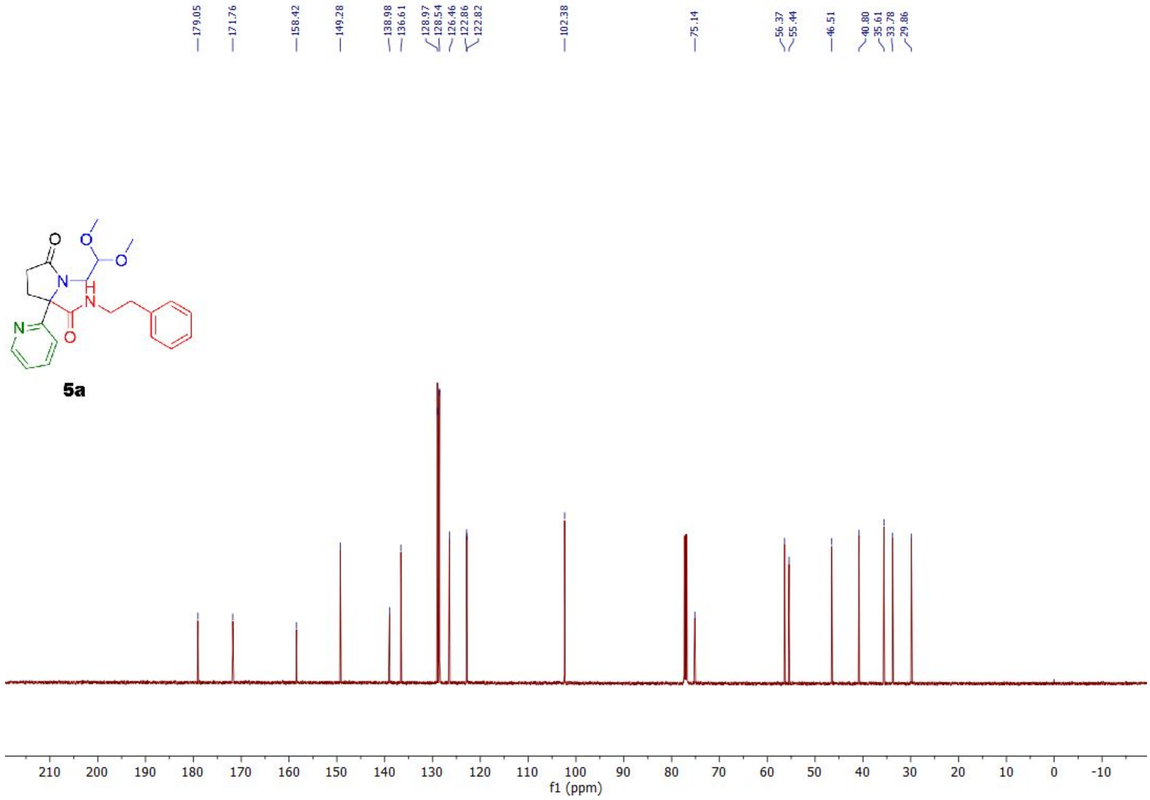

^{1}H NMR spectrum of **5b** (500 MHz, $CDCl_3$)

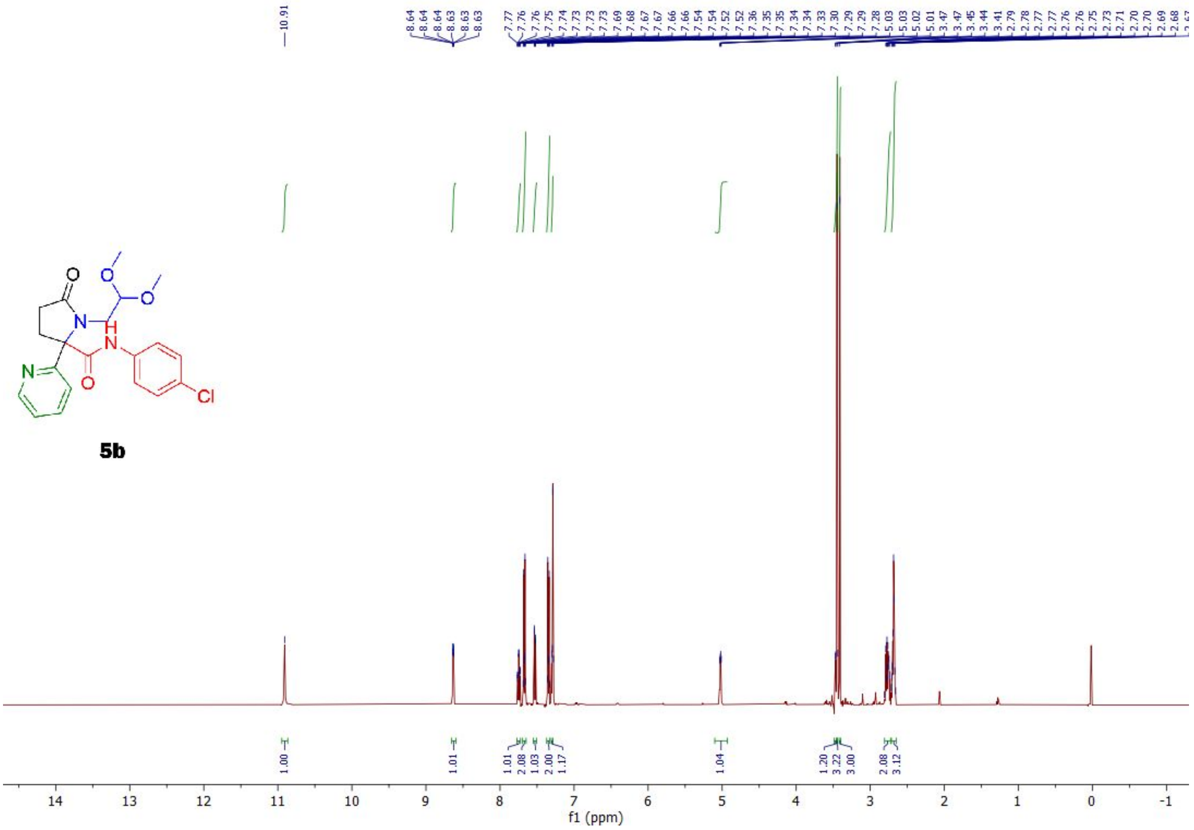

$^{13}C\{^{1}H\}$ NMR spectrum of **5b** (126 MHz, $CDCl_3$)

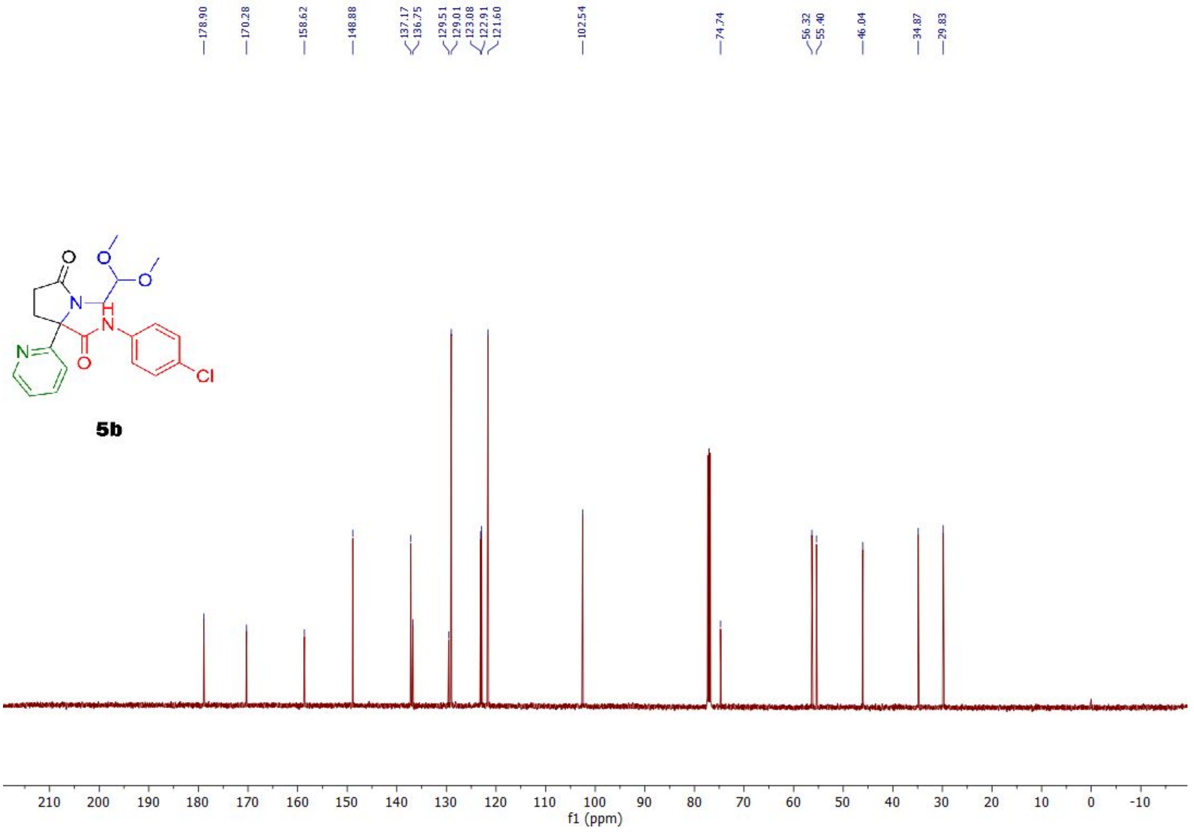

^{1}H NMR spectrum of **5c** (500 MHz, $CDCl_3$)

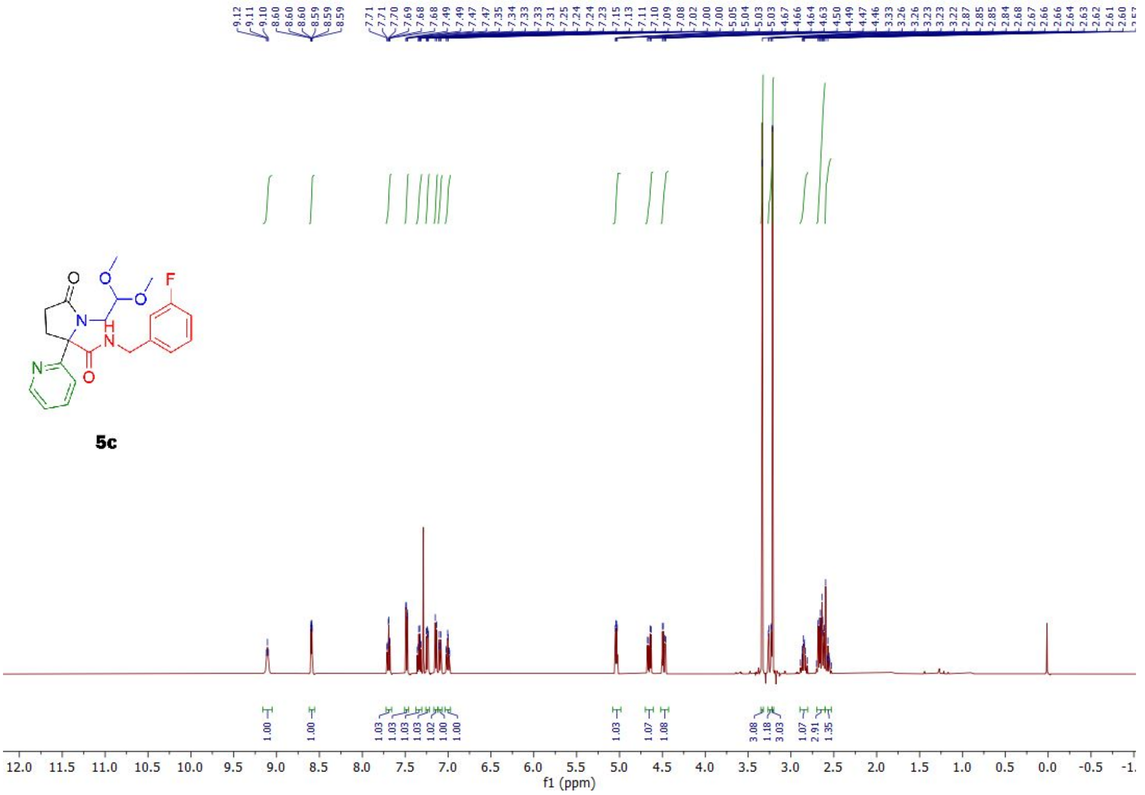

$^{13}C\{^{1}H\}$ NMR spectrum of **5c** (126 MHz, $CDCl_3$)

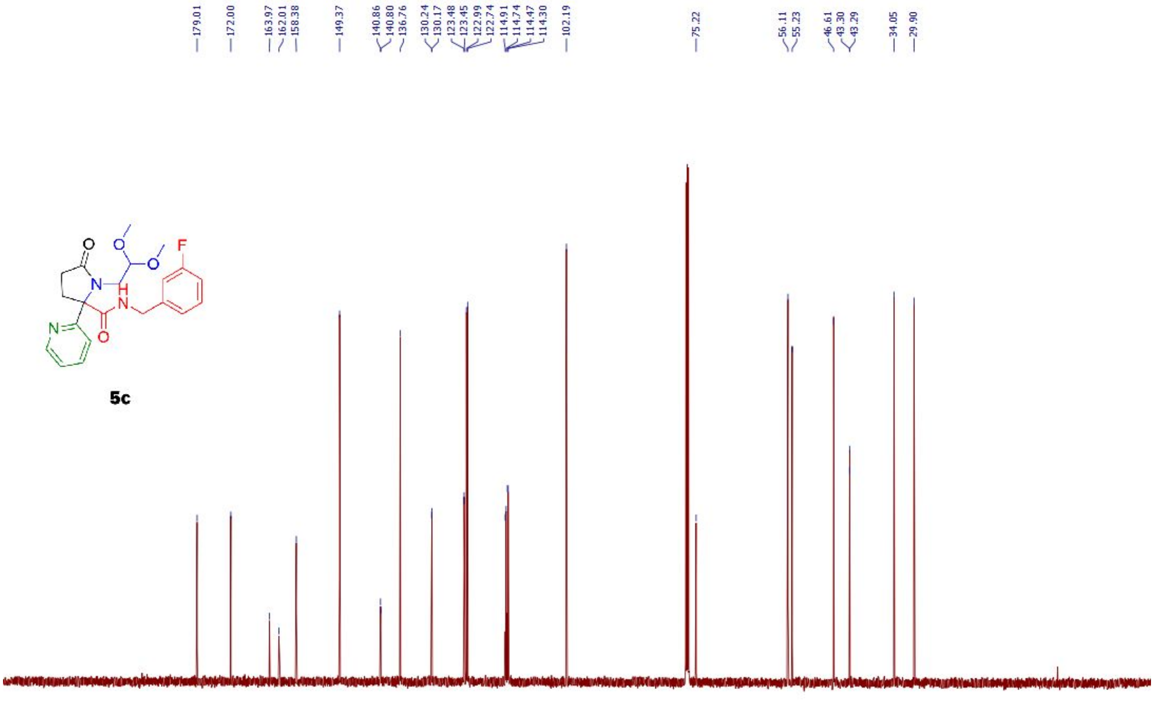

^{1}H NMR spectrum of **5d** (500 MHz, $CDCl_3$)

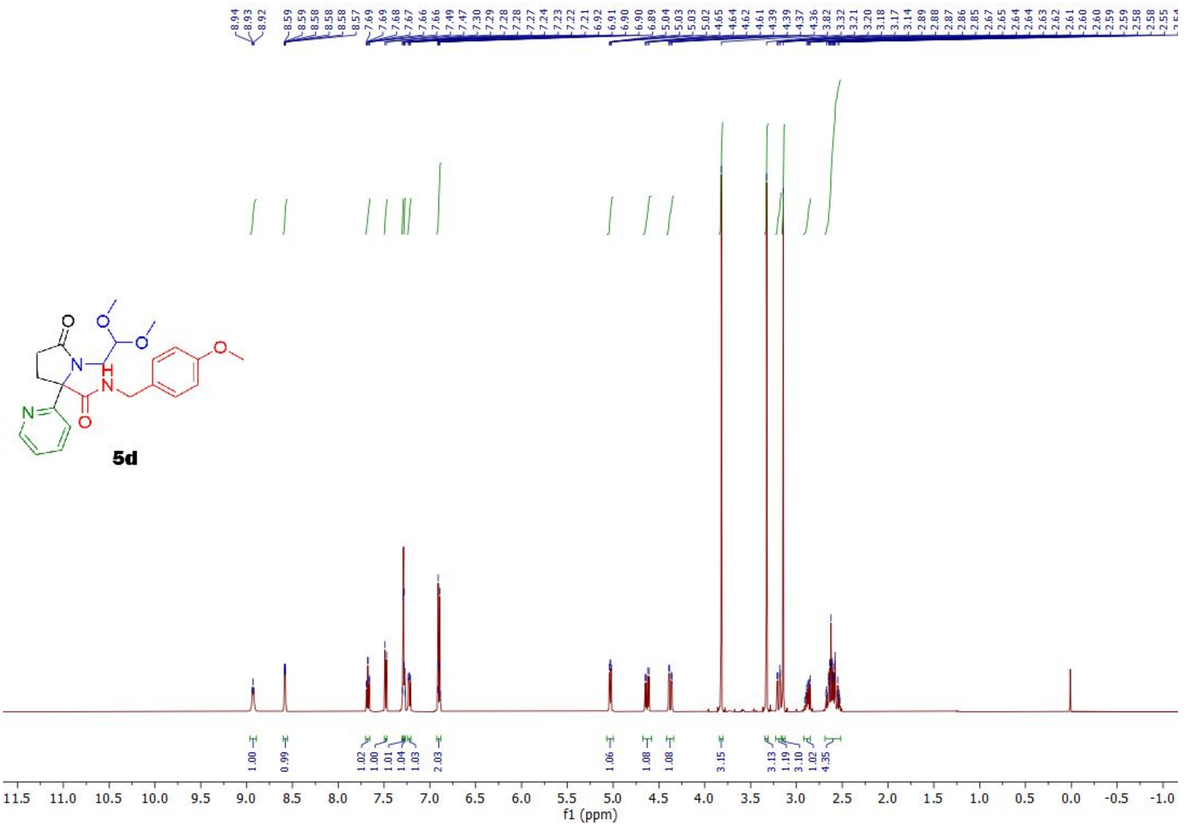

^{13}C{^{1}H} NMR spectrum of **5d** (126 MHz, $CDCl_3$)

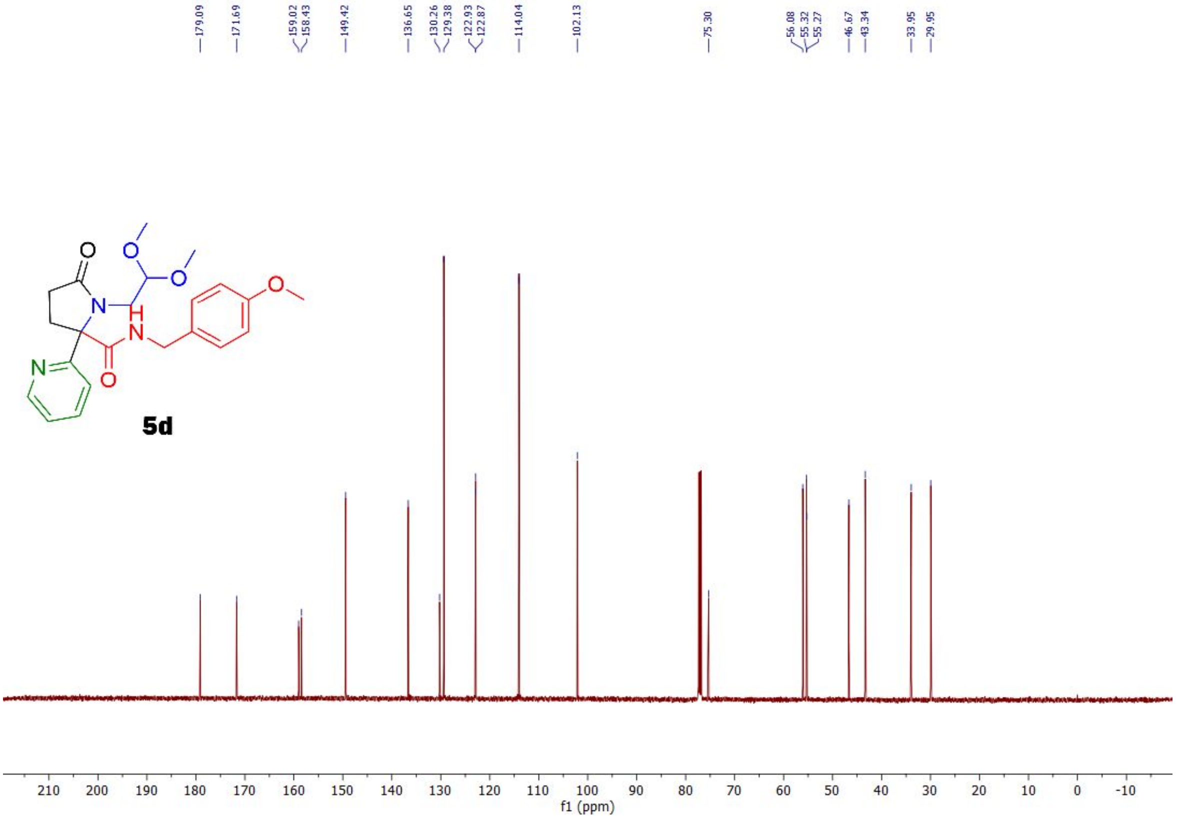

^{1}H NMR spectrum of **5e** (500 MHz, $CDCl_3$)

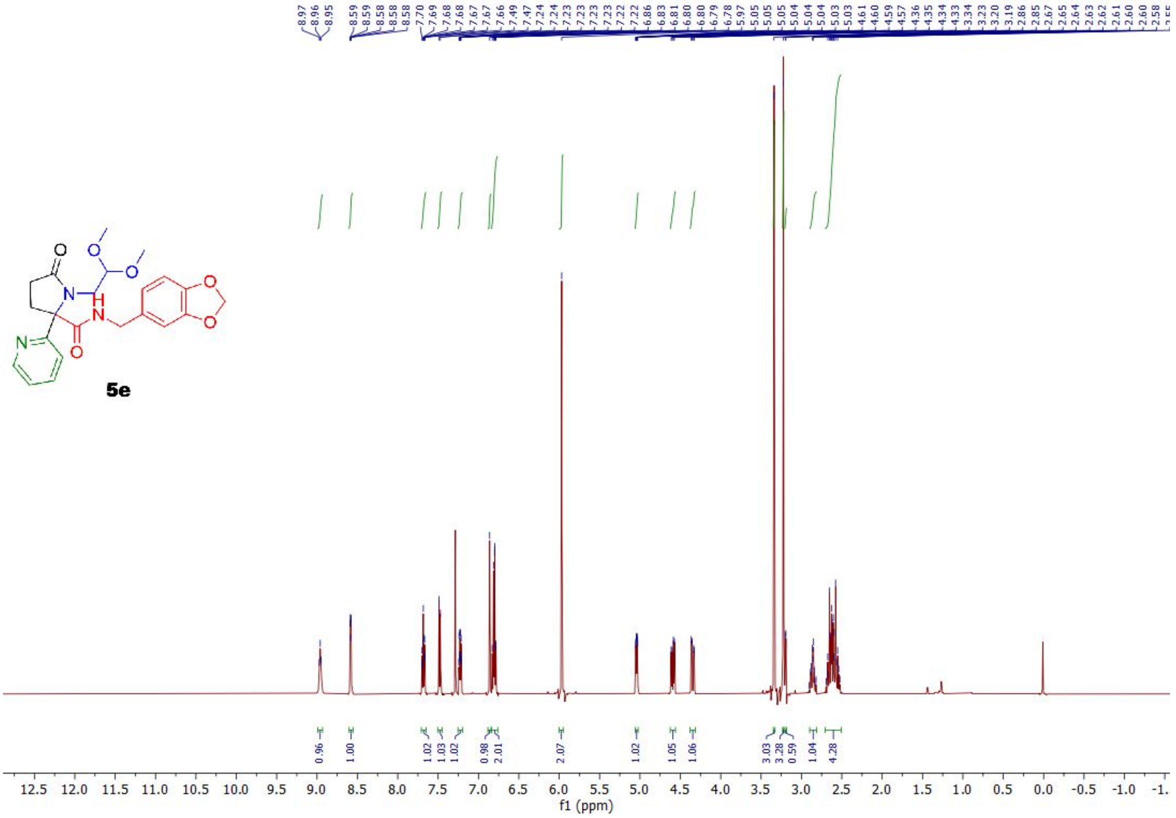

$^{13}C\{^{1}H\}$ NMR spectrum of **5e** (126 MHz, $CDCl_3$)

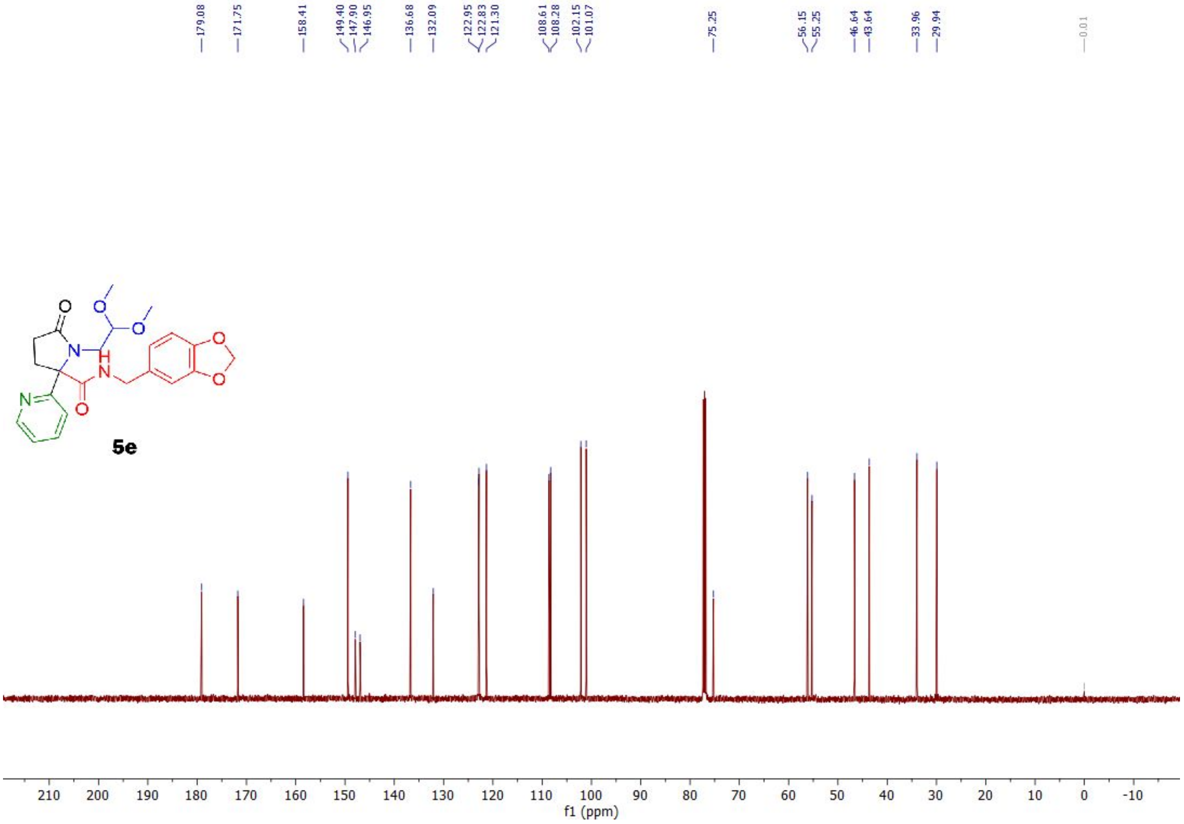

^{1}H NMR spectrum of **5f** (500 MHz, $CDCl_3$)

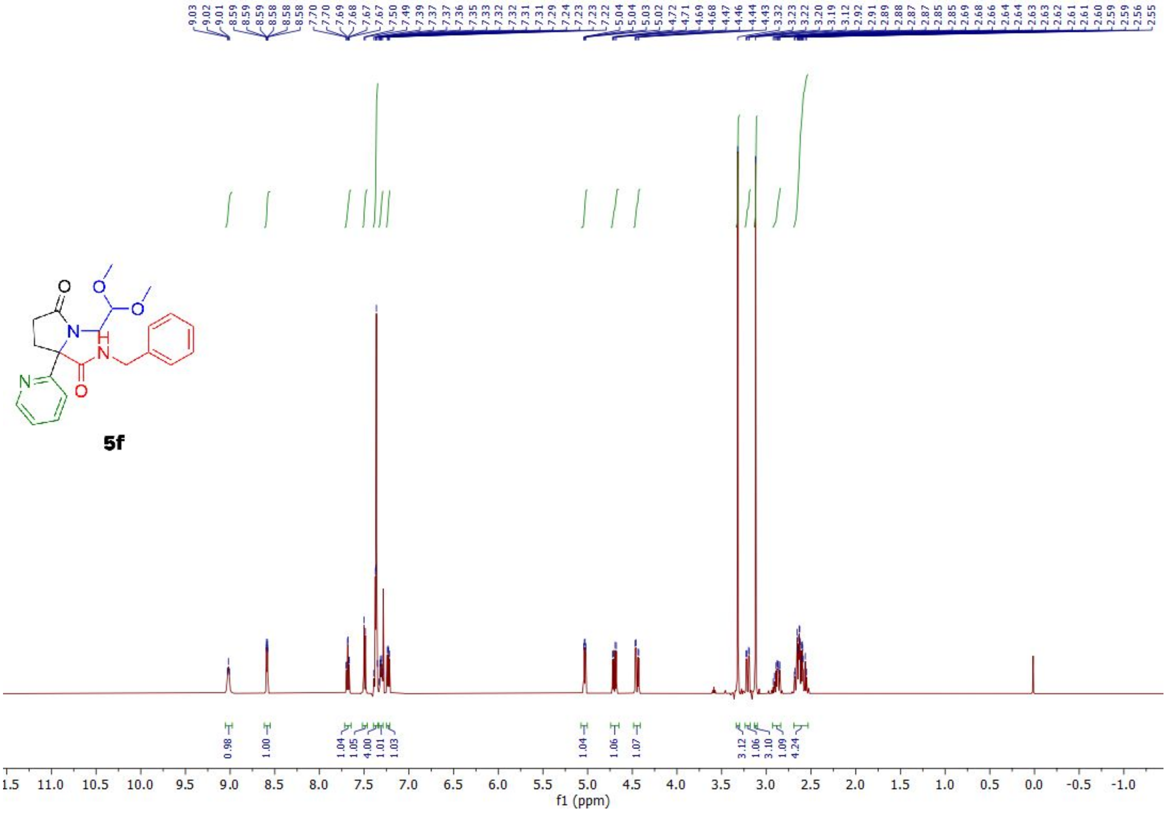

$^{13}C\{^{1}H\}$ NMR spectrum of **5f** (126 MHz, $CDCl_3$)

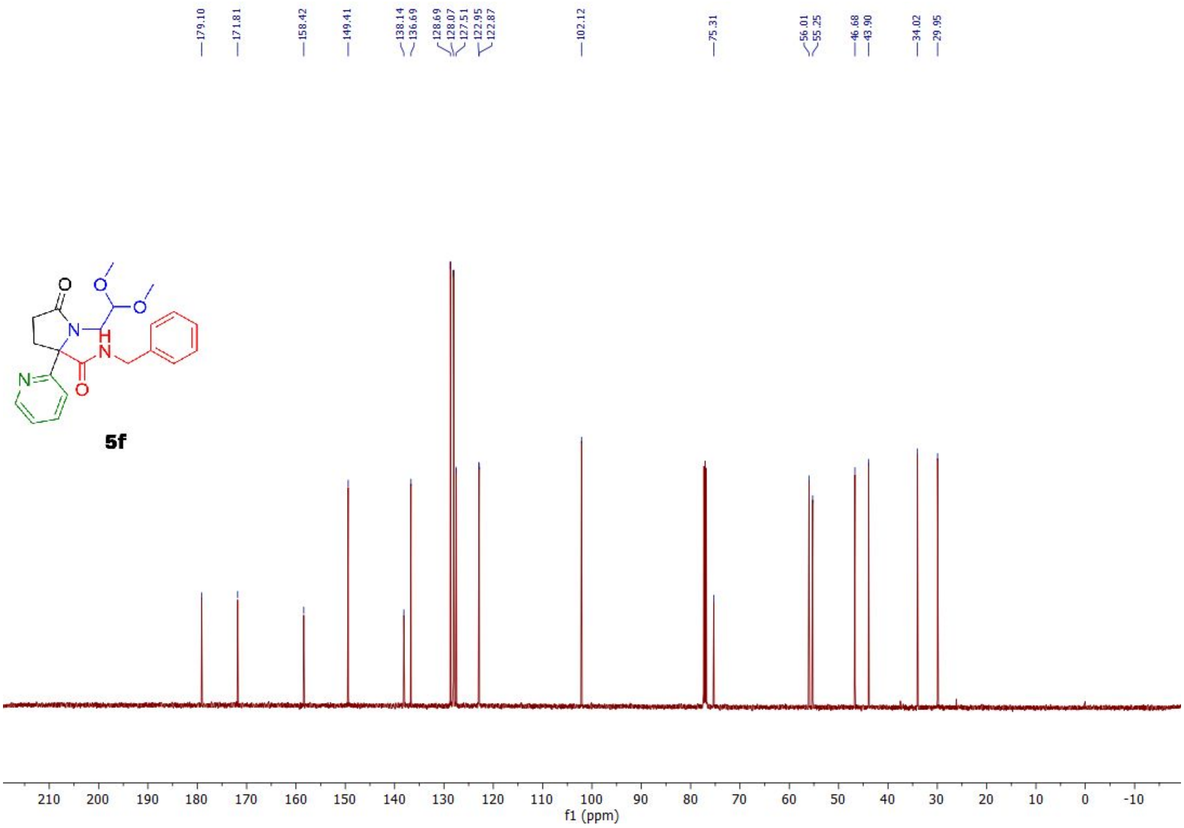

^{1}H NMR spectrum of **5g** (500 MHz, $CDCl_3$)

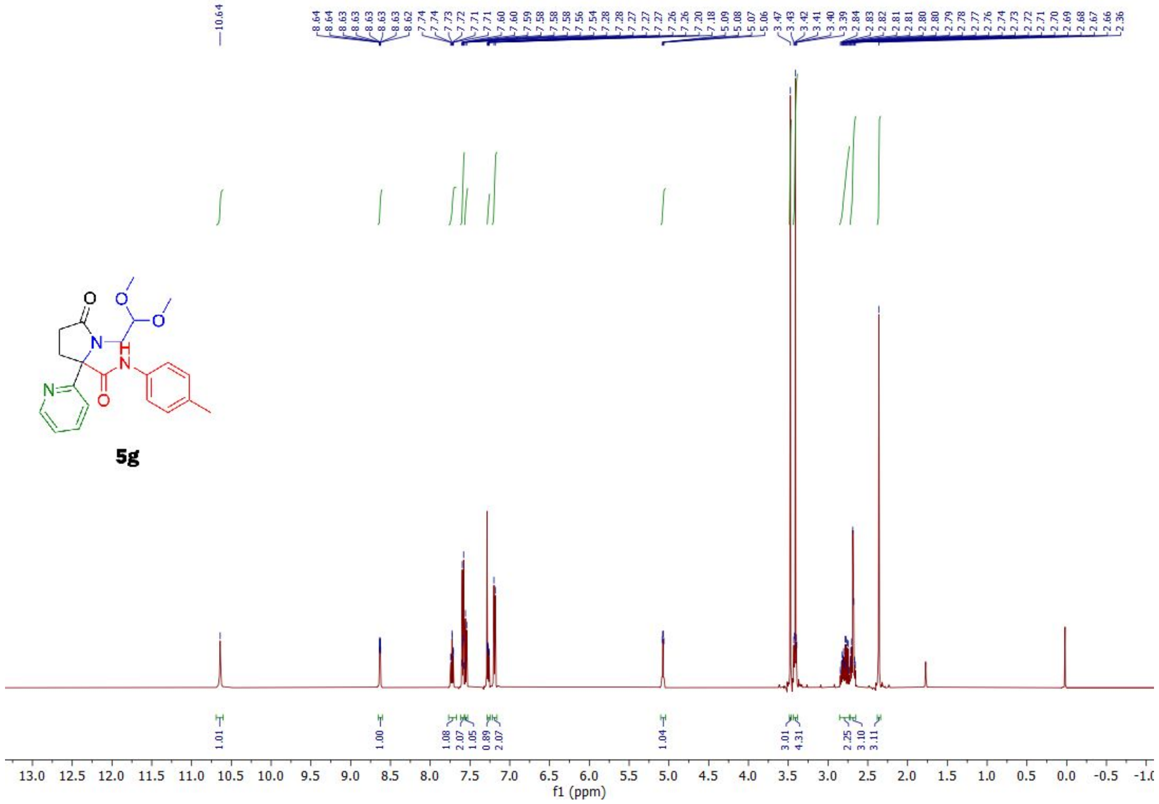

$^{13}C\{^1H\}$ NMR spectrum of **5g** (126 MHz, $CDCl_3$)

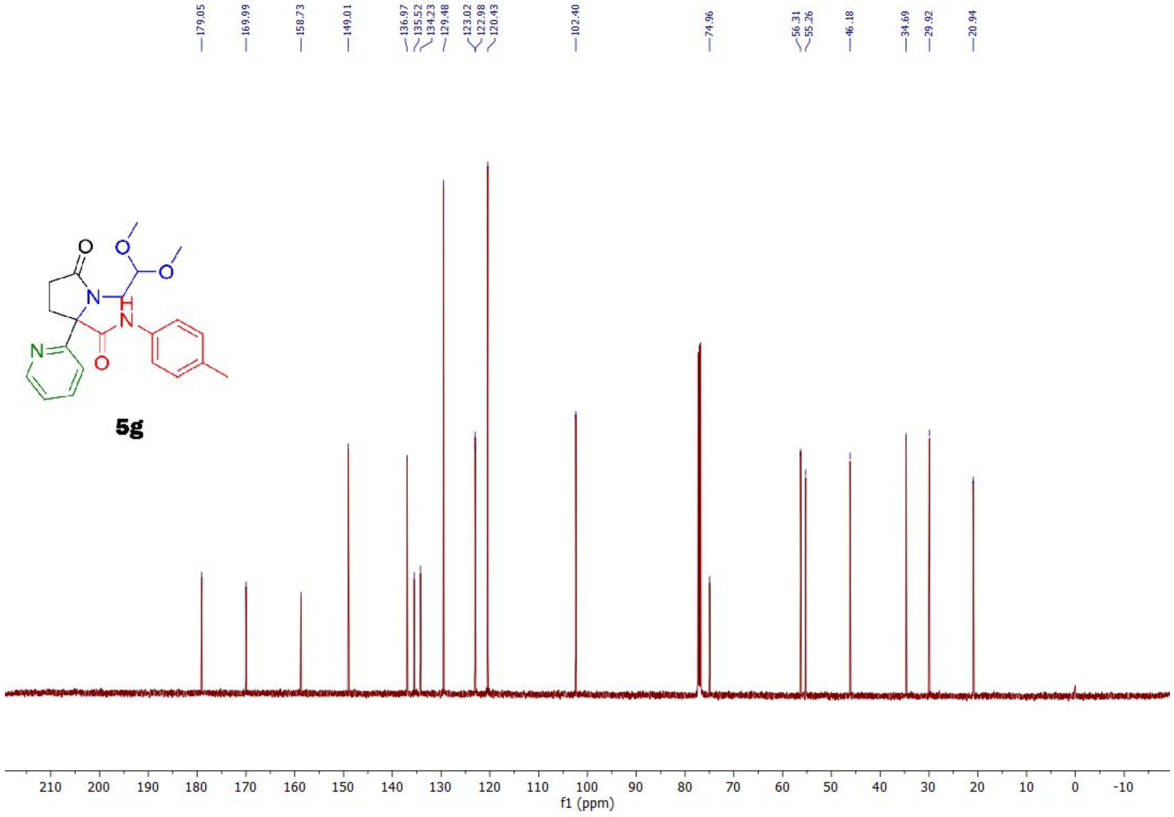

^1H NMR spectrum of **5h** (500 MHz, $CDCl_3$)

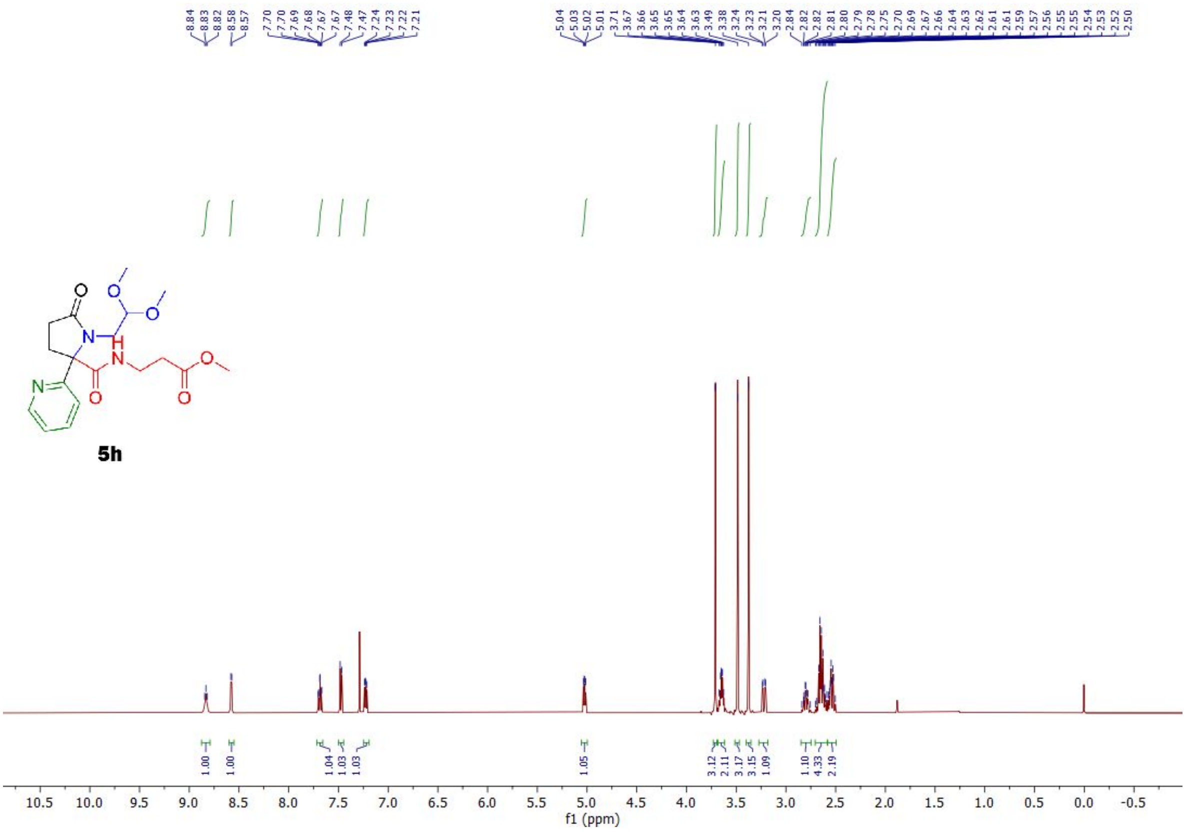

$^{13}C\{^{1}H\}$ NMR spectrum of **5h** (126 MHz, $CDCl_3$)

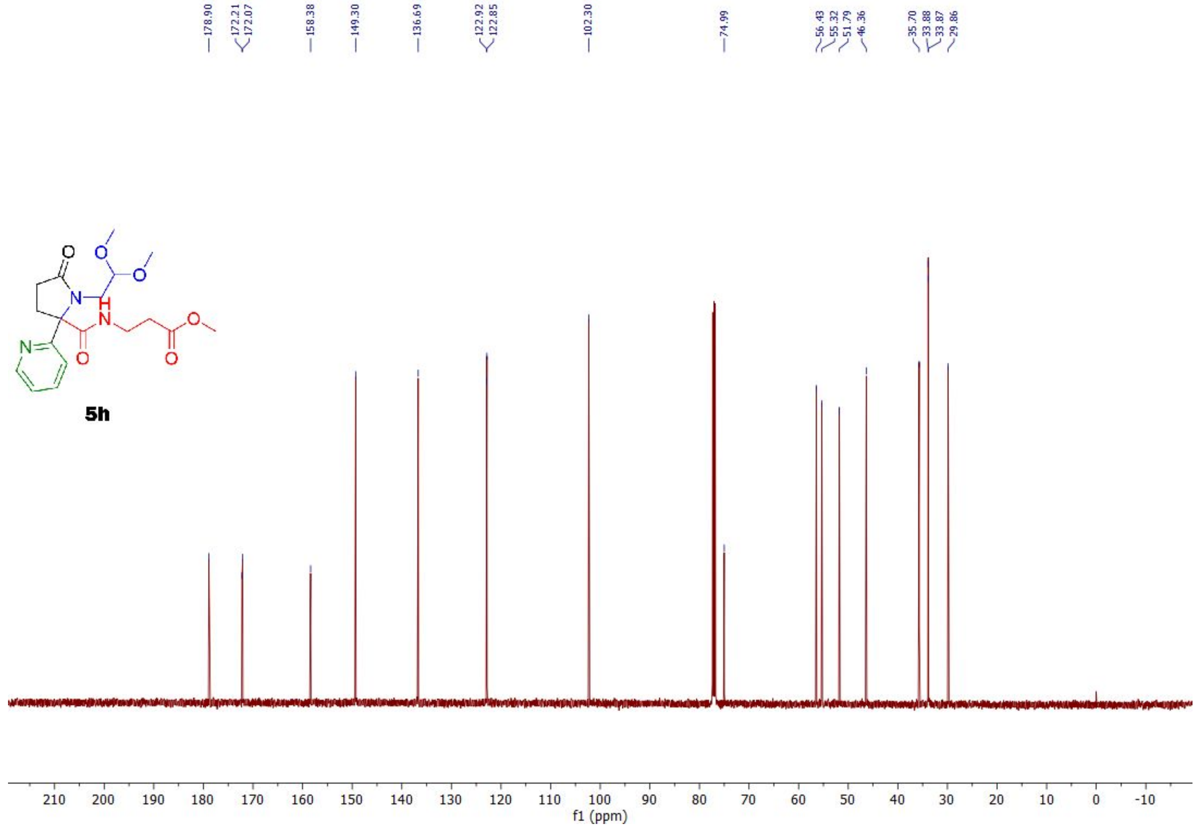

^{1}H NMR spectrum of **5i** (500 MHz, $CDCl_3$)

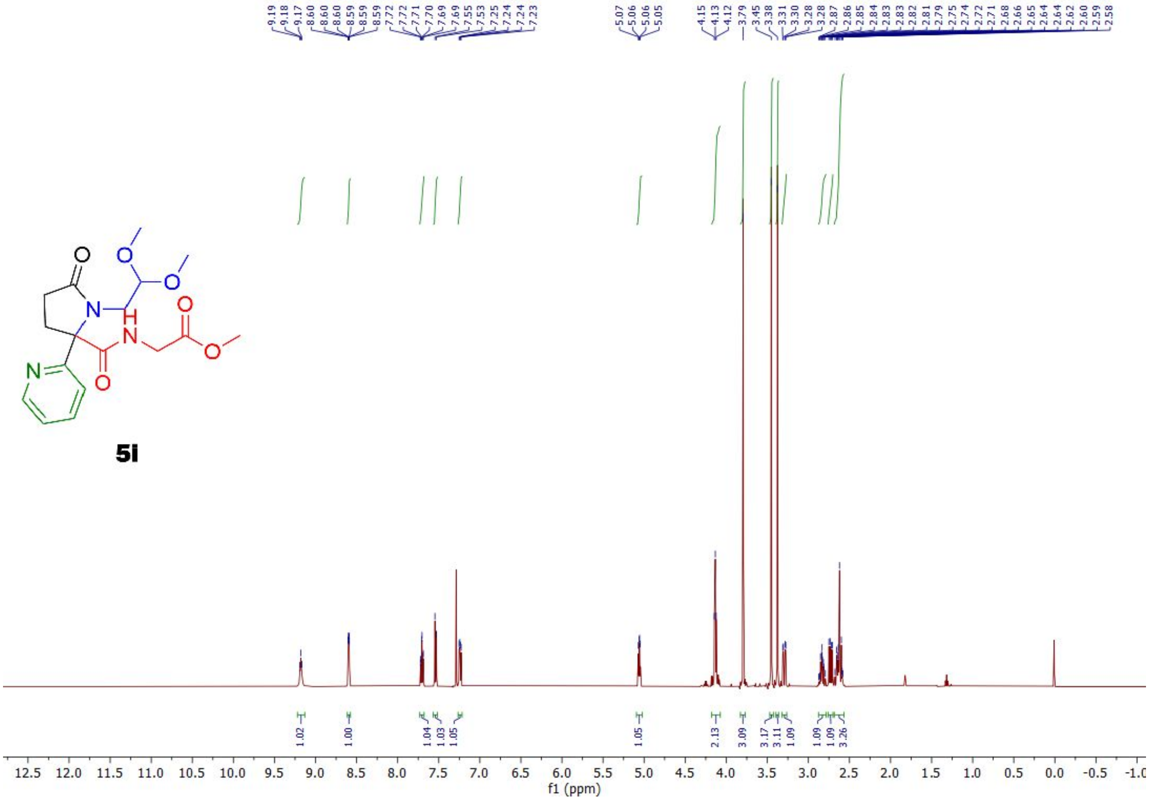

$^{13}C\{^{1}H\}$ NMR spectrum of **5i** (126 MHz, $CDCl_3$)

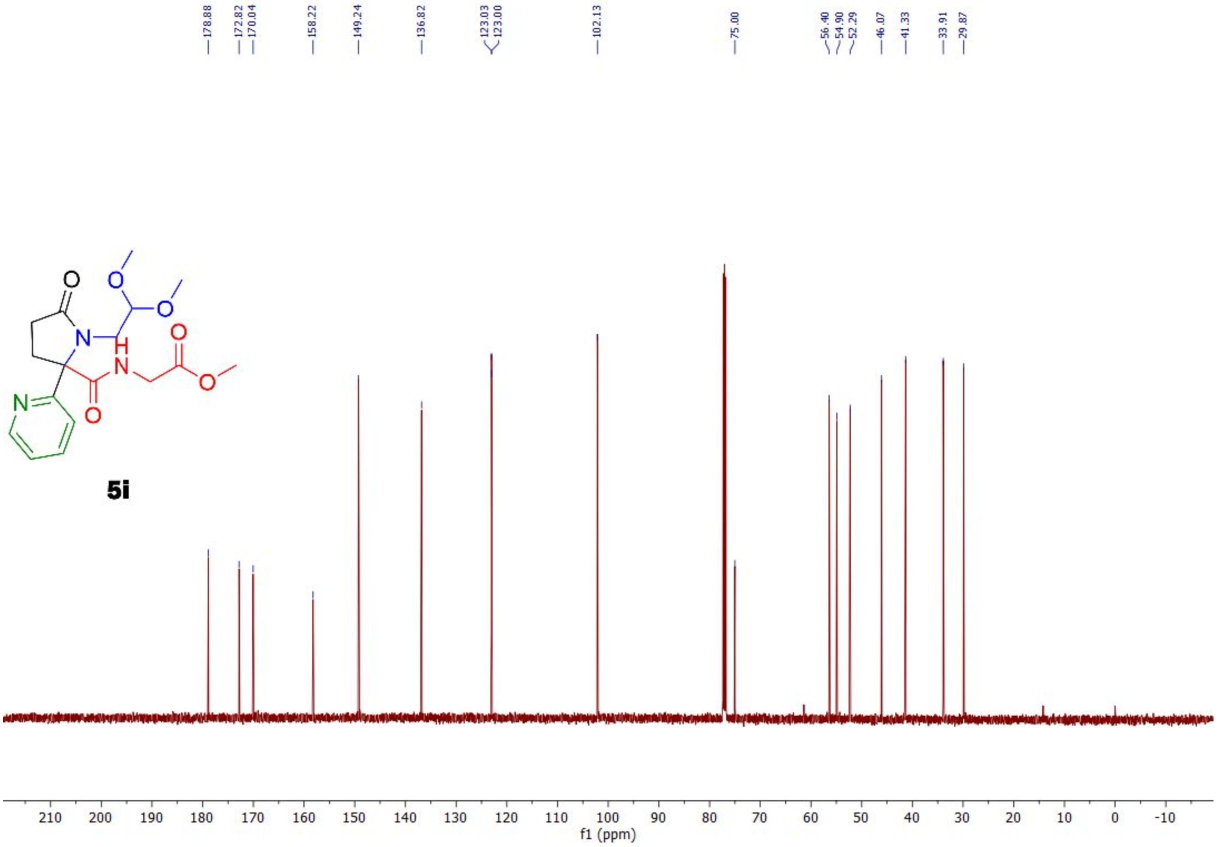

^{1}H NMR spectrum of **5j** (500 MHz, $CDCl_3$)

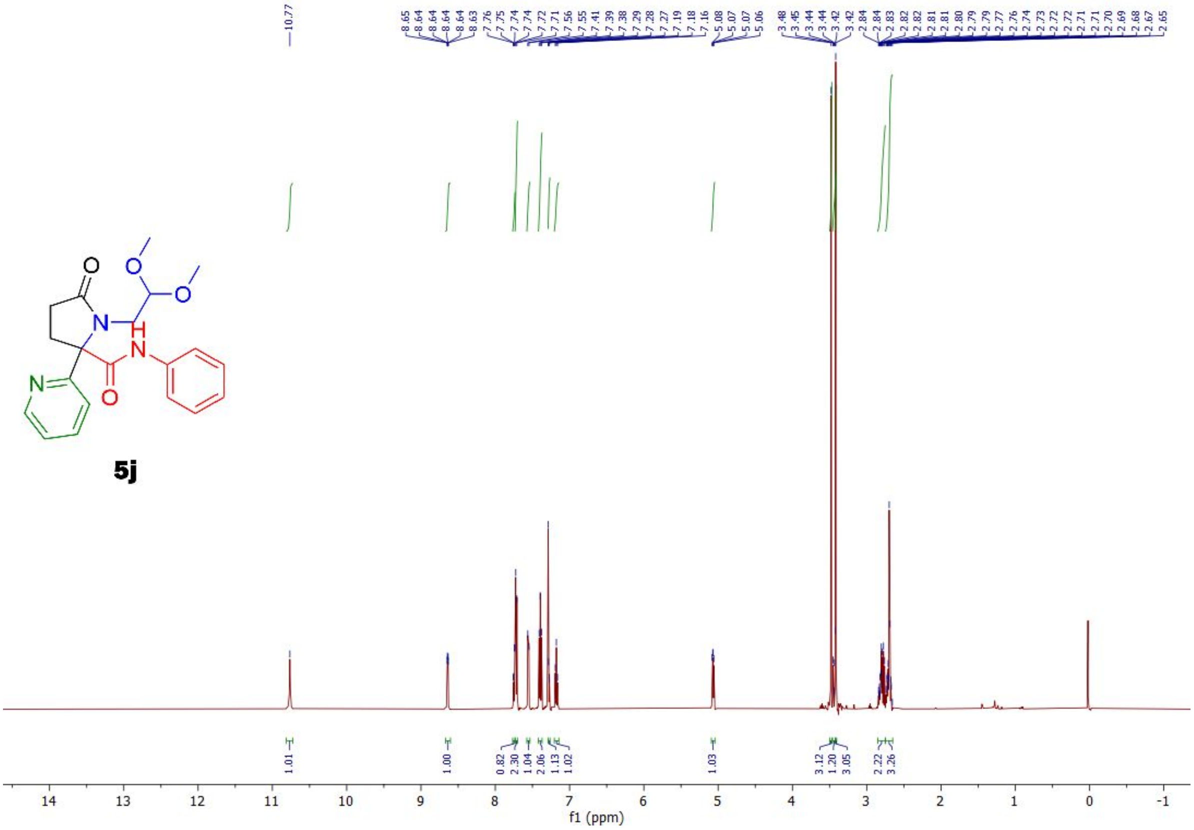

^{13}C{^{1}H} NMR spectrum of **5j** (126 MHz, $CDCl_3$)

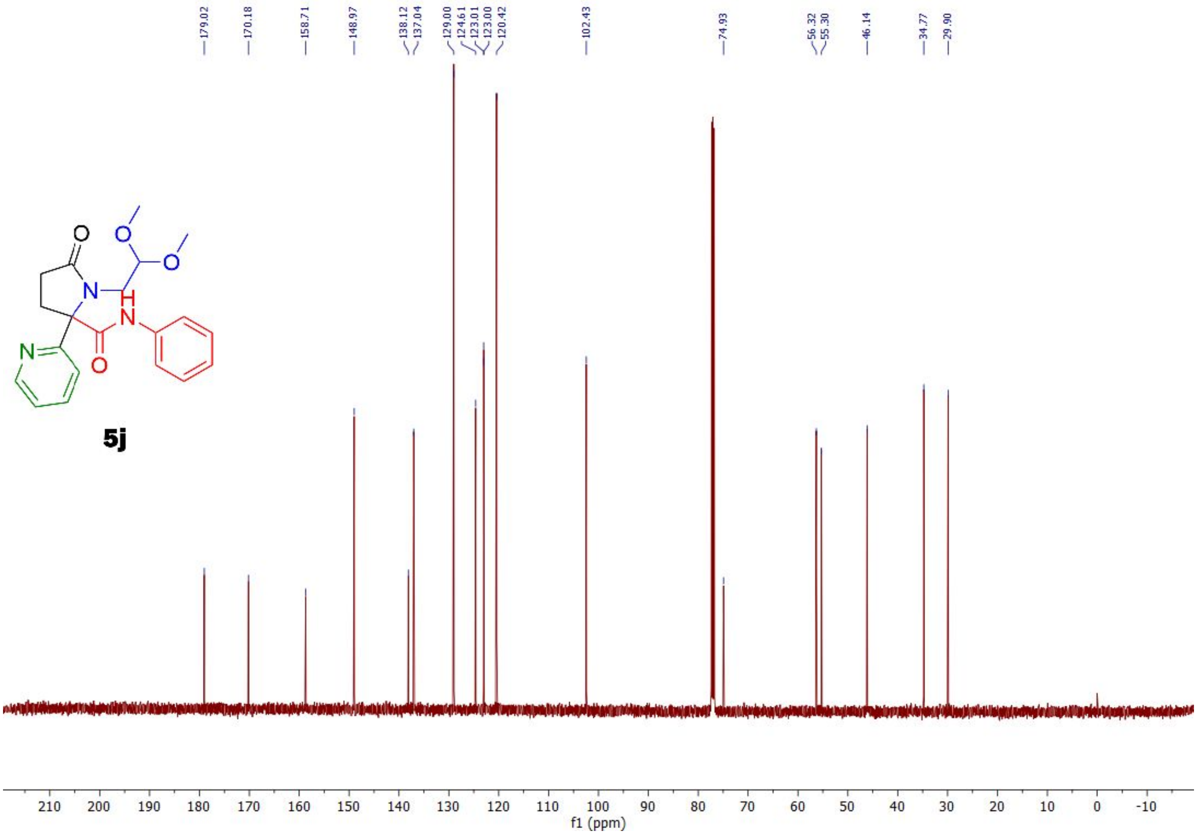

^{1}H NMR spectrum of **5k** (500 MHz, $CDCl_3$)

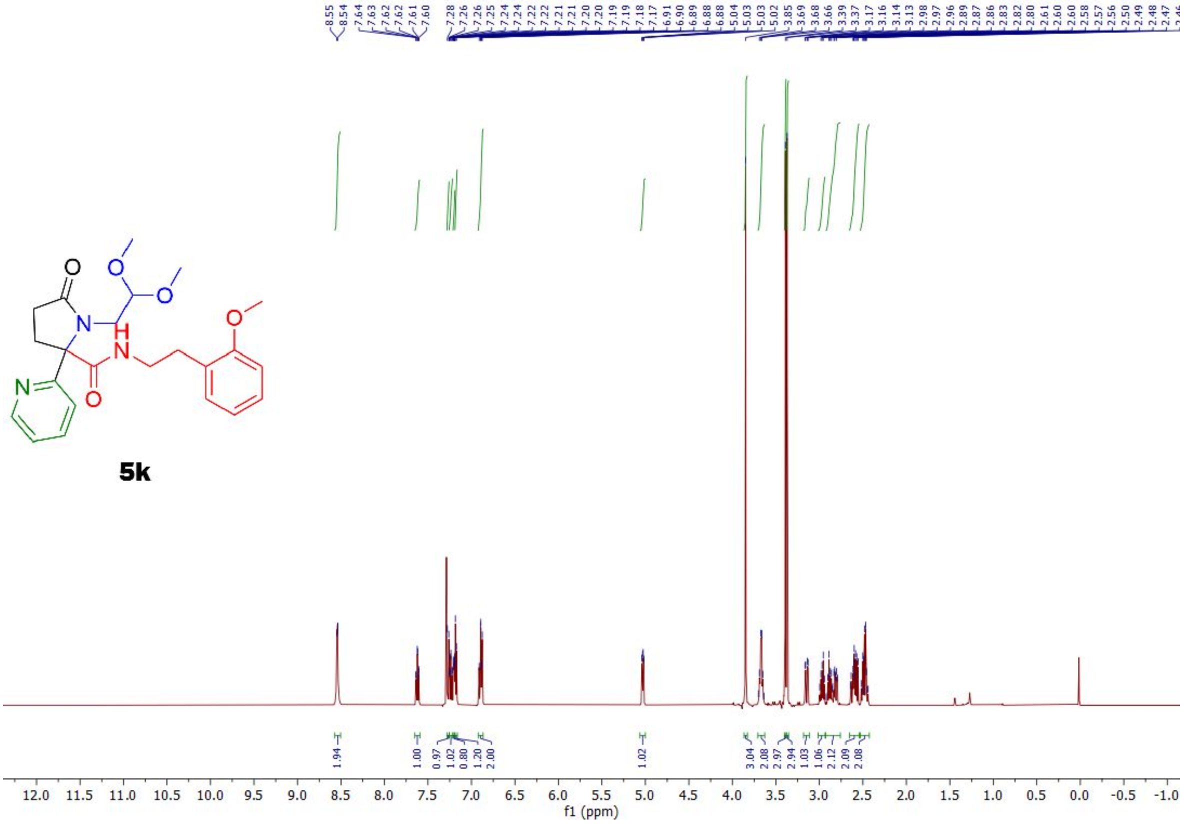

$^{13}C\{^{1}H\}$ NMR spectrum of **5k** (126 MHz, $CDCl_3$)

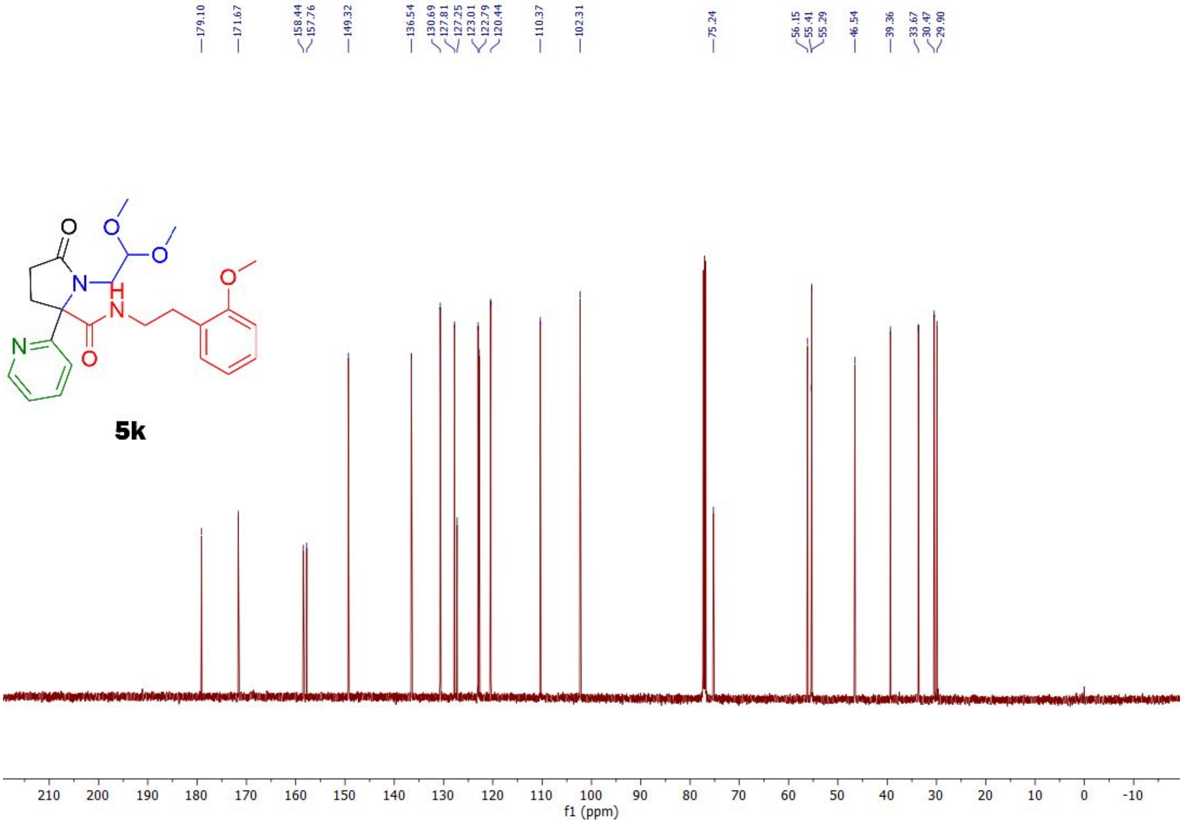

^{1}H NMR spectrum of **5l** (500 MHz, $CDCl_3$)

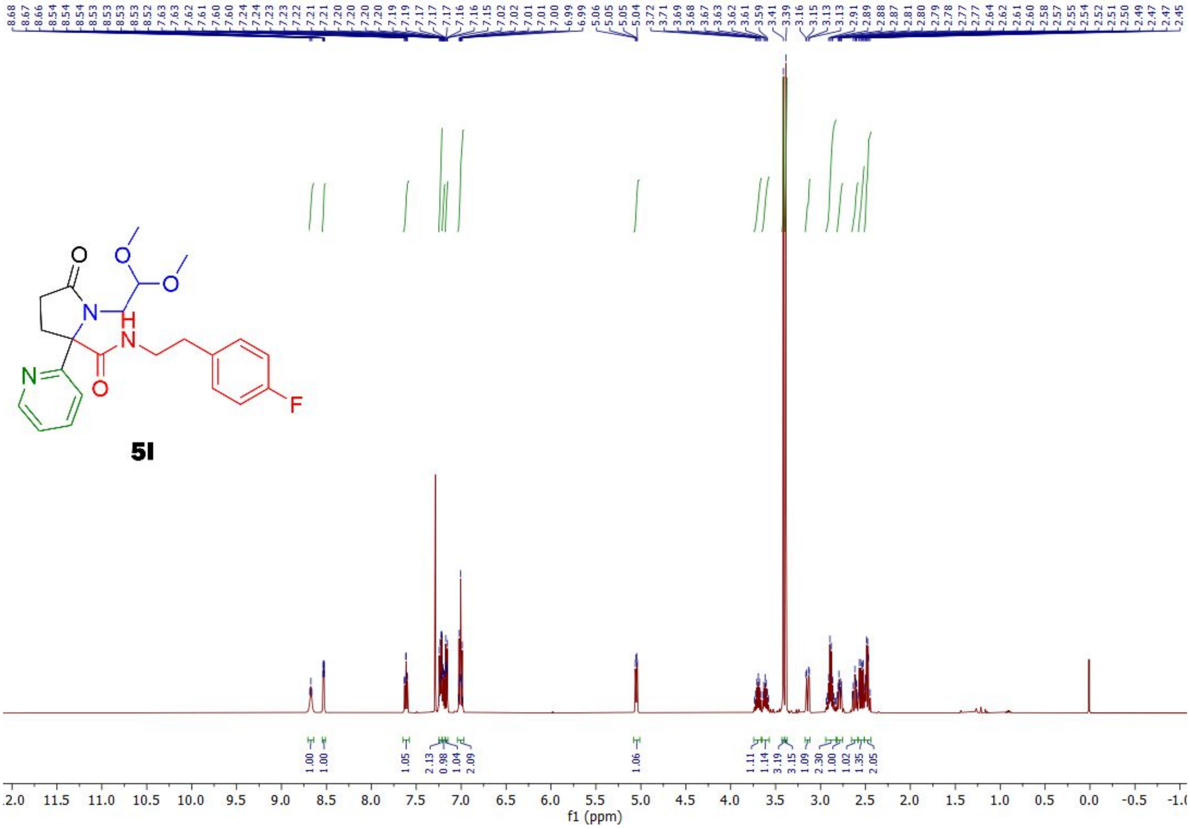

$^{13}C\{^{1}H\}$ NMR spectrum of **5l** (126 MHz, $CDCl_3$)

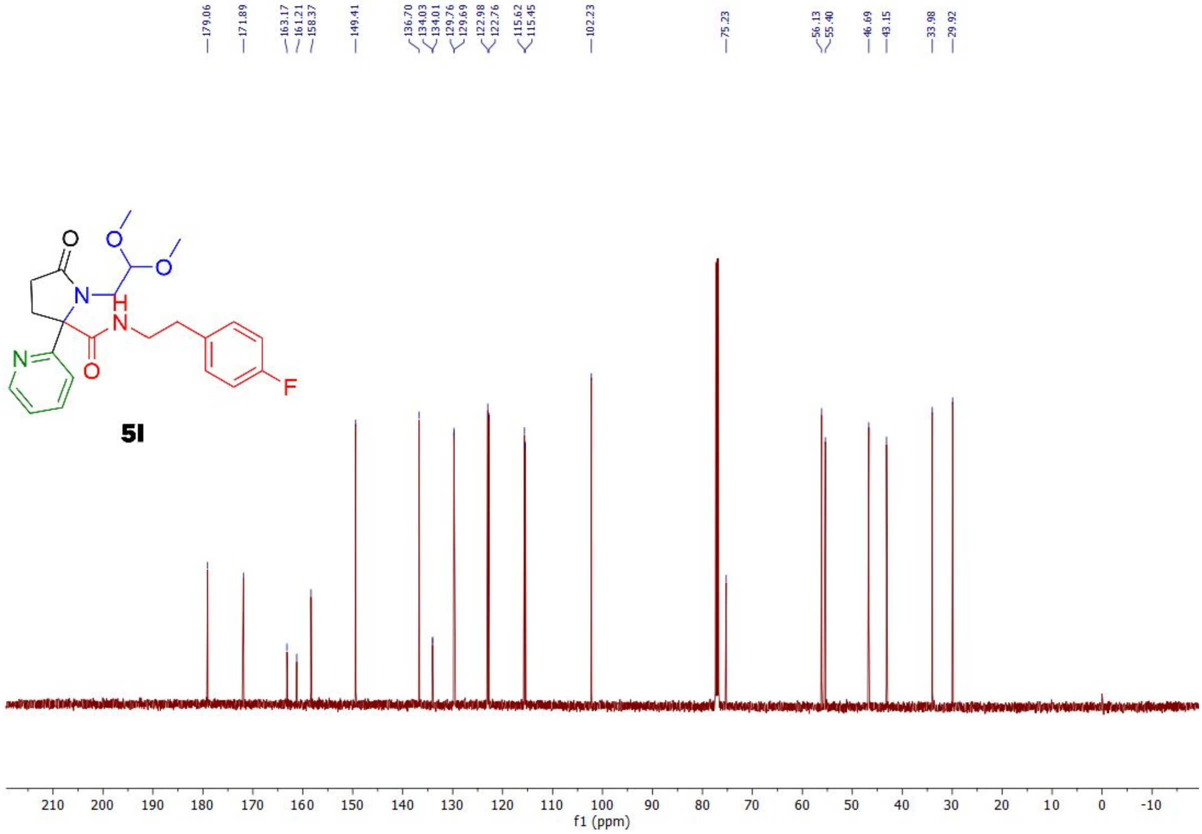

^{1}H NMR spectrum of **5m** (500 MHz, $CDCl_3$)

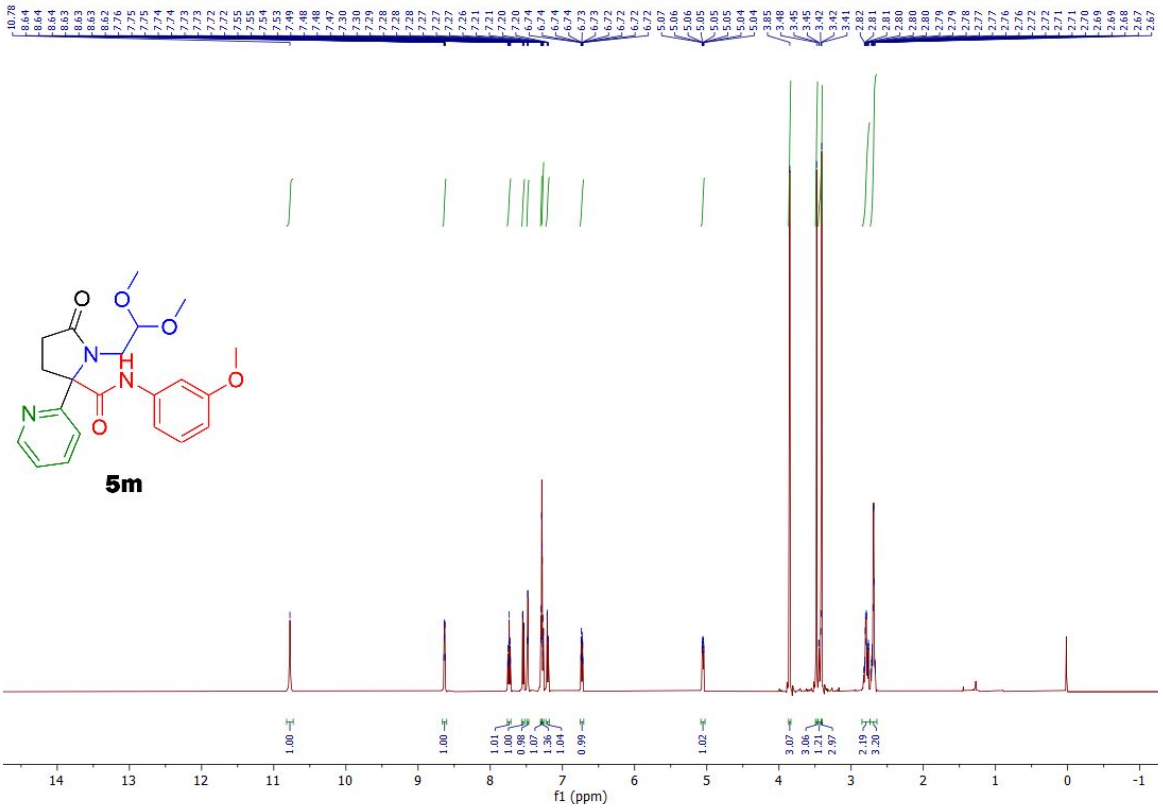

$^{13}C\{^{1}H\}$ NMR spectrum of **5m** (126 MHz, $CDCl_3$)

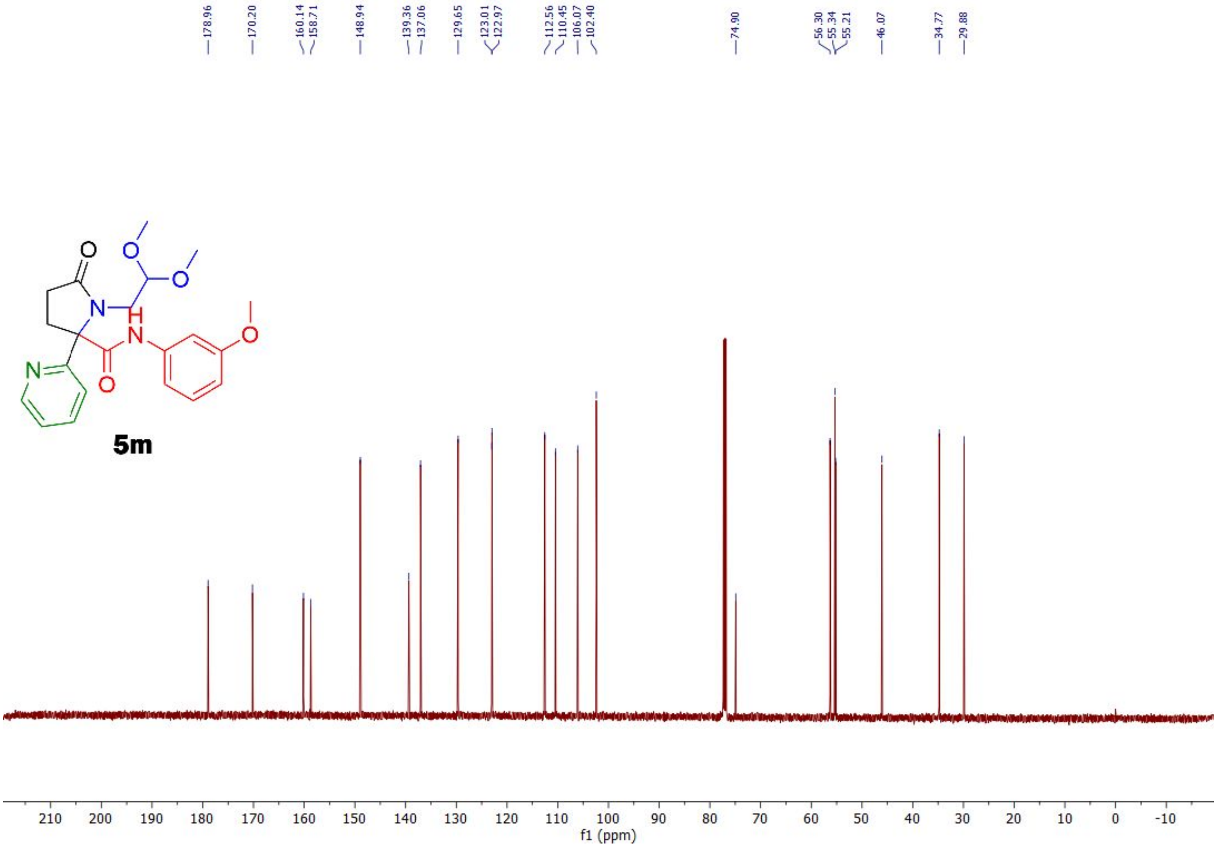

^1H NMR spectrum of **5n** (500 MHz, $CDCl_3$)

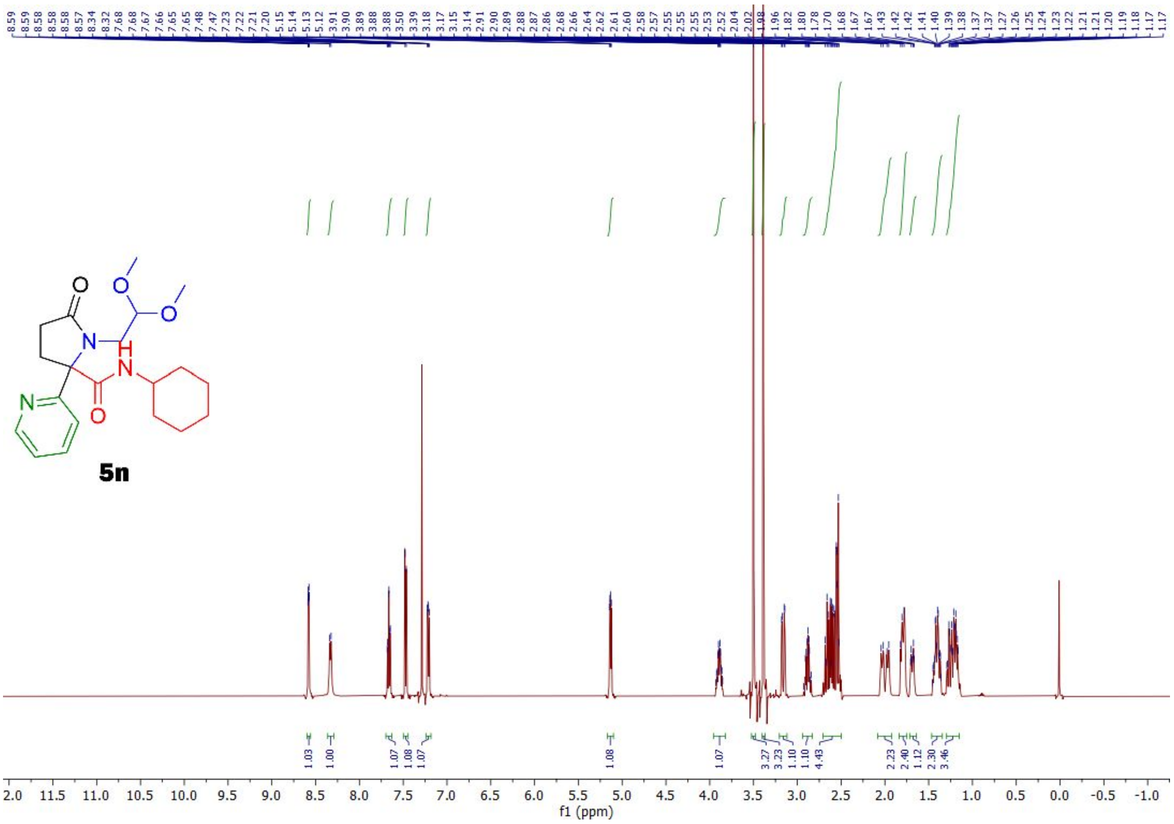

$^{13}C\{^1H\}$ NMR spectrum of **5n** (126 MHz, $CDCl_3$)

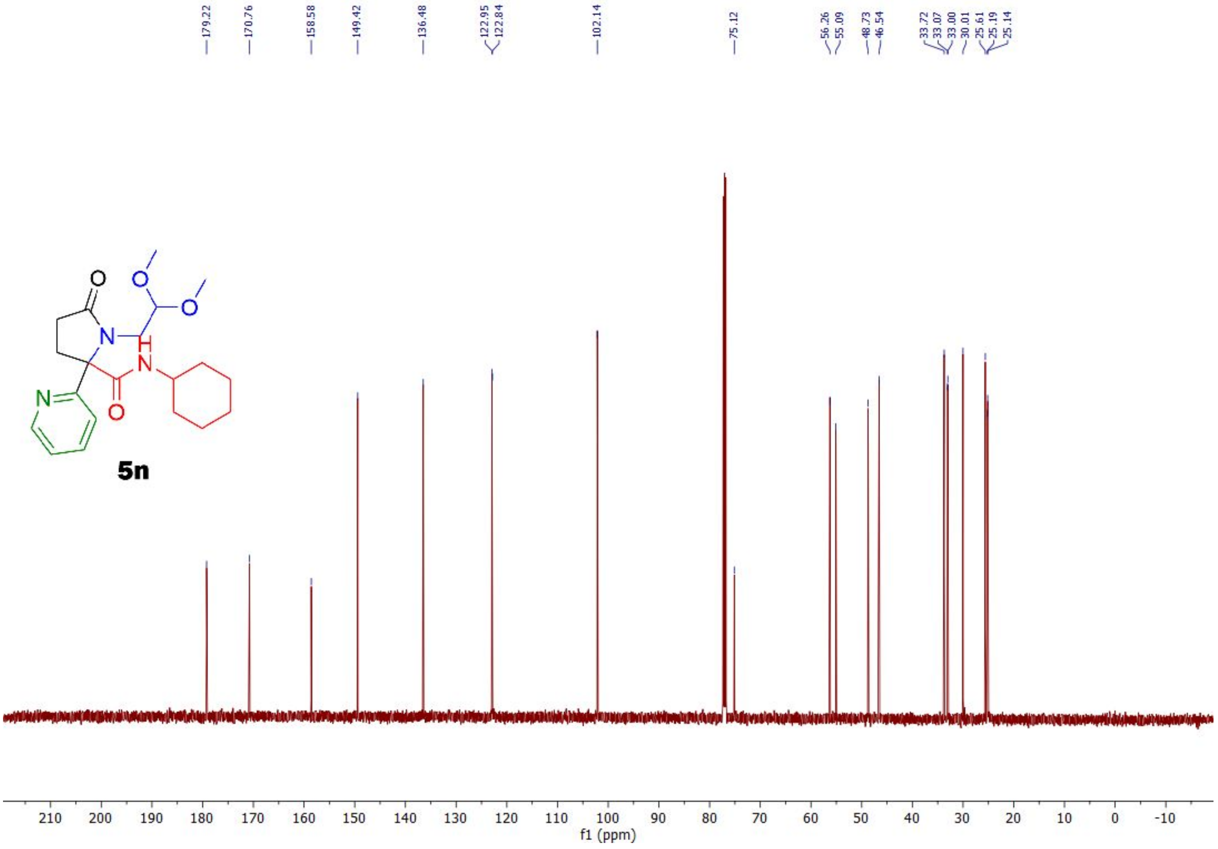

^{1}H NMR spectrum of **5o** (500 MHz, $CDCl_3$)

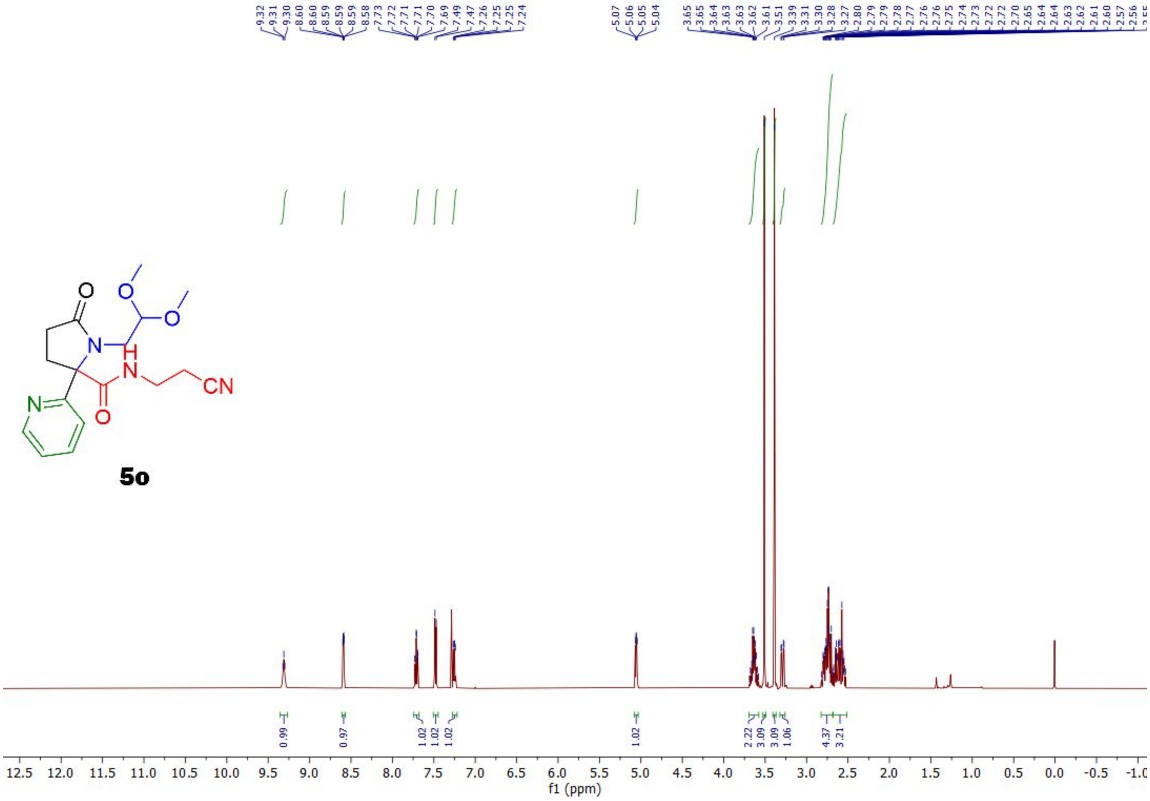

$^{13}C\{^{1}H\}$ NMR spectrum of **5o** (126 MHz, $CDCl_3$)

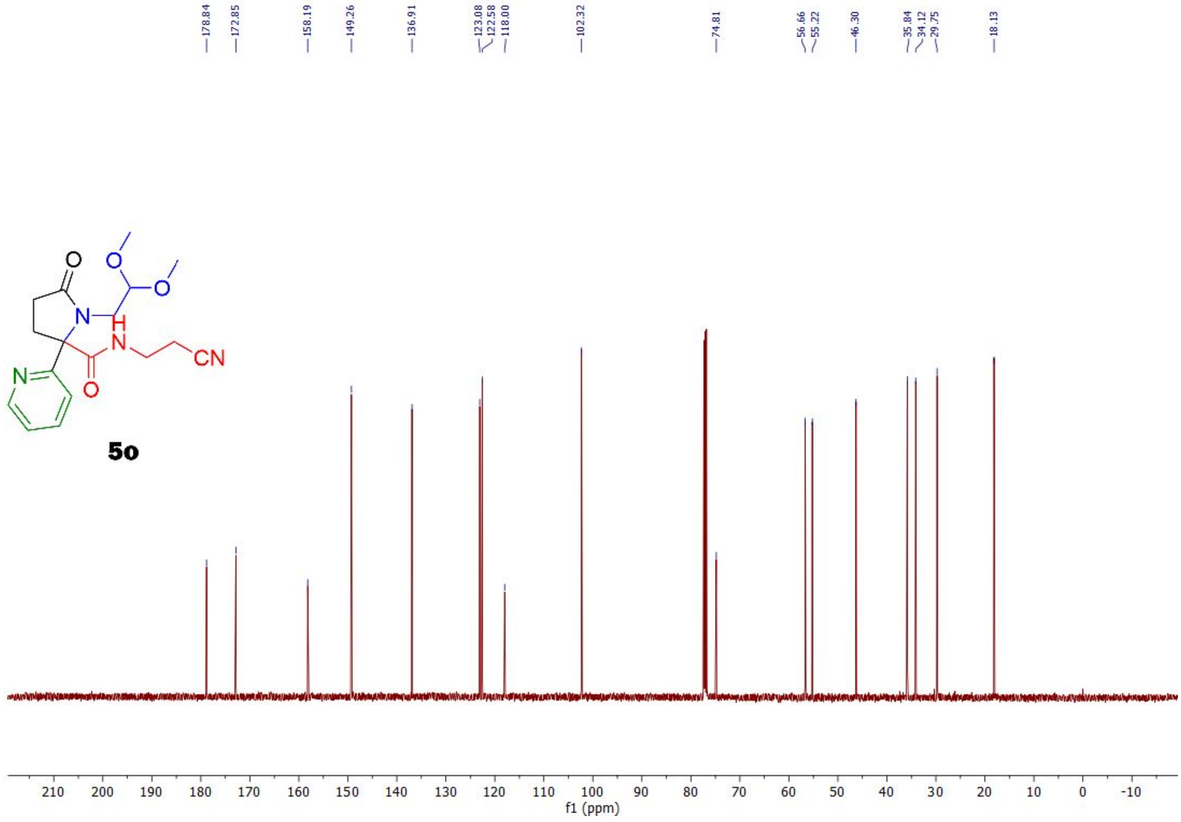

^{1}H NMR spectrum of **5p** (500 MHz, $CDCl_3$)

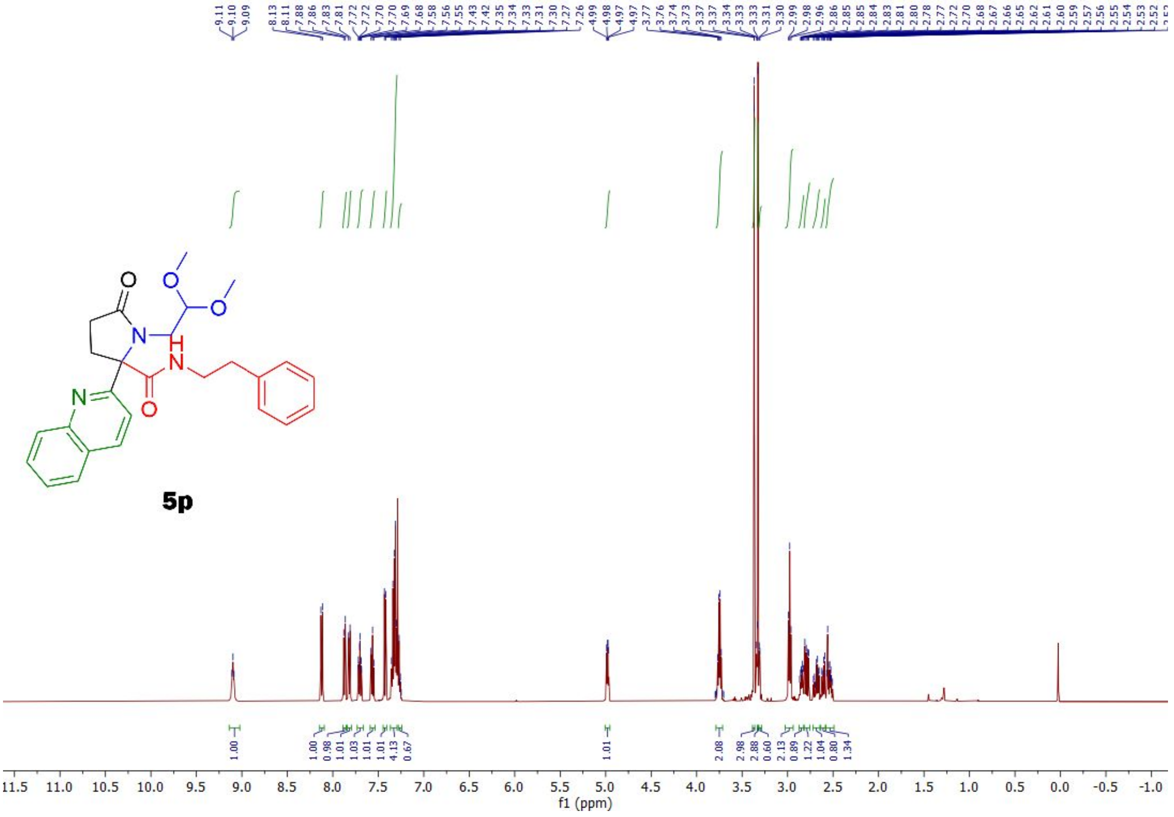

$^{13}C\{^{1}H\}$ NMR spectrum of **5p** (126 MHz, $CDCl_3$)

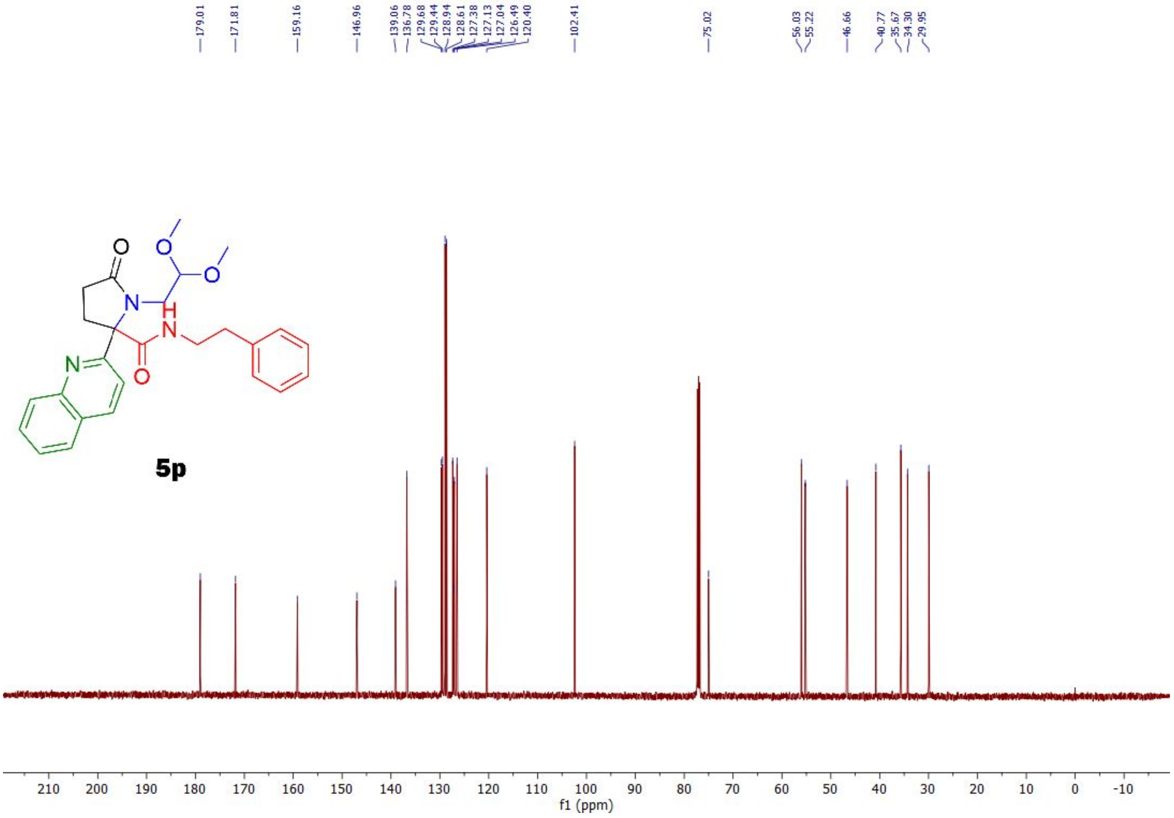

^{1}H NMR spectrum of **5q** (500 MHz, $CDCl_3$)

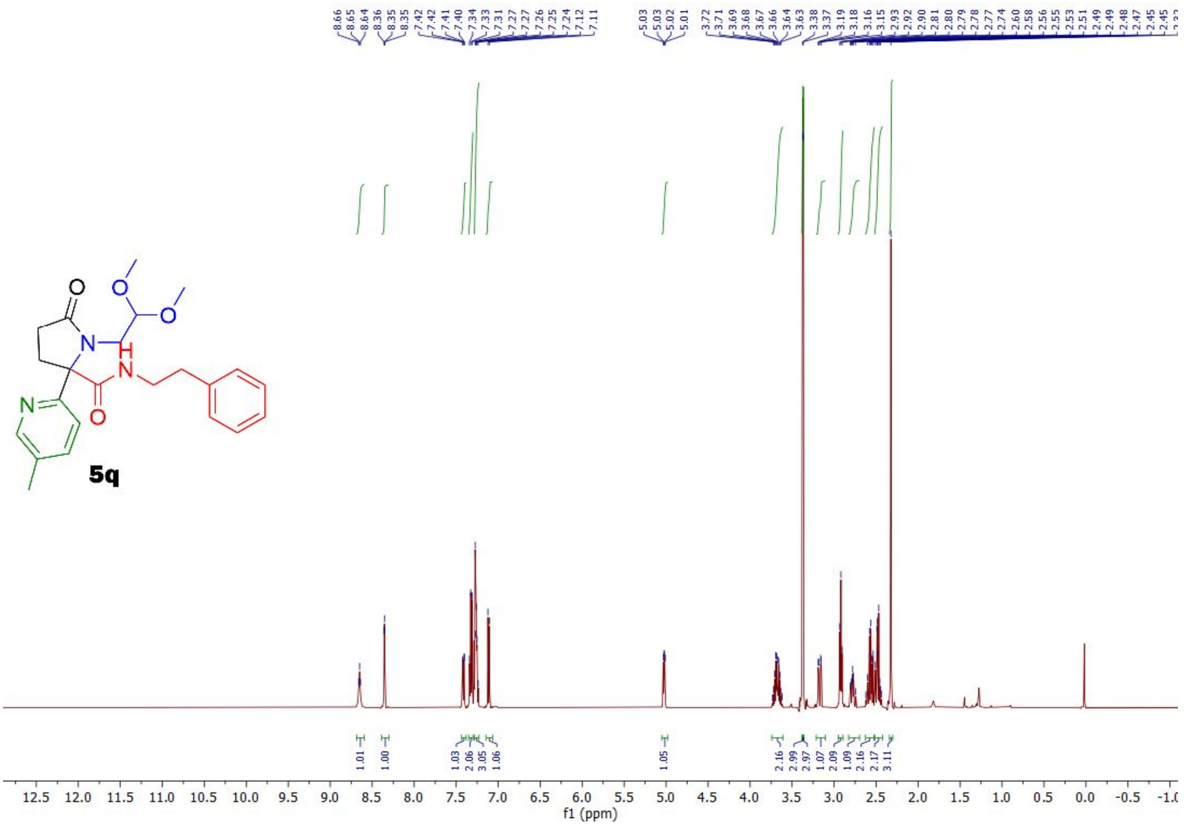

^{13}C{^{1}H} NMR spectrum of **5q** (126 MHz, $CDCl_3$)

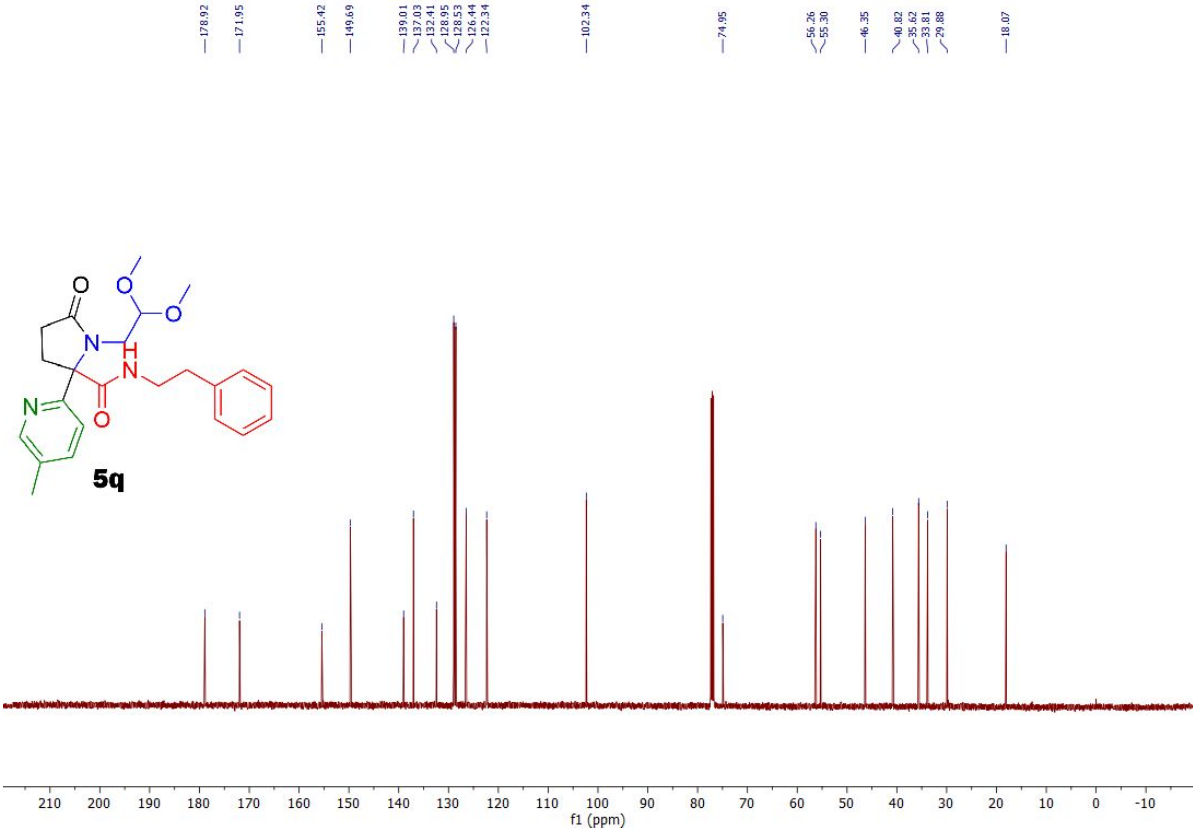

^{1}H NMR spectrum of **5r** (500 MHz, $CDCl_3$)

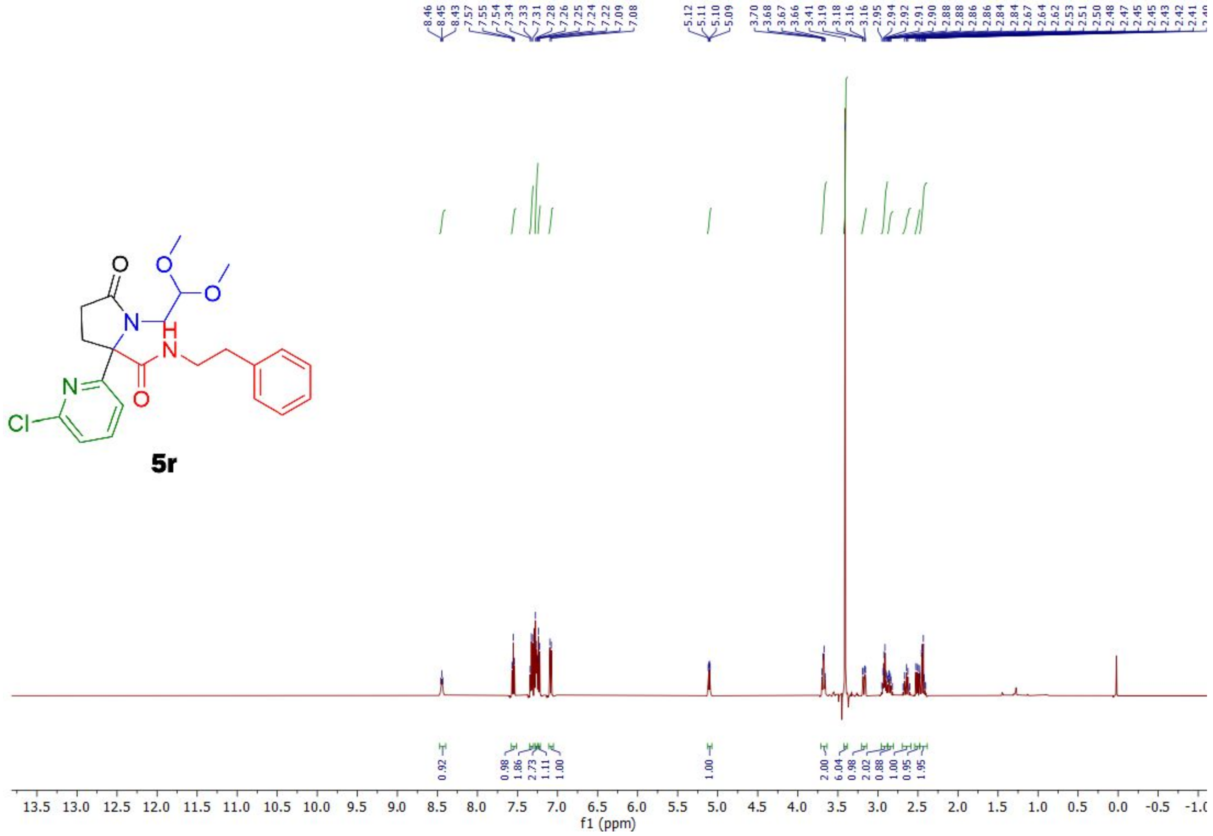

^{13}C{^{1}H} NMR spectrum of **5r** (126 MHz, $CDCl_3$)

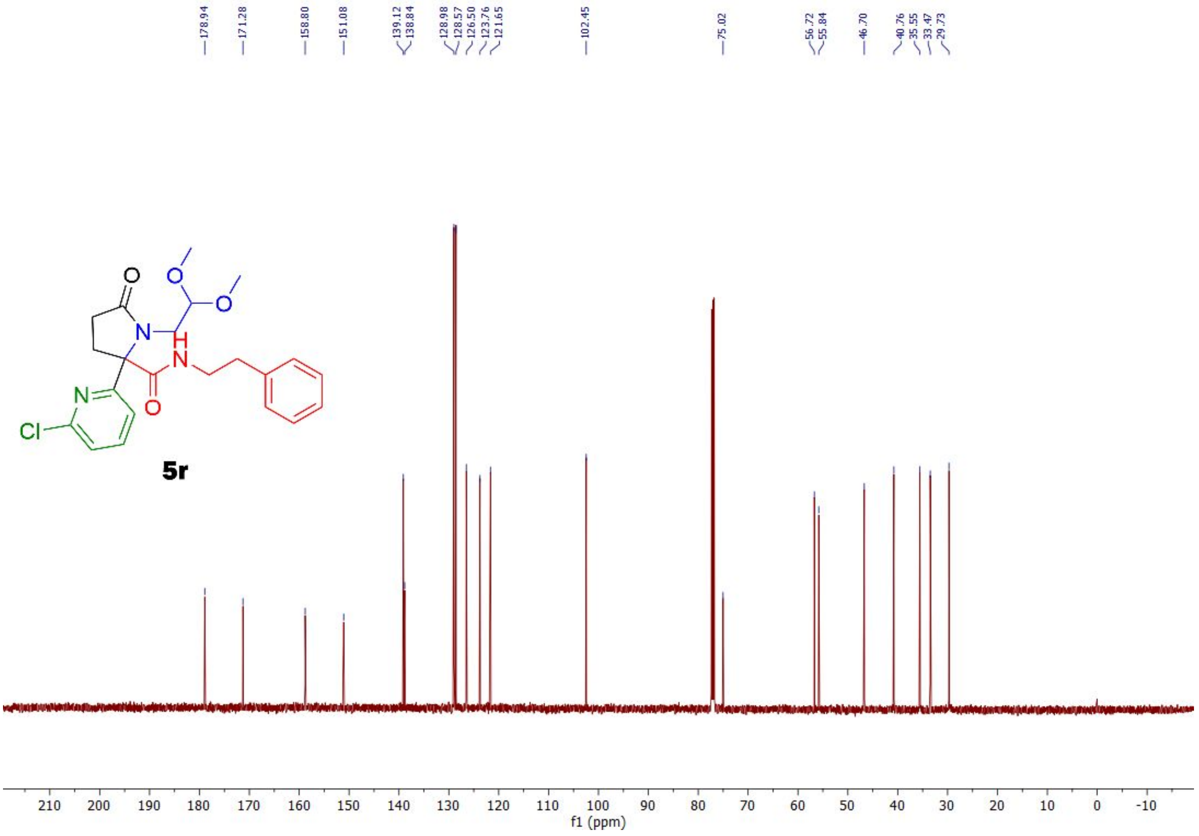

^{1}H NMR spectrum of **5s** (500 MHz, $CDCl_3$)

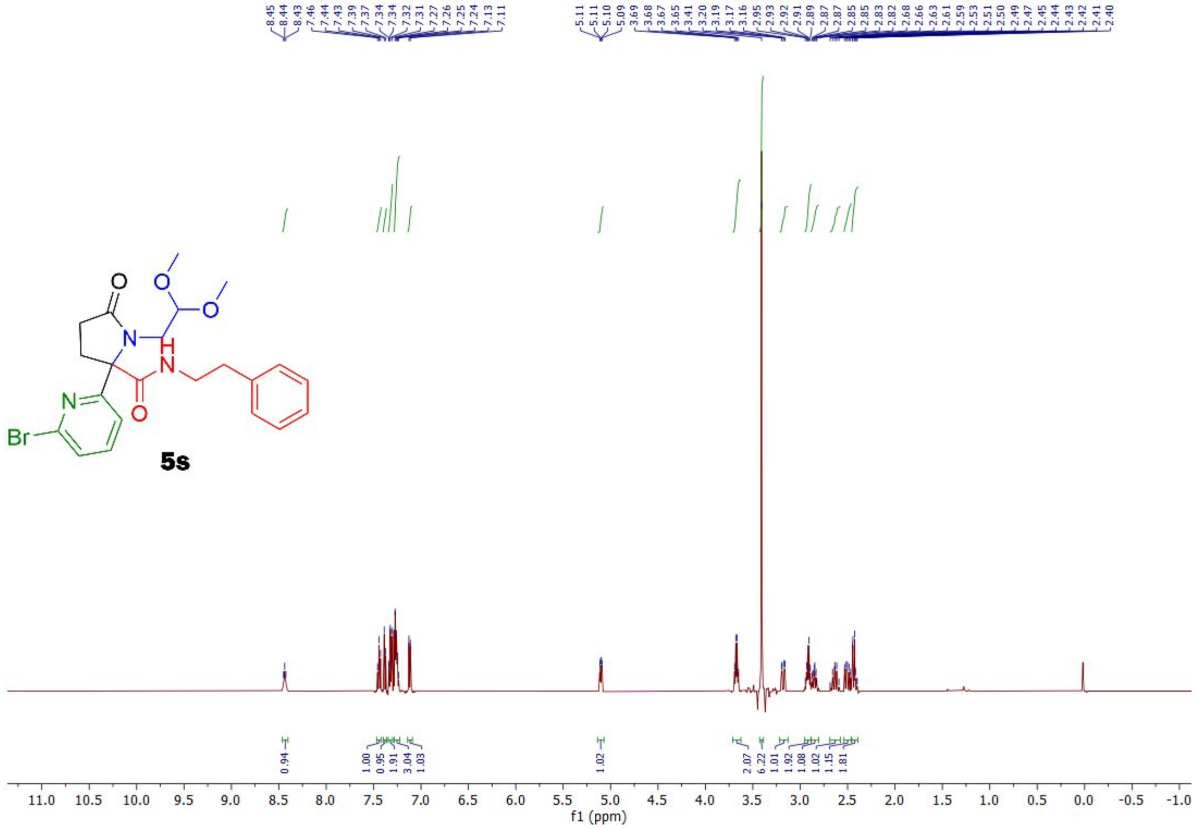

^{13}C{^{1}H} NMR spectrum of **5s** (126 MHz, $CDCl_3$)

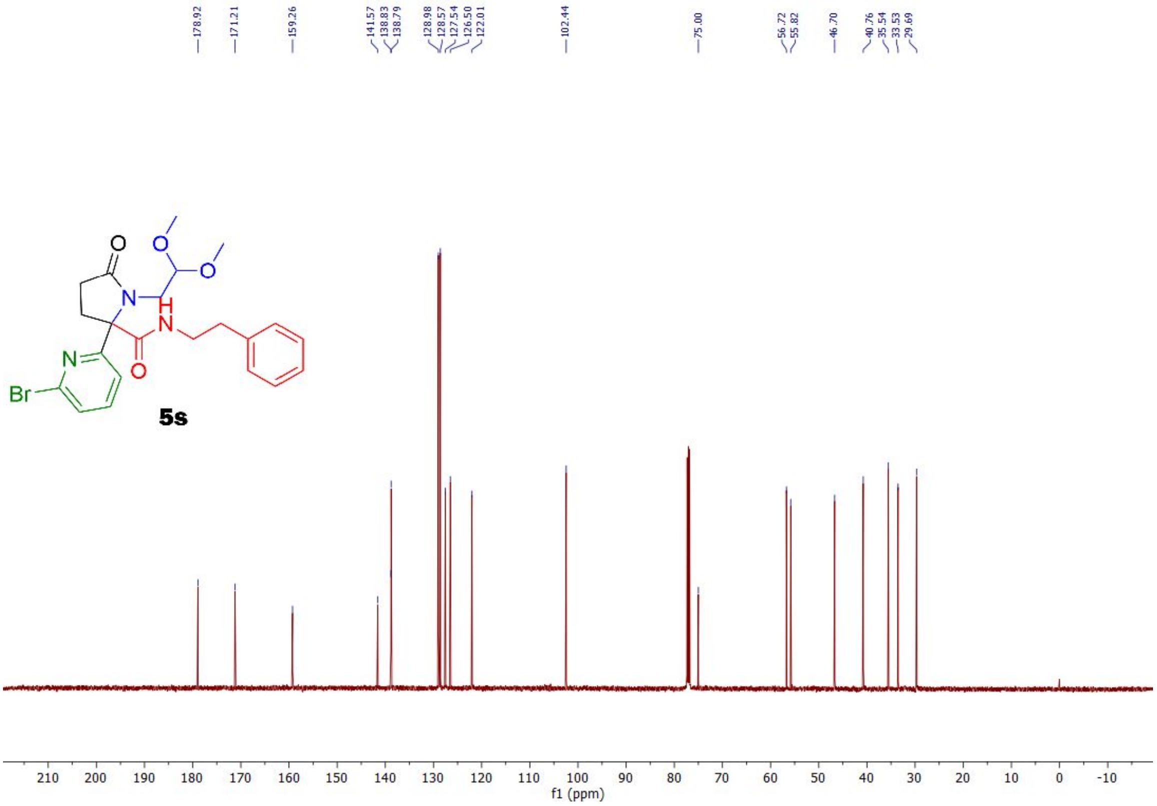

^{1}H NMR spectrum of **5t** (500 MHz, $CDCl_3$)

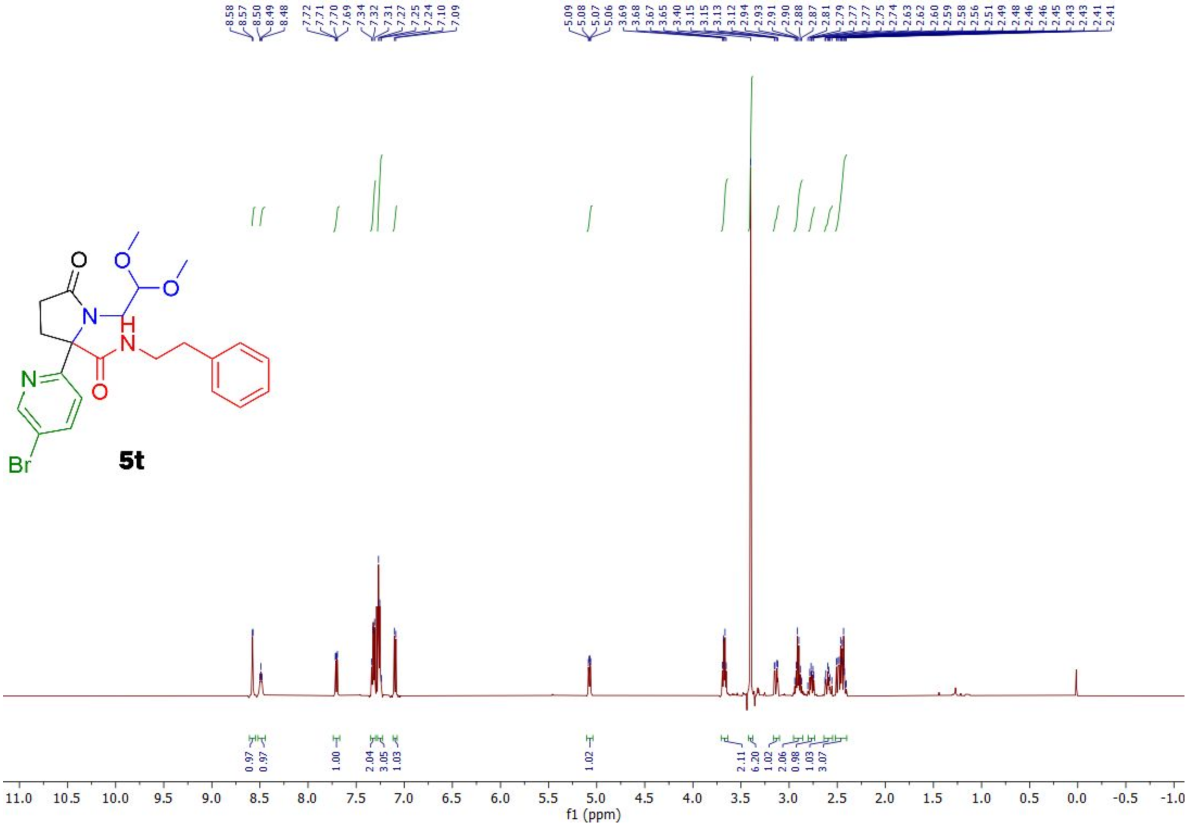

$^{13}C\{^{1}H\}$ NMR spectrum of **5t** (126 MHz, $CDCl_3$)

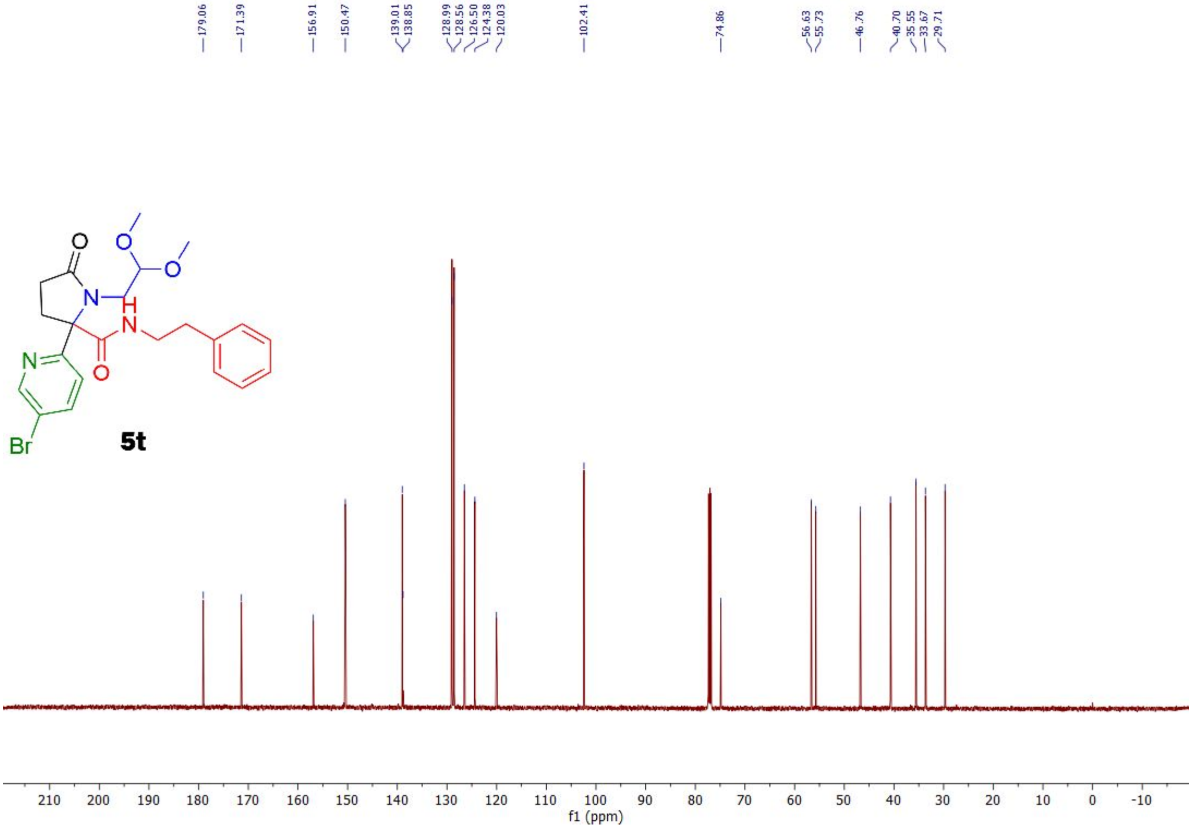

^{1}H NMR spectrum of **5u** (500 MHz, $CDCl_3$)

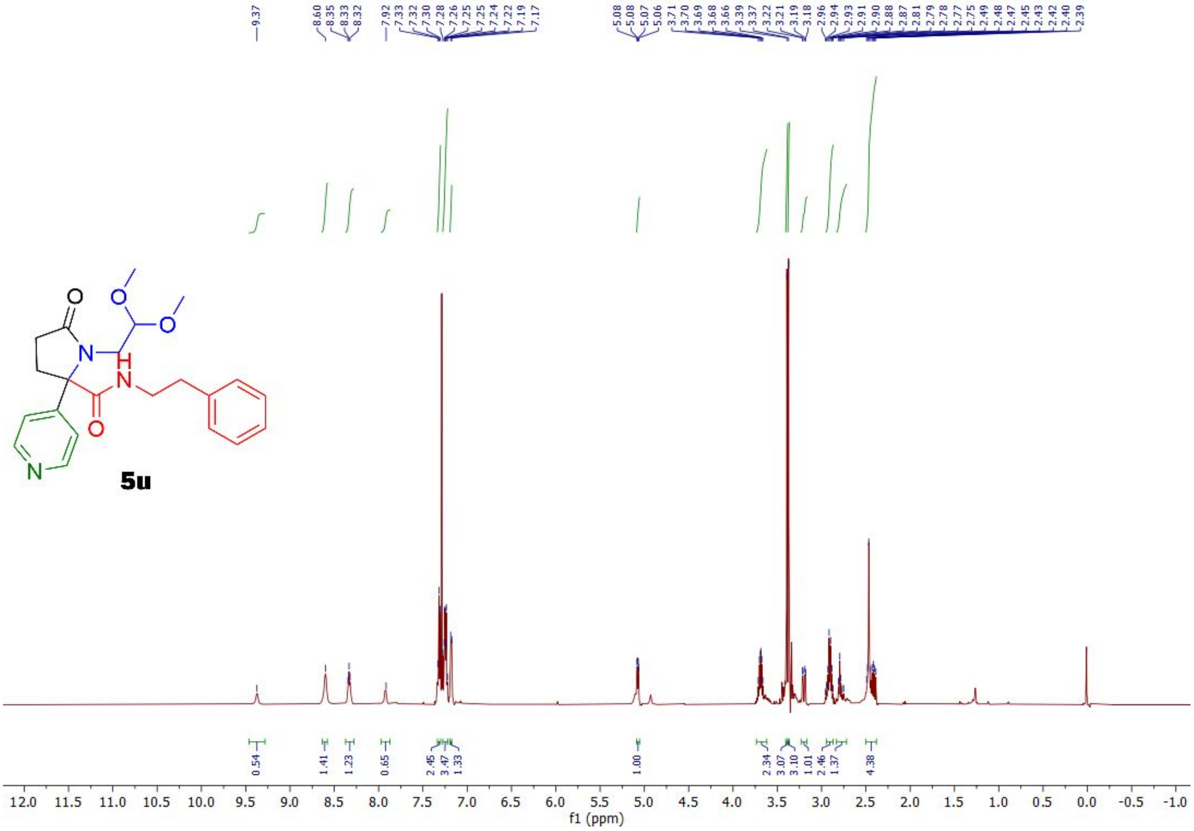

^{13}C{^{1}H} NMR spectrum of **5u** (126 MHz, $CDCl_3$)

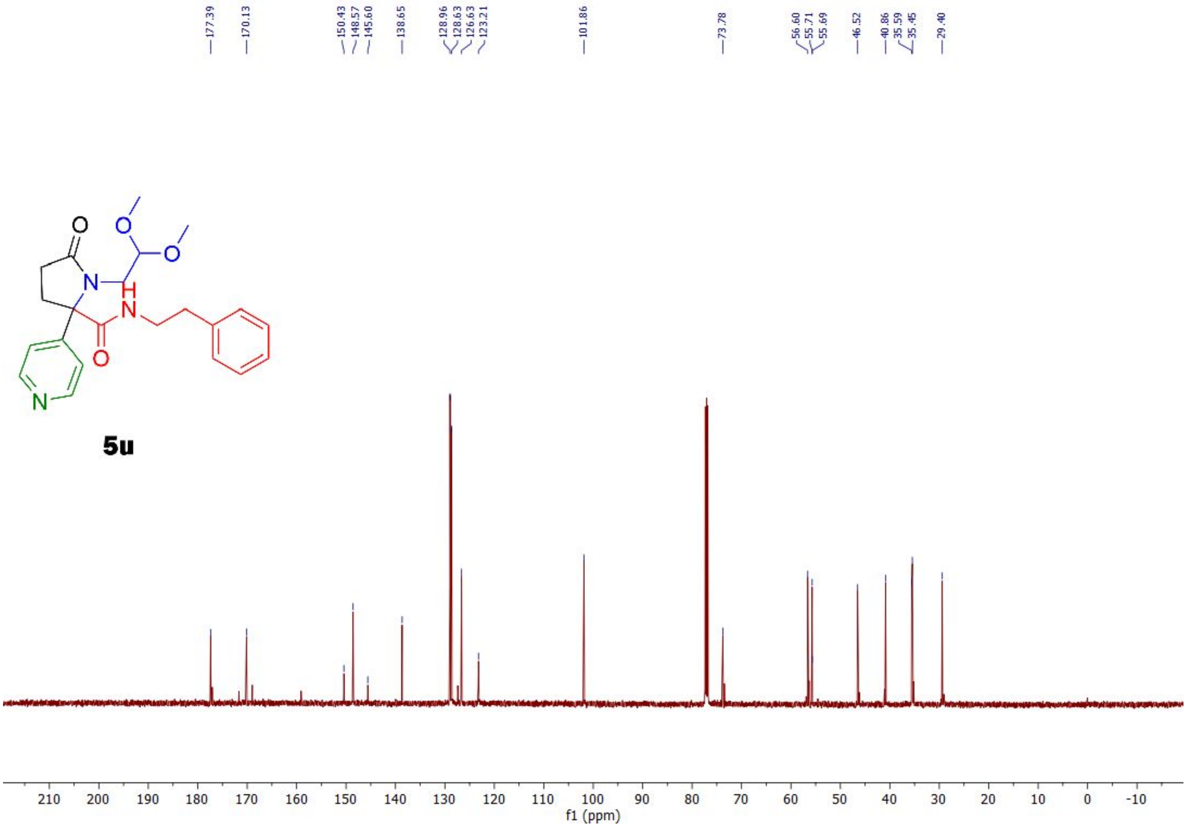

^{1}H NMR spectrum of **5v** (500 MHz, $CDCl_3$)

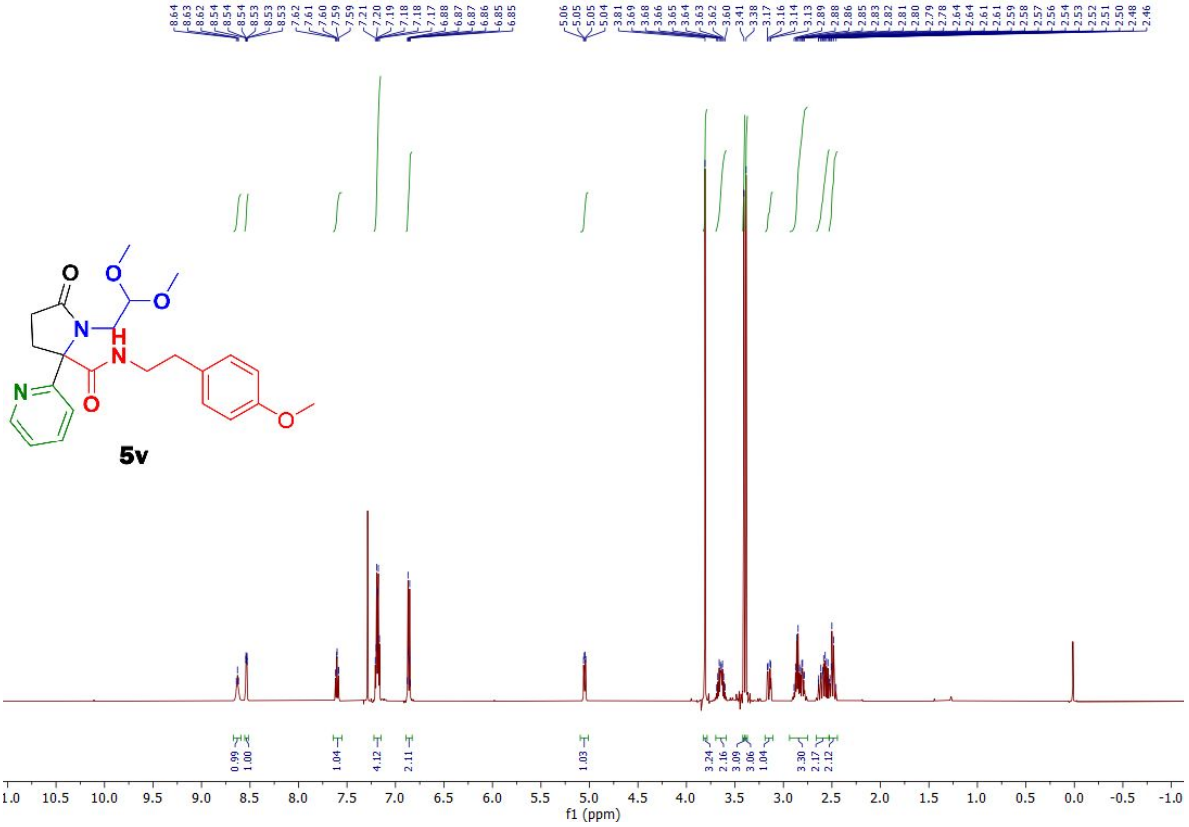

$^{13}C\{^{1}H\}$ NMR spectrum of **5v** (126 MHz, $CDCl_3$)

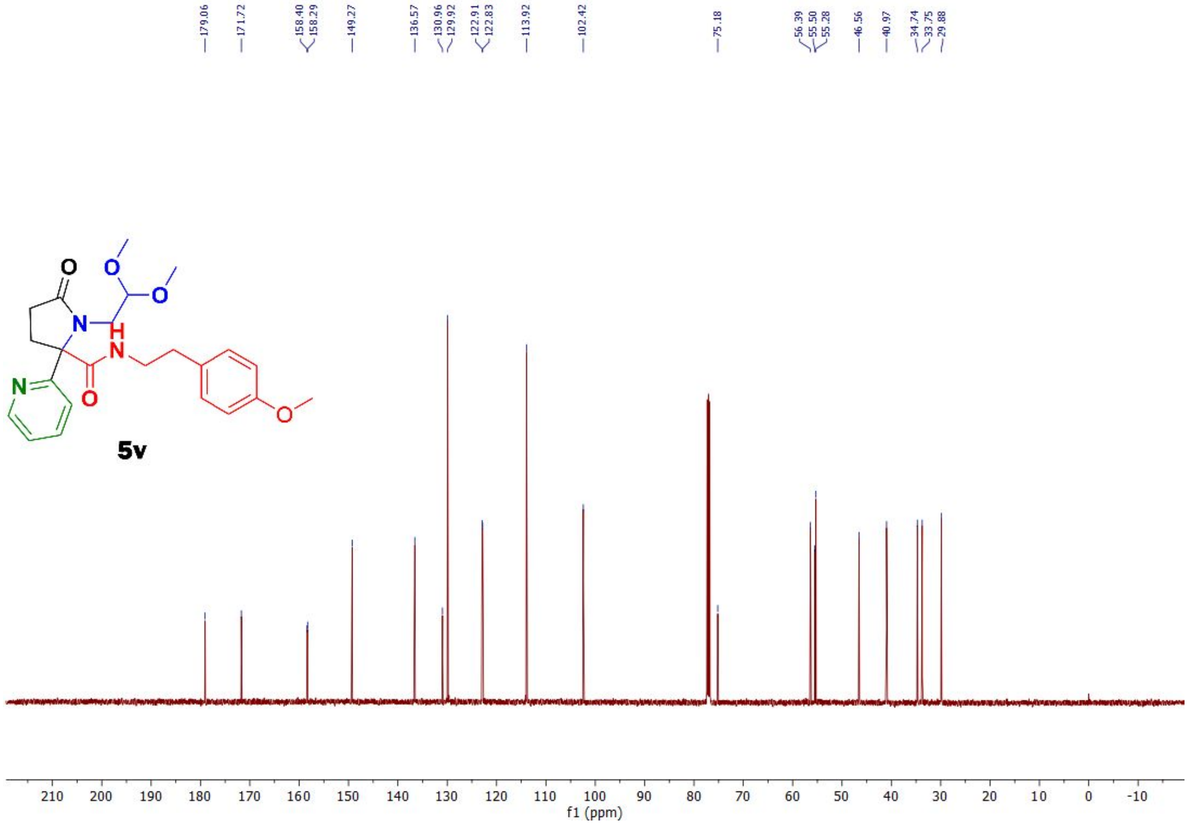

^{1}H NMR spectrum of **5w** (500 MHz, $CDCl_3$)

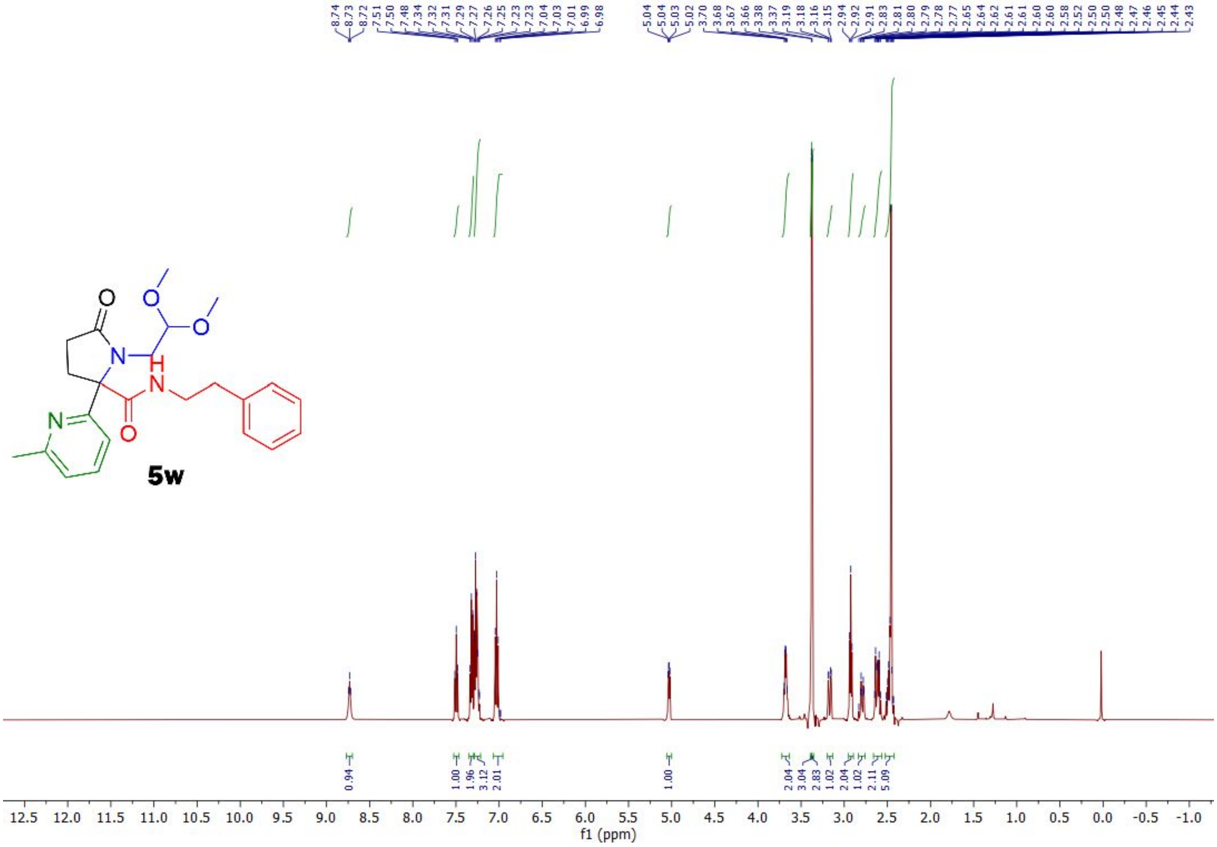

$^{13}C\{^{1}H\}$ NMR spectrum of **5w** (126 MHz, $CDCl_3$)

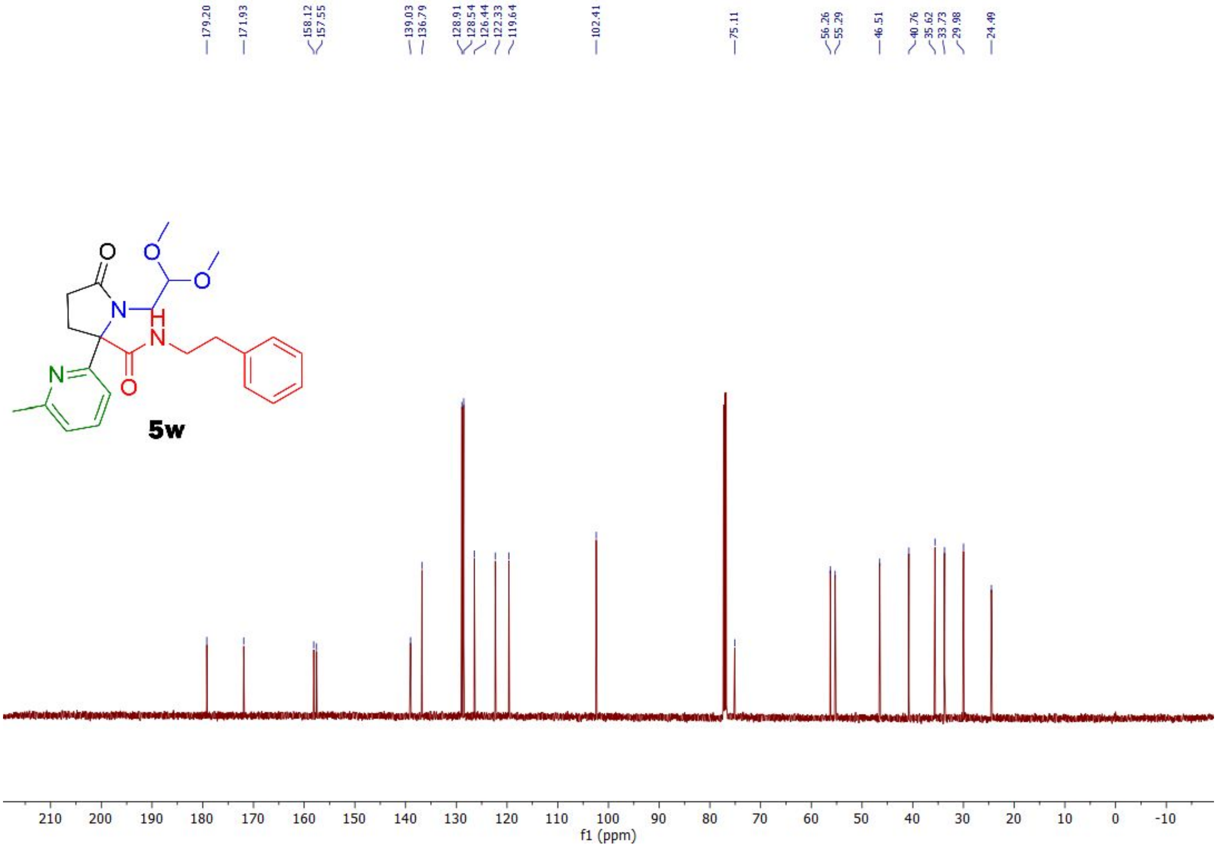

^{1}H NMR spectrum of **5x** (500 MHz, $CDCl_3$)

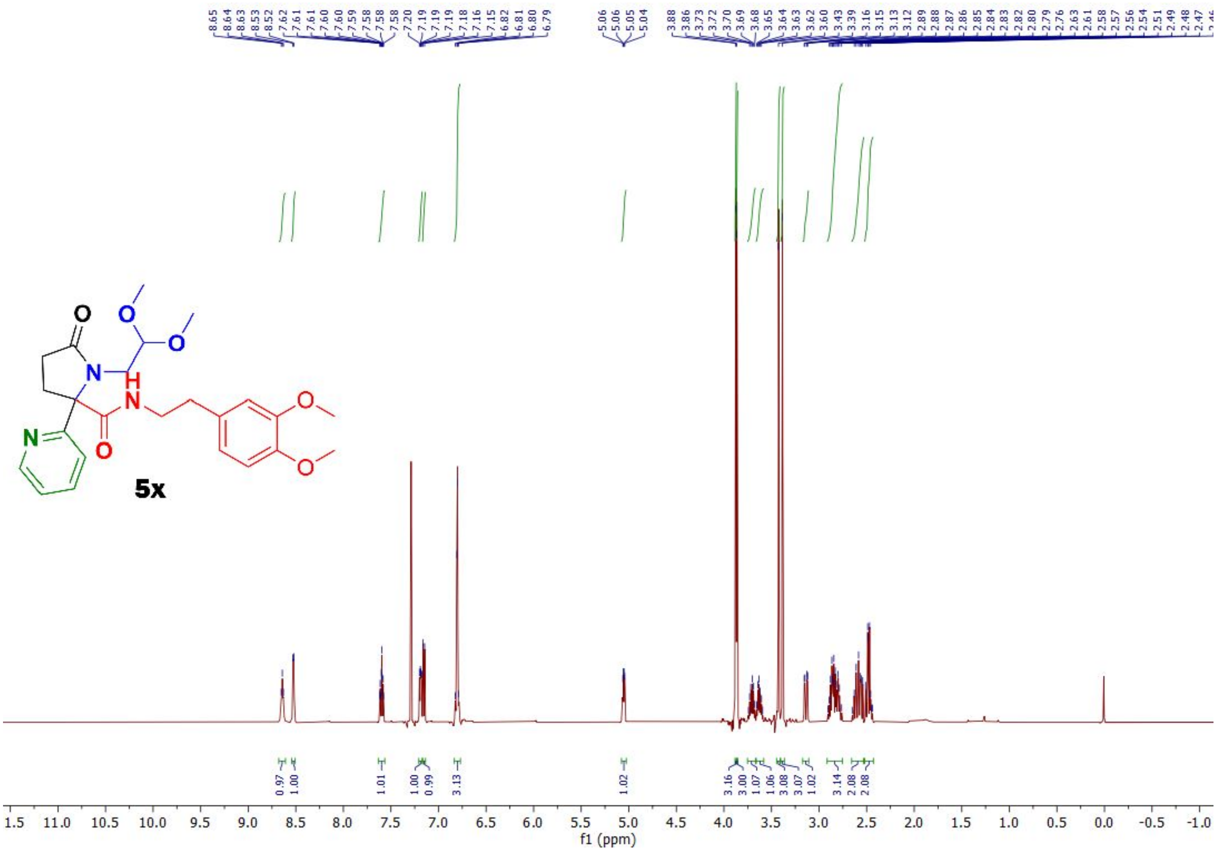

$^{13}C\{^{1}H\}$ NMR spectrum of **5x** (126 MHz, $CDCl_3$)

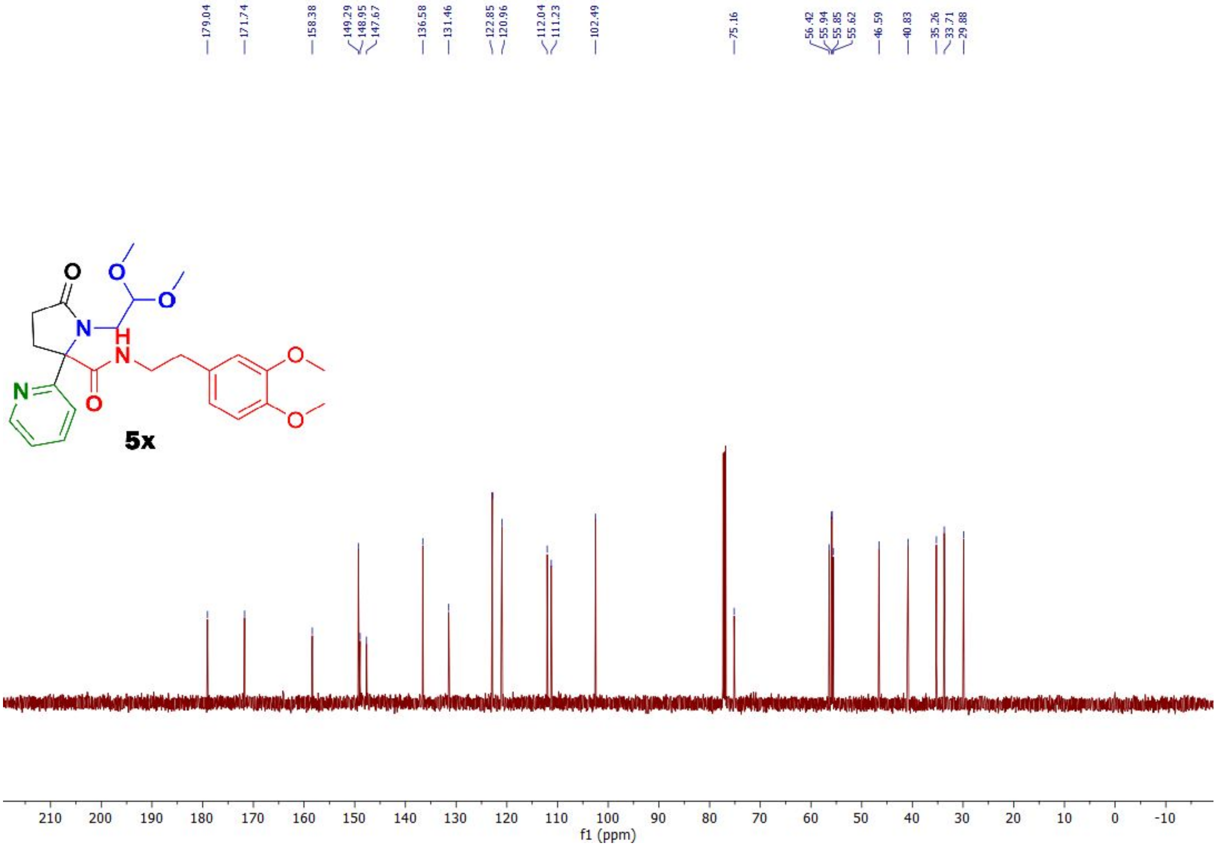

^{1}H NMR spectrum of **5y** (500 MHz, $CDCl_3$)

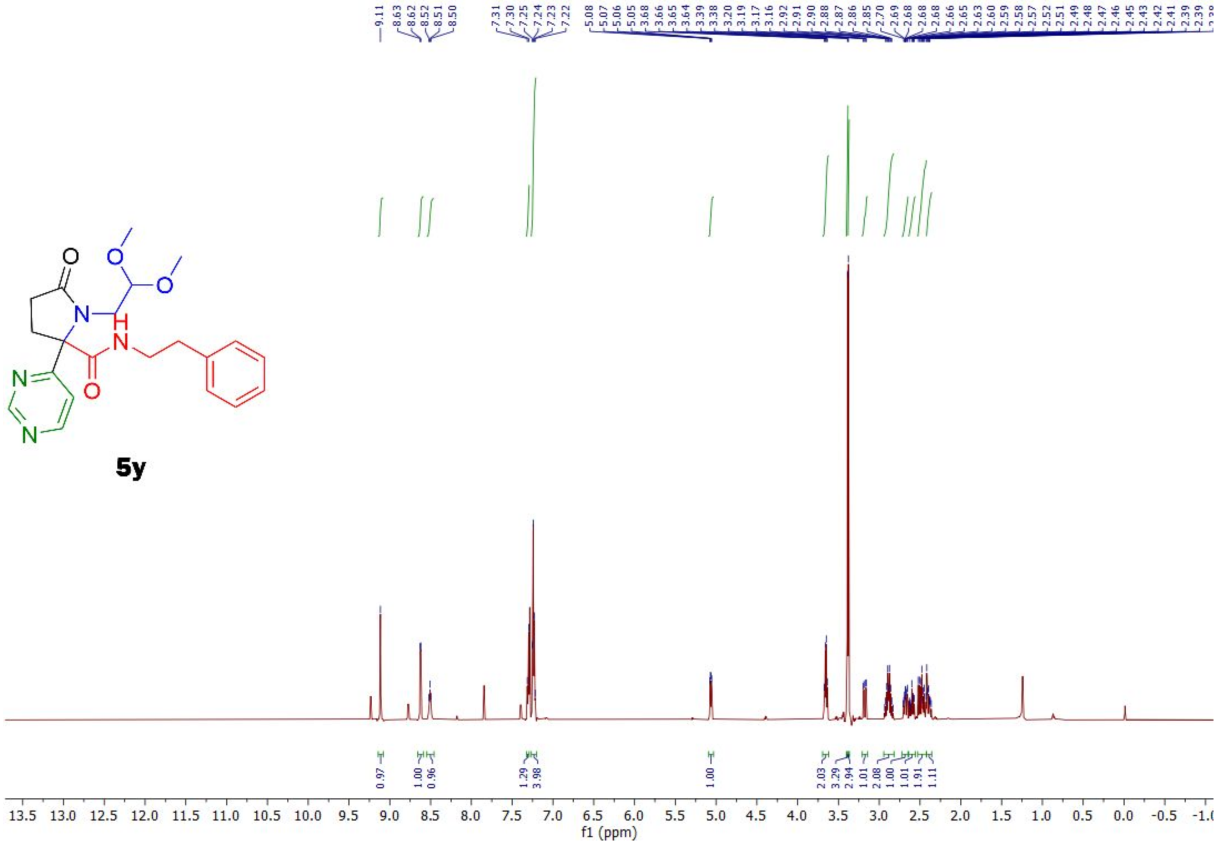

$^{13}C\{^{1}H\}$ NMR spectrum of **5y** (126 MHz, $CDCl_3$)

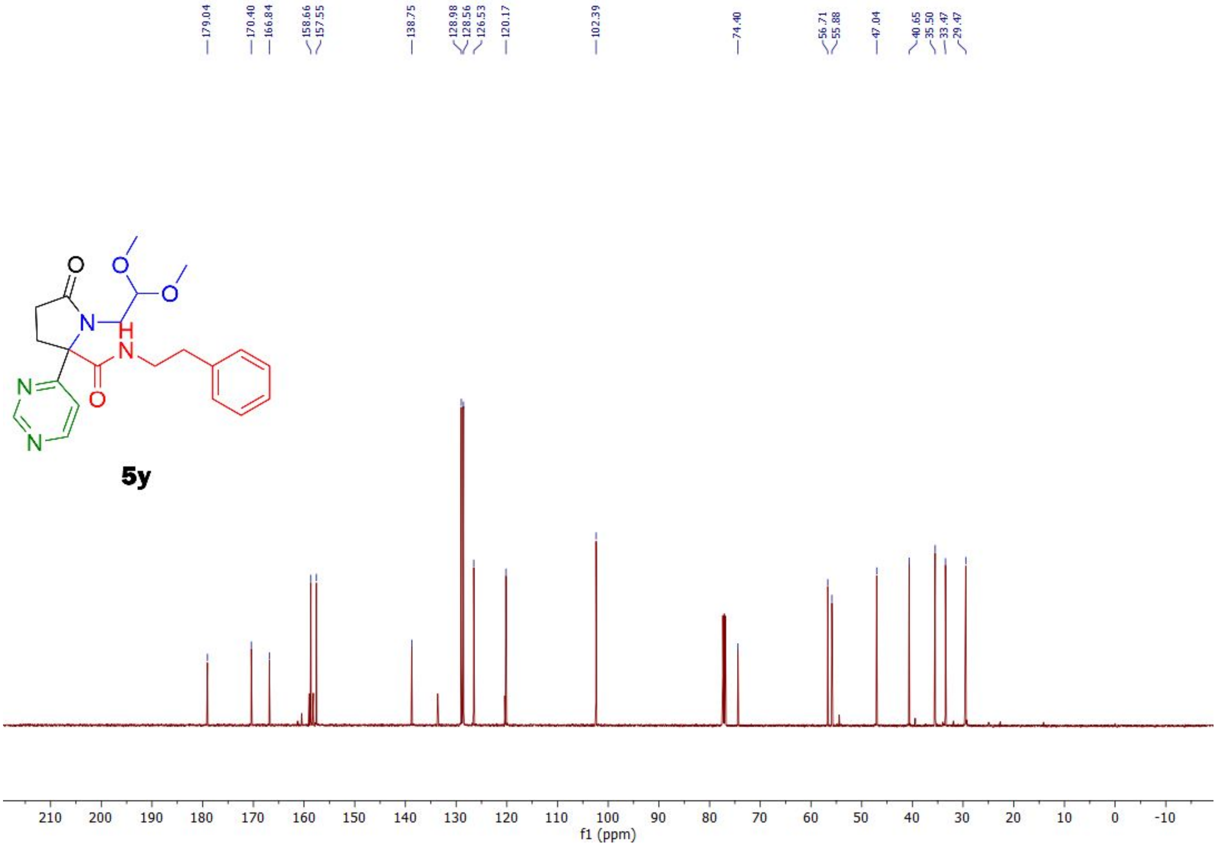

^{1}H NMR spectrum of **6a** (500 MHz, $CDCl_3$)

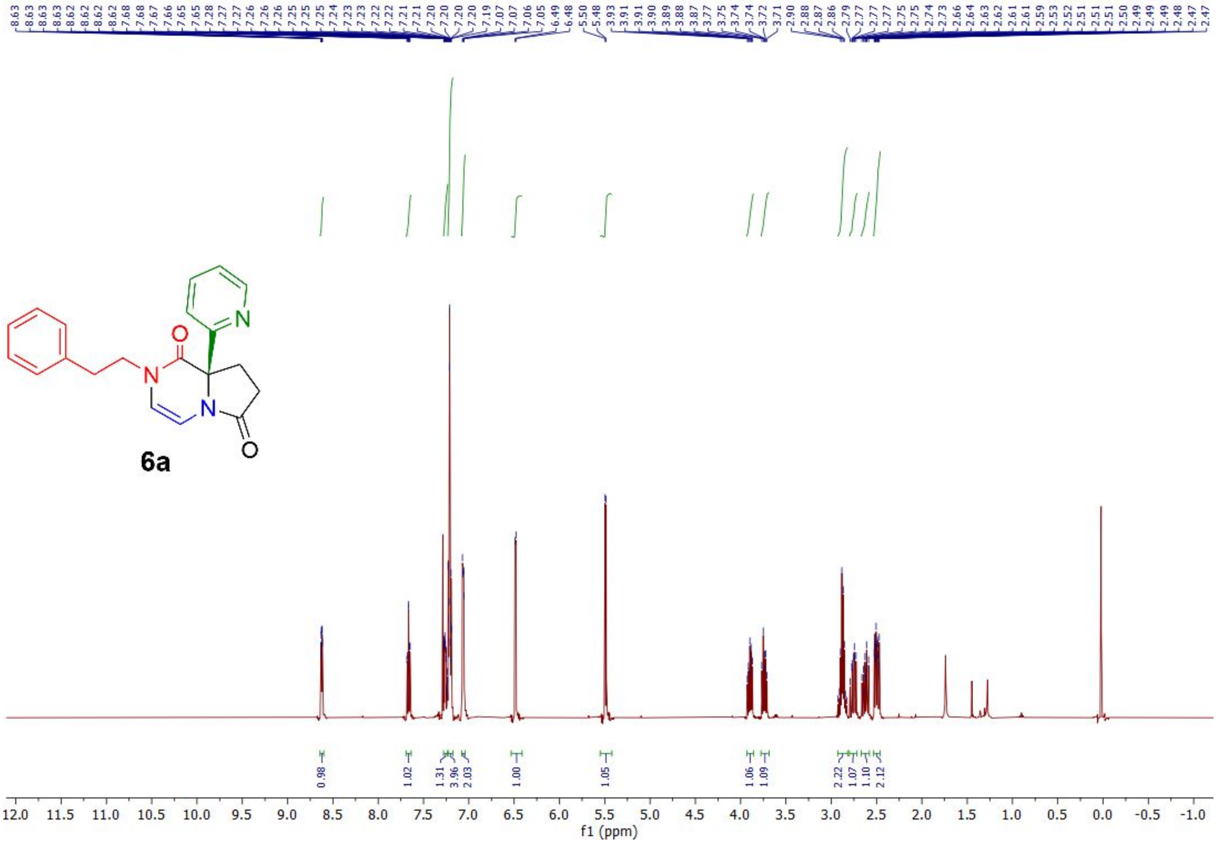

^{13}C{^{1}H} NMR spectrum of **6a** (126 MHz, $CDCl_3$)

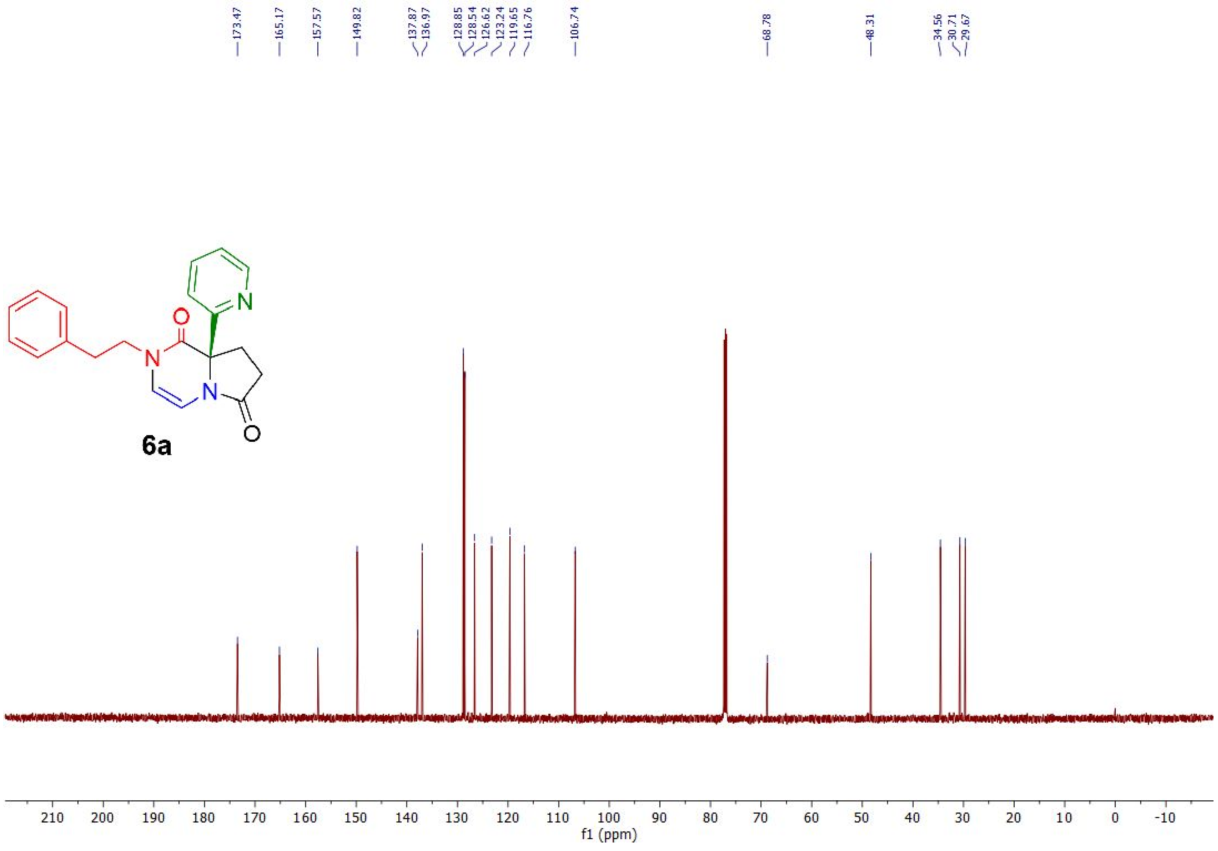

^{1}H NMR spectrum of **6b** (500 MHz, $CDCl_3$)

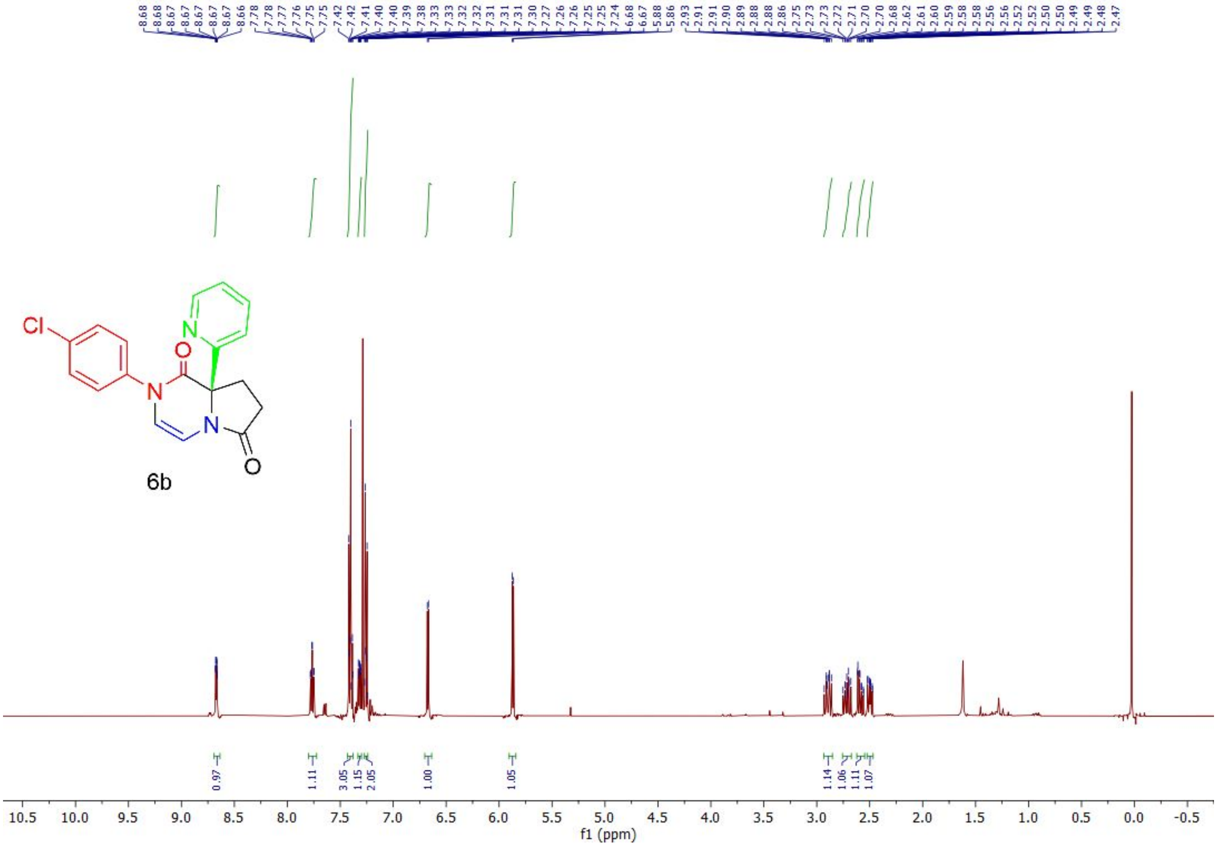

^{13}C{^{1}H} NMR spectrum of **6b** (126 MHz, $CDCl_3$)

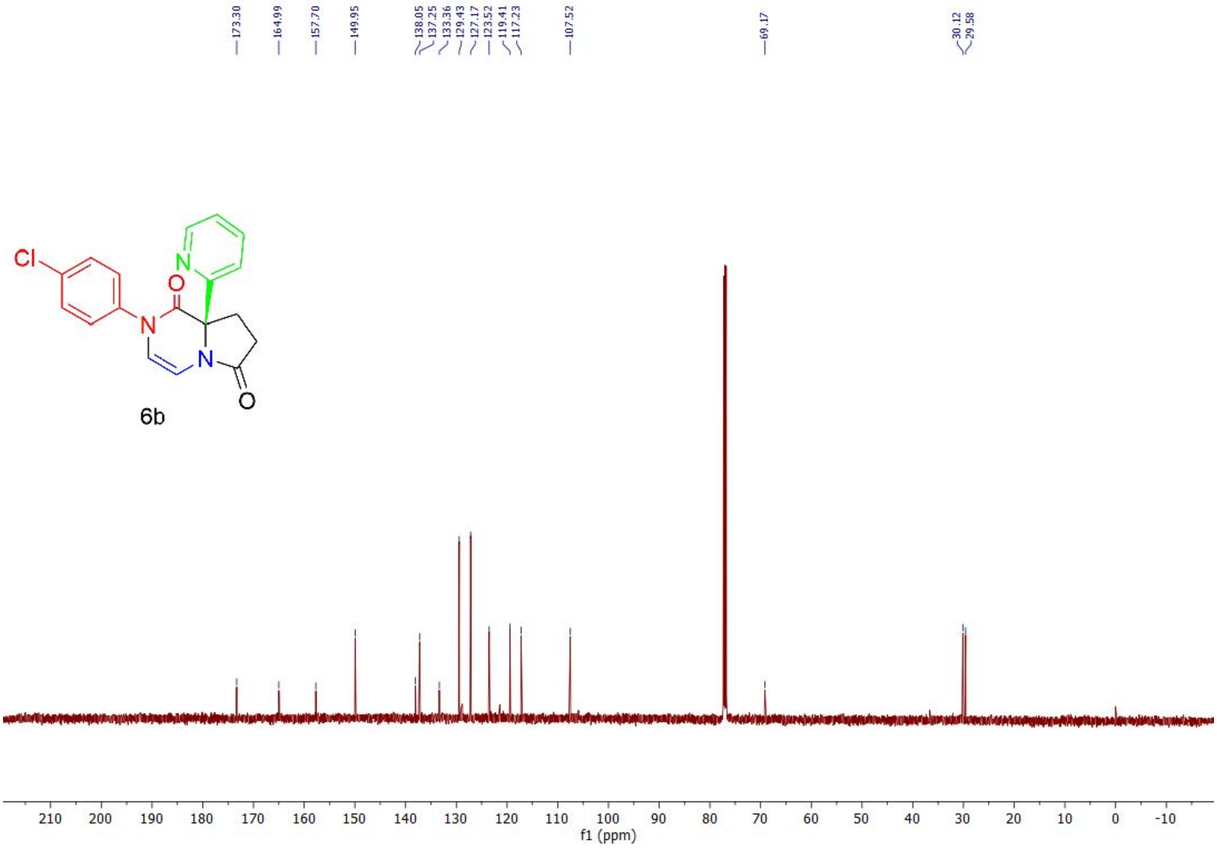

^{1}H NMR spectrum of **6c** (500 MHz, $CDCl_3$)

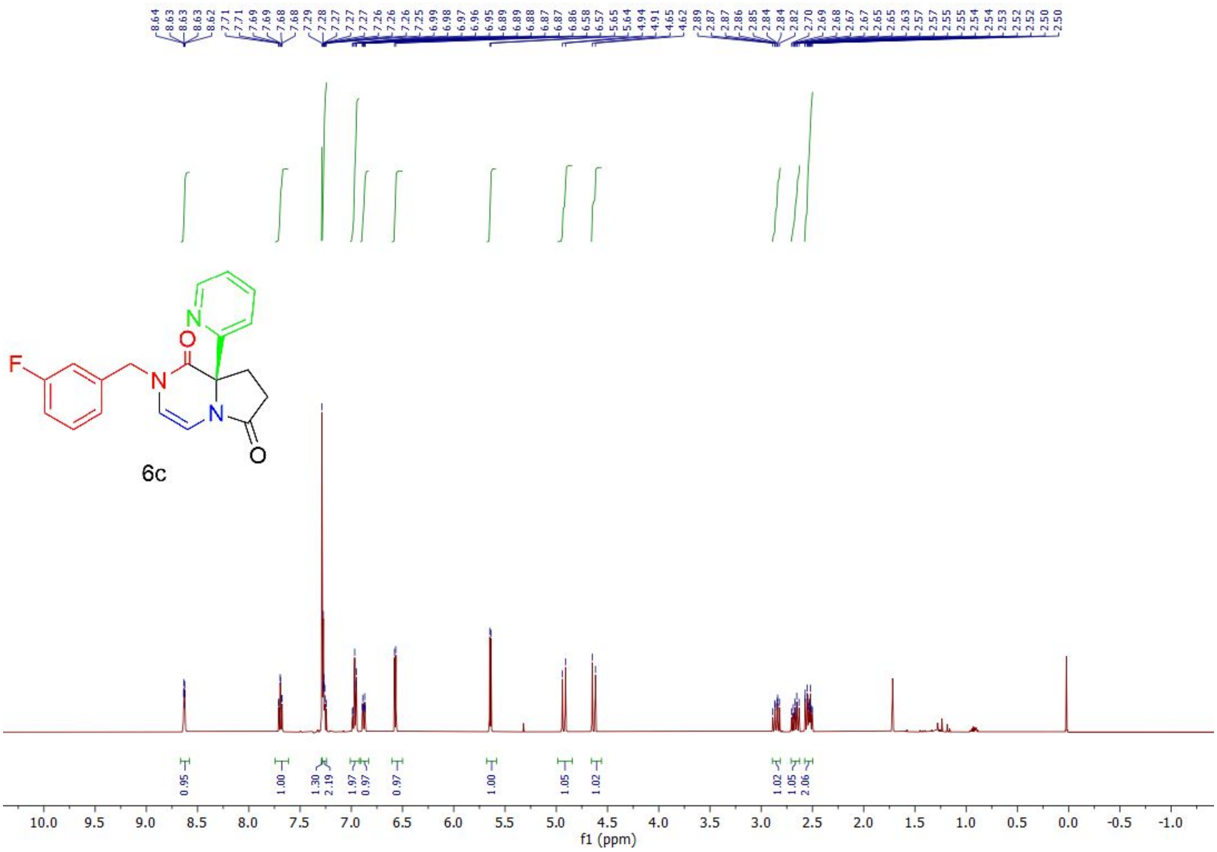

$^{13}C\{^{1}H\}$ NMR spectrum of **6c** (126 MHz, $CDCl_3$)

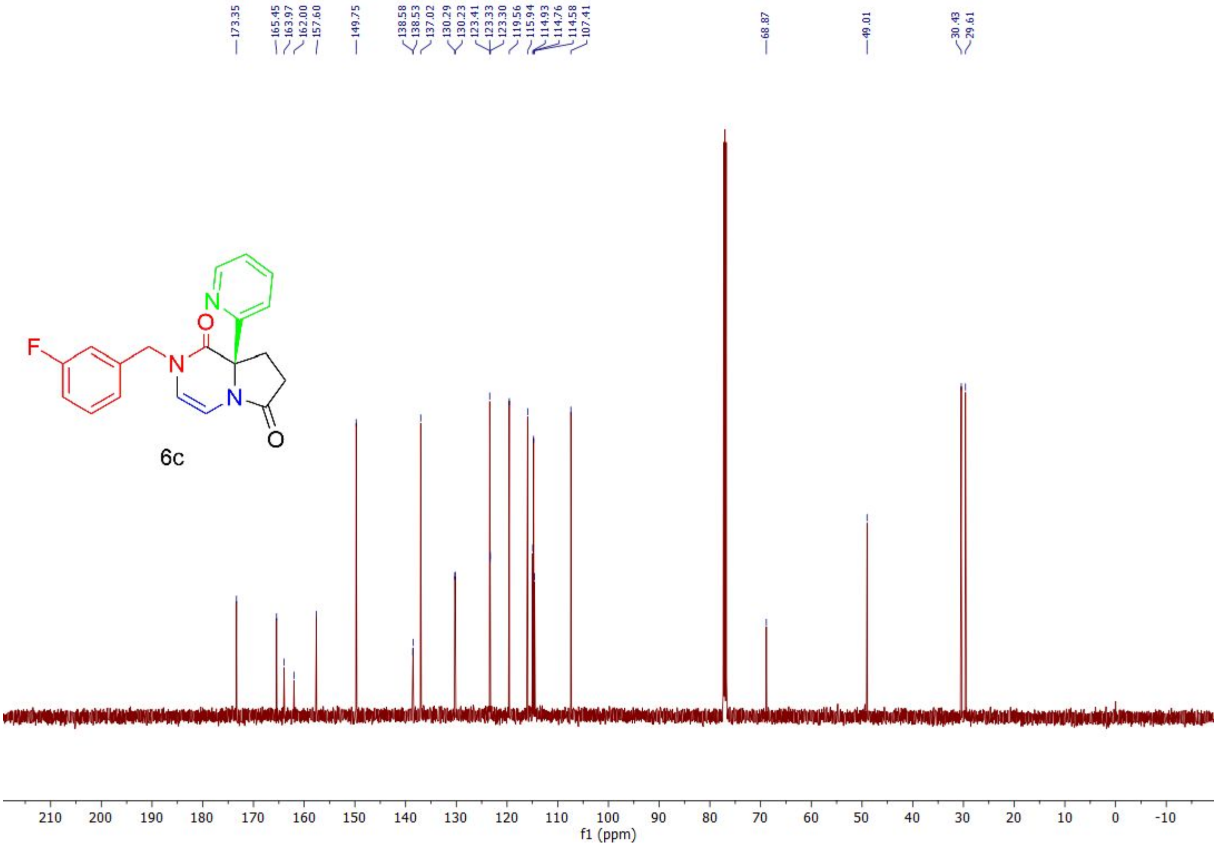

^{1}H NMR spectrum of **6d** (500 MHz, $CDCl_3$)

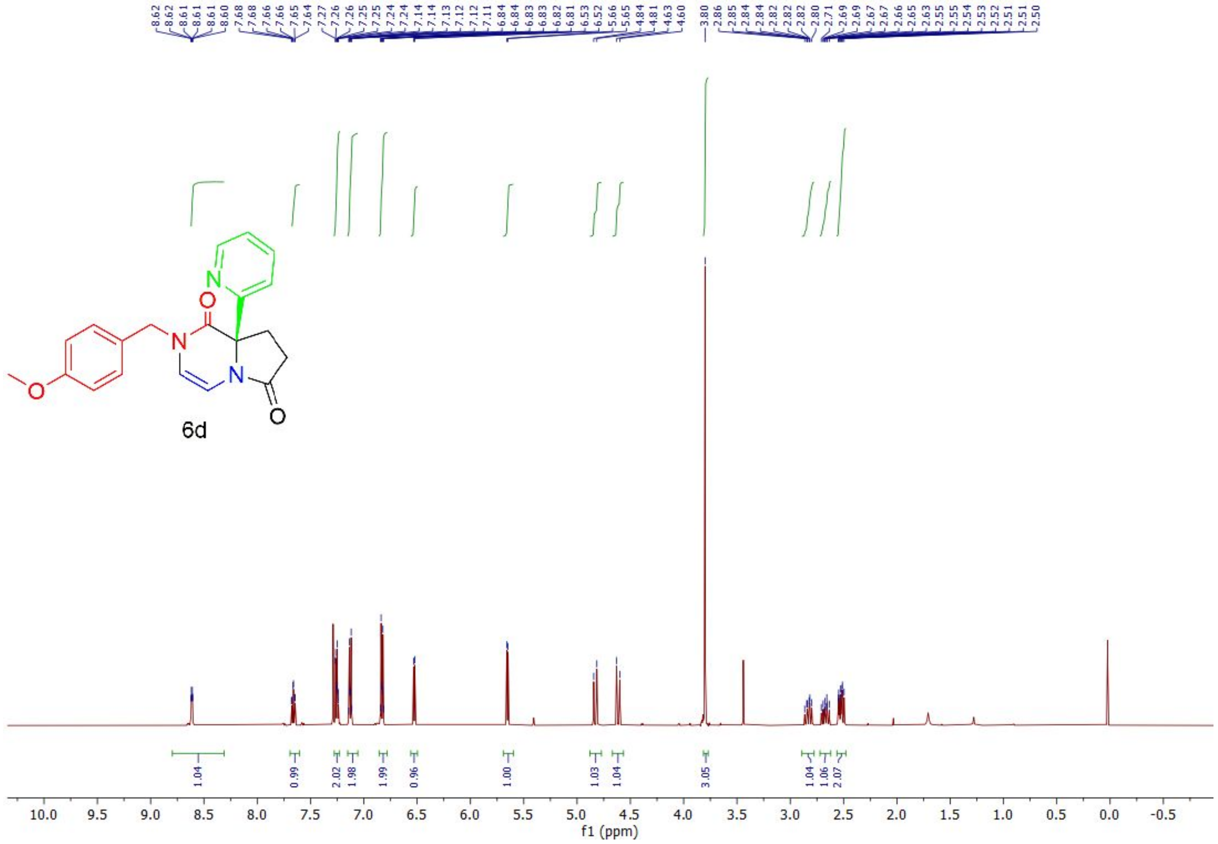

$^{13}C\{^{1}H\}$ NMR spectrum of **6d** (126 MHz, $CDCl_3$)

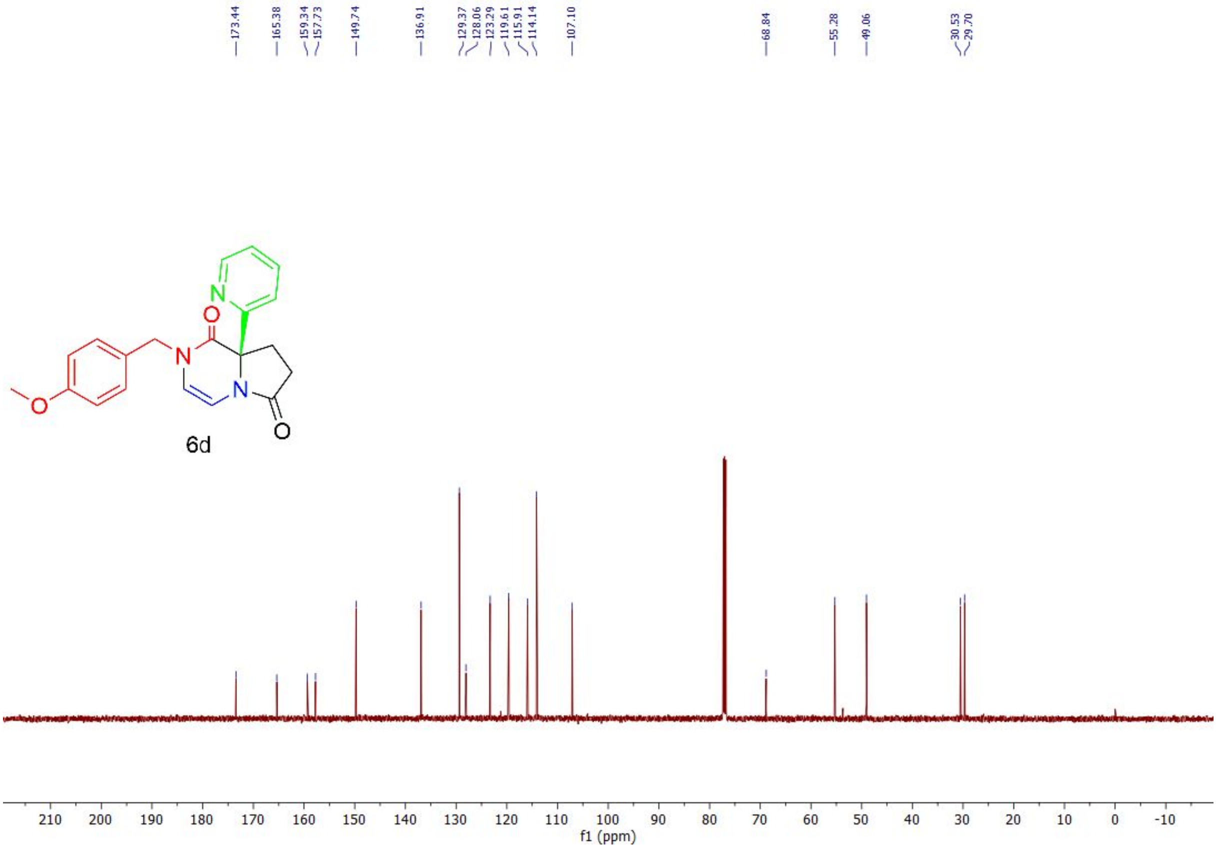

^{1}H NMR spectrum of **6e** (500 MHz, $CDCl_3$)

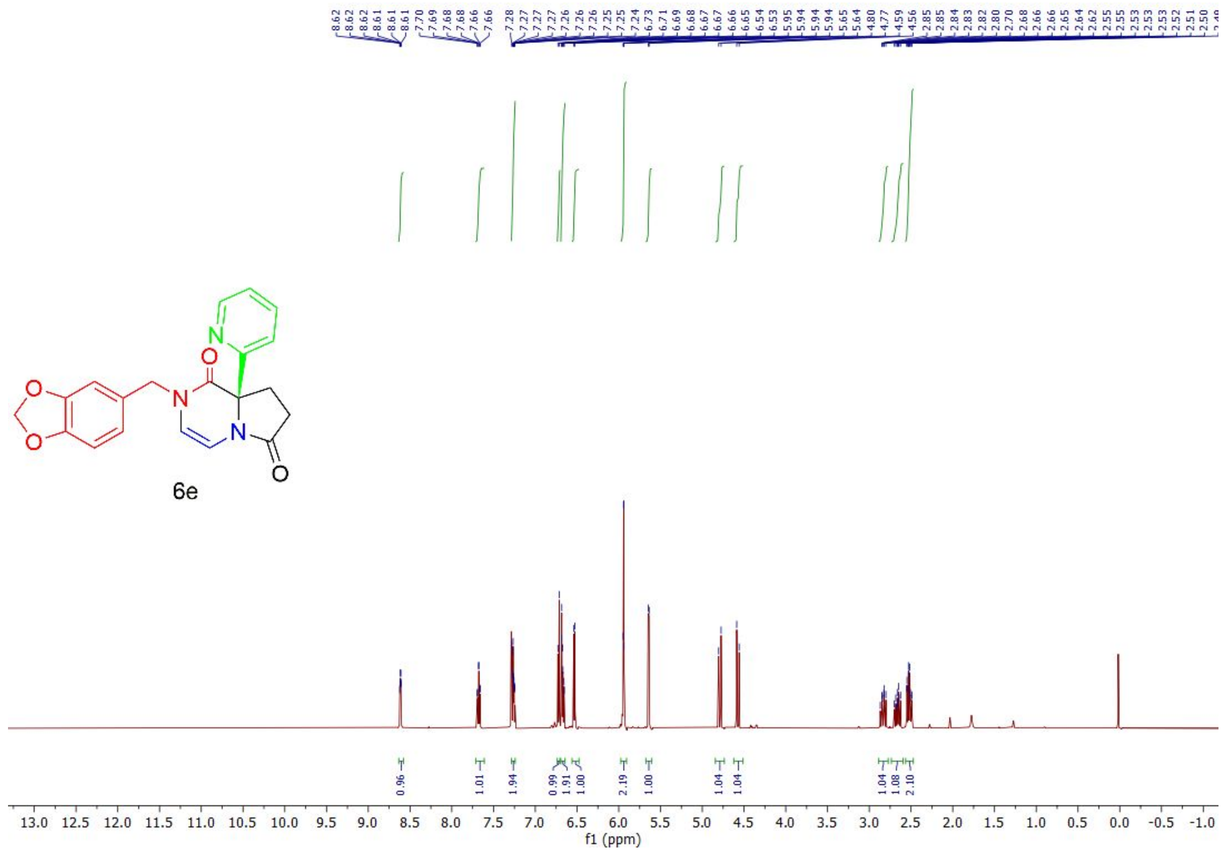

$^{13}C\{^{1}H\}$ NMR spectrum of **6e** (126 MHz, $CDCl_3$)

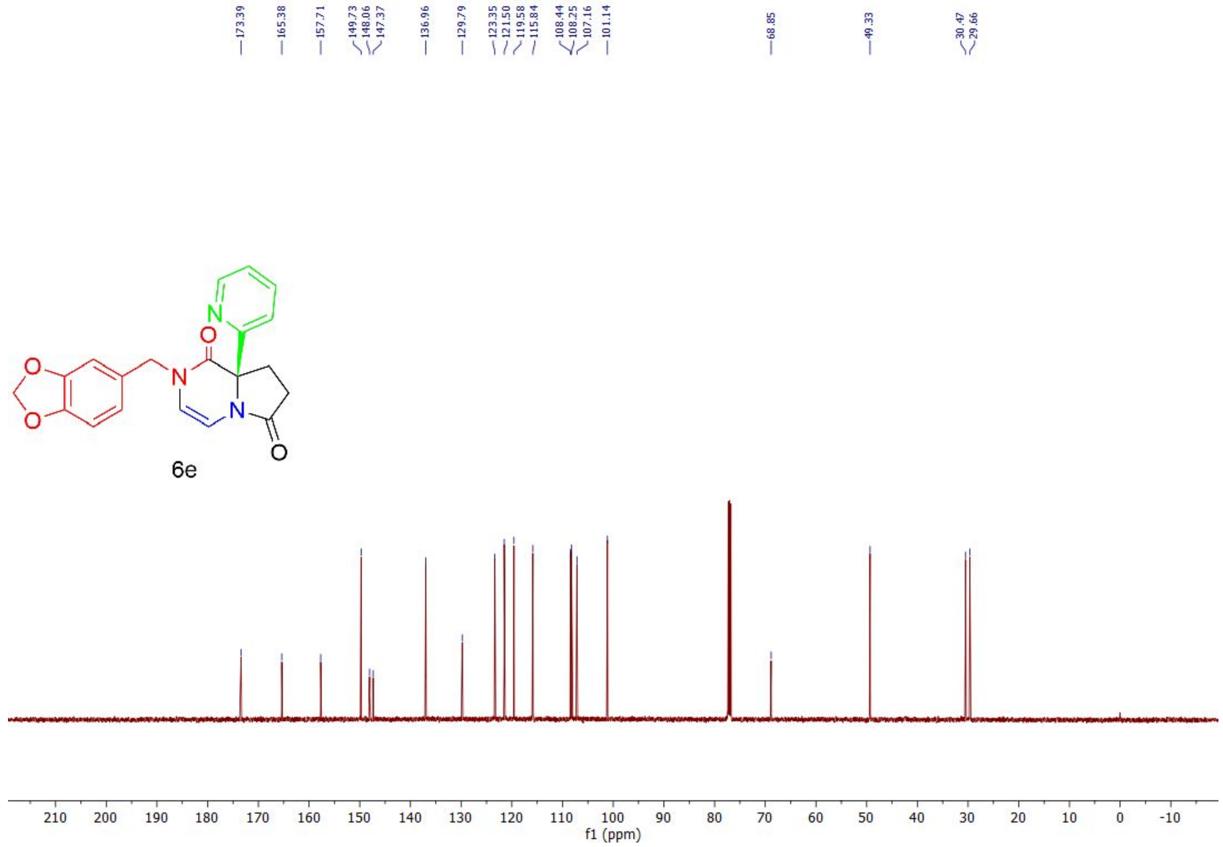

^{1}H NMR spectrum of **6f** (500 MHz, $CDCl_3$)

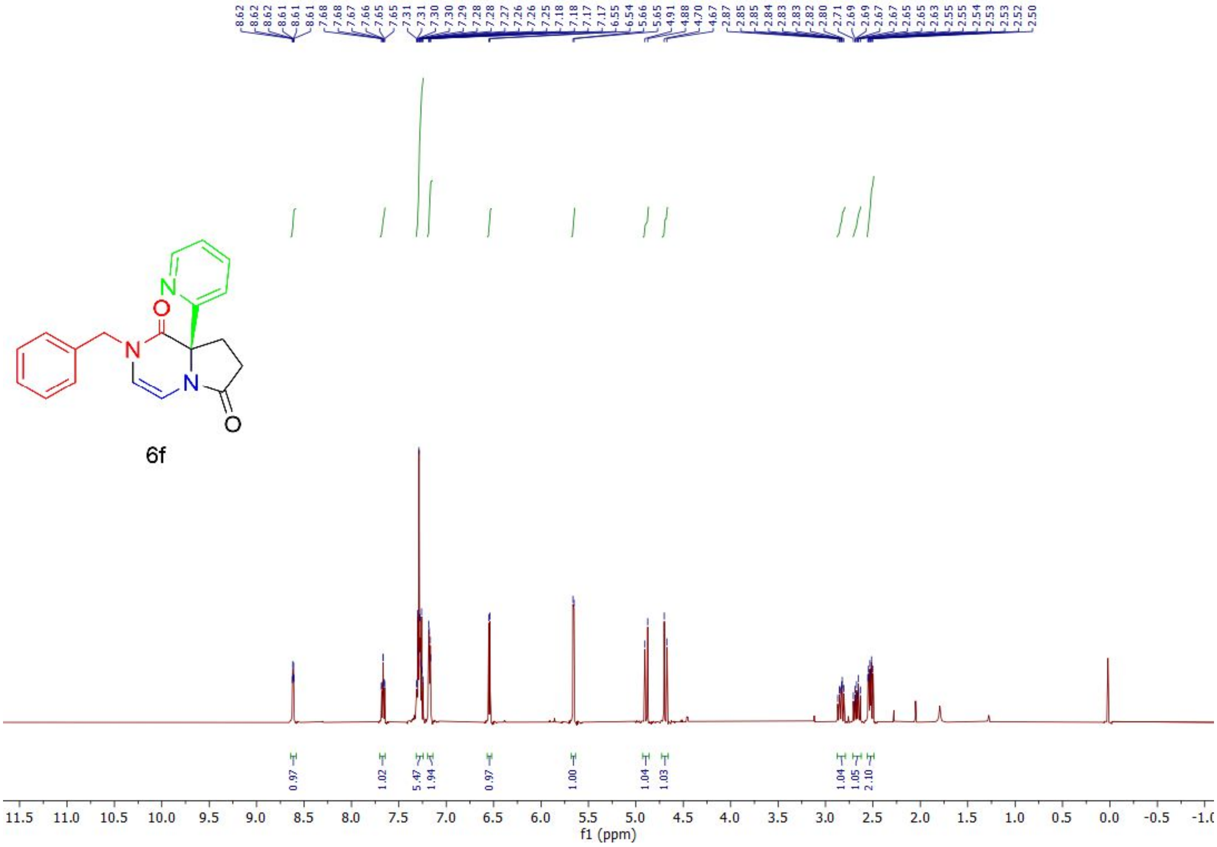

^{13}C{^{1}H} NMR spectrum of **6f** (126 MHz, $CDCl_3$)

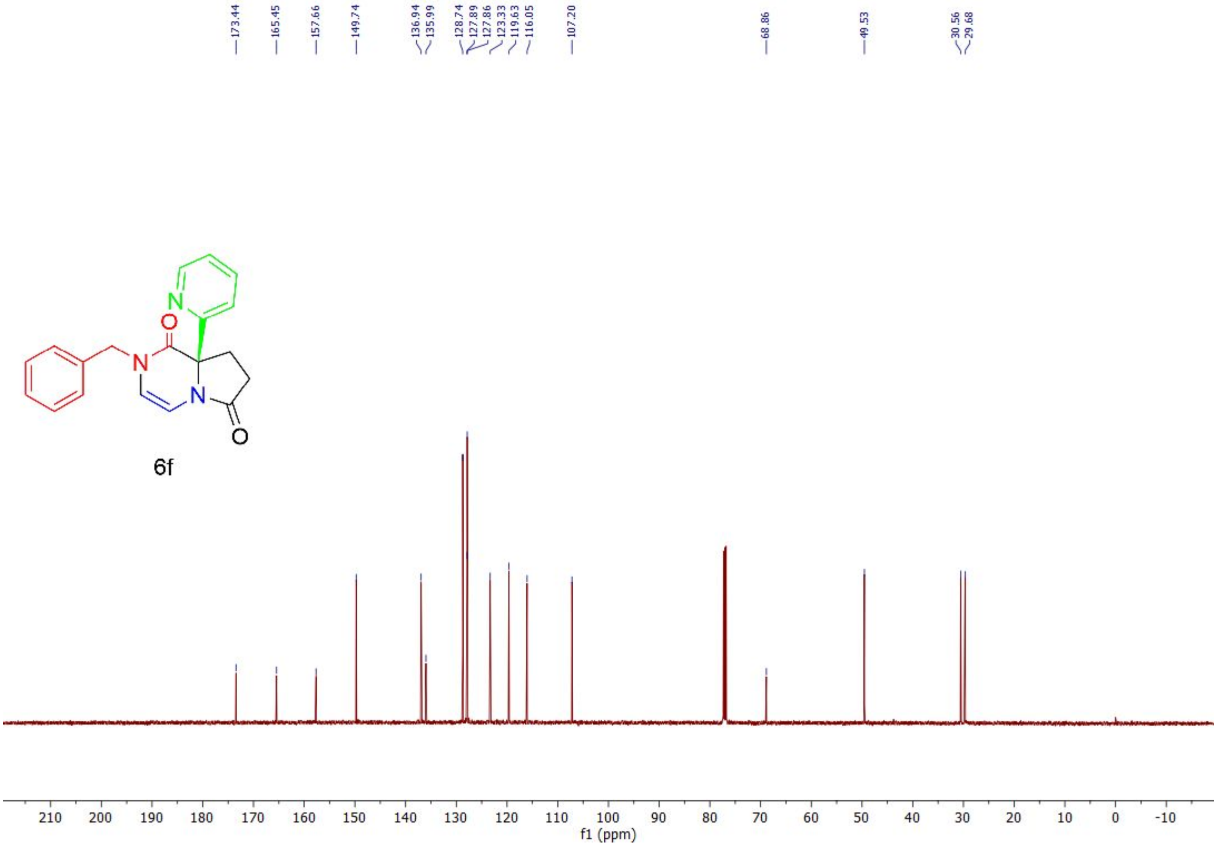

^{1}H NMR spectrum of **6g** (500 MHz, $CDCl_3$)

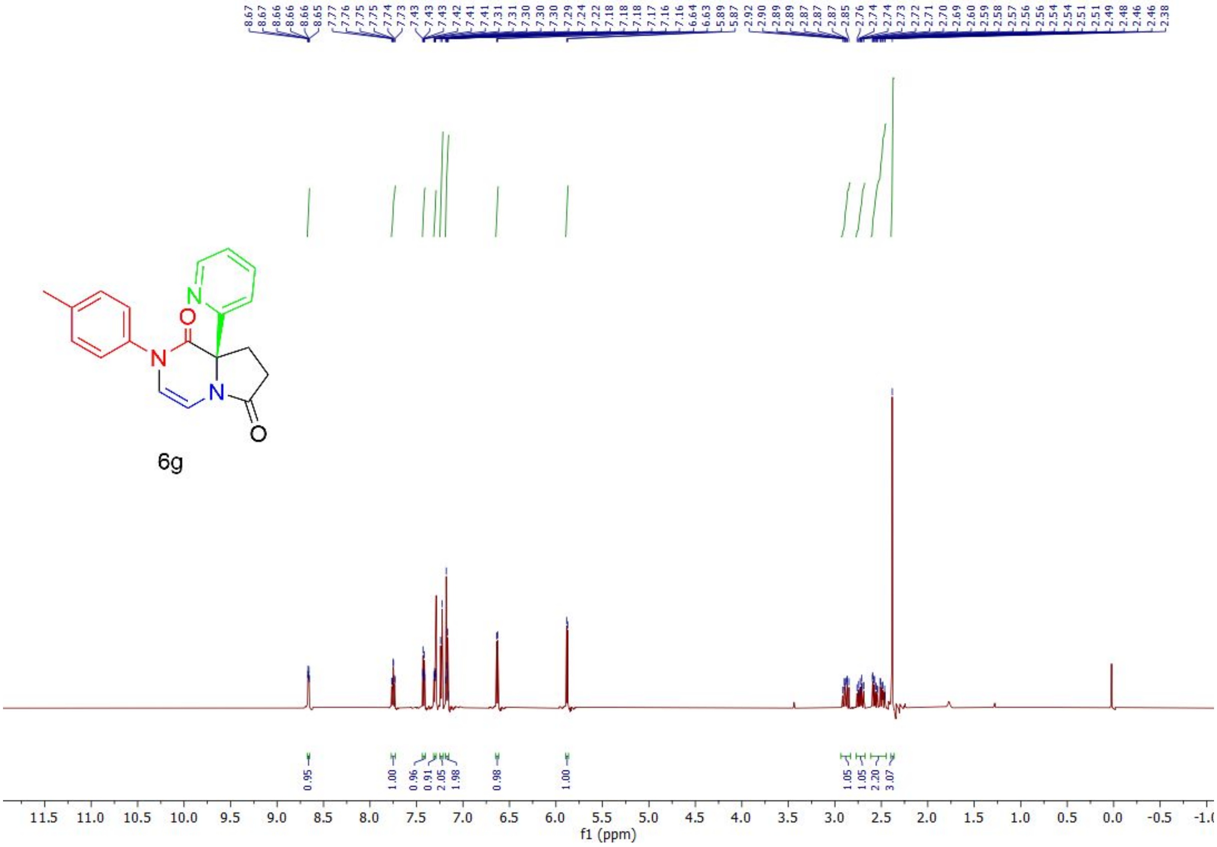

$^{13}C\{^{1}H\}$ NMR spectrum of **6g** (126 MHz, $CDCl_3$)

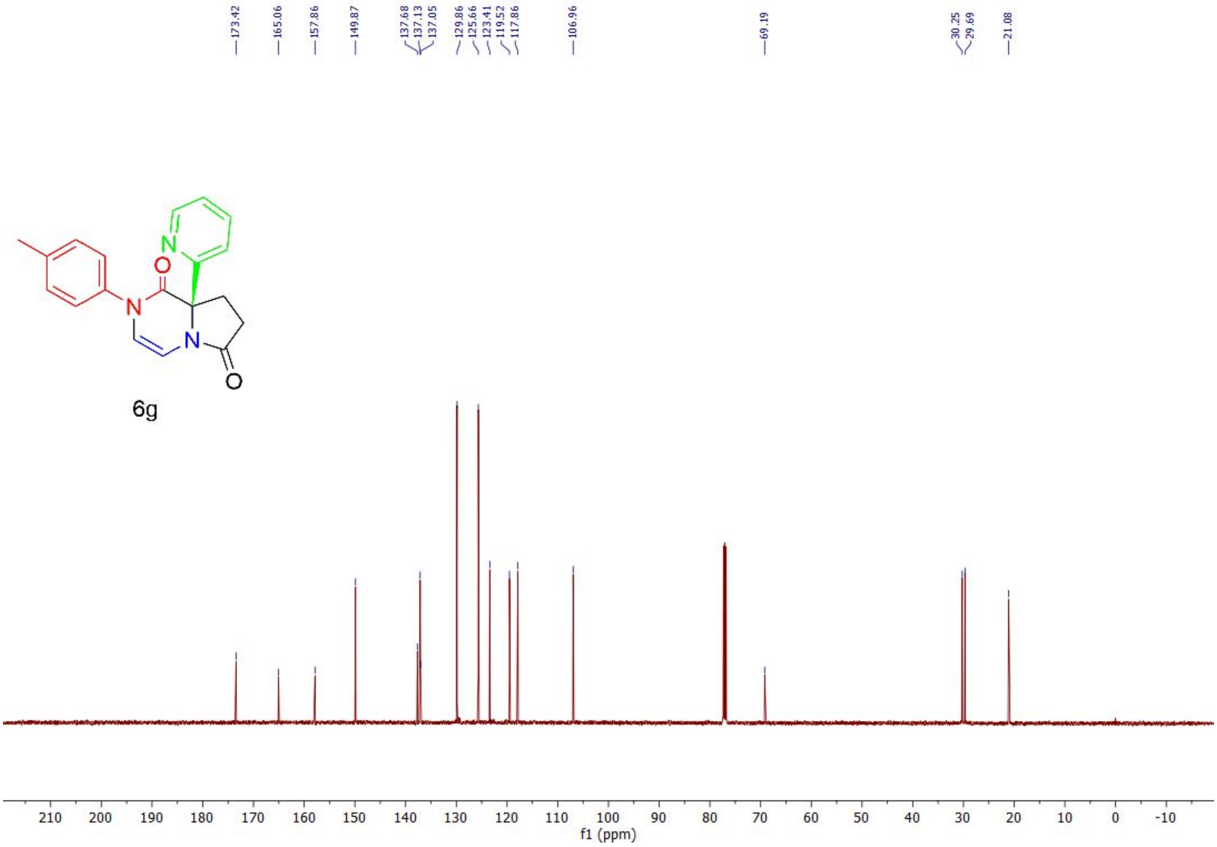

^{1}H NMR spectrum of **6h** (500 MHz, $CDCl_3$)

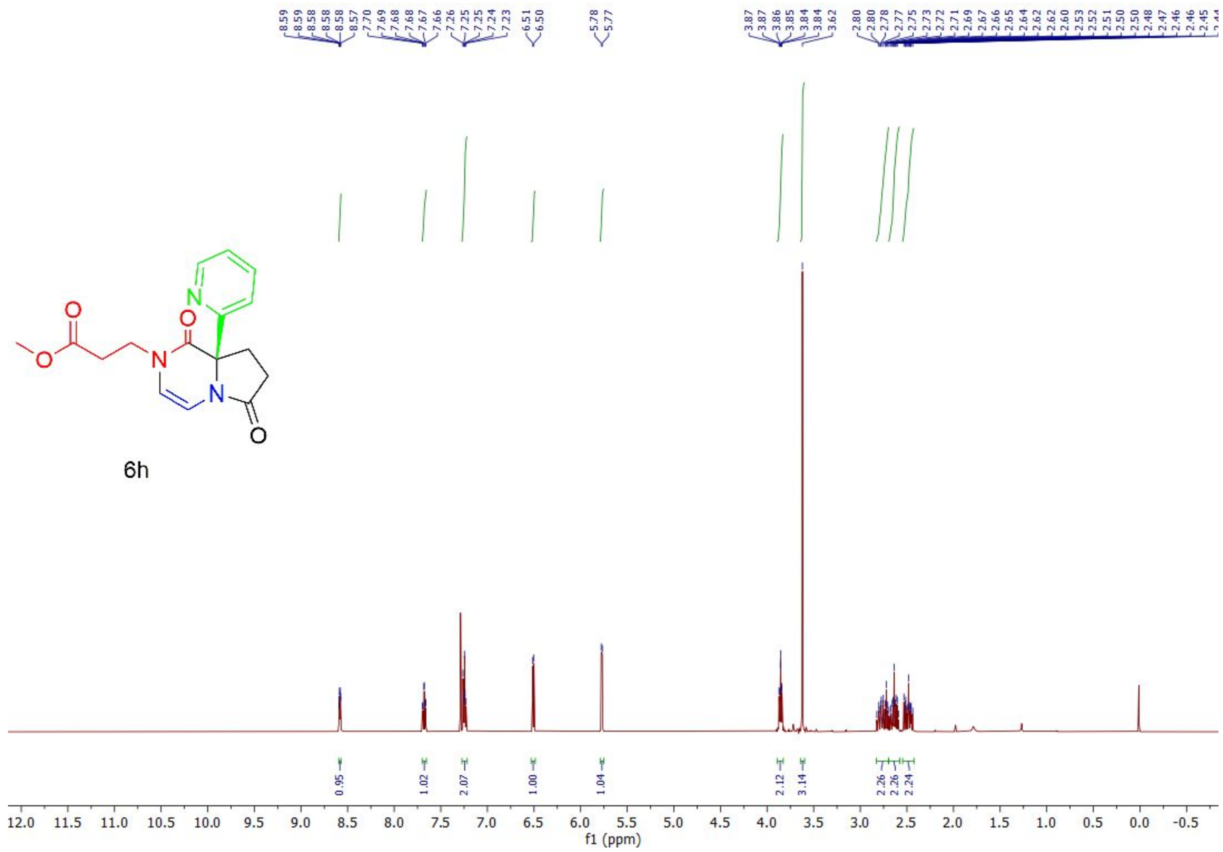

$^{13}C\{^{1}H\}$ NMR spectrum of **6h** (126 MHz, $CDCl_3$)

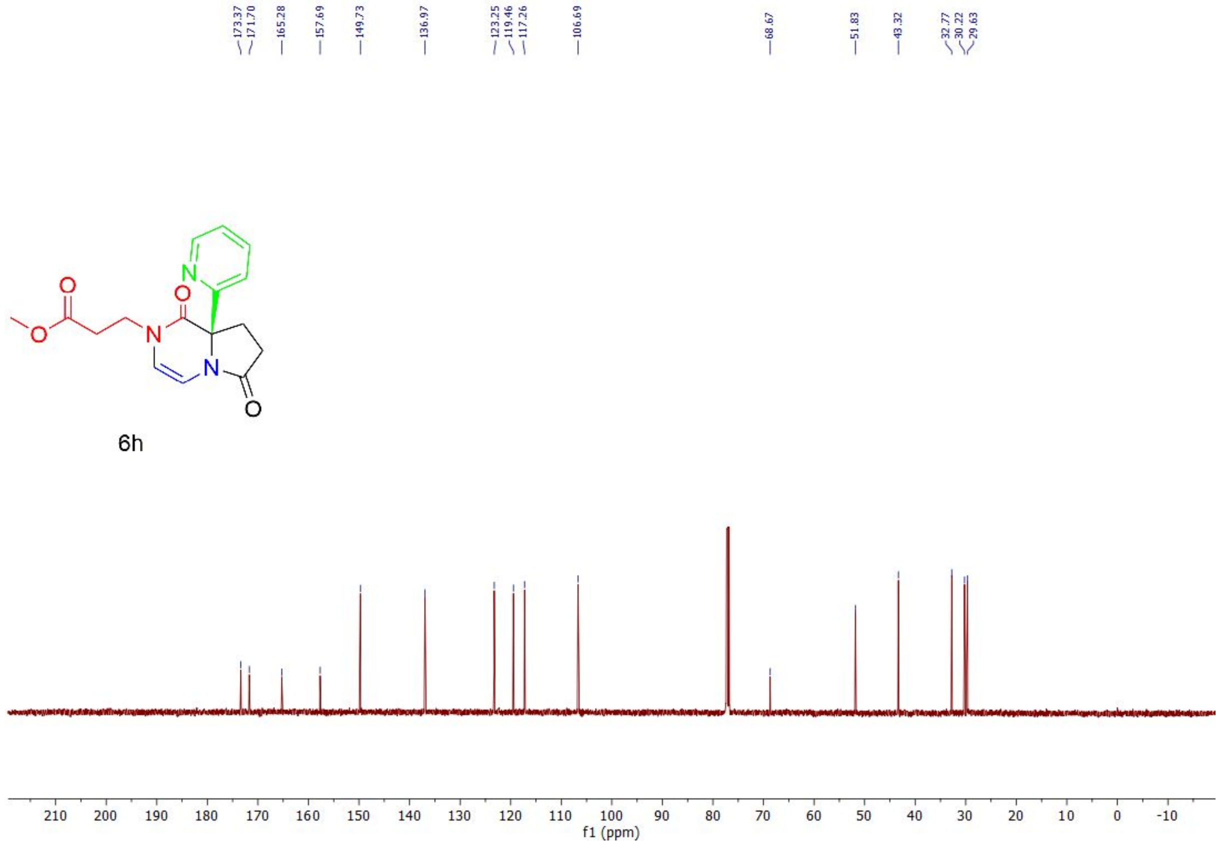

^{1}H NMR spectrum of **6i** (500 MHz, $CDCl_3$)

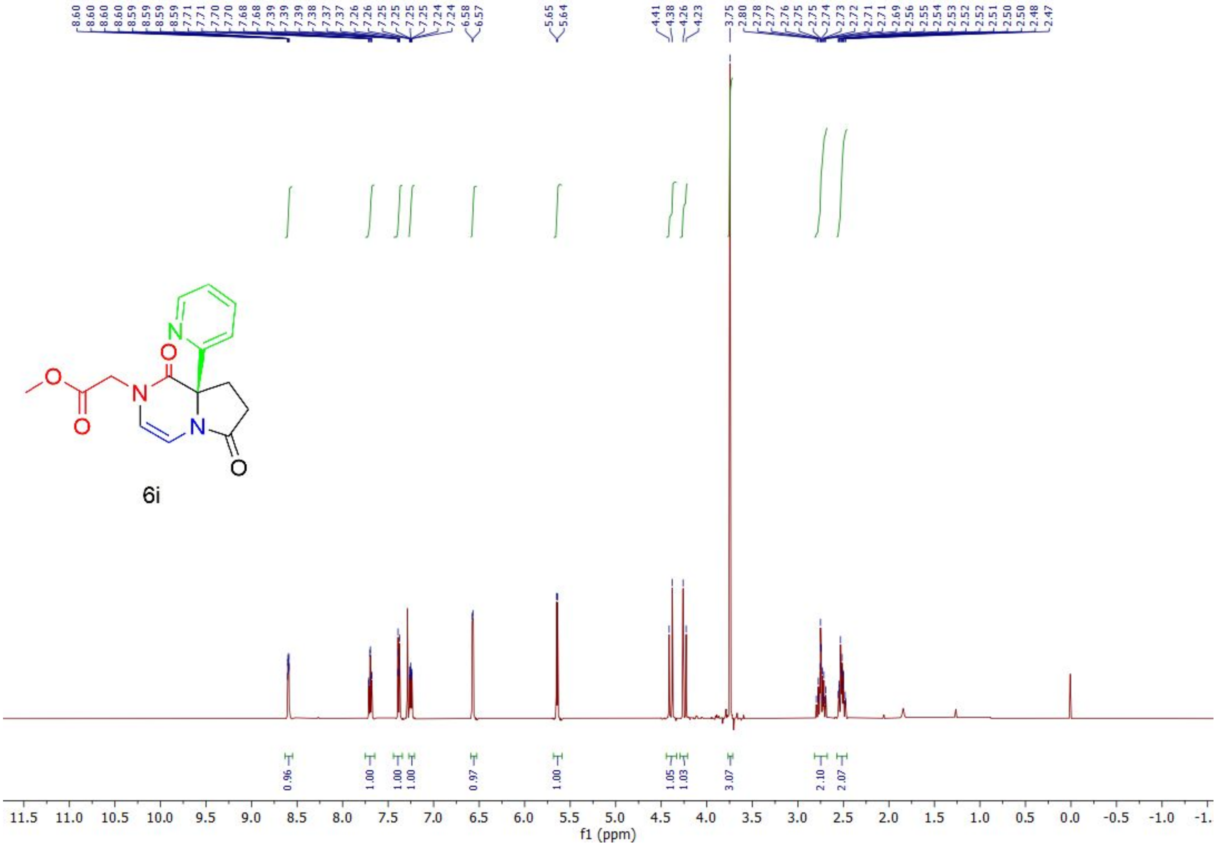

$^{13}C\{^1H\}$ NMR spectrum of **6i** (126 MHz, $CDCl_3$)

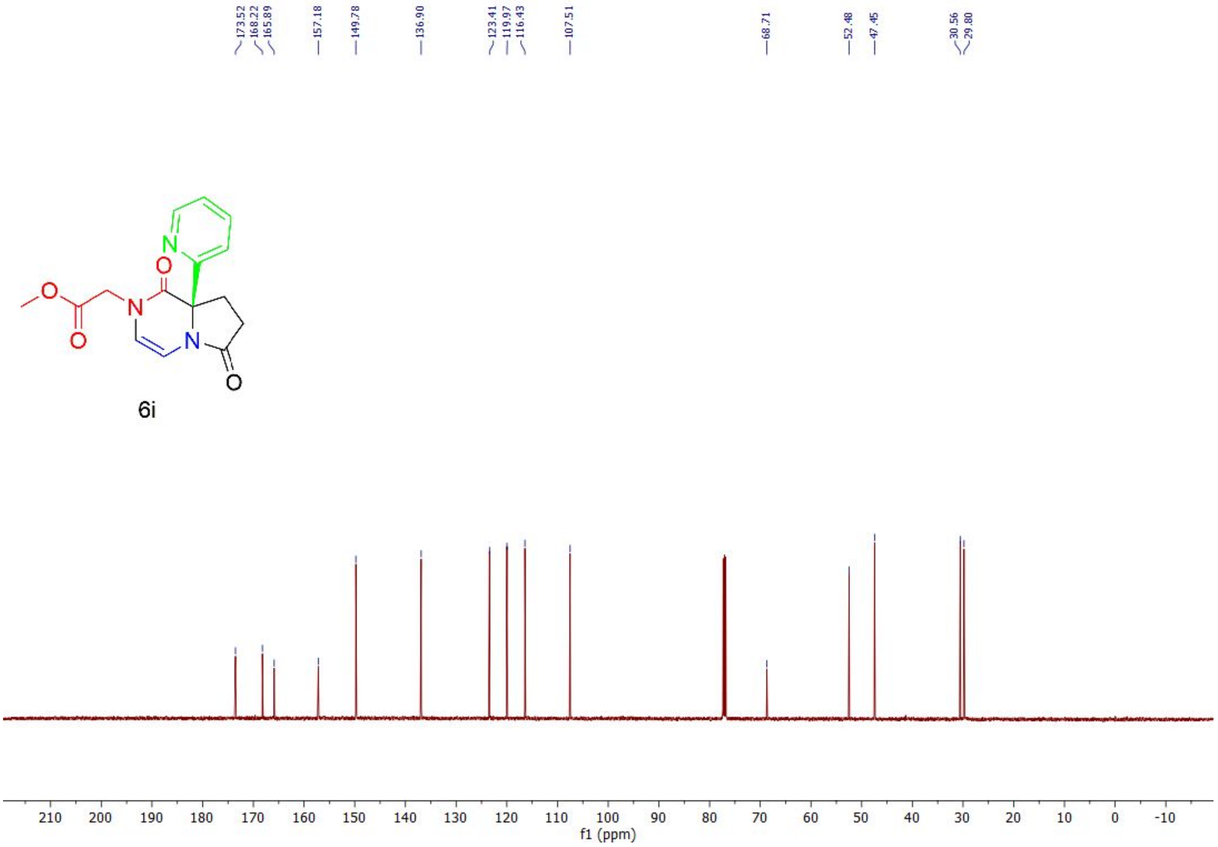

^{1}H NMR spectrum of **6j** (500 MHz, $CDCl_3$)

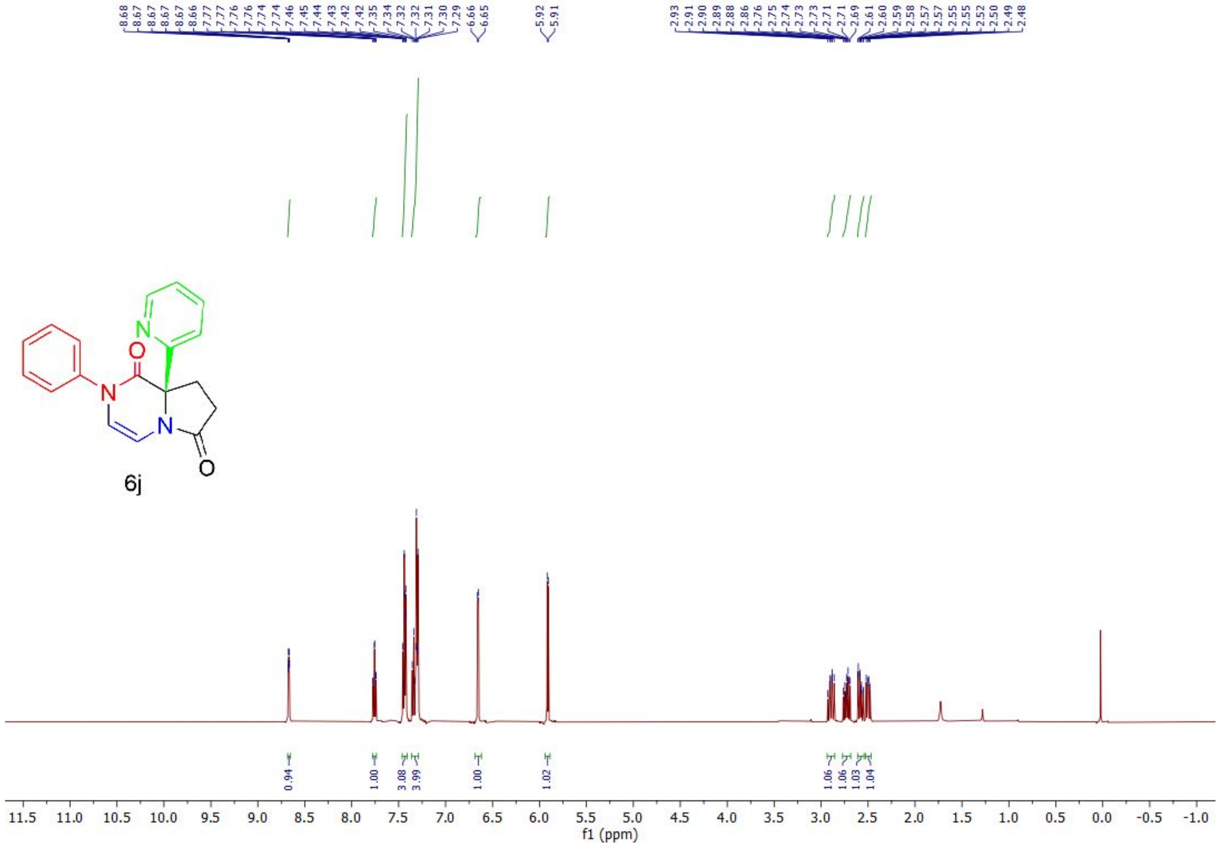

$^{13}C\{^{1}H\}$ NMR spectrum of **6j** (126 MHz, $CDCl_3$)

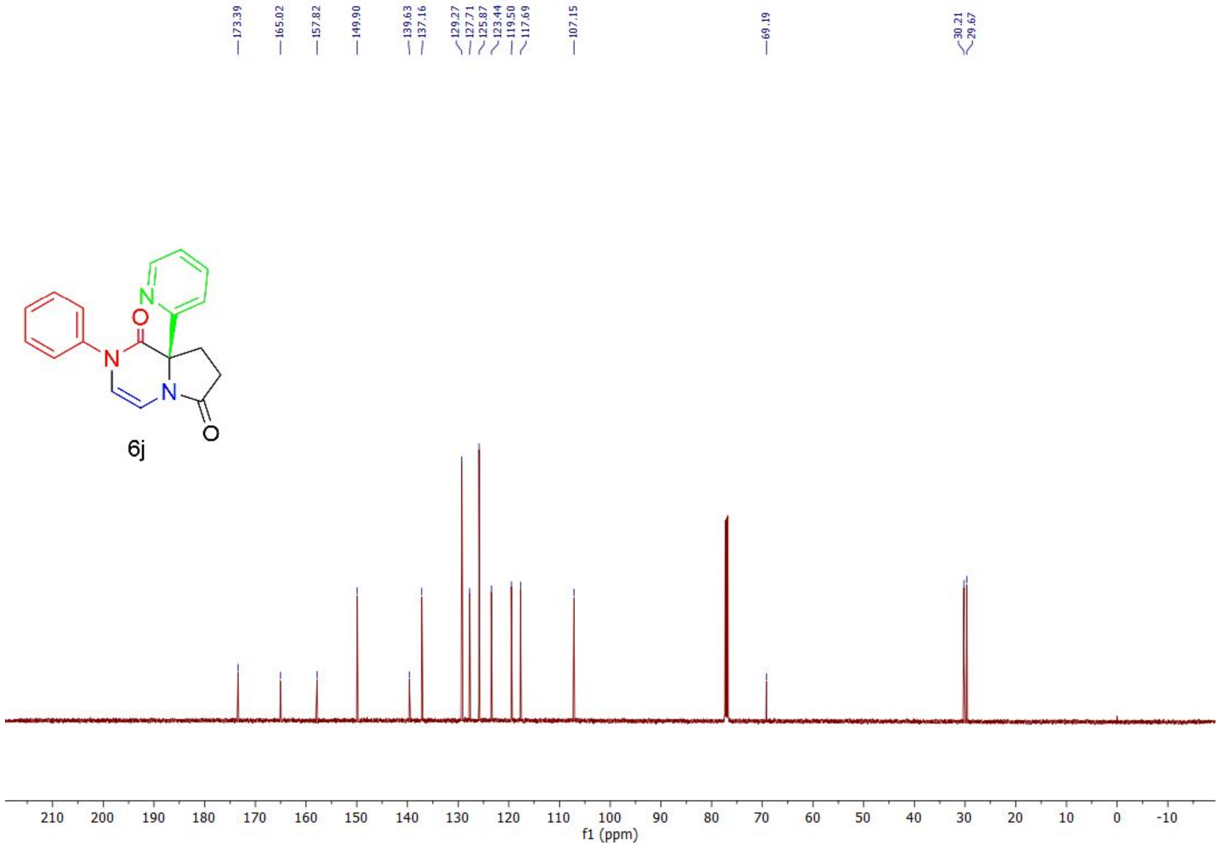

^{1}H NMR spectrum of **6k** (500 MHz, $CDCl_3$)

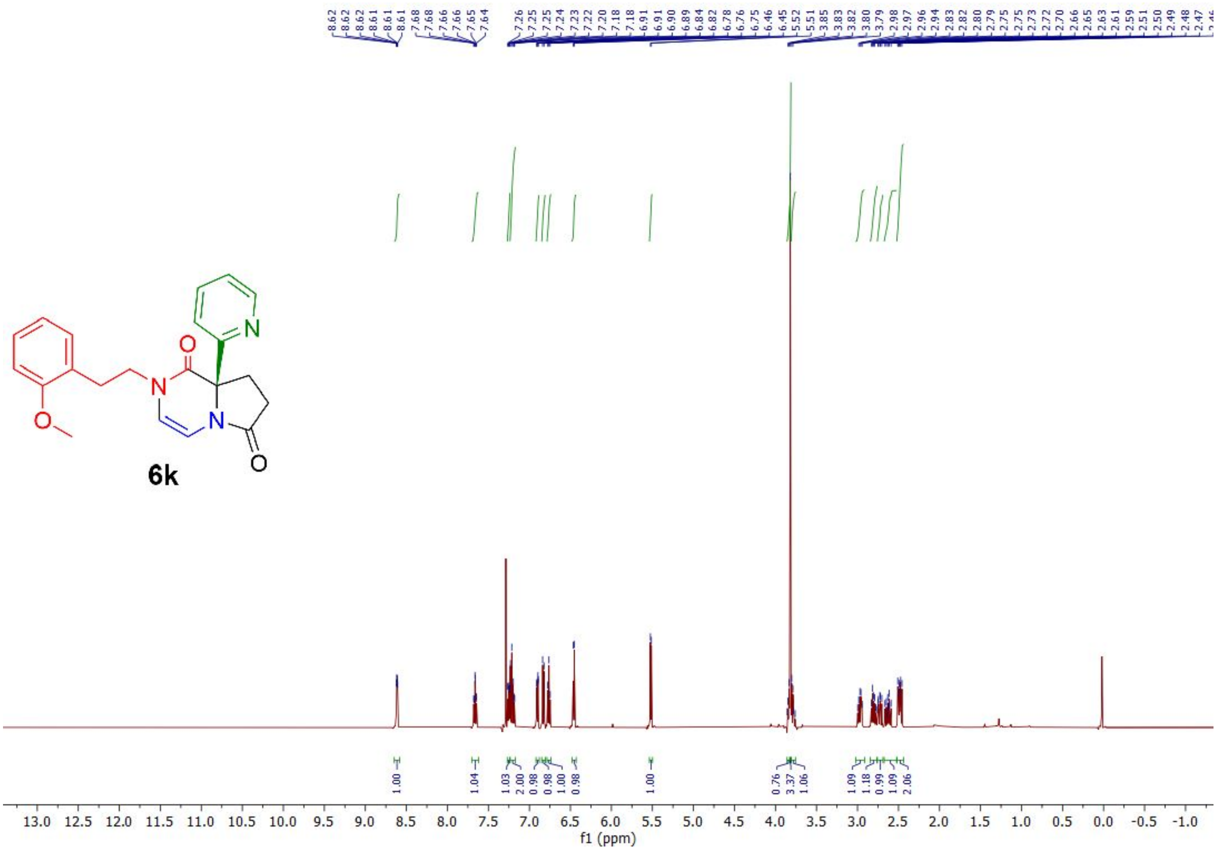

^{13}C{^{1}H} NMR spectrum of **6k** (126 MHz, $CDCl_3$)

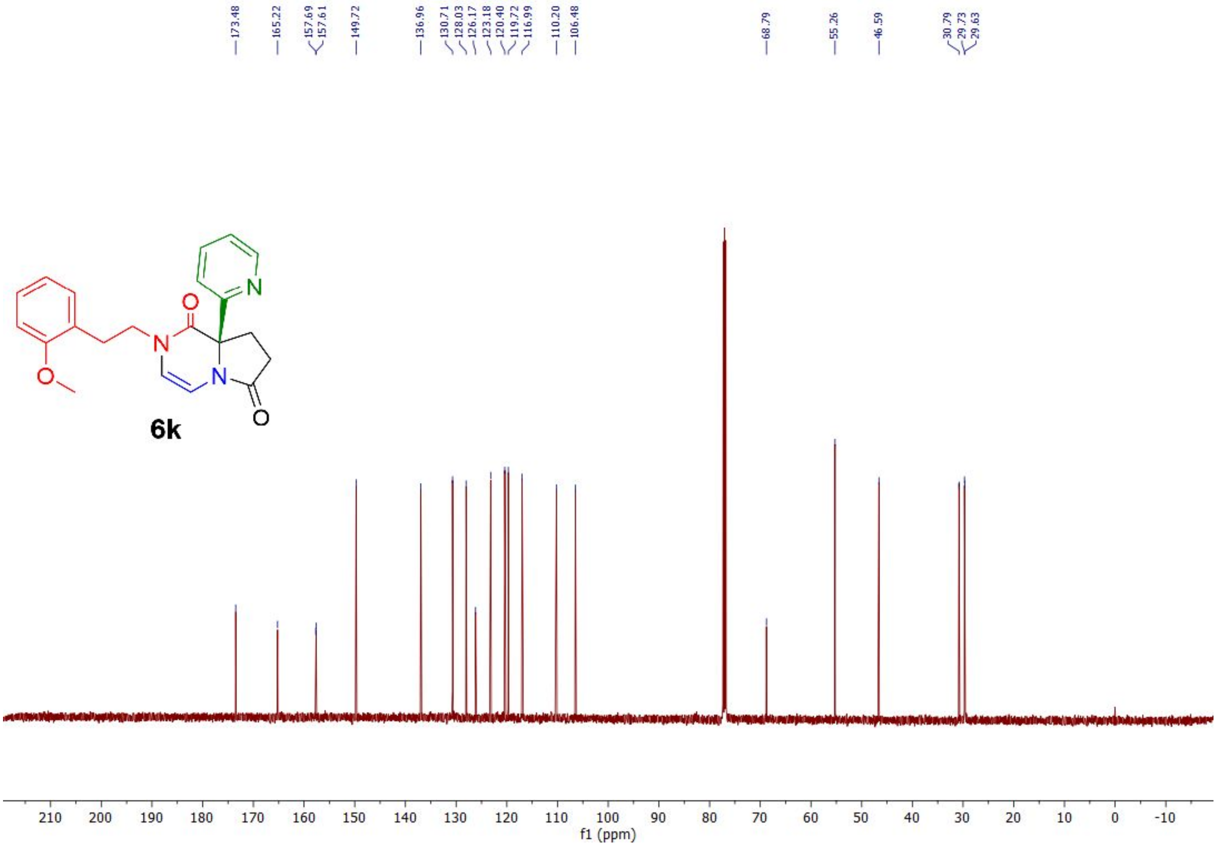

^{1}NMR spectrum of **6l** (500 MHz, $CDCl_3$)

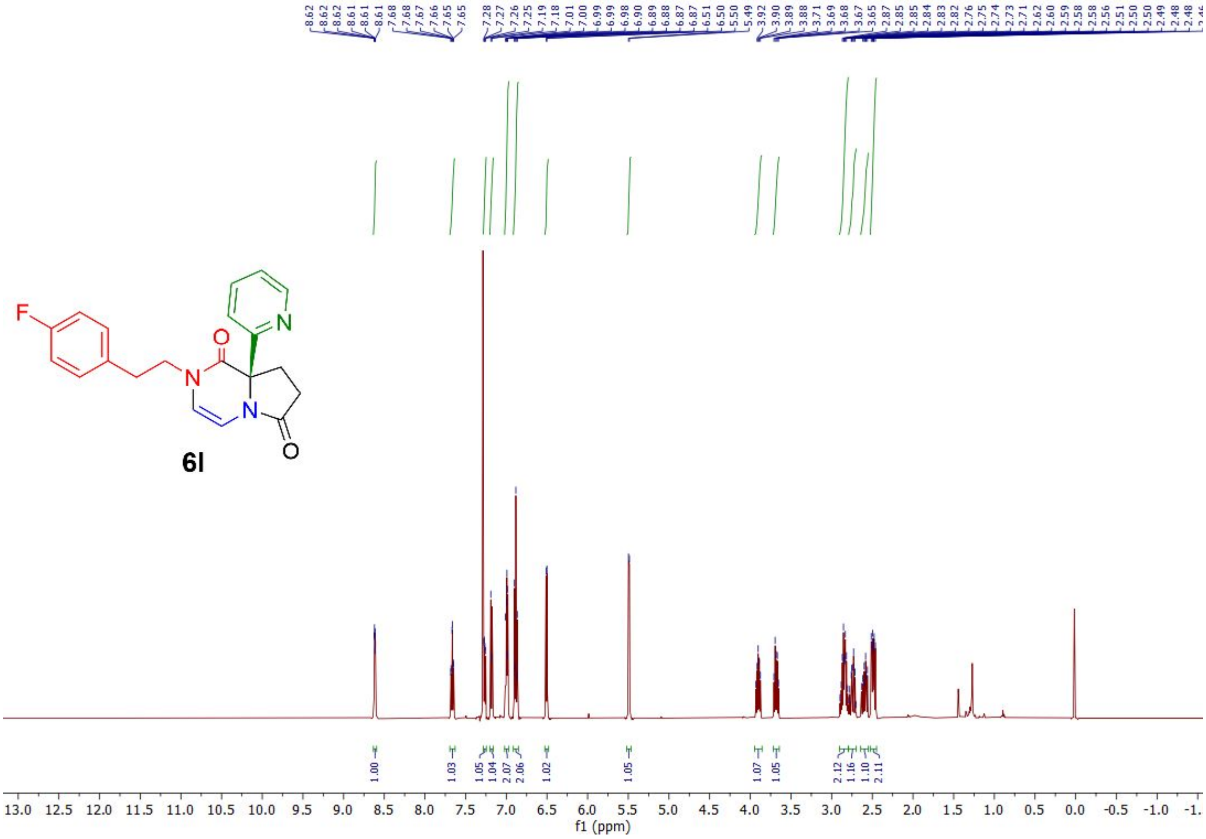

$^{13}C\{^{1}H\}$ NMR spectrum of **6l** (126 MHz, $CDCl_3$)

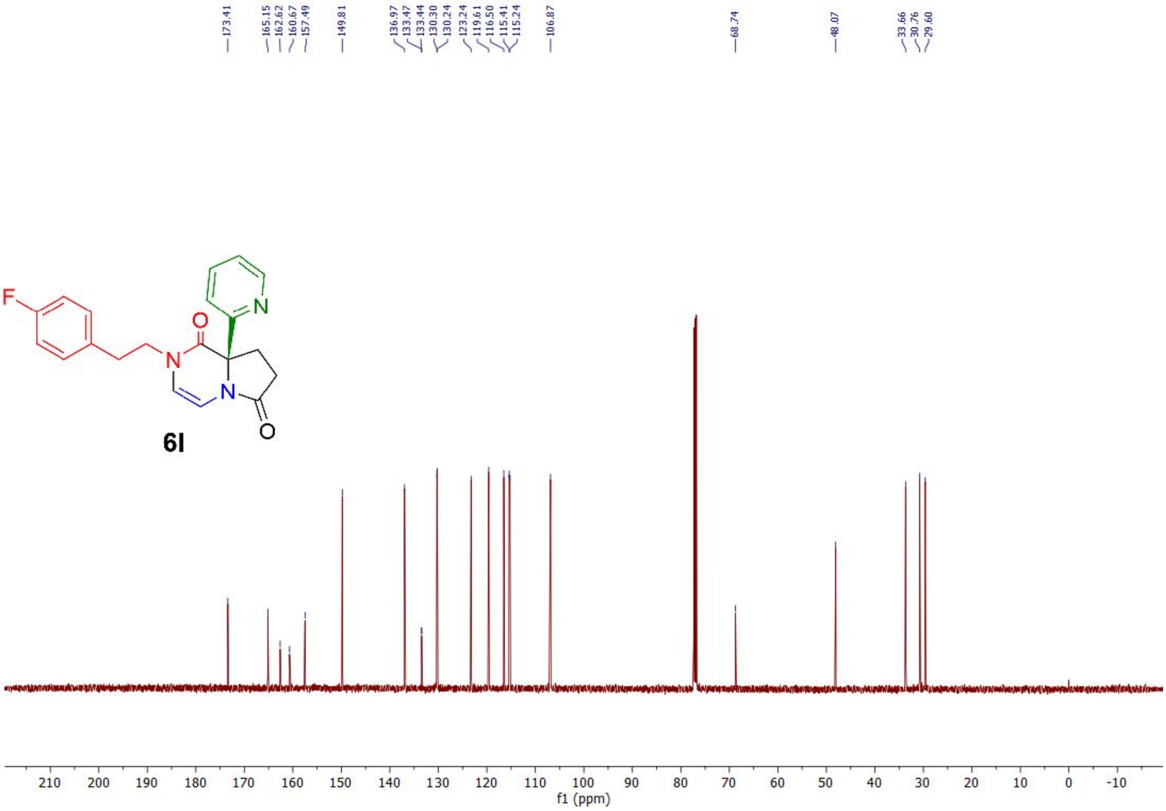

[1]NMR spectrum of **6m** (500 MHz, $CDCl_3$)

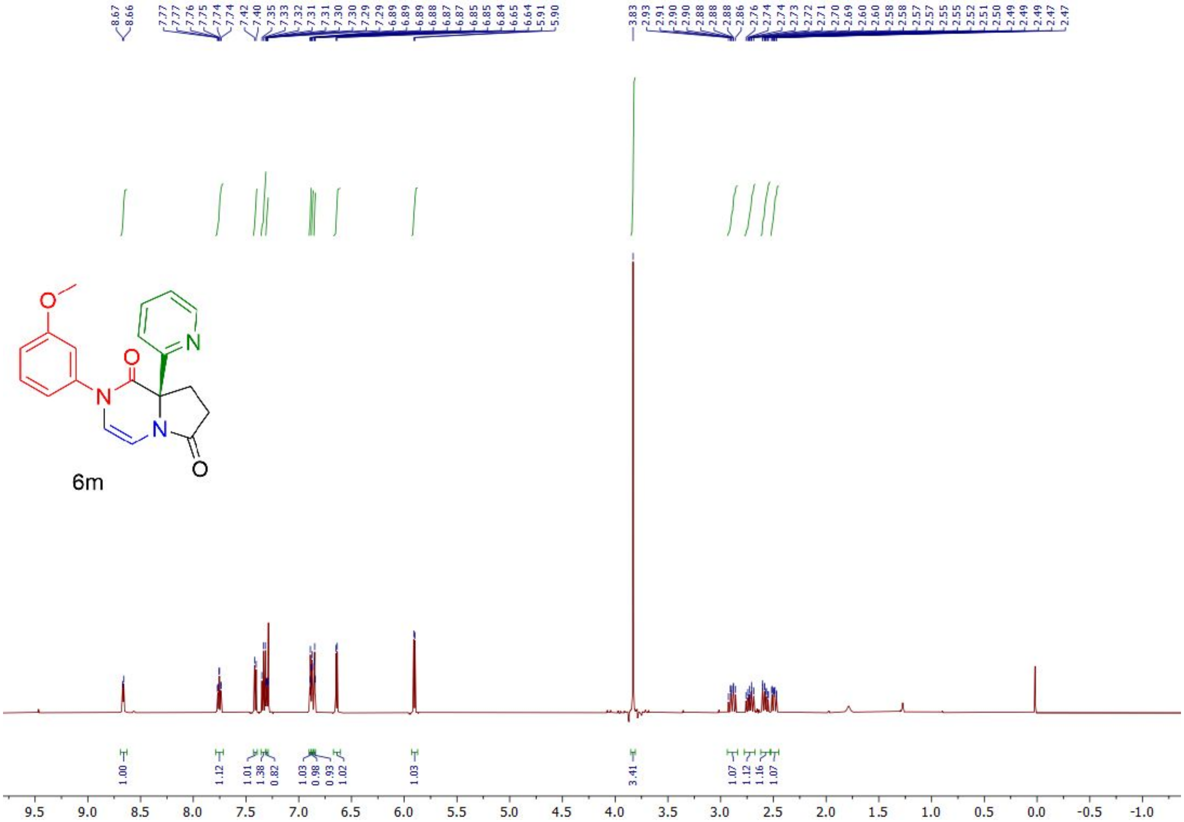

$^{13}C\{^1H\}$ NMR spectrum of **6m** (126 MHz, $CDCl_3$)

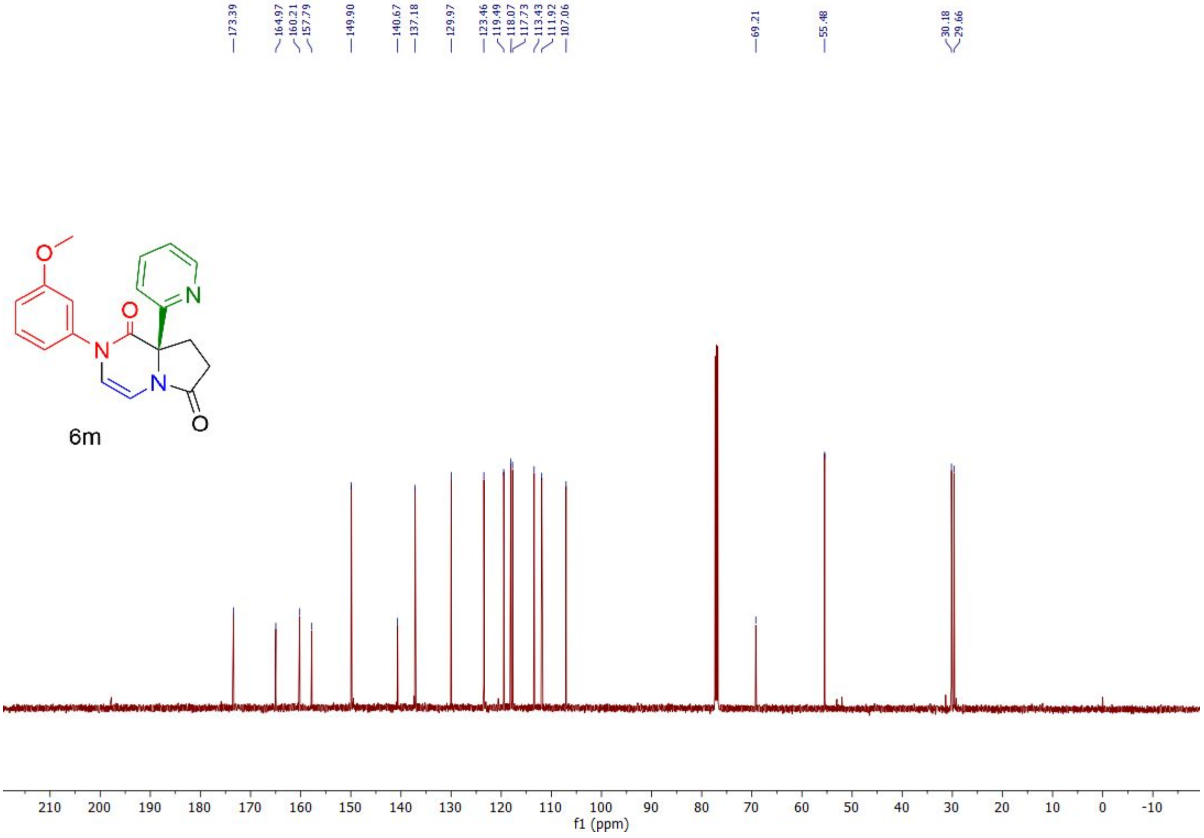

[1]NMR spectrum of **6n** (500 MHz, $CDCl_3$)

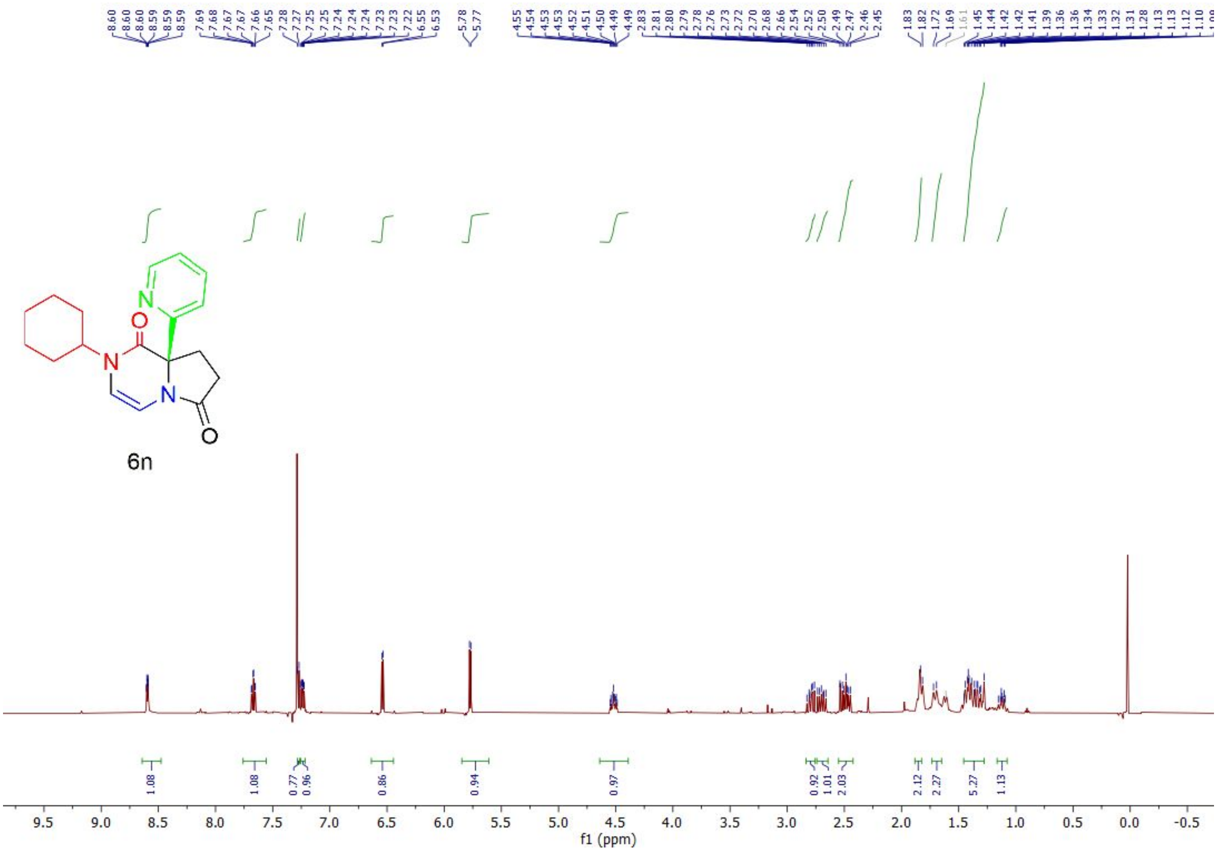

$^{13}C\{^1H\}$ NMR spectrum of **6n** (126 MHz, $CDCl_3$)

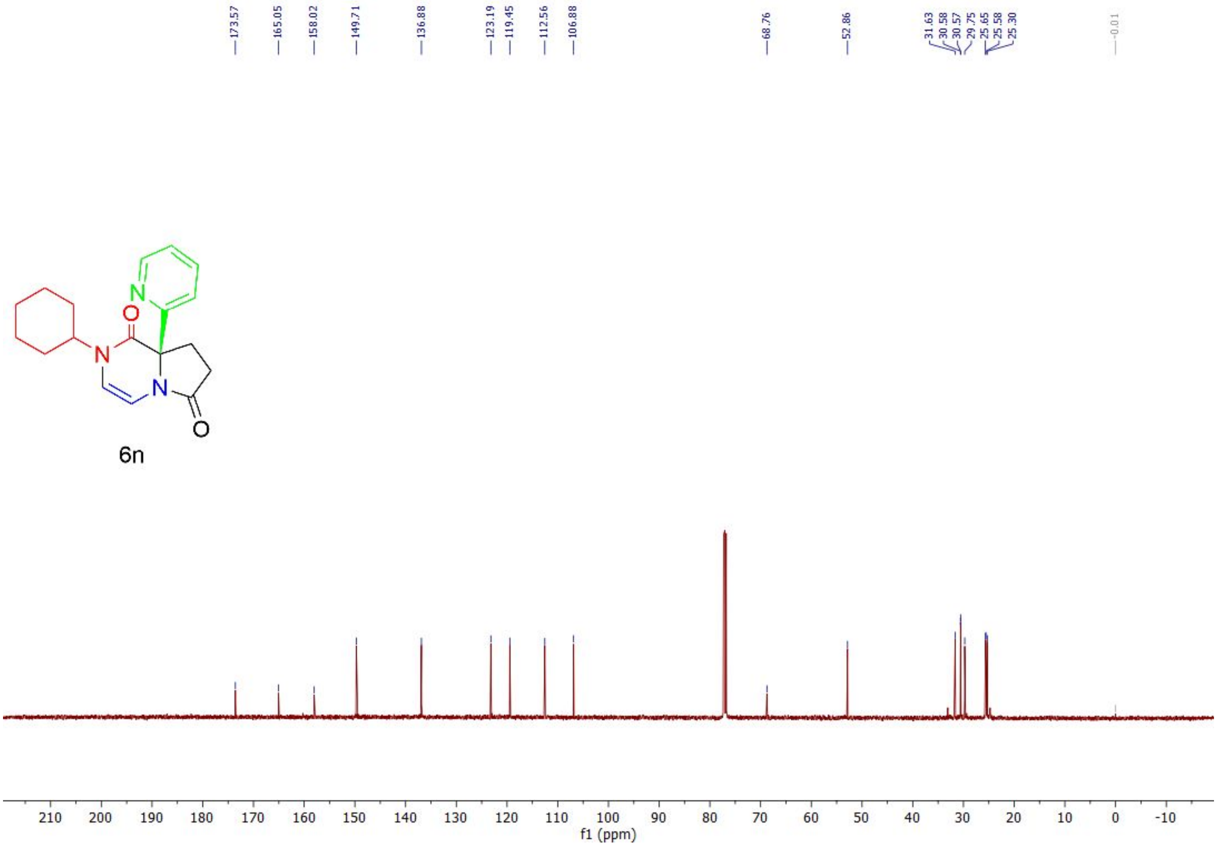

[1]NMR spectrum of **6o** (500 MHz, $CDCl_3$)

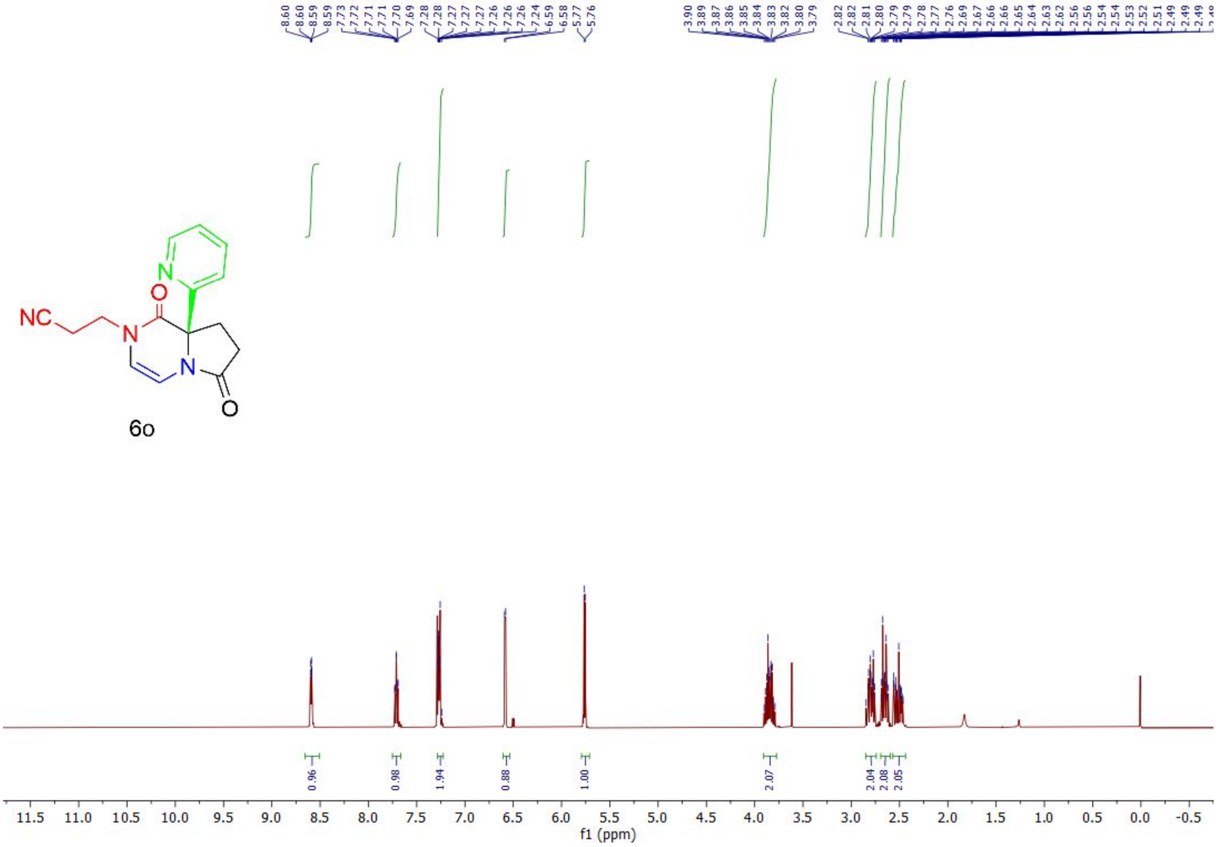

^{13}C{^{1}H} NMR spectrum of **6o** (126 MHz, $CDCl_3$)

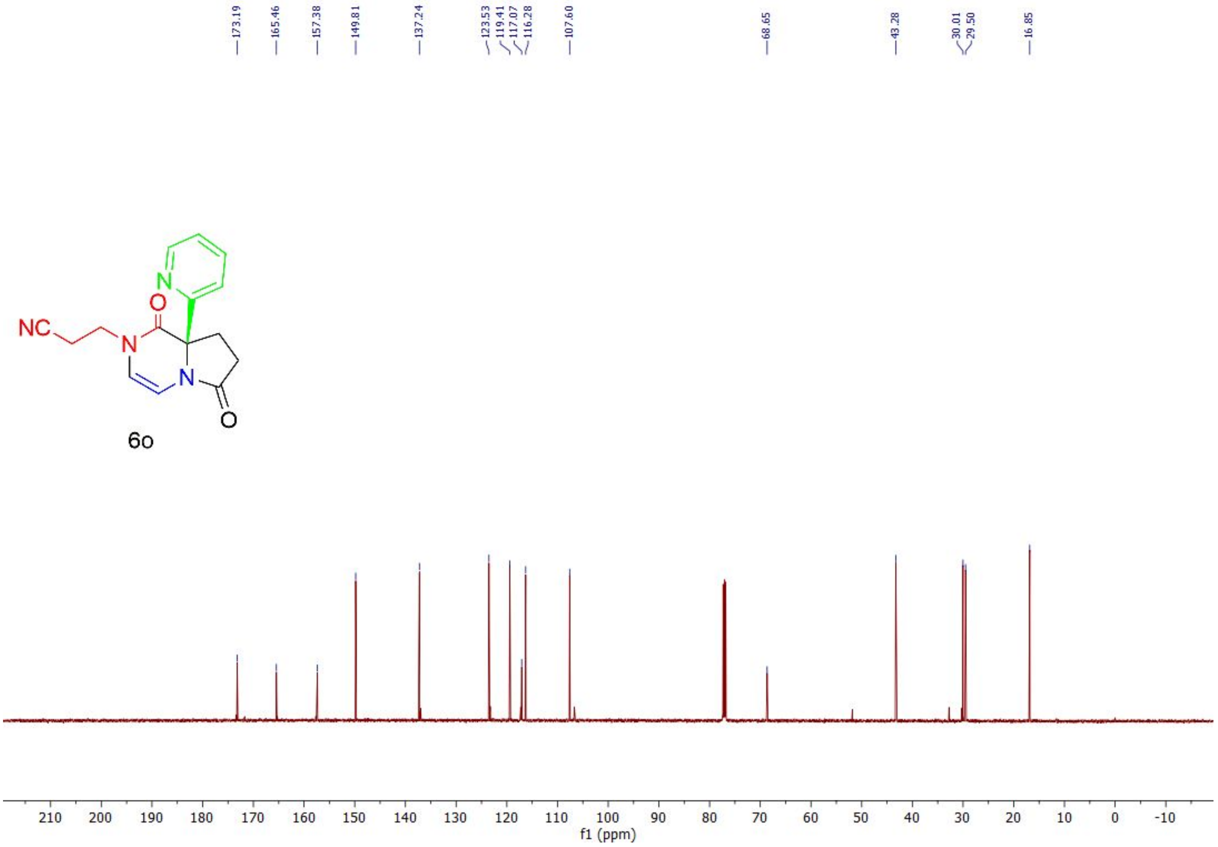

[1]NMR spectrum of **6p** (500 MHz, $CDCl_3$)

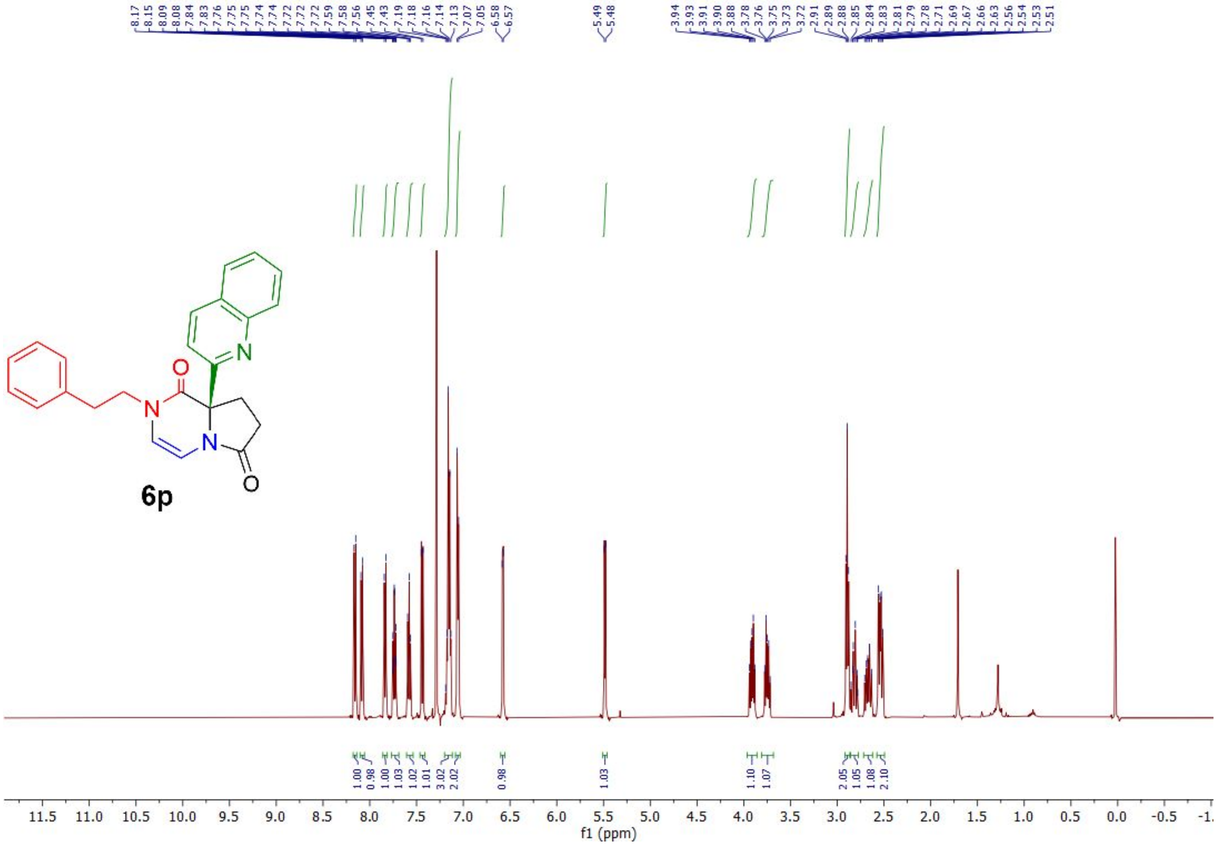

^{13}C{^{1}H} NMR spectrum of **6p** (126 MHz, $CDCl_3$)

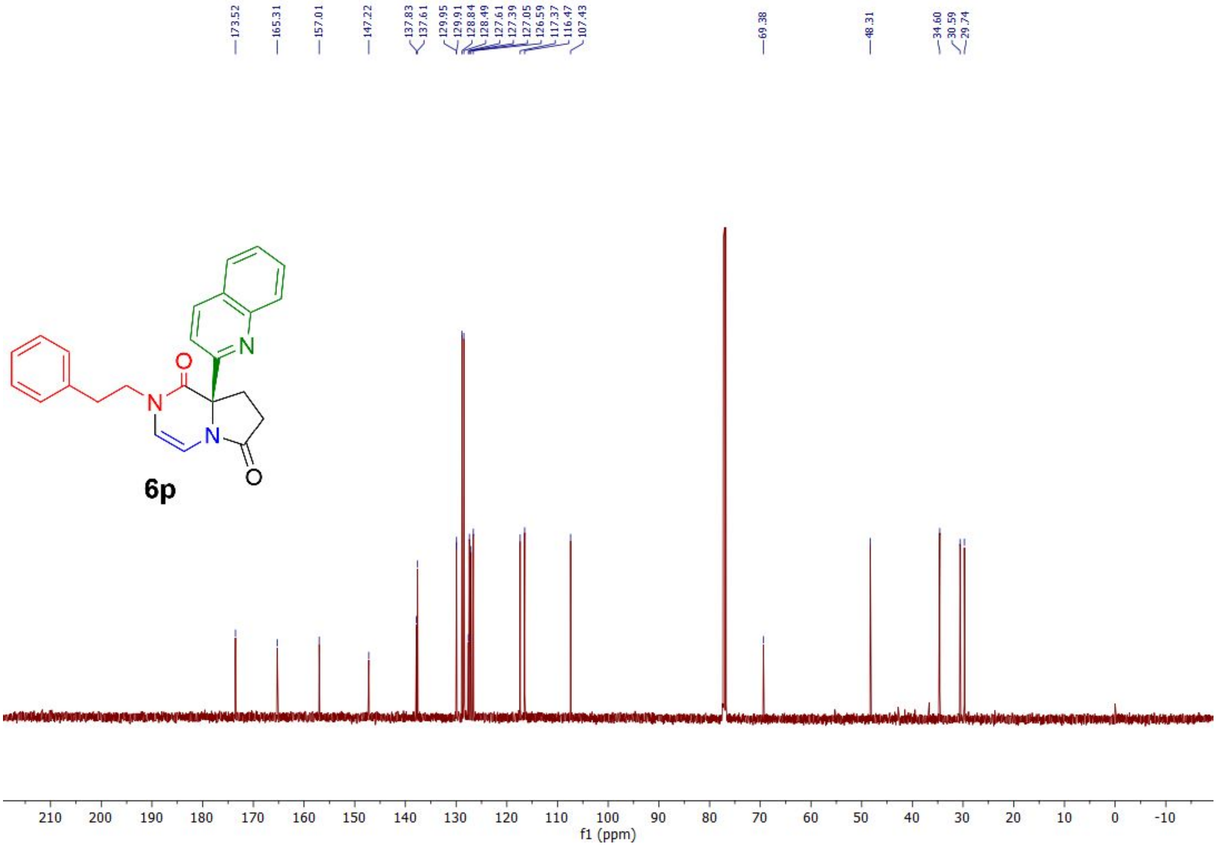

[1]NMR spectrum of **6q** (500 MHz, $CDCl_3$)

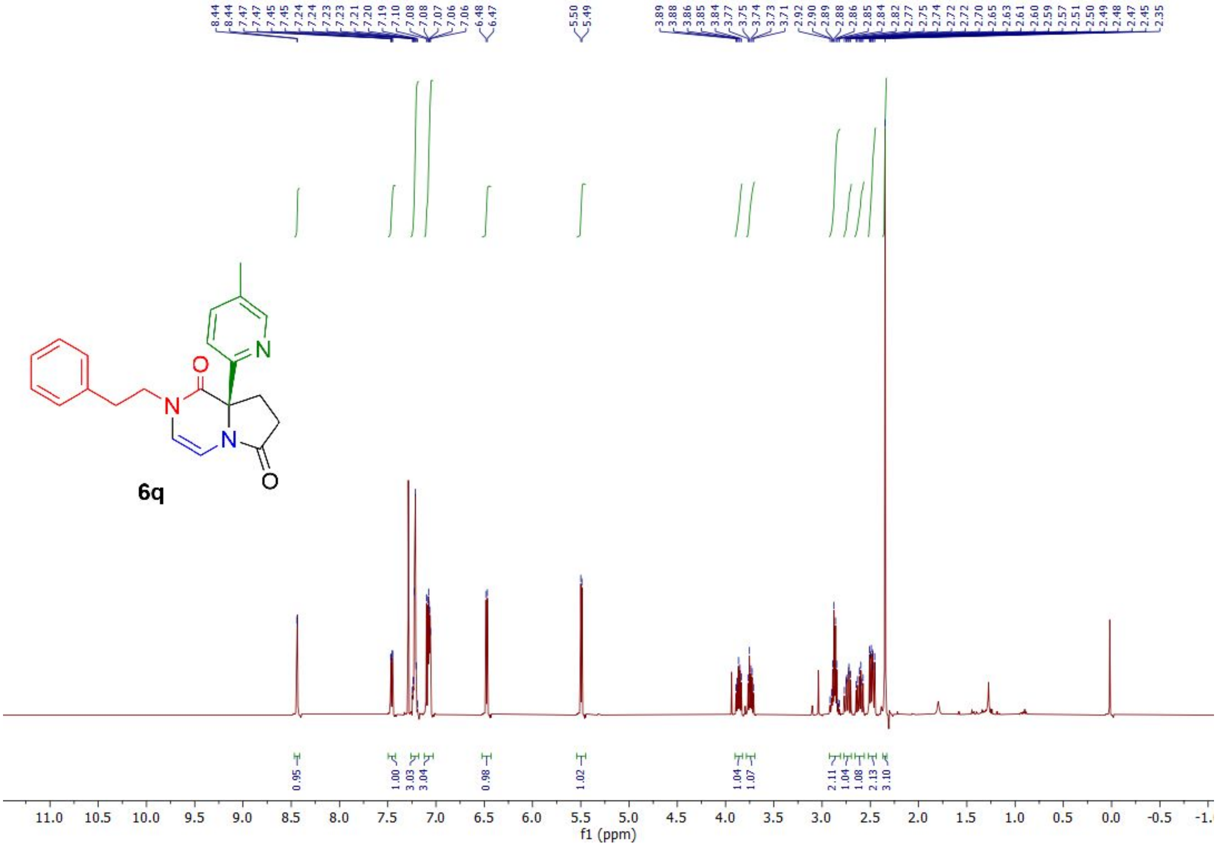

$^{13}C\{^{1}H\}$ NMR spectrum of **6q** (126 MHz, $CDCl_3$)

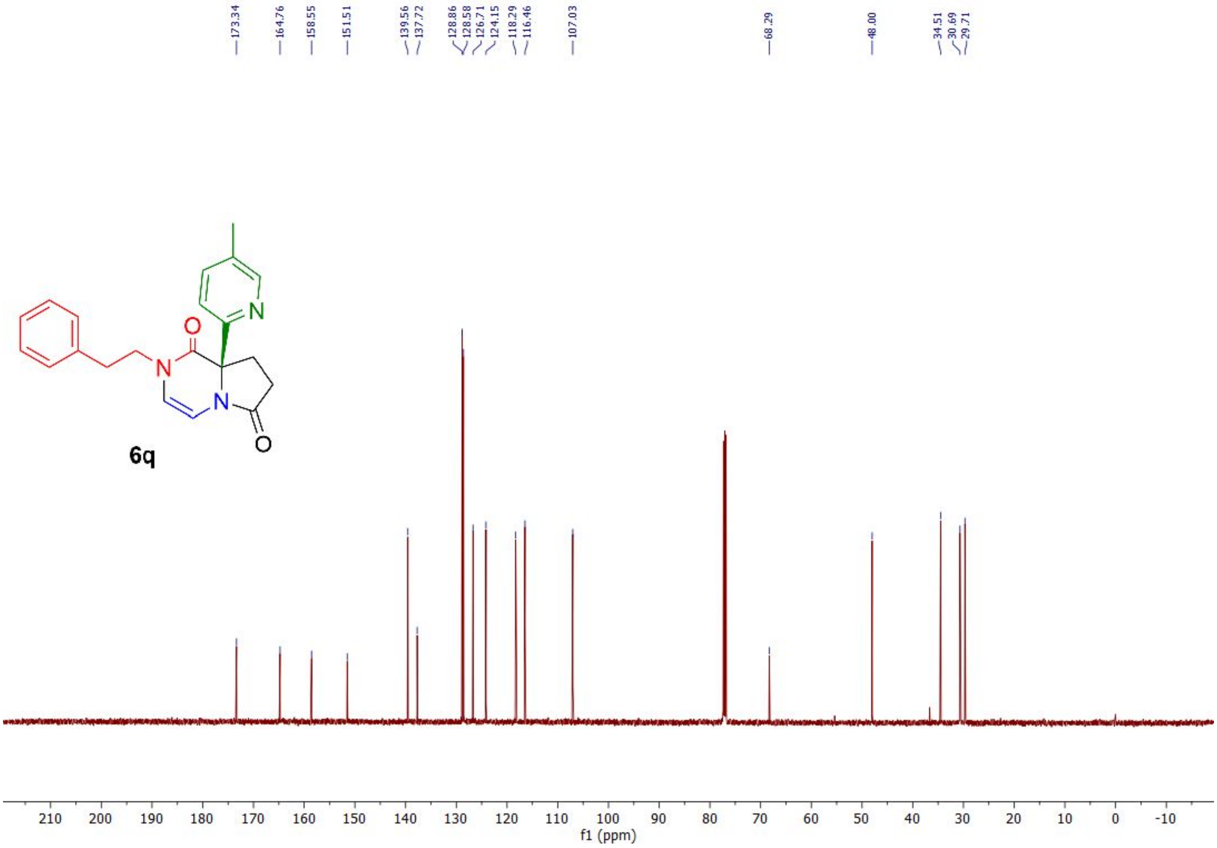

^{1}NMR spectrum of **6r** (500 MHz, $CDCl_3$)

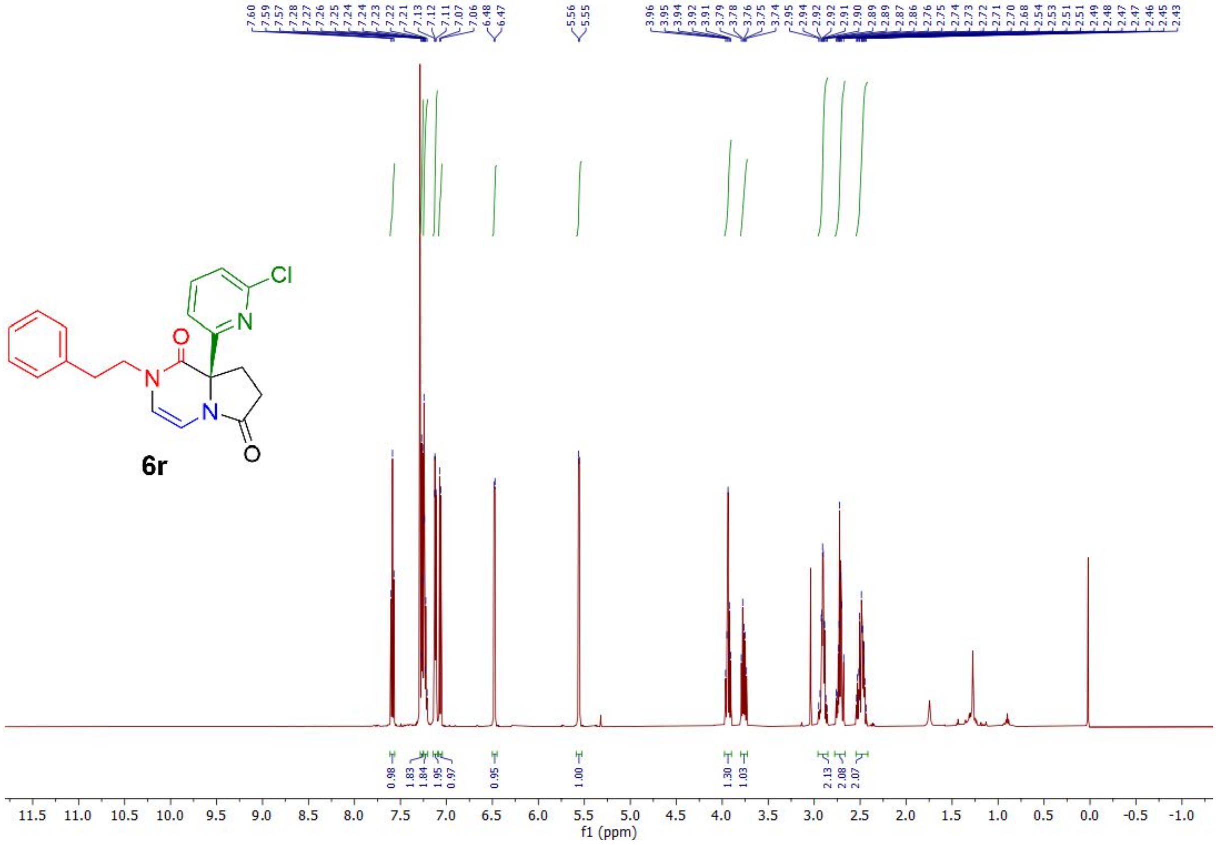

$^{13}C\{^{1}H\}$ NMR spectrum of **6r** (126 MHz, $CDCl_3$)

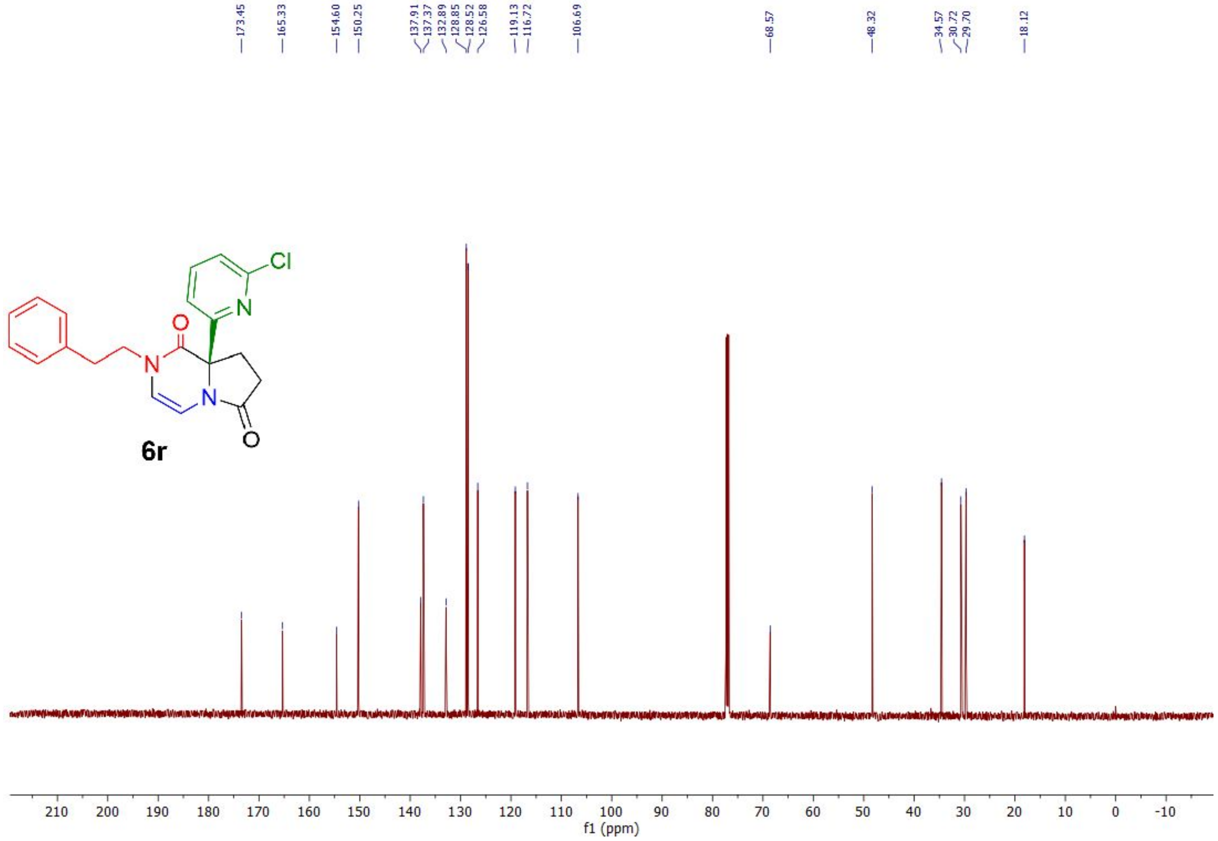

[1]NMR spectrum of **6s** (500 MHz, $CDCl_3$)

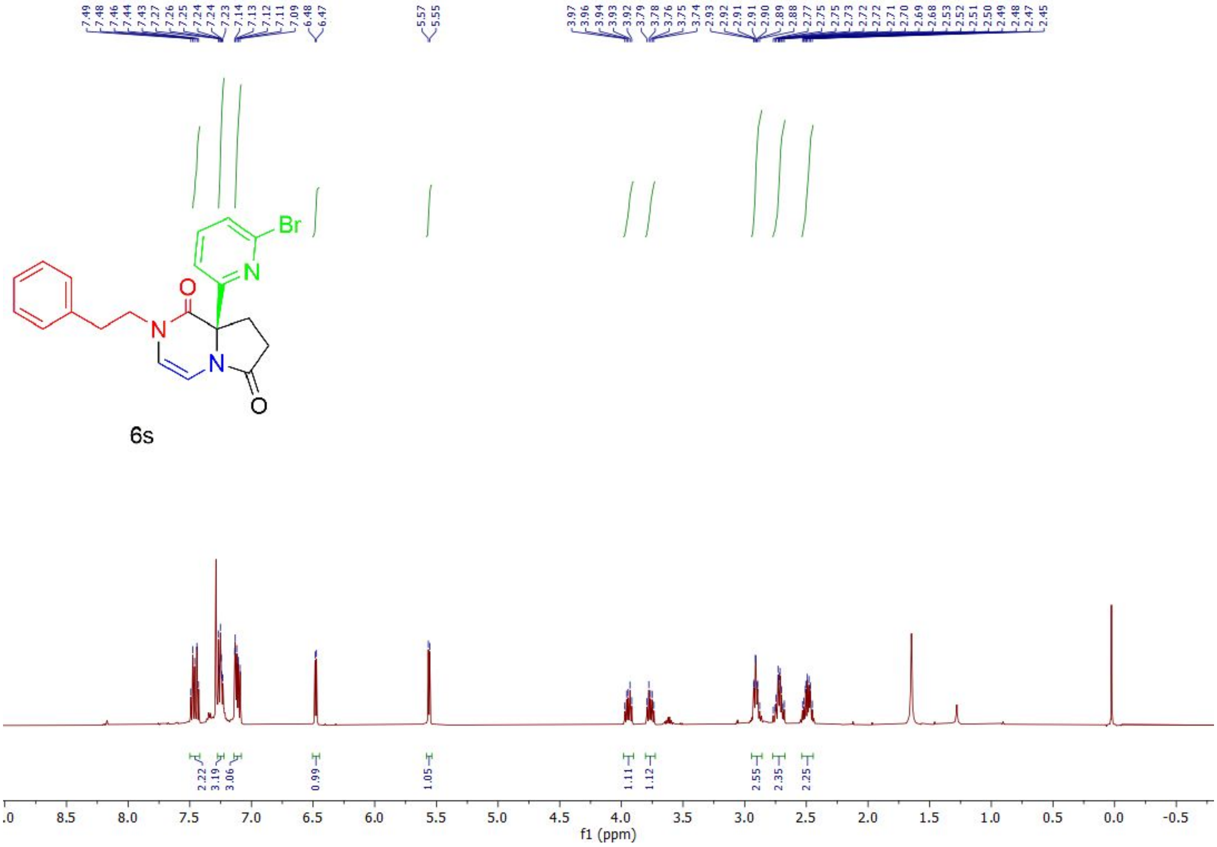

$^{13}C\{^1H\}$ NMR spectrum of **6s** (126 MHz, $CDCl_3$)

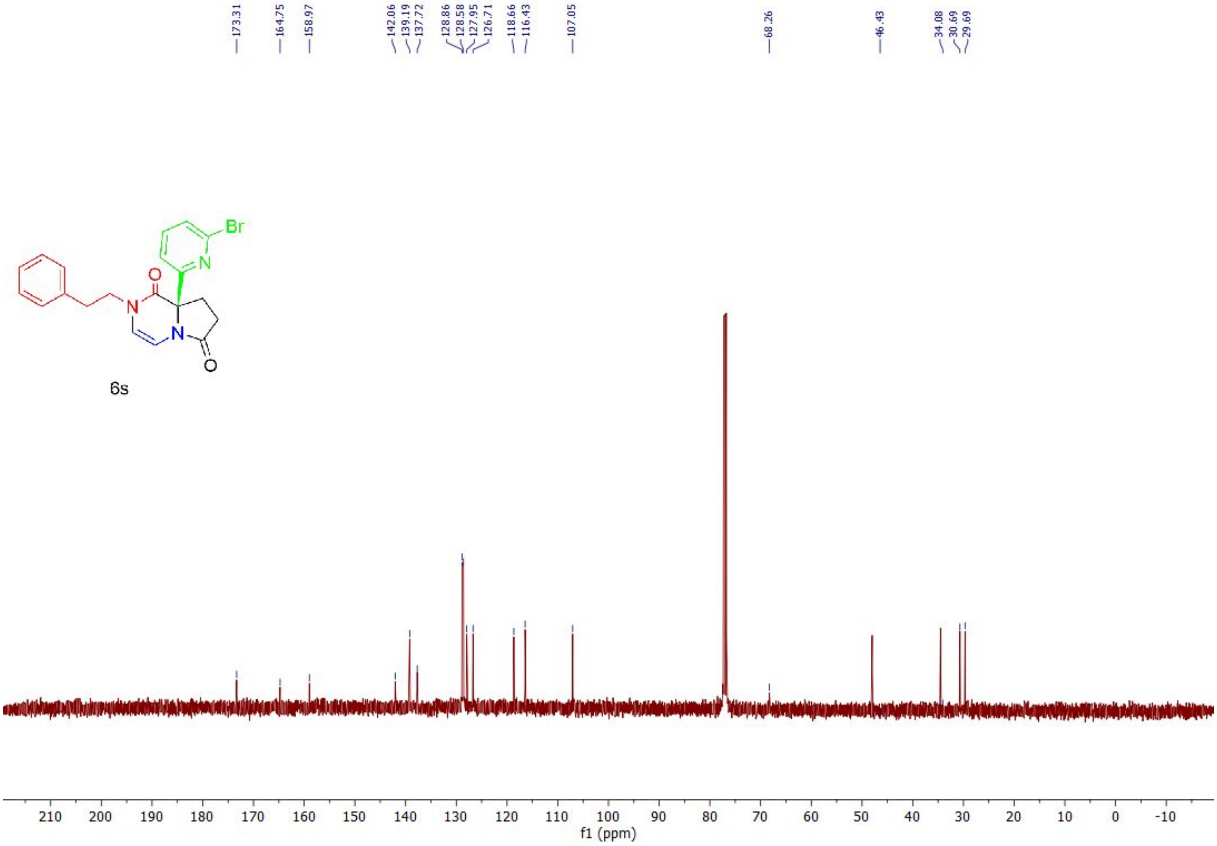

^{1}NMR spectrum of **6t** (500 MHz, $CDCl_3$)

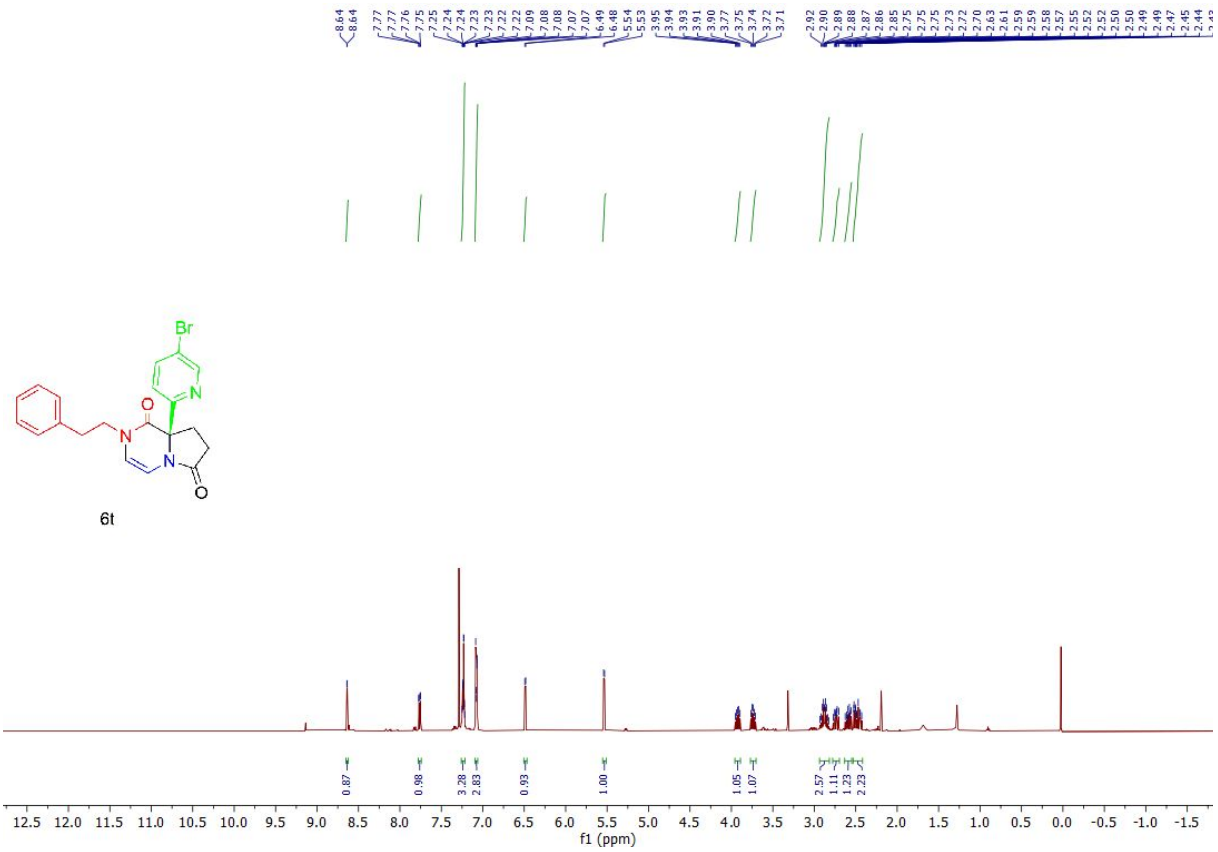

$^{13}C\{^{1}H\}$ NMR spectrum of **6t** (126 MHz, $CDCl_3$)

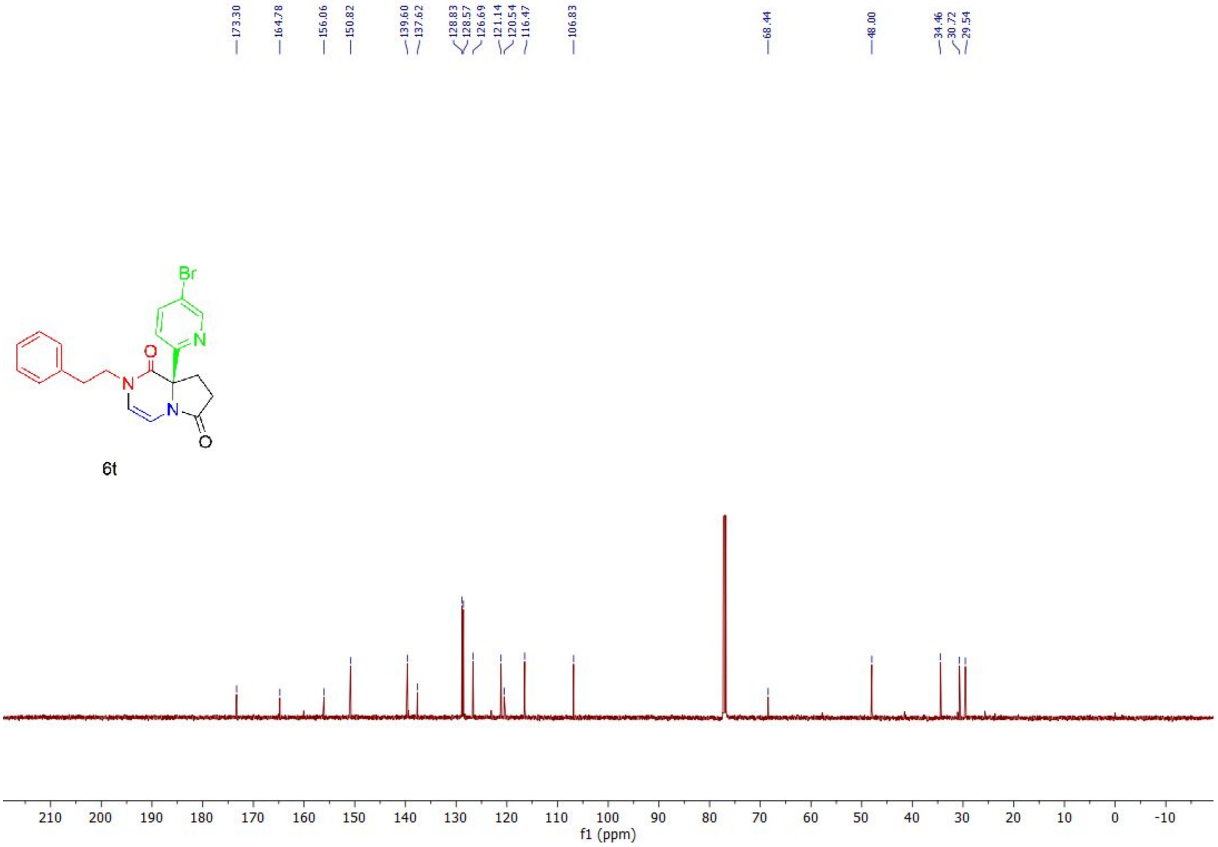

^{1}NMR spectrum of **6u** (500 MHz, $CDCl_3$)

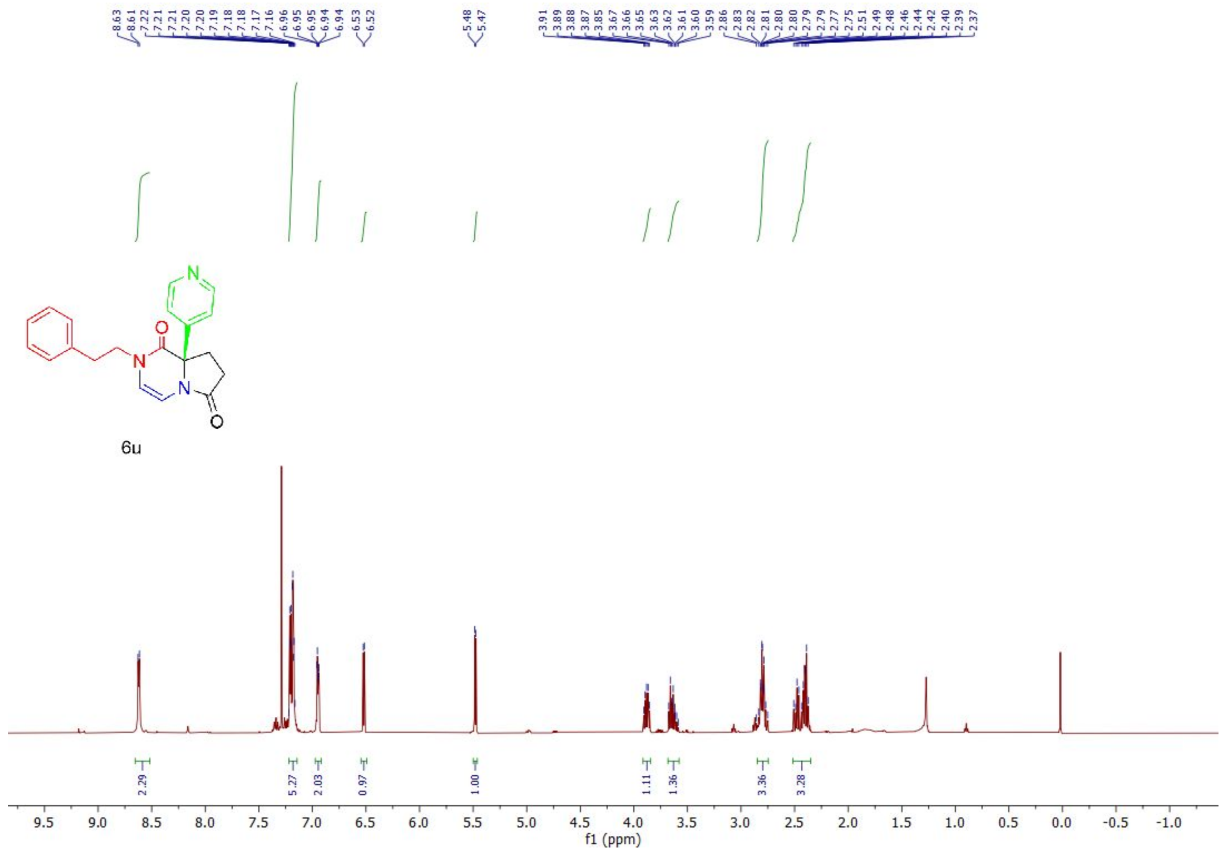

$^{13}C\{^{1}H\}$ NMR spectrum of **6u** (126 MHz, $CDCl_3$)

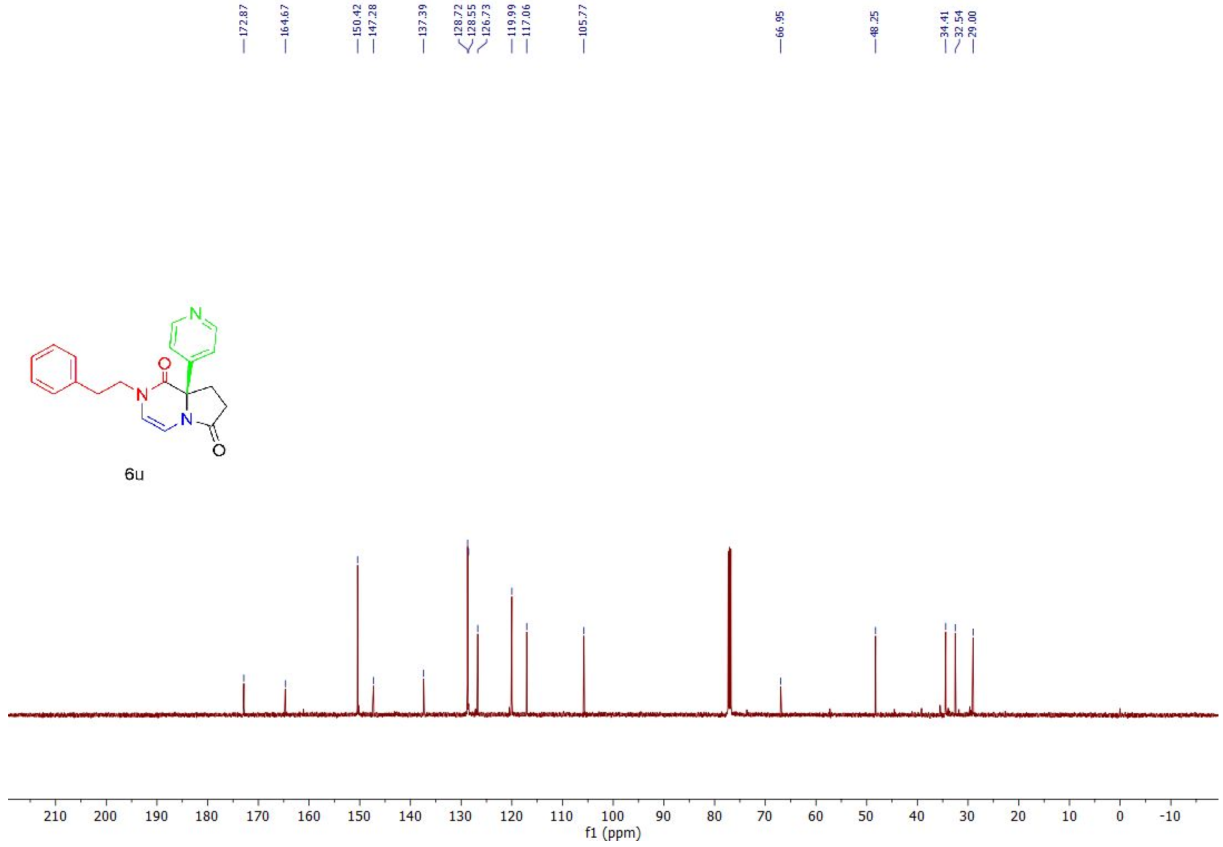

^{1}NMR spectrum of **7a** (500 MHz, $CDCl_3$)

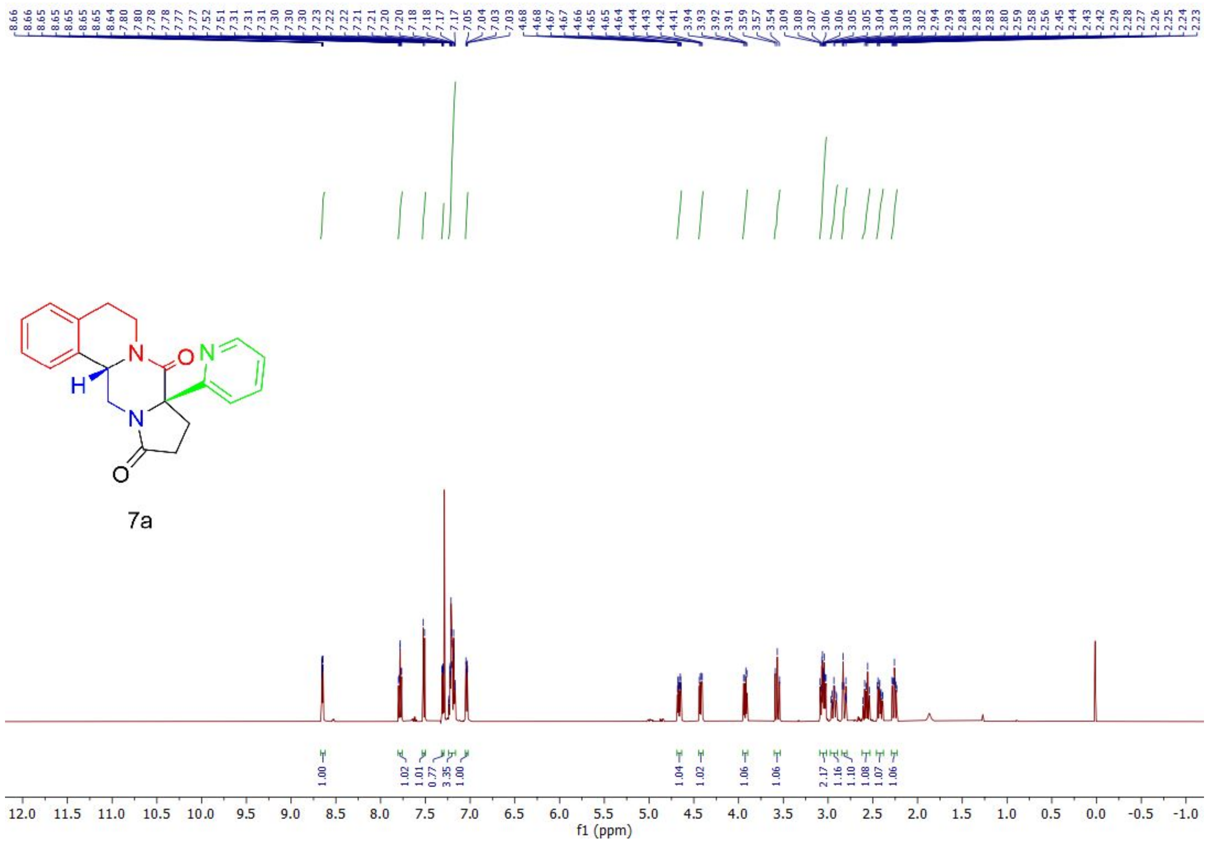

$^{13}C\{^{1}H\}$ NMR spectrum of **7a** (126 MHz, $CDCl_3$)

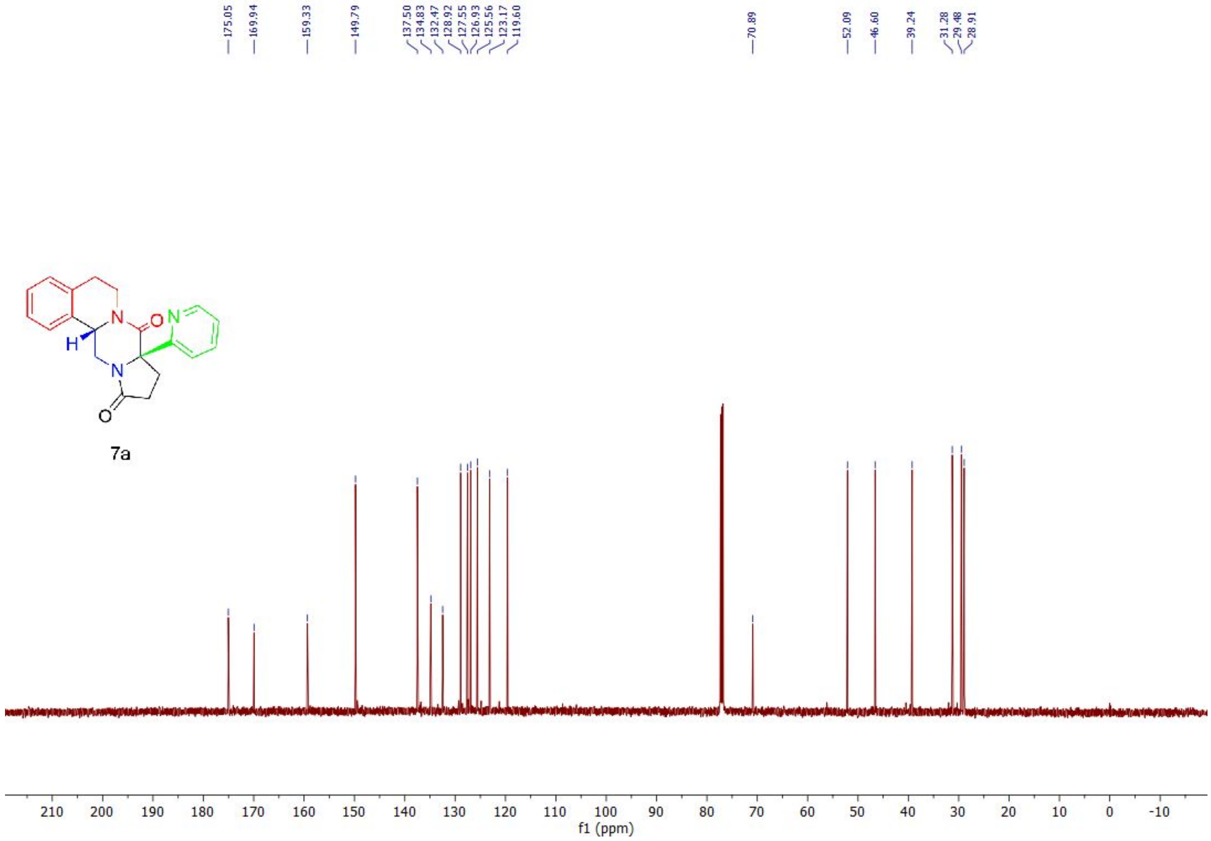

[1]NMR spectrum of **7b** (500 MHz, $CDCl_3$)

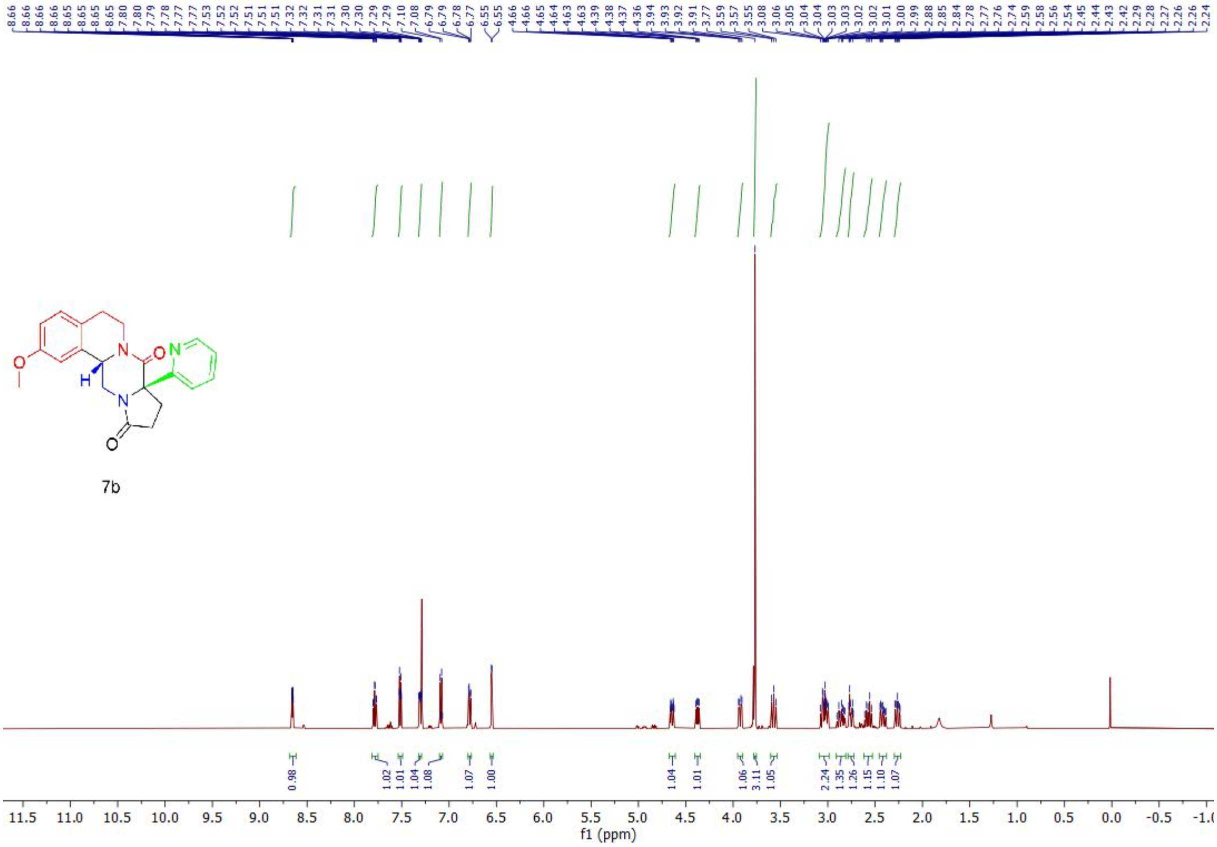

7b

^{13}C{^{1}H} NMR spectrum of **7b** (126 MHz, $CDCl_3$)

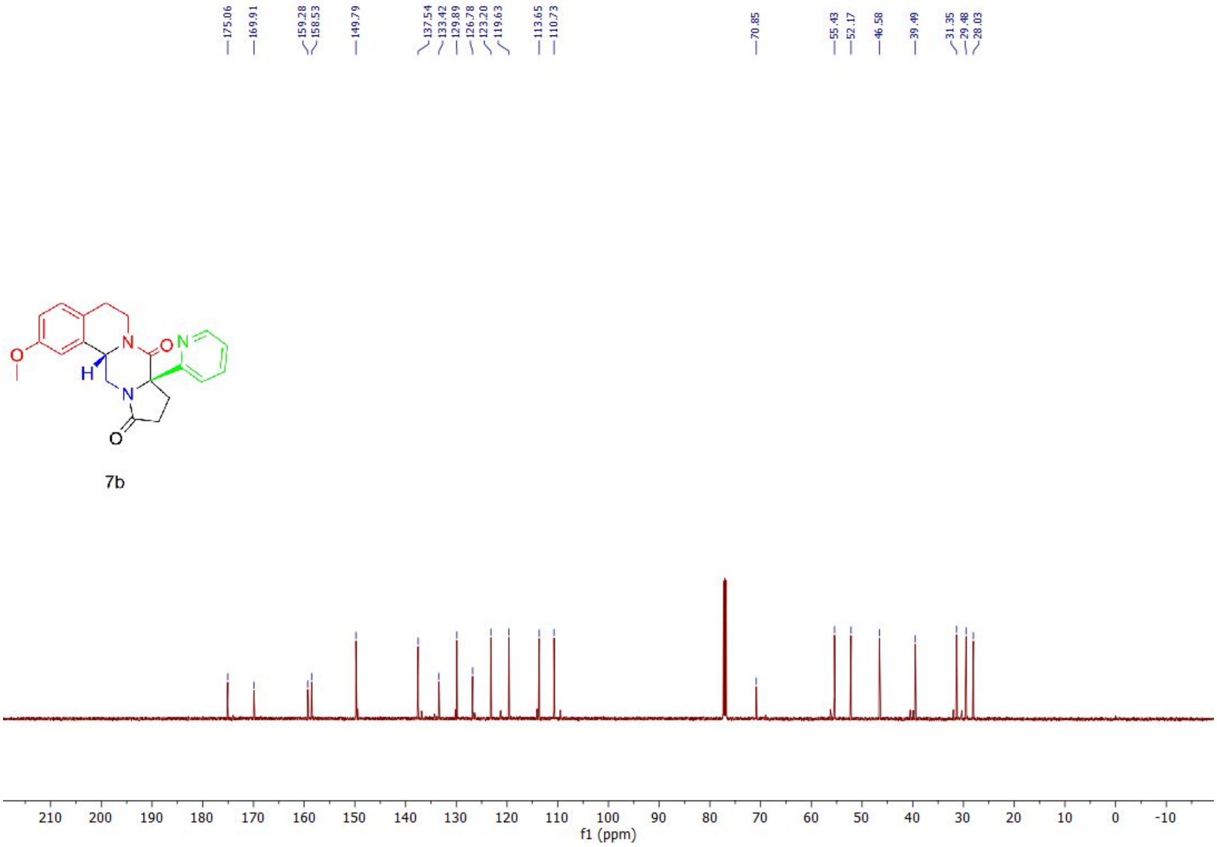

7b

[1]NMR spectrum of **7c** (500 MHz, $CDCl_3$)

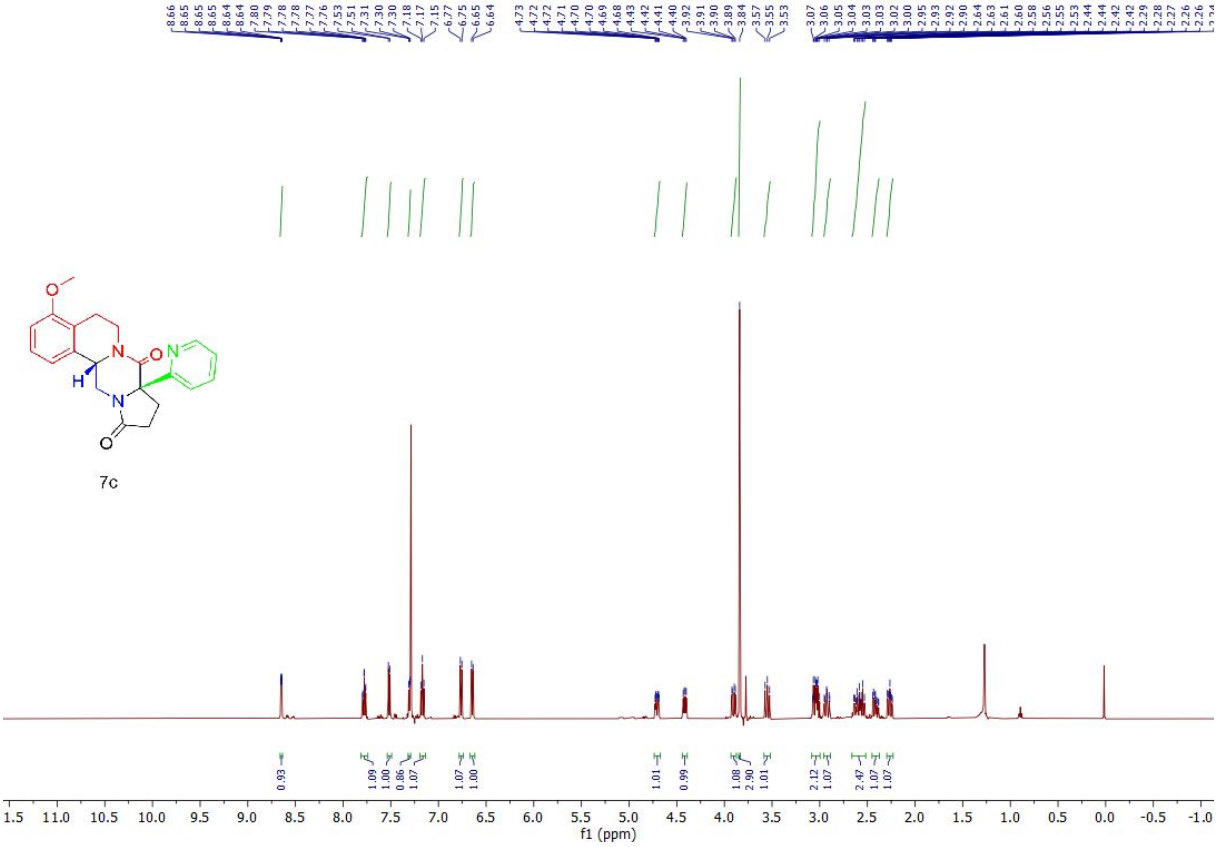

$^{13}C\{^{1}H\}$ NMR spectrum of **7c** (126 MHz, $CDCl_3$)

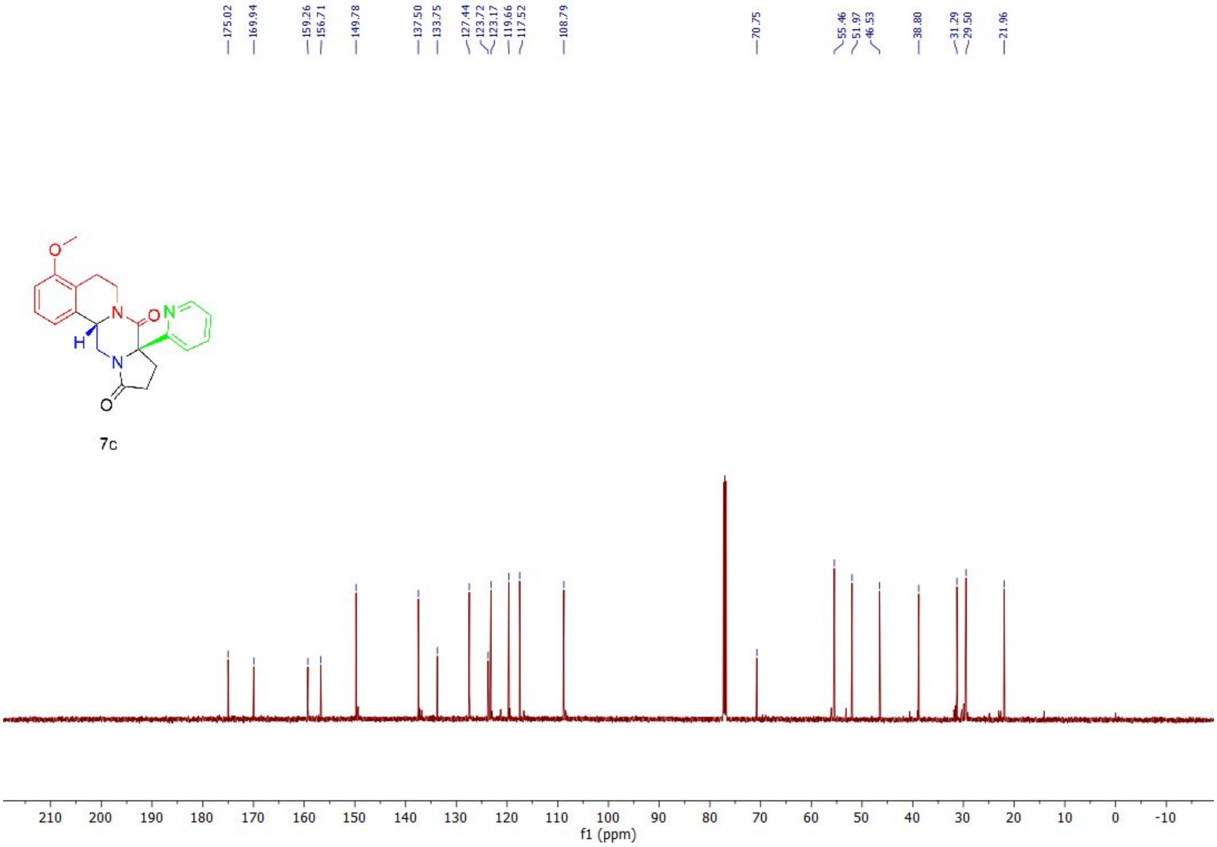

^{1}NMR spectrum of **7d** (500 MHz, $CDCl_3$)

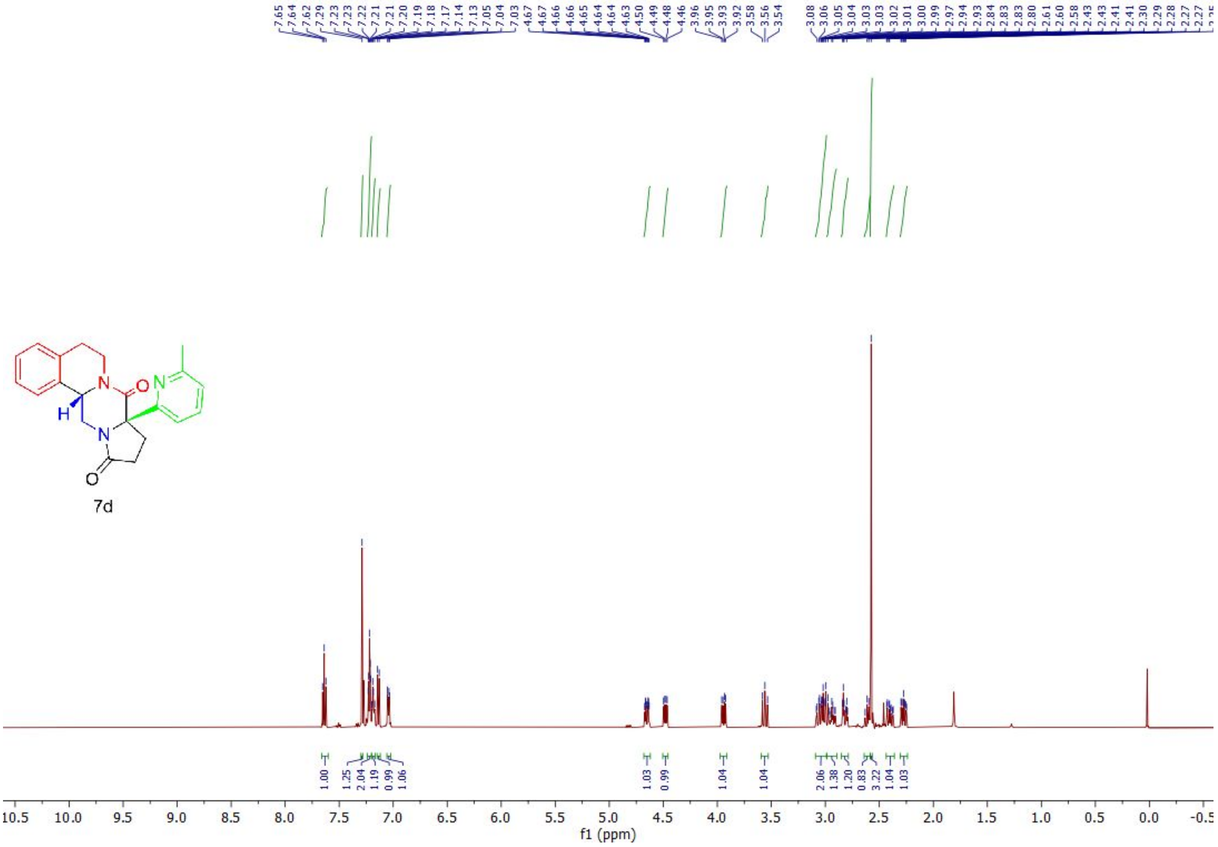

$^{13}C\{^1H\}$ NMR spectrum of **7d** (126 MHz, $CDCl_3$)

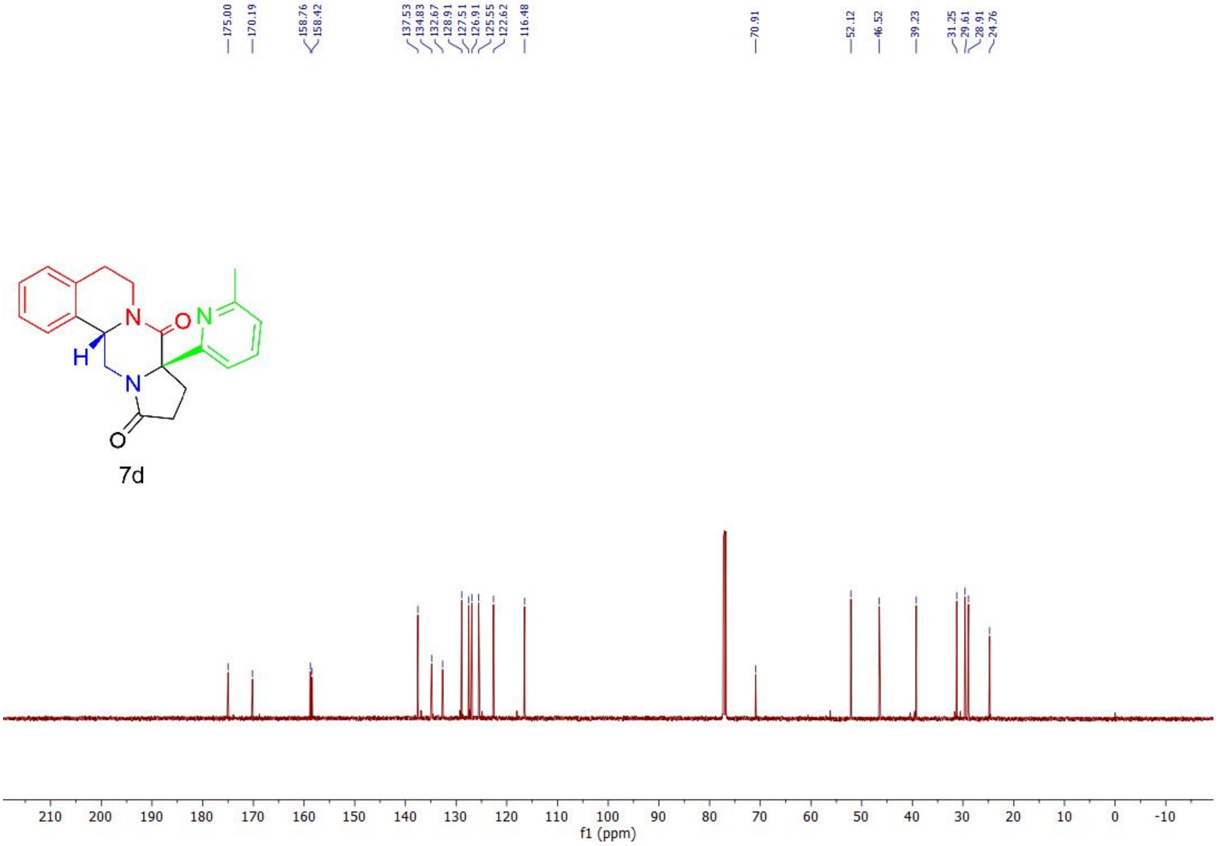

^{1}NMR spectrum of **7e** (500 MHz, $CDCl_3$)

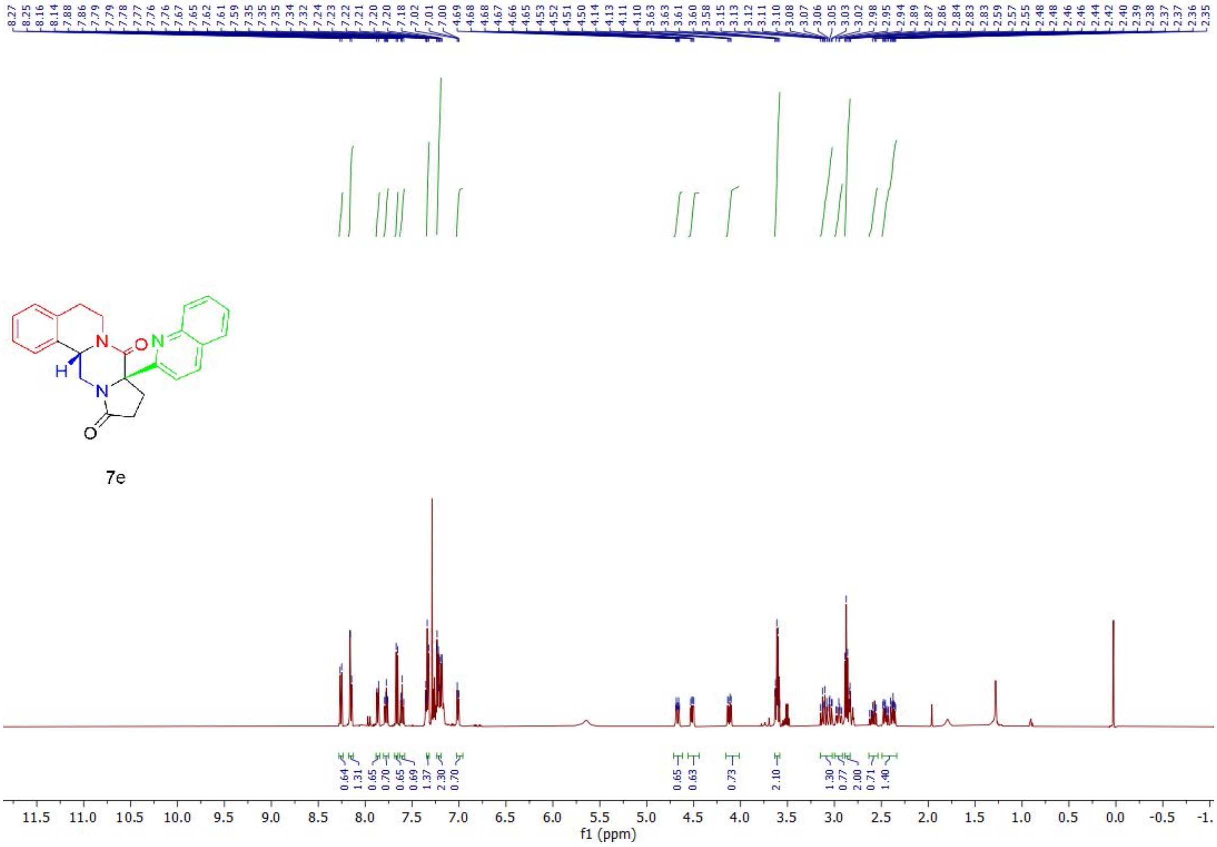

$^{13}C\{^{1}H\}$ NMR spectrum of **7e** (126 MHz, $CDCl_3$)

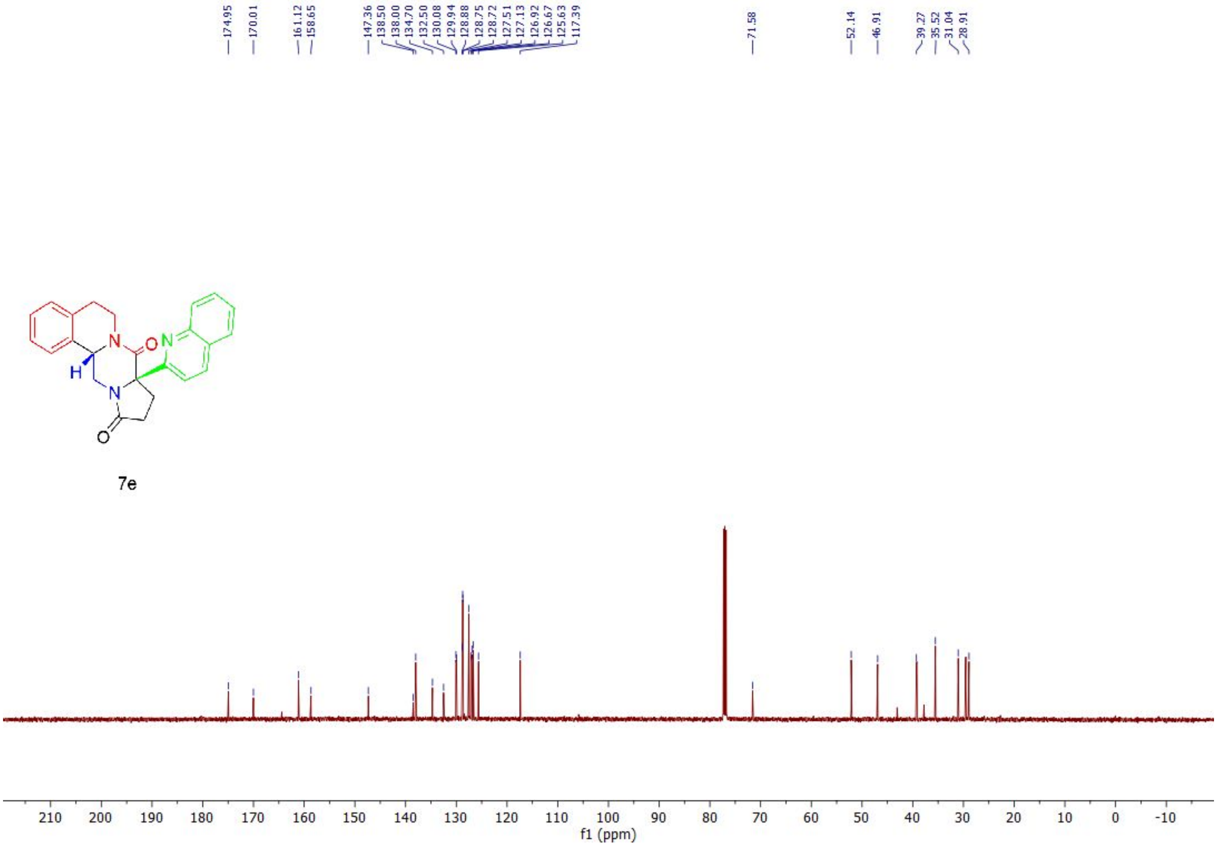

[1]NMR spectrum of **7f** (500 MHz, $CDCl_3$)

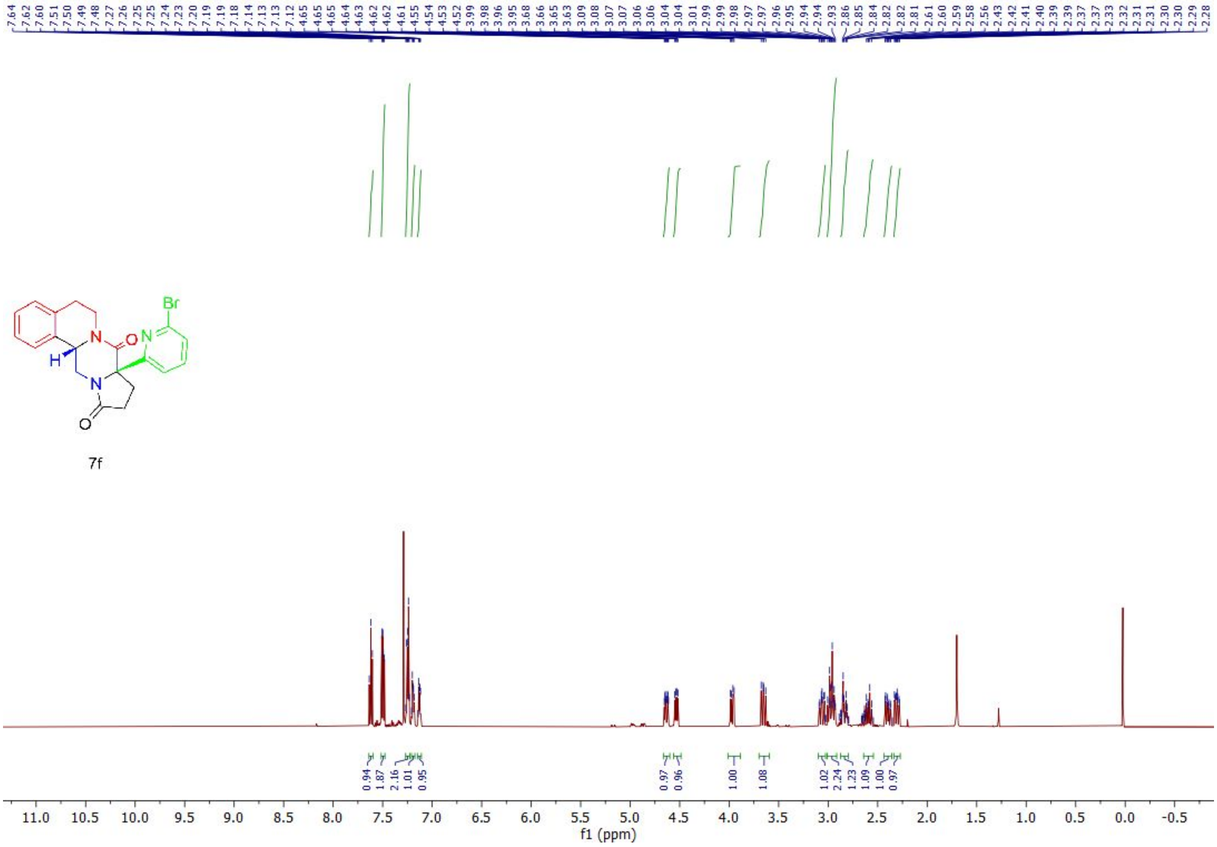

$^{13}C\{^1H\}$ NMR spectrum of **7f** (126 MHz, $CDCl_3$)

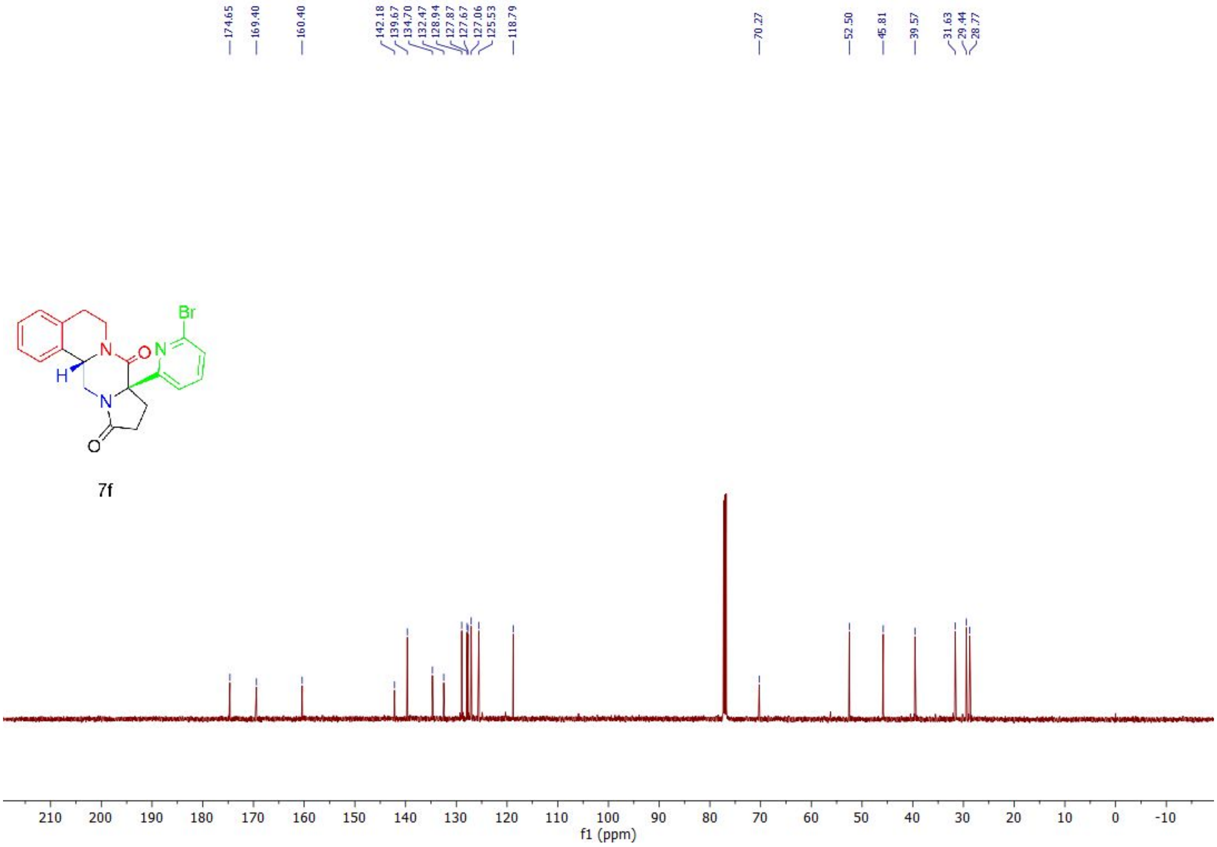

^{1}NMR spectrum of **7g** (500 MHz, $CDCl_3$)

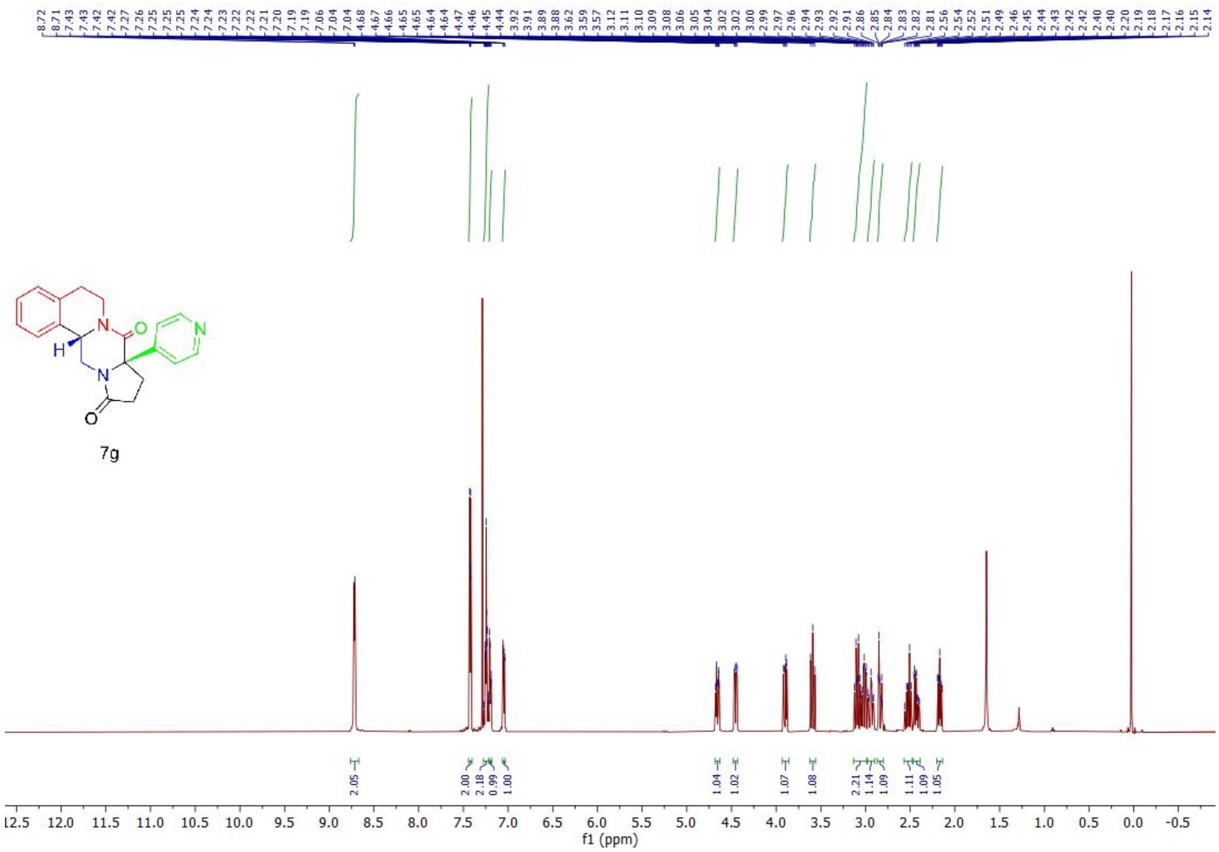

^{13}C{^{1}H} NMR spectrum of **7g** (126 MHz, $CDCl_3$)

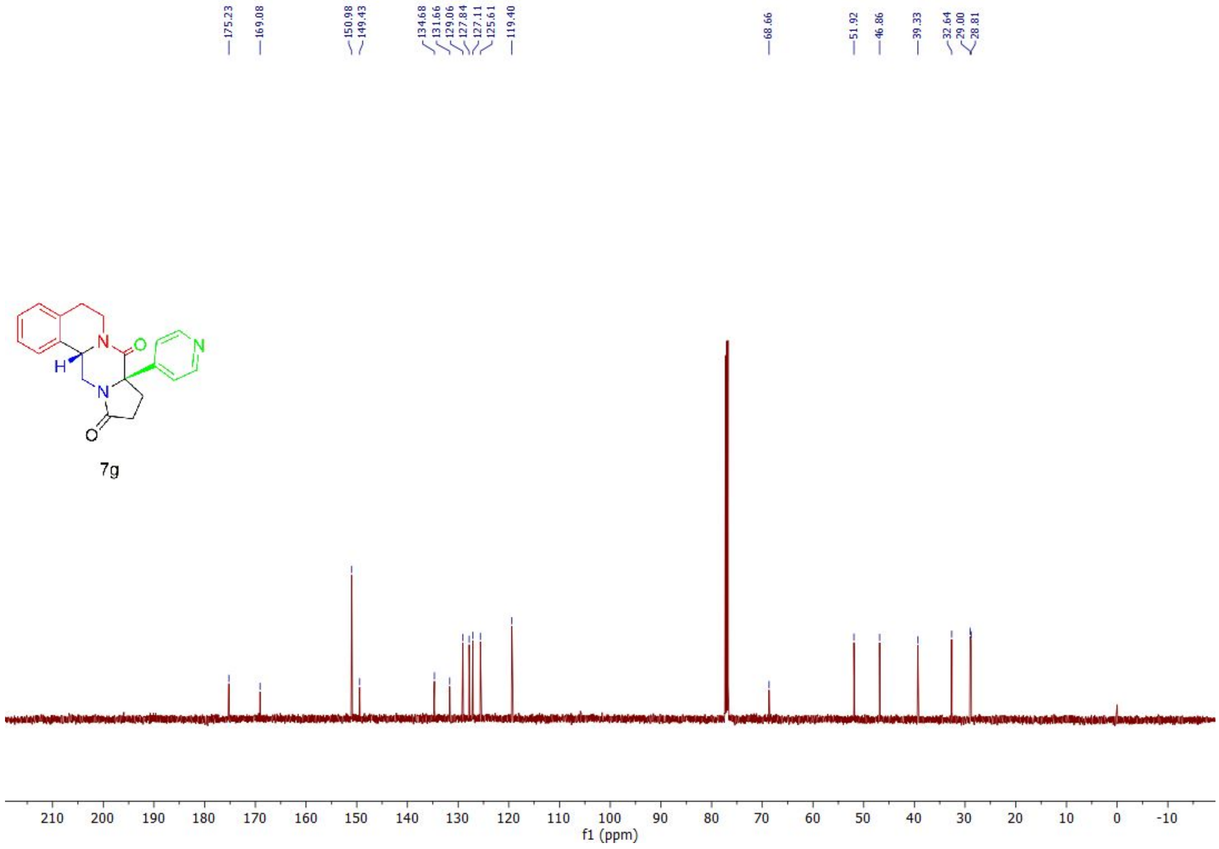

[1]NMR spectrum of **7h** (500 MHz, $CDCl_3$)

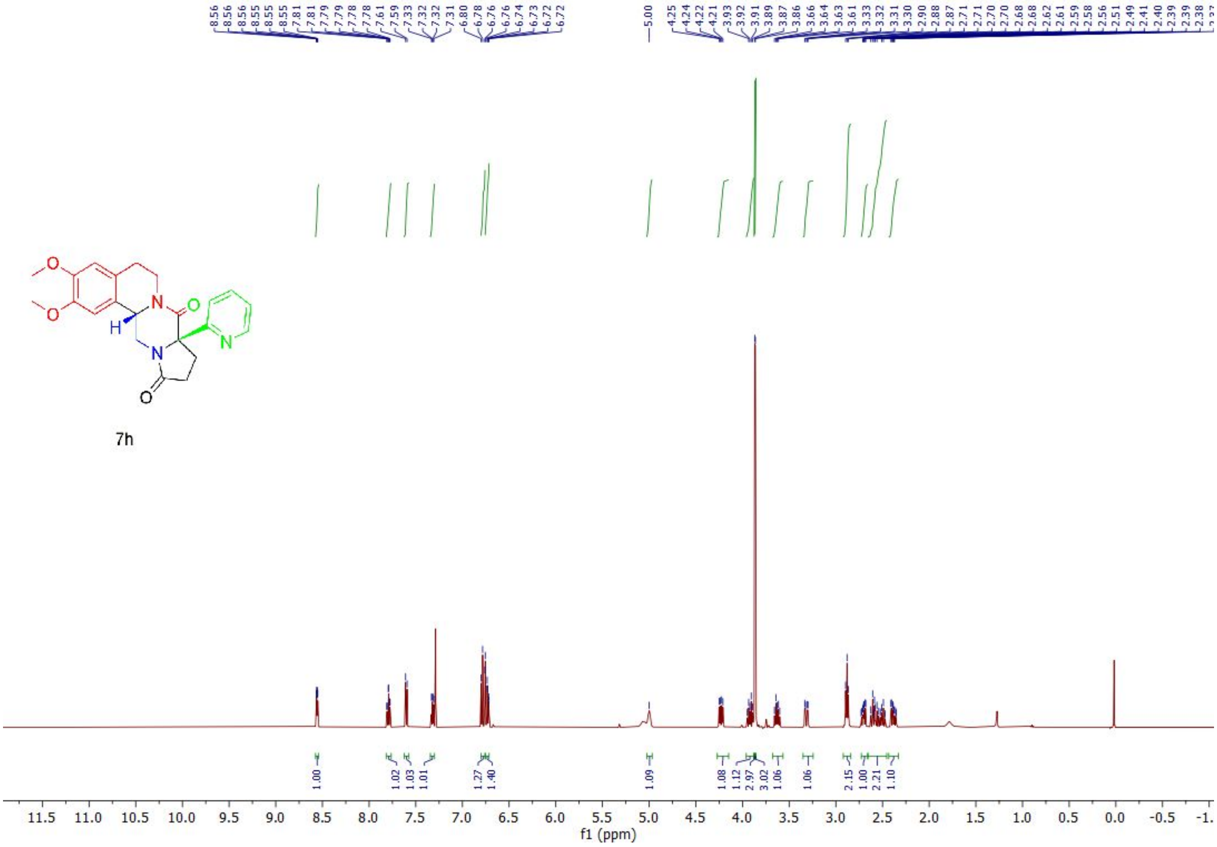

$^{13}C\{^{1}H\}$ NMR spectrum of **7h** (126 MHz, $CDCl_3$)

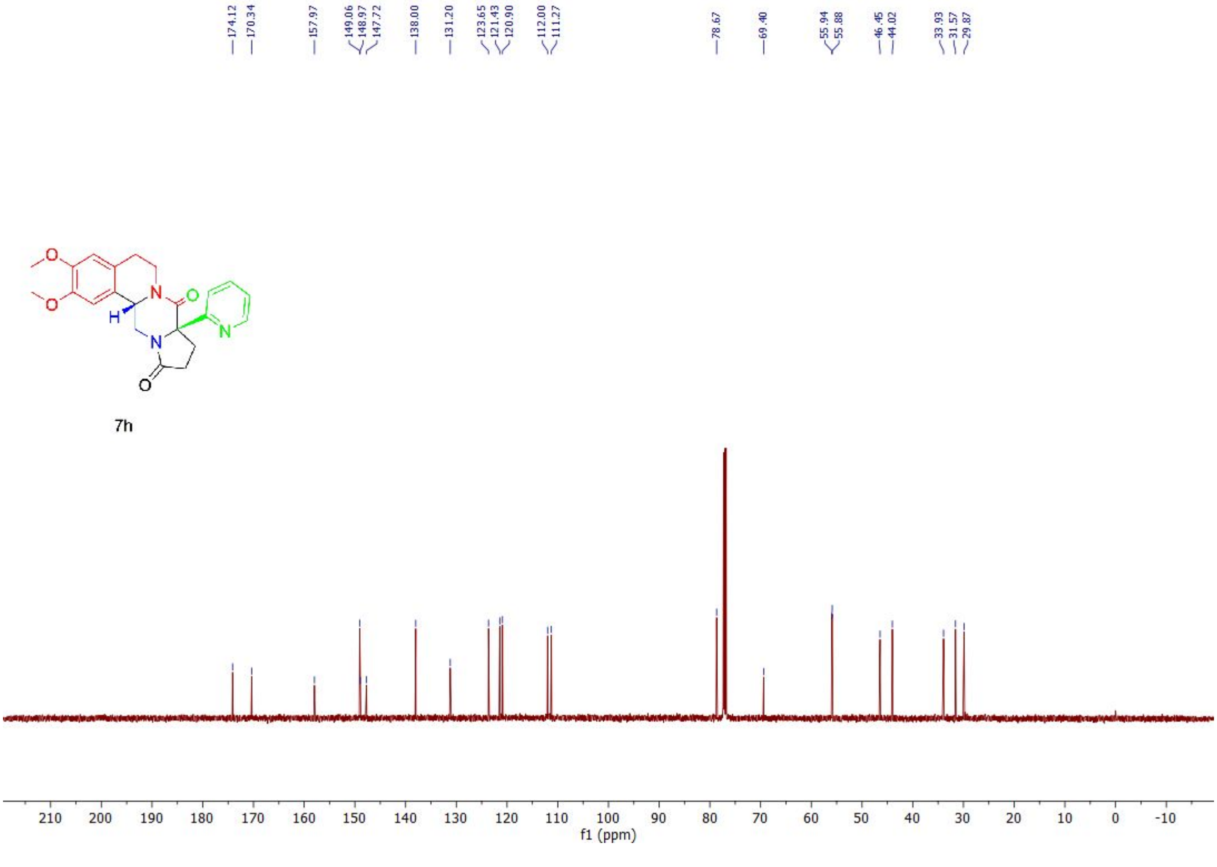

^{1}NMR spectrum of **7i** (500 MHz, $CDCl_3$)

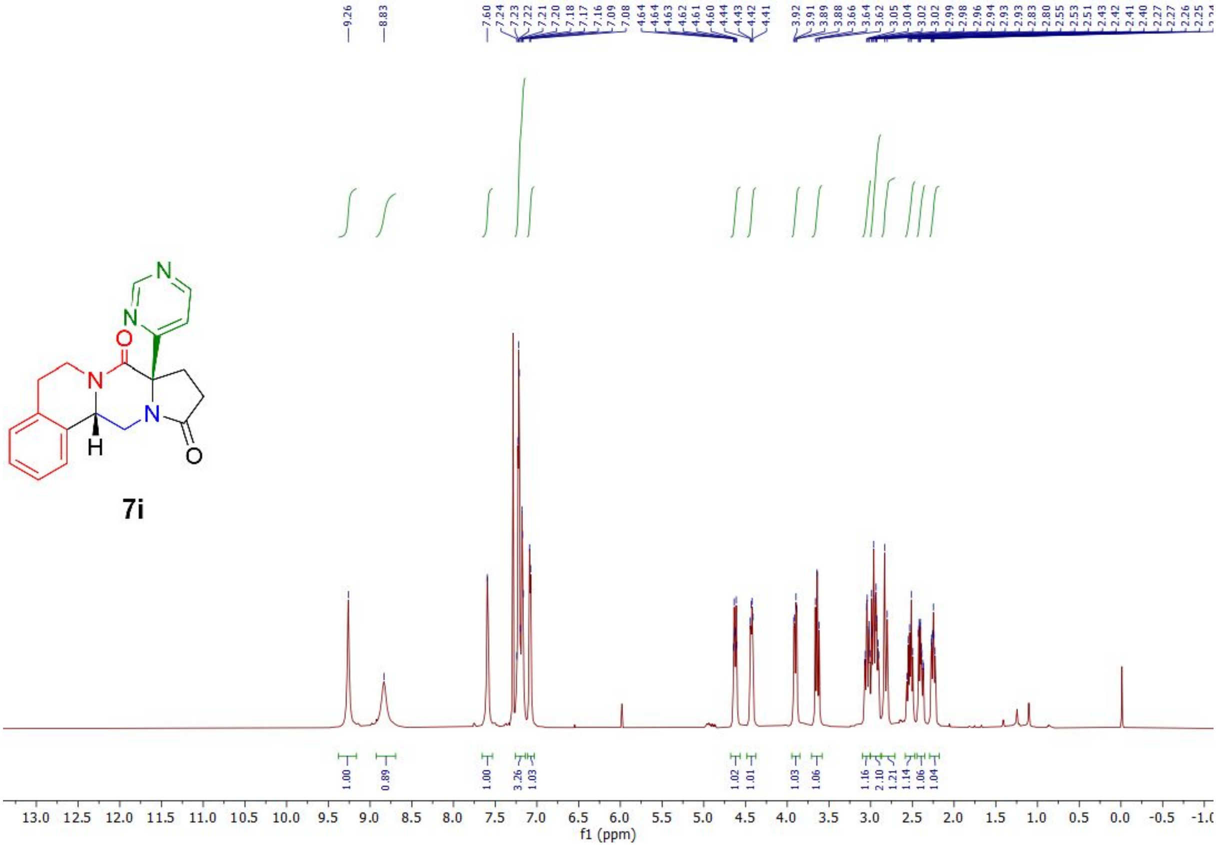

$^{13}C\{^{1}H\}$ NMR spectrum of **7i** (126 MHz, $CDCl_3$)

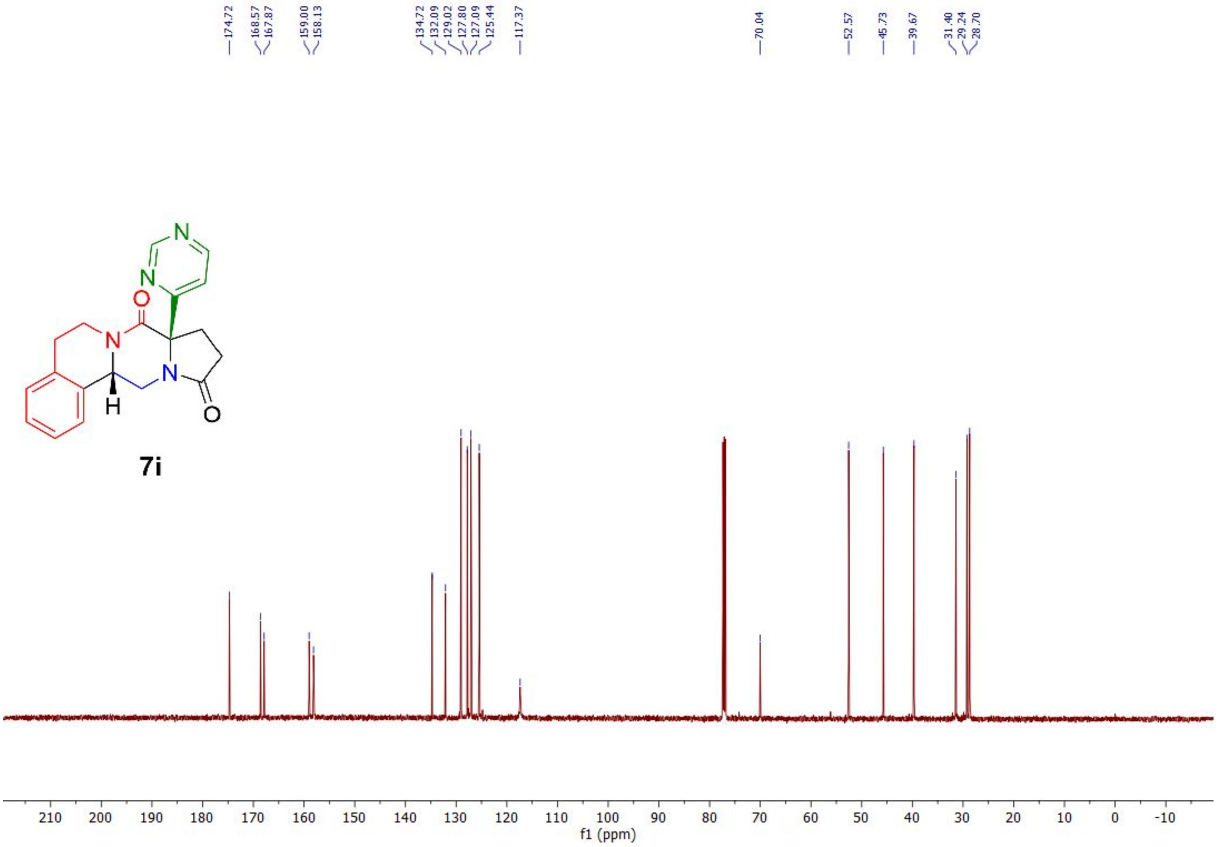

^{1}NMR spectrum of **8a** (500 MHz, $CDCl_3$)

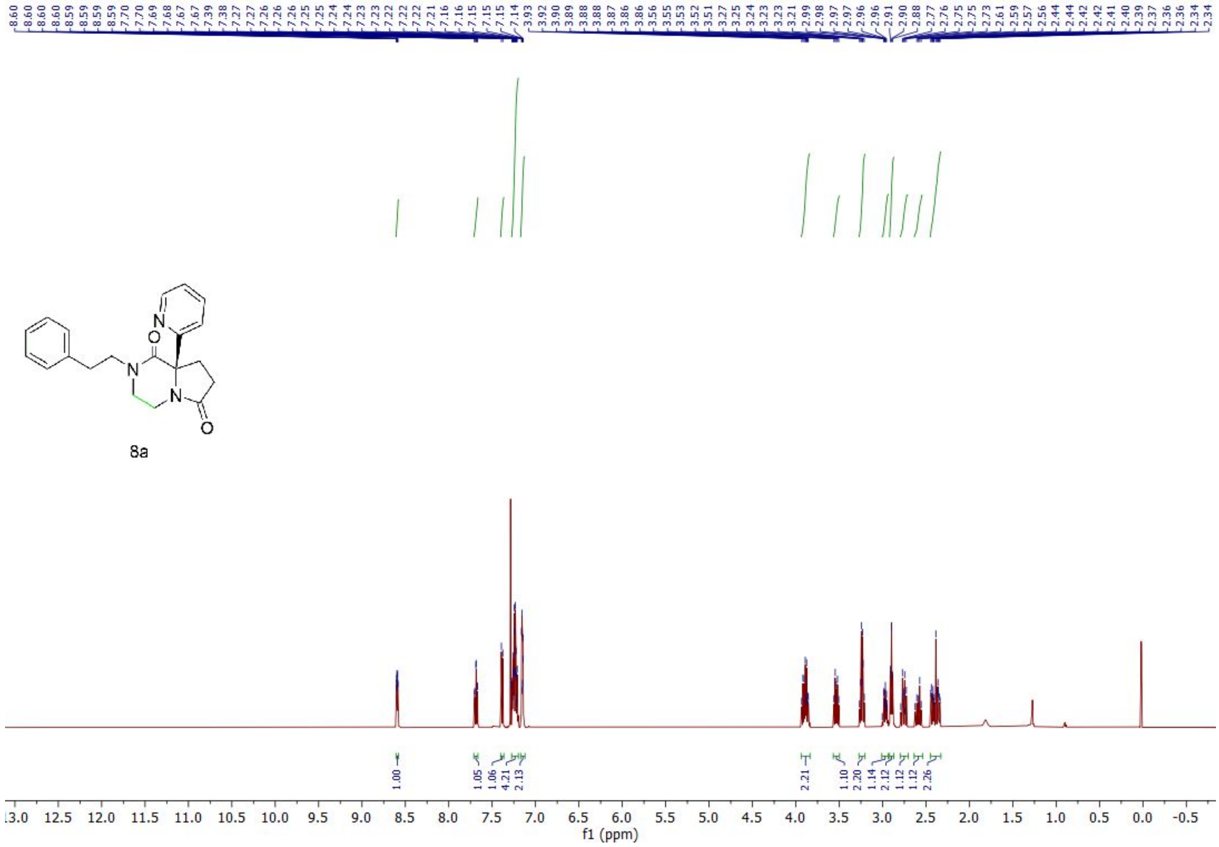

$^{13}C\{^{1}H\}$ NMR spectrum of **8a** (126 MHz, $CDCl_3$)

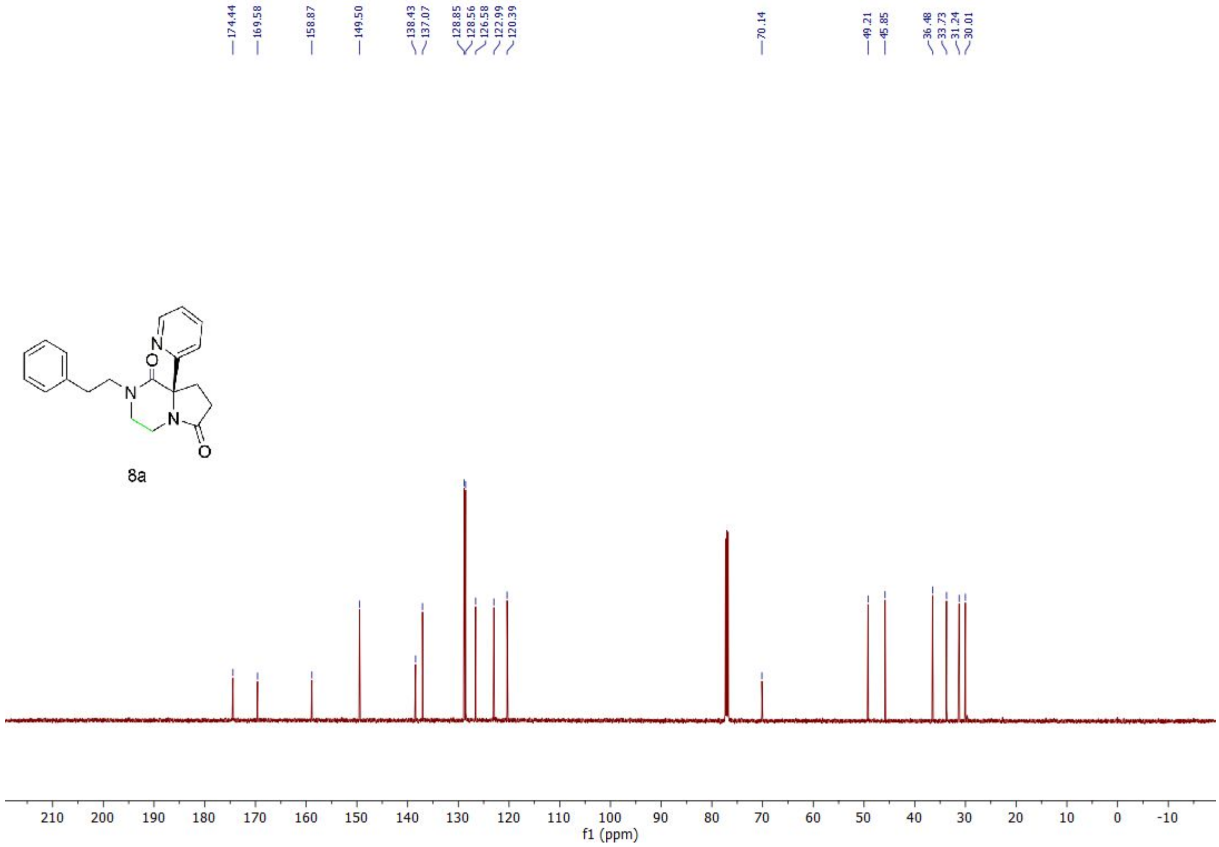

[1]NMR spectrum of **8b** (500 MHz, $CDCl_3$)

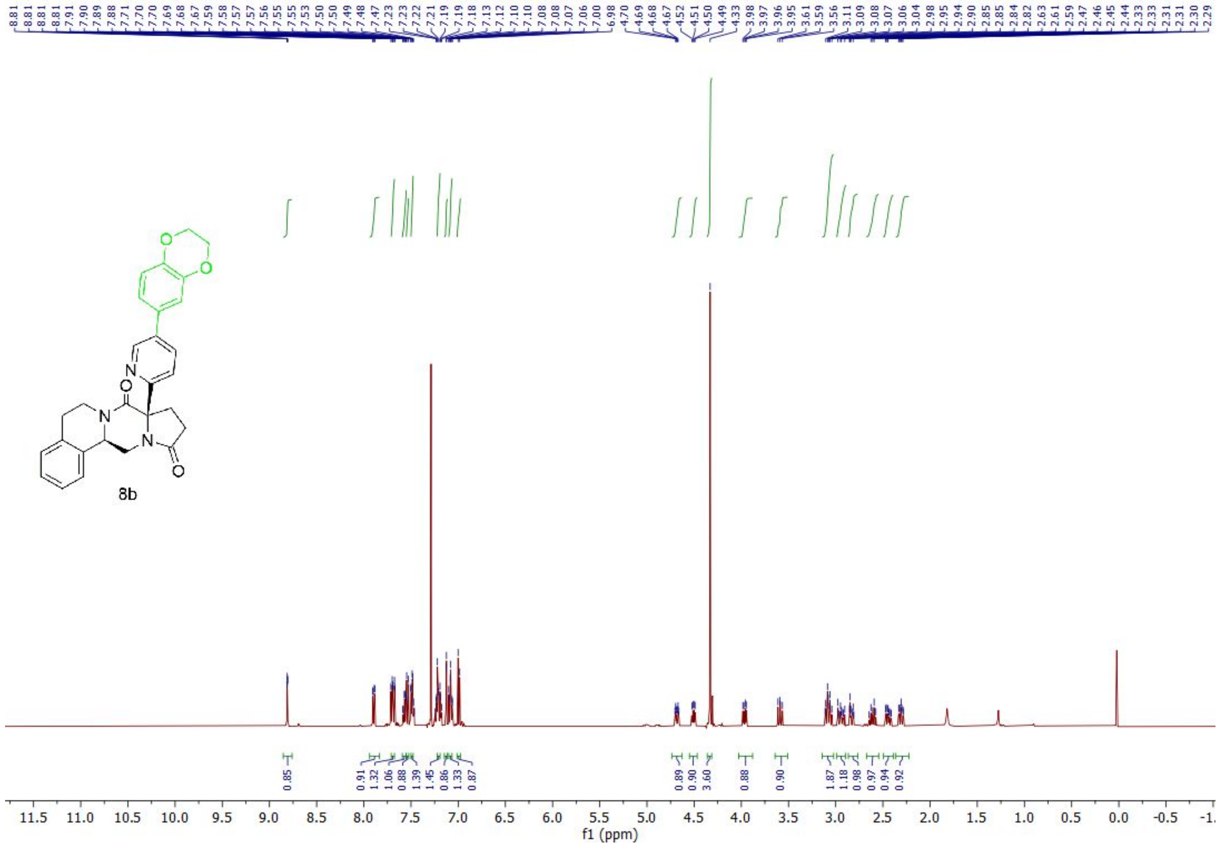

$^{13}C\{^{1}H\}$ NMR spectrum of **8b** (126 MHz, $CDCl_3$)

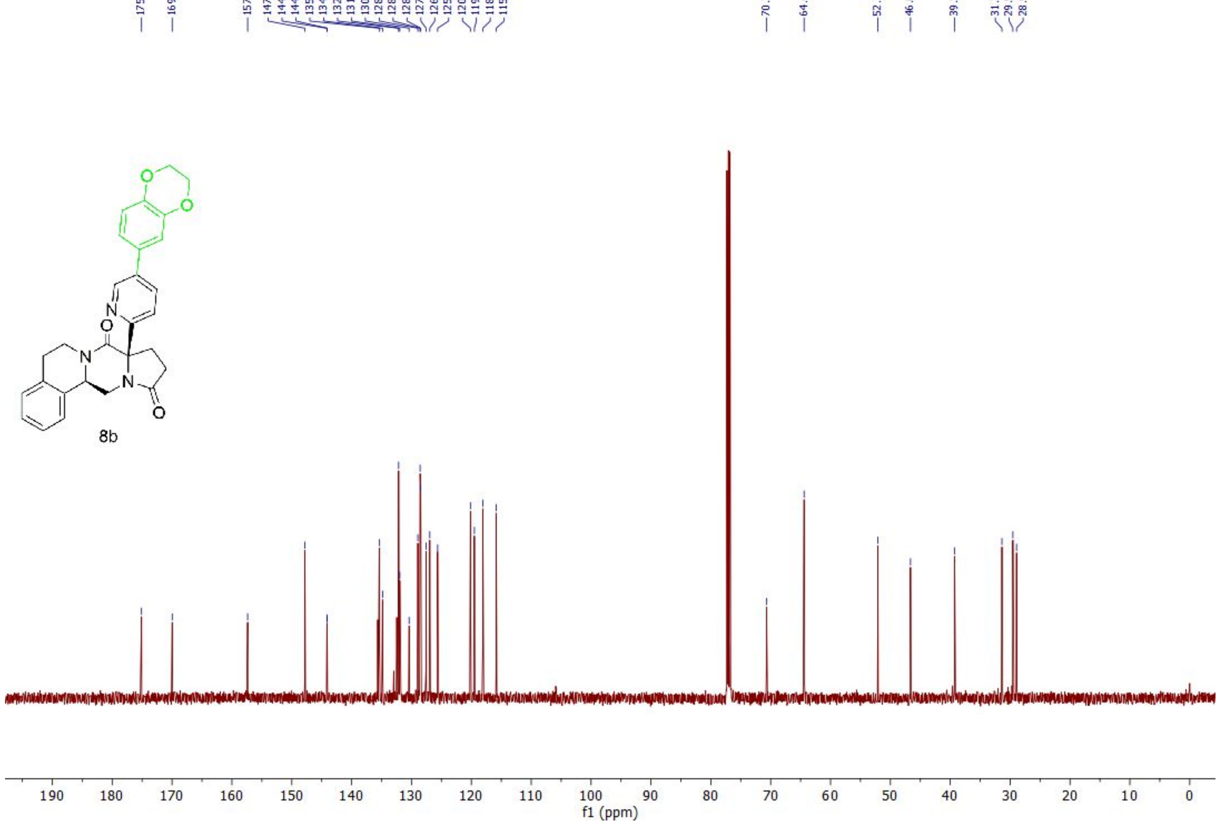

X-ray structure determination

Single crystals were obtained from dichloromethane solutions by vapour diffusion in room temperature. X-ray diffraction data for single crystal of compound **5x** (MEDH_5b), **6a** (BDFF_52) and **7a** (BDH_00) was collected using Rigaku XtaLAB Synergy S Dualflex diffractometer (four circle diffractometer with a mirror monochromator) with HyPix detector and a PhotonJet CuKα radiation source (λ = 1.54184 Å) for **5x, 6a** and **7a**. Additionally, the diffractometer was equipped with a CryoJet HT cryostat system (Oxford Instruments) allowing low temperature experiments, performed at 100 (11) K. The obtained data set was processed with CrysAlisPro software [S1]. The phase problem was solved by direct methods using SUPERFLIP [S2]. Parameters of obtained models were refined by full-matrix least-squares on F^2 using SHELXL-2014/6 [S3]. Calculations were performed using WinGX integrated system (ver. 2014.1) [S4]. Figure was prepared with Mercury 3.7 software [S5].

All non-hydrogen atoms were refined anisotropically. All hydrogen atoms attached to carbon atoms were positioned with the idealised geometry and refined using the riding model with the isotropic displacement parameter U_{iso}[H] = 1.2 (or 1.5 (methyl groups only)) U_{eq}[C]. Crystal data and structure refinement results for presented crystal structure are shown in Table S1. The molecular geometry (asymmetric unit) observed in the crystal structure is shown in Figure S1.

Crystallographic data have been deposited with the Cambridge Crystallographic Data Centre as supplementary publication no. CCDC 2141575 (**5x**), no. CCDC 2089898 (**6a**), CCDC 2089897 (**7a**). Copies of the data can be obtained, free of charge, on application to CCDC, 12 Union Road, Cambridge CB2 1EZ, UK, (fax: +44-(0)1223-336033 or e-mail: deposit@ccdc.cam.ac.uk).

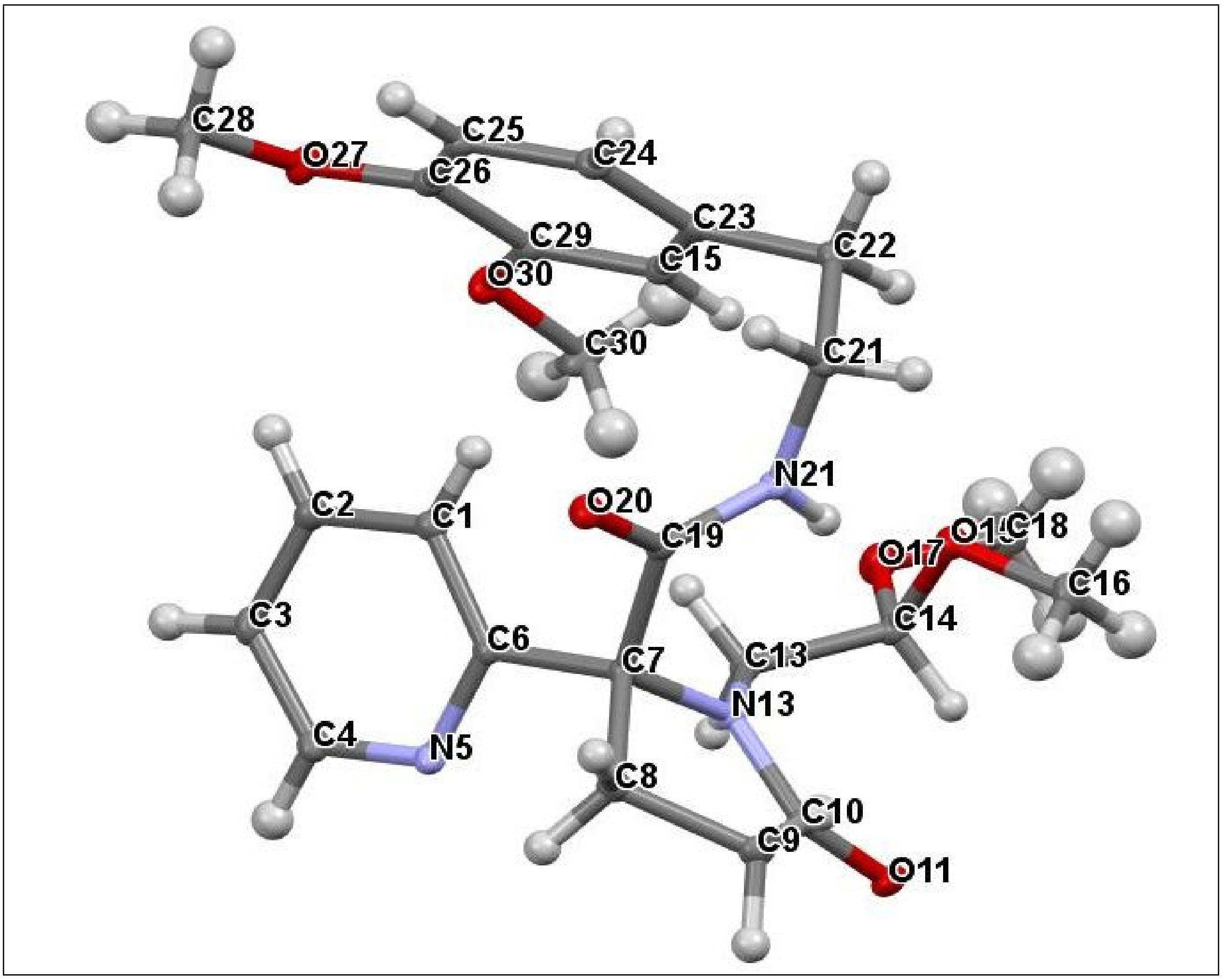

Figure S1. Molecular geometry observed in the crystal structure of compound **5x**(MEDH_5b) showing the atom labelling scheme. The positional disorder within the benzene ring is observed with equal site occupancy (50:50). Displacement ellipsoids of non-hydrogen atoms are drawn at the 30% probability level. H atoms are presented as small spheres with an arbitrary radius.

Table S1. Crystal data and structure refinement results for compound **5x**.

	**5x**
Empirical moiety formula	$C_{24} H_{31} N_3 O_6$
Formula weight [g/mol]	457.52
Crystal system	Monoclinic
Space group	$P2_1/c$
Unite cell dimensions	a = 13.48122(11) Å<br>b = 9.64068(8) Å<br>c = 17.57934(13) Å<br>α=90°<br>β= 93.4732(7)°<br>γ=90°
Volume [Å^3]	2280.56(3)
Z	4
D_{calc} [Mg/m^3]	1.332
μ [mm^{-1}]	4.814
F(000)	976
Crystal size [mm^3]	0.2 x 0.15 x 0.15
Θ range	2.52° to 79.58°
Index ranges	-17 ≤ h ≤ 17,<br>-12 ≤ k ≤ 12,<br>-20 ≤ l ≤ 22
Refl. collected	32315
Independent reflections	4912<br>[R(int) = 0.0398]
Completeness [%] to Θ	99.1 (Θ 73.11°)
Absorption correction	Multi-scan
Tmin. and Tmax.	0.888 and 0.905
Data/ restraints/parameters	4912 / 0 / 307
GooF on F2	1.051
Final R indices [I>2sigma(I)]	R1= 0.0357,<br>wR2= 0.0923
R indices (all data)	R1= 0.0372,<br>wR2= 0.0923
$\Delta\rho_{max}$, $\Delta\rho_{min}$ [e·Å^{-3}]	0.33 and -0.23

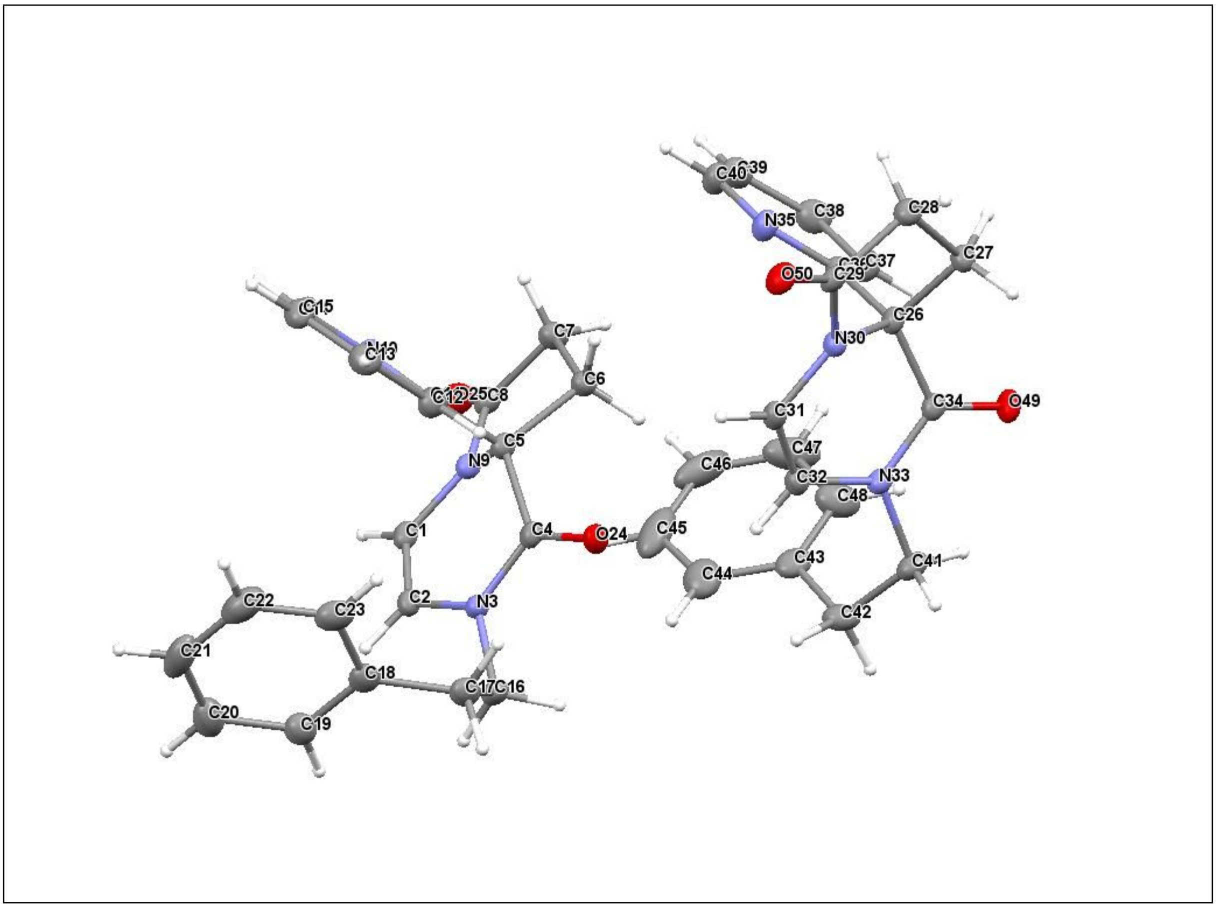

Figure S2. Molecular geometry observed in the crystal structures of compound **6a** (BDFF_52) (asymmetric unit), showing the atom labelling scheme. The positional disorder within the benzene ring is observed with equal site occupancy (50:50). Displacement ellipsoids of non-hydrogen atoms are drawn at the 30% probability level. H atoms are presented as small spheres with an arbitrary radius.

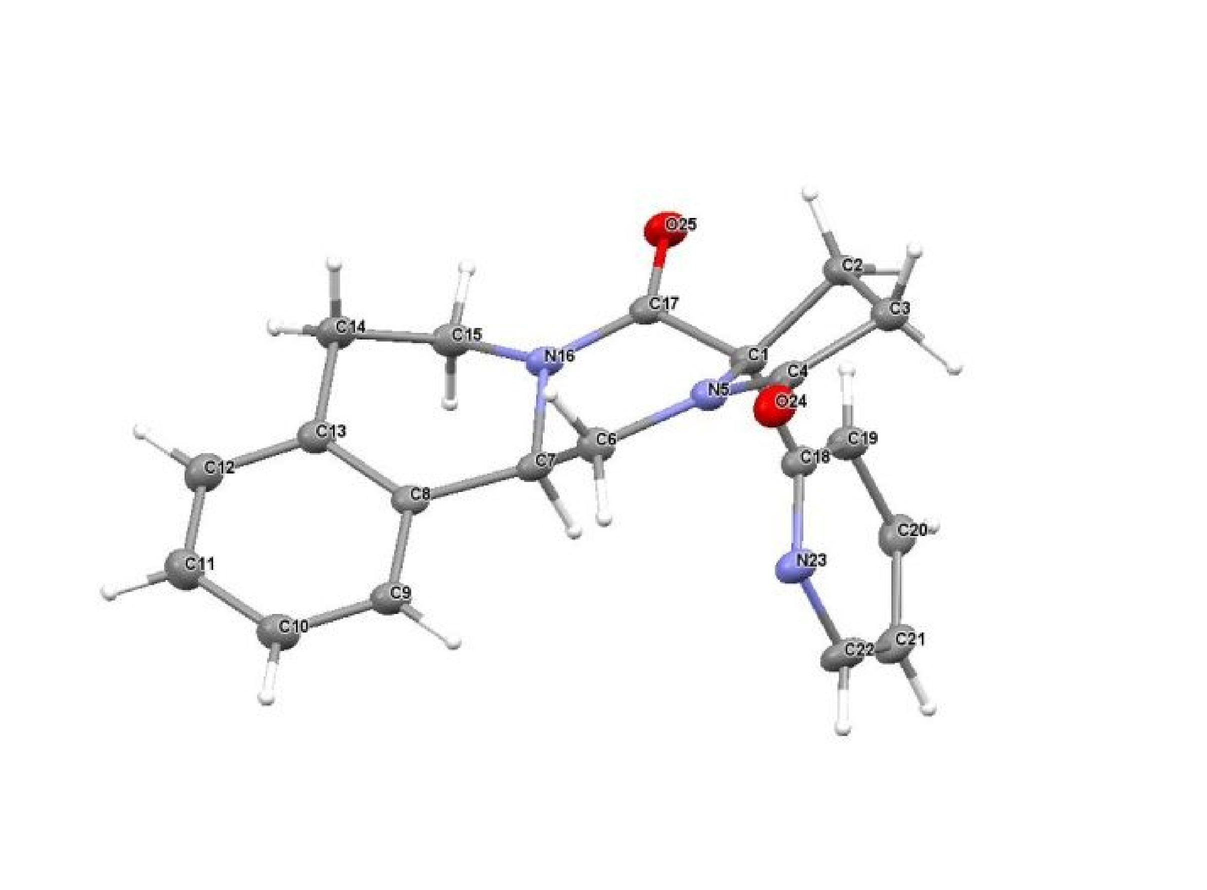

Figure S3. Molecular geometry observed in the crystal structures of compound **7a** (BDH_00) (asymmetric unit), showing the atom labelling scheme. The positional disorder within the benzene ring is observed with equal site occupancy (50:50). Displacement ellipsoids of non-hydrogen atoms are drawn at the 30% probability level. H atoms are presented as small spheres with an arbitrary radius.

Table S2. Crystal data and structure refinement results for compound **6a** and **7a**.

	**6a**	**7a**
Empirical moiety formula	$C_{20} H_{19} N_3 O_2$	$C_{20} H_{19} N_3 O_2$
Formula weight [g/mol]	333.38	333.38
Crystal system	Monoclinic	Triclinic
Space group	$P2_1/n$	$P\bar{1}$
Unite cell dimensions	a = 12.35225(14) Å<br>b = 9.56319(12) Å<br>c = 27.9380(3) Å<br>α=90°<br>β=91.1698(10)°<br>γ=90°	a = 9.1187(2) Å<br>b = 9.46413(16) Å<br>c = 11.1547(2) Å<br>α=67.7203°<br>β=66.604(2)°<br>γ=100.01°
Volume [Å^3]	3299.55(7)	810.64(3)
Z	8	2
D_{calc} [Mg/m^3]	1.342	1.366
μ [mm^{-1}]	0.712	0.725
F(000)	1408	352
Crystal size [mm^3]	0.2 x 0.2 x 0.15	0.5 x 0.4 x 0.15
Θ range	3.16° to 77.32°	5.09° to 80.03°
Index ranges	-14 ≤ h ≤ 15,<br>-11 ≤ k ≤ 12,<br>-35 ≤ l ≤ 35	-11 ≤ h ≤ 11,<br>-12 ≤ k ≤ 12,<br>-14 ≤ l ≤ 14
Refl. collected	47518	16409
Independent reflections	6912<br>[R(int) = 0.0483]	3350<br>[R(int) = 0.1148]
Completeness [%] to Θ	99.9 (Θ 71.98°)	98.9 (Θ 71.9°)
Absorption correction	Multi-scan	Multi-scan
Tmin. and Tmax.	0.897 and 1.000	0.897 and 1.000
Data/ restraints/parameters	6912 / 0 / 2452	3350 / 0 / 2327
GooF on F2	1.103	1.145
Final R indices [I>2sigma(I)]	R1= 0.0450,<br>wR2= 0.1176	R1= 0.0664,<br>wR2= 0.1938
R indices (all data)	R1= 0.0422,<br>wR2= 0.1221	R1= 0.0642,<br>wR2= 0.1886
$\Delta\rho_{max}$, $\Delta\rho_{min}$ [e·Å^{-3}]	0.44 and -0.32	0.45 and -0.43

References:

[S1] Rigaku-Oxford Diffraction; CrysAlisPro Oxford Diffraction Ltd, Abingdon, England V 1. 171. 36. 2. (release 27-06-2012 CN (2006)

[S2] Palatinus, L. Chapuis, G., J. Appl. Cryst. 2007, 40, 786.

[S3] Sheldrick, G. M. ActaCryst. 2008, A64, 112-122.

[S4] Farrugia, L., J. J. Appl. Cryst. 1999, 32, 837-838.

[S5] Macrae C. F., Edgington P.R., McCabe P., Pidcock E., Shields G.P., Taylor R., Towler M., & van de Streek J., J. Appl. Cryst. 2006, 39, 453-457.
